# Spatial spillover effects of urban innovation on productivity growth: A case study of 108 cities in the Yangtze River Economic Belt

**DOI:** 10.1371/journal.pone.0294997

**Published:** 2023-12-21

**Authors:** Zhengtao Li, Zhixian Chai, Laihe Ren

**Affiliations:** School of Economics, Zhejiang University of Finance and Economics, Hangzhou, China; Soochow University, CHINA

## Abstract

The spatial spillover of urban innovation has increasingly become an important factor affecting urban total factor productivity (TFP) growth. Based on the panel data of 108 cities in the Yangtze River Economic Belt from 2004 to 2020, this paper illustrates the spatial-temporal evolution trend of urban innovation and manufacturing total factor productivity and uses the spatial Dubin model to study the direct and indirect effects of urban innovation on manufacturing total factor productivity. The main conclusions are as follows:(1)Spatial correlation and heterogeneity between urban innovation and manufacturing total factor productivity prove to be significant in the Yangtze River Economic Belt;(2)Urban innovation has a significant positive impact on manufacturing total factor productivity and has a positive spillover effect on surrounding cities; (3)The effect of urban innovation on manufacturing total factor productivity varies with time and region;(4) Mechanism analysis shows that talent agglomeration significantly promotes the positive impact of urban innovation on manufacturing total factor productivity, while the spillover effect is insignificant.

## 1. Introduction

In the recent 20 years, China’s urbanization rate has increased rapidly from 41.8% in 2003 to 63.9% in 2020. Accordingly, the urban innovation ability also grows at high speed and becomes an important driver of productivity growth. The number of patent grants experience a volume explosion from 158,972 in 2003 to 1,699,193 in 2020, with an average annual growth rate of 14.1%. The increasing urban agglomerations make their influence total factor productivity beyond the border through spatial spillovers. While spatial spillover tends to show significant spatial dependence [1], the role of innovation-driven productivity becomes more complex. In recent years, the growth of total factor productivity of the Chinese manufacturing industry has decreased, and its contribution to economic growth has also declined [2]. What about the impact of urban innovation on manufacturing total factor productivity? From the perspective of spatial interaction, to what extent does the growth of manufacturing total factor productivity benefit from the improvement of its own innovation ability and to what extent does it benefit from the spillover effect of innovation in neighboring cities? From a knowledge perspective, what role does the intermediate variable such as talent aggregation play in this process? In order to solve the above problems, this paper conducts a spatial econometric analysis based on the panel data of 108 cities in the Yangtze River Economic Belt from 2004 to 2020. we will first study the spatial correlation between urban innovation and manufacturing total factor productivity, and then we will analyze the spillover effects of urban innovation on manufacturing TFP in more detail, lastly, we will give some discussion about the specific path for urban innovation to influence TFP using talent agglomeration to indicate intermediate effects test.

Early pieces of literature make a large number of studies on innovation and productivity from the enterprise [3, 4] and industry levels [5, 6], focusing on the contribution of input or output of innovation to productivity with fruitful conclusions [7–9]. However, the effects of the space factor are relatively ignored, while spatial proximity deriving from regional integration affects the spatial distribution pattern of innovation output [10]. Scholars generally recognize the importance of the spatial distribution of innovation or total factor productivity [11] and start to add spatial factors into their research. The spatial correlation and dependence of regional innovation have also been recognized [12]. Anselin et al. (1997) find that the spillover effect of university R&D level on innovative activities is significant [13]. Yi et al. (2018) measured and decomposed the patent total factor productivity of high-tech industries in China during 1998–2015 based on the Malmquist index, and find that the average patent total factor productivity of high-tech industries shows increasing trends, but the index has spatial heterogeneity among provinces [14]. Zhang et al. (2010) find that foreign R&D capital has a significant positive technology spillover effect on high-tech enterprises, while local R&D plays an important role in improving the total factor productivity of low and medium-tech enterprises [15]. Jiao (2018) uses the provincial data of China from 1998 to 2004 to build a regional TFP growth model that includes both the scale of R&D investment and the allocation of R&D resources, and find that the two variables have obvious spatial agglomeration characteristics, and R&D investment has a significant inhibitory effect on the TFP of local and spatially related regions [16].

By the way, the closest to this article is the article by Kijek and Matras Bolibok (2019) [11]. The author uses the cross-sectional data of the EU region in 2015 and uses a spatial lag model. An empirical study finds that regional innovation performance has a positive impact on total factor productivity. As the author said in the article, "At the regional level, there is a lack of empirical research on the relationship between innovation and total factor productivity." And it is worth exploring whether the spatial spillover effects are consistent in different regions. Compared with the existing research, the text’s fringes are contributed to two points: First, spatial spillover is taken into account to analyze the effects of urban innovation on the TFP of the manufacturing industry. Second, the regulatory effect of urban innovation on manufacturing TFP has been identified from the perspective of talent agglomeration.

## 2. Theoretical analysis and research hypothesis

### 2.1 Spatial heterogeneity analysis of urban innovation

Urban innovation is an important driving force for manufacturing TFP growth, including direct and indirect effects. The direct effect includes three levels: from the perspective of enterprises, innovation-oriented human capital, physical capital, etc., through R&D expenditure and introduction of new products and processes, improve the added value of products or reduce costs, to promote efficiency growth [17]. From the perspective of industry, urban innovation improves coordination through upstream and downstream industry chains based on input-output relationships, drives the upgrading of industrial structure, and promotes the improvement of TFP [18]. From the perspective of the government, the effects of factor substitution, innovation compensation, and resource relocation can jointly influence manufacturing TFP and have heterogeneous impacts on the TFP of different industries [19].

In the context of regional integration, spatial interaction, and indirect effect becomes more important. Cities play a dual role as producers and absorbers of innovation, promoting knowledge spillover through human capital, R&D cooperation, and policy coordination. Not only can innovation input affect the economic efficiency of neighboring regions through spatial spillover [20], but the cross-regional flow of new products along the industry chain also causes the spillover of embedded innovation knowledge. With the deepening of functional differentiation and industrial division within urban agglomerations, such spillover effect will become stronger, which is manifested in the central city to different node cities. The size of the indirect effect of spillover often depends on the absorptive capacity of the target city. The stronger the absorption capacity, the stronger the effect of innovation spillover.

As the Yangtze River Economic Belt spans the eastern, central, and western parts of China, there are big differences in factor endowment, marketization process, human capital, and policy system among different regions, which affect the diffusion and development of innovation capability in each region. From the perspective of factor endowment, when the innovation mode of the city is compatible with the economic development status, endogenous innovation based on independent research and development can promote the improvement of TFP [21]. From the perspective of the marketization process, different regions have significant differences in economic denationalization, product market, factor market, etc., leading to a different performance of economic development mode, thus affecting the growth of total factor productivity [22]. From the perspective of the institutional environment, local laws and regulations, supervision mechanisms, and property rights mechanisms are inconsistent. Regions with a poor institutional environment have insufficient input in independent innovation and low total factor productivity. Moreover, restrictions on the existing household registration system and factor flow mechanism will also lead to the widening of the inter-regional gap and exacerbate the gap in innovation output between different cities. Hindering the improvement of total factor productivity [17]. So we propose the following hypothesis:

H1: Urban innovation can promote the improvement of manufacturing total factor productivity, but regional heterogeneity exists.
H2: Urban innovation can affect the total factor productivity of the manufacturing industry in surrounding cities through the spillover effect, and regional heterogeneity also exists.

### 2.2 The regulatory effect of talent agglomeration

Talent agglomeration, with higher knowledge level, is manifested both in the accumulation of innovative talent resources such as scientific research institutions and universities in a specific region, and in the collaborative interaction of talents within the scope of agglomeration, which will bring significant agglomeration effect and promote the effects of innovation on manufacturing TFP [23]. This agglomeration effect affects urban innovation mainly through the matching mechanism and learning effect. On the one hand, the gathering of urban innovative talents within a certain range can strengthen the interaction and matching with talents of the same or different specialties in terms of professional skills and knowledge sharing, to break through the time-space limitation of knowledge transmission to a greater extent, enhance the scope and frequency of face-to-face communication, promote the dissemination of innovation consciousness and innovation ability, and expand the sources of innovation knowledge. So as to improve the number of innovation results, and promote the level of economic efficiency. On the other hand, agglomeration tends to build up an innovation environment, an atmosphere of innovation cooperation, and the improvement of talents’ skill level, which can be realized through the matching mechanism and interactive learning, so as to stimulate talents’ innovation willingness, reduce the risk cost of innovation activities, improve R&D efficiency and the transformation of innovation results [24], which is conducive to the growth of total factor productivity.

H3: Talent agglomeration positively moderates the positive effect of urban innovation on manufacturing TFP

## 3. Research method

### 3.1 Model

To investigate the impact of urban innovation on the TFP of the manufacturing industry, the following benchmark panel measurement model is set in combination with the theoretical mechanism of innovation-driven total factor productivity:

$${\mathrm{Tfp}}_{\mathrm{i,t}}=\alpha_{0}+\alpha_{1}{\mathrm{Innov}}_{\mathrm{i,t}}+\alpha_{2}Z_{\mathrm{i,t}}{+\tau }_{i}{+\varphi }_{t}{+\varepsilon }_{\mathrm{i,t}}$$ (1)

Considering the possible spatial spillover effect of urban innovation on total factor productivity. In this paper, the time-space dual fixed effect Dubin model is set to test the impact of innovation on manufacturing total factor productivity in 108 cities in the Yangtze River Economic Belt. The spatial econometrical model is set as follows:

$${\mathrm{Tfp}}_{\mathrm{i,t}}{=\rho \mathrm{WTfp}}_{\mathrm{i,t}}{+\beta }_{1}{\mathrm{Innov}}_{\mathrm{i,t}}{+\beta }_{2}{\mathrm{WInnov}}_{\mathrm{i,t}}{+\gamma X}_{\mathrm{i,t}}{+\tau }_{i}{+\varphi }_{t}{+\varepsilon }_{\mathrm{i,t}}$$ (2)

where *i* represents individual cities in the Yangtze River Economic Belt, *t* denotes the year, and *Tfp*_*i*,*t*_ is the total factor productivity of the manufacturing industry, which measures the development level of the manufacturing industry of a city. *Innov*_*i*,*t*_ is the core explanatory variable to measure the level of urban innovation. *τ*_*i*_ and *φ*_*t*_ are individual effects and time effects respectively, *ε*_*i*,*t*_ is the random disturbance term; *W* is an *N* by *N* space weight matrix. In order to more objectively reflect the spatial effect of urban innovation on the TFP of the manufacturing industry, two spatial weight matrices are selected for spatial econometric analysis in this paper, namely the 0–1 adjacency matrix and inverse distance matrix. Assumptions used *l*_*ij*_ to represent the length of the border, spatial weight matrix based on Rook proximity of is defined as:

$$W_{\mathrm{ij}}=\{\begin{array}{c} 1,\;l_{\mathrm{ij}}>0\\ 0,\;l_{\mathrm{ij}}=0 \end{array}$$ (3)

According to the first law of geography, the spatial interaction between space units farther away will be smaller. So the distance between spatial units to represent the decline of a spatial interaction effect, local latitude and longitude information from city to city is calculated by the cities the geographic distance between *d*_*ij*_, is based on geographic distance space weight matrix is defined as follows:

$$W_{\mathrm{ij}}=\{\begin{array}{c} \frac{1}{d_{\mathrm{ij}}},{<d}_{\mathrm{ij}}\leq d\\ 0,\;{d<d}_{\mathrm{ij}} \end{array}$$ (4)

### 3.2 Variables and data

#### 3.2.1 Variable selection and description

Explained variable: manufacturing total factor productivity (TFP). In this paper, the DEA-Malmquist index is adopted, based on the data from 2003 to 2020. In the measurement, the labor force variable is measured by the total number of employees per unit at the end of the year, and the capital stock is calculated by referring to the approach of Zhang (2004) [25] and using the perpetual inventory method. The specific calculation method is *K*_*t*_ *= (1-d) K*_*t-1*_ *+I*_*t*_, in which, *K*_*t*_ and *I*_*t*_ are the capital stock and investment in *t* years respectively. *d* is the depreciation rate. The initial capital stock is expressed by dividing the actual fixed asset investment in 2003 by 10%, and the depreciation rate is expressed by 9.6%. The output indicator is expressed as the adjusted value added of manufacturing in each city.

Core explanatory variable: Innovation level (Innov). The number of patents can truly measure the level of urban innovation [26, 27]. Currently, the number of urban patent applications or grants is widely used by scholars to measure the output of urban innovation. Compared with the number of patent applications, the number of patent grants can better reflect a city’s innovation quality and technological application. Therefore, this paper uses the number of patents granted by 10,000 people as the proxy variable of urban innovation ability. At the same time, the number of 10,000 patent applications is used to test robustness.

Moderating variable: Talent concentration. Location entropy is a commonly used method to measure agglomeration degree and specialization level [28]. This paper uses location entropy to calculate the level of talent agglomeration(see Formula 5), where *M*_*i*,*t*_, *M*_*t*_, *N*_*i*,*t*,_ and *N*_*t*_ respectively represent the number of teachers in colleges and universities of the city *i* in year *t*, the total number of teachers in colleges and universities of 108 cities in the Yangtze River Economic Belt in year *t*, unit employees of the city *i* in year *t*, and all unit employees of 108 cities in the Yangtze River Economic Belt in year *t*.

$${\mathrm{talent}}_{\mathrm{i,j}}=\frac{M_{\mathrm{i,t}}{/M}_{t}}{N_{\mathrm{i,t}}{/N}_{t}}$$ (5)

Control variables: technical level (*Ptech*), city size (*Popu*), education level (*Edu*), urban infrastructure level (*Road*), openness level (*Open*), economic development degree (*Pgdp*), industrial structure (*Ind*), and government intervention degree (*Gov*) are selected as control variables. The technology level is measured by the per capita expenditure on science and technology, and the city size is expressed by the total population at the end of the year. Education level is expressed by the number of urban colleges and universities; Urban infrastructure level is expressed by per capita road area; The level of opening up is expressed by the actual use of foreign capital; The level of economic development is expressed by the per capita GDP of the city. The industrial structure is expressed by the proportion of the added value of secondary industry and tertiary industry; The extent of government intervention is expressed as the proportion of fiscal revenue to GDP. In order to eliminate the effect of heteroscedasticity, the natural logarithm of all variables is used.

#### 3.2.2 Descriptive analysis

This paper uses panel data from 108 cities in 11 provinces (including municipalities directly under the Central Government) of the Yangtze River Economic Belt from 2004 to 2020. All data are from the officially published China Statistical Yearbook, China Urban Statistical Yearbook, China Regional Economic Statistical Yearbook, China Urban Construction Statistical Yearbook, provincial and municipal statistical Yearbook, prefecture-level municipal Statistical Yearbook and national economic and social development Statistical Bulletin, etc. For the very small number of missing data, this paper uses the method of authoritative literature to fill in the missing data by interpolation. GDP, industrial added value, and other data involved in this paper are adjusted according to the price index based on 2003. The descriptive statistics of each variable after processing are shown in Table 1. The minimum value and maximum value of Lntfp in manufacturing are 0.646 and 0.847, indicating that there is a large gap between the total factor productivity of manufacturing in different cities. The minimum value of urban innovation (Lninnov) is 0.011, and the maximum value is 5.234, showing obvious differences. The results of the other control variables are within the appropriate range. The correlation test shows that there is no multicollinearity problem in this paper, and the specific results are shown in S1 Table.

**Table 1:** Descriptive statistics of variables.

Variable	obs.	Mean	Median	Std.Dev	Min	Max
Lntfp	1,836	0.727	0.723	0.025	0.646	0.847
Lninnov	1,836	1.508	1.202	1.207	0.011	5.234
Lntalent	1,836	0.553	0.485	0.322	0	1.766
Lnptech	1,836	13.028	13.23	1.867	8.054	17.974
Lnpopu	1,836	6.01	6.129	0.624	3.93	8.136
Lnedu	1,836	10.55	10.477	1.311	5.442	13.881
Lnroad	1,836	2.699	2.693	0.605	0.438	11.188
Lnopen	1,836	11.813	11.916	2.027	3.989	16.452
Lnpgdp	1,836	9.918	9.876	0.798	7.872	12.303
Lnind	1,836	1.531	1.535	0.525	0.47	3.589
Lngov	1,836	0.101	0.091	0.051	0.006	0.338

## 4. Spatial analysis

We use ArcGIS10.7 software to draw the spatiotemporal trends in 2005, 2010, 2015, and 2020, focusing on the spatial agglomeration and spatiotemporal evolution trend of the total productivity of the manufacturing industry and urban innovation.

### 4.1 Spatial and temporal evolution of urban innovation

**Fig 1:**
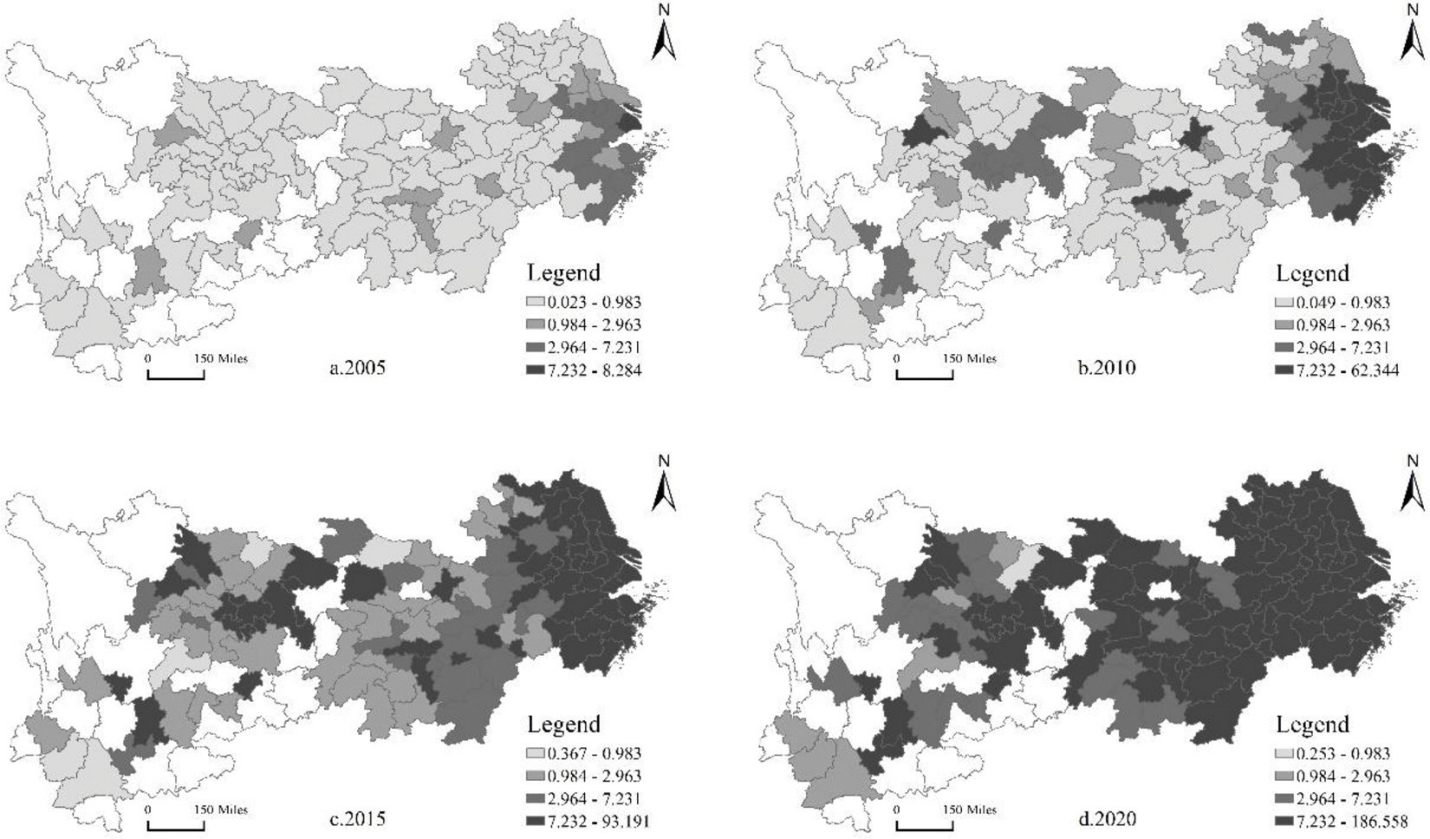
Spatial and temporal evolution trend of urban innovation in the Yangtze River Economic Belt. Source of base map: The open source map data service provided by the National Platform for Common GeoSpatial Information Services (https://www.tianditu.gov.cn).

Fig 1 shows the spatial and temporal evolution of the innovation level of urban agglomerations in the Yangtze River Economic Belt represented by the number of patents granted by 10,000 people. On the whole, the urban innovation level among different regions of the Yangtze River Economic Belt shows an increasing trend and spatial agglomeration and radiation expansion with time. Meanwhile, there is obvious spatial heterogeneity. It is reflected in the difference in innovation levels among different regions and cities in the Yangtze River Economic Belt. In 2005, only Shanghai’s innovation level was in the highest digit range of 7.232–8.284, while a few cities, such as Hangzhou, Suzhou, and Nanjing, were in the second digit range of 2.964–7.231. The number of patents granted by 10,000 people in the cities of the Yangtze River Economic Belt is at a low level, showing a phenomenon of low and low agglomeration. In 2010, except for Wuhan and Changsha in the middle reaches and Chengdu in the upper reaches, the number of patent grants in Anhui, Zhejiang, Jiangsu, and Shanghai in the inner provinces of the Yangtze River Delta economic area is significantly higher than that in the middle and upper reaches of the Yangtze River Economic Belt and shows a spatiotemporal characteristic of gradually accumulating to the east as time goes. Meanwhile, urban innovation shows obvious spatial heterogeneity in the Yangtze River Economic Belt. In 2015, the innovation level of all cities increase significantly. Except for Xiangyang in the middle reaches, Zhaotong in the upper reaches, and Lincang and Pu ‘er in the lowest quantile, most of the other cities were in the first two quantiles, and the innovation agglomeration phenomenon of downstream cities became increasingly prominent. In 2020, the number of patents granted in downstream cities is above 7.232, and more than half of the cities in the whole Yangtze River Economic Belt are in the highest digit range. At this time, the agglomeration phenomenon of high and medium-high urban innovation is more significant. In addition, the innovation level of the Chengdu-Chongqing economic zone also achieves significant progress, gradually forming an innovation highland with Chongqing and Chengdu as the core.

### 4.2 Spatio-temporal evolution of manufacturing TFP

**Fig 2:**
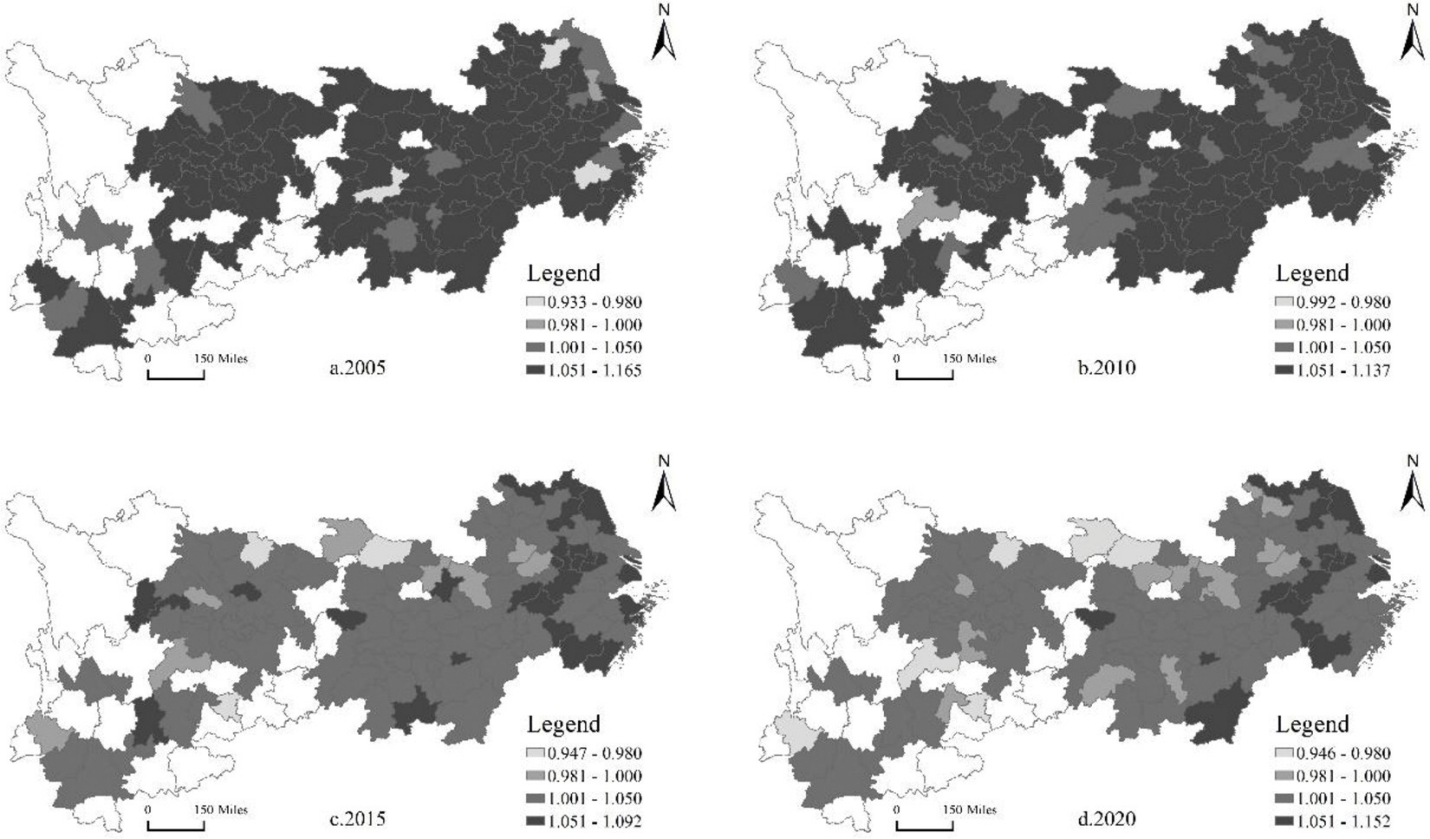
Spatial and temporal evolution trend of the TFP of the manufacturing industry in the Yangtze River Economic Belt. Source of base map: The open source map data service provided by the National Platform for Common GeoSpatial Information Services (https://www.tianditu.gov.cn).

By constructing the input-output index of the manufacturing TFP, we use the Data Envelopment Analysis method to estimate the manufacturing TFP of 108 cities in the Yangtze River Economic Belt from 2004 to 2020, and the spatial-temporal evolution trend is shown in Fig 2. On the whole, the TFP of the manufacturing industry shows a trend of slow decline over time, which is consistent with the research of Liu et al. [29] and Li et al. [30], and the TFP of the manufacturing industry in different cities also reflect spatial heterogeneity. In 2005, except for a few cities such as Yiyang City and Suqian City, the TFP of the manufacturing industry in most cities is in the highest digit range of 1.051–1.165, and so is that in 2010. Higher productivity is closely related to China’s economic development stage: before 2010, China’s economy maintained high-speed growth for several years. As can be seen from Table 2, the growth rates of manufacturing output in the three regions of the Yangtze River Economic Belt are significantly higher than that of capital and labor input. After 2010, the growth rate of input and output declines, and the TFP of manufacturing industry declines as well, which is similar to the conclusion of Li et al. [31]. The possible reason is that the large-scale economic stimulus policies implemented in China after the financial crisis, however, the extensive economic stimulus policies do not promote high-quality economic development. In addition to Shanghai, Nanjing, and Ningbo in the downstream and a few cities in the middle and upstream such as Wuhan and Kunming, the TFP of manufacturing industries in most cities are in the second-highest score range of 1.001–1.050. Regional agglomeration shows the spatial characteristics of the sparse distribution of the highest score and highly concentrated second-highest score. The TFP of some downstream cities is higher than that of upstream and middle cities.

**Table 2:** Input-output growth rate and total factor productivity of the manufacturing industry.

Region	The growth rate of manufacturing output (%)	The growth rate of capital input (%)	The growth rate of labor input (%)	Total factor productivity in manufacturing
downstream	11.29	8.52	4.82	1.08
midstream	13.98	16.24	3.91	1.065
upstream	10.58	12.22	5.93	1.064
Mean value	11.95	12.33	4.89	1.07

Note: All input-output growth rates are calculated based on 2003.

## 5. Empirical analysis

### 5.1 Spatial econometric analysis

#### 5.1.1 Spatial autocorrelation

Before using the spatial econometric method to analyze the spatial spillover effect of urban innovation on manufacturing total factor productivity, the spatial autocorrelation among 108 cities in the Yangtze River Economic Belt should be tested first. Moran’s I index is a common statistic for testing global spatial autocorrelation, and its calculation formula is as follows:

$$Moran'sI\frac{N\sum_{i=1}^{N}\sum_{j=1}^{N}\omega_{ij}(x_{i}‐\bar{x})(x_{j}‐\bar{x})}{S\sum_{i}^{N}{\left(x_{i}‐\bar{x}\right)}^{2}}$$ (6)

Where, $S=\Sigma_{i=1}^{N}\Sigma_{j=1}^{N}\omega_{\mathrm{ij}}$ represents the sum of all elements of the space weight matrix. *N* is the Number of representation space units, *x*_*i*,_ and *x_j_* respectively variable x in the first the i and j of observation values, $\bar{x}$ is the mean of the variable x, *ω_ij_* said the spatial weight matrix of the element. A value greater than zero means positive spatial autocorrelation, while a value less than zero means negative spatial autocorrelation. The closer the value is to 1, the higher the degree of spatial agglomeration; the closer it is to -1, the higher the degree of spatial dispersion.

As can be seen from Table 3, for both forms of the spatial weight matrix, the global Moran’s I index of manufacturing TFP and urban innovation is significantly positive at a 1% level. The comparison of coefficient shows that the coefficient size difference between the two weight matrices is obvious. The spatial autocorrelation coefficient between urban innovation and manufacturing TFP under the adjacency matrix is significantly higher than that under the geographical distance weight matrix. Moran’s I index of manufacturing TFP generally decreases and then increases over time, while Moran’s I index of urban innovation generally fluctuates and increases. The above results show that there is an obvious positive spatial correlation between them, but the specific relationship needs further spatial econometric analysis.

**Table 3:** TFP and Innov Global Moran’s I index for the Yangtze River Economic Belt.

Year	Adjacency matrix		Inverse distance matrix	
	TFP	Innov	TFP	Innov
2004	0.231***	0.210***	0.058***	0.051***
2005	0.422***	0.214***	0.07***	0.056***
2006	0.262***	0.210***	0.037***	0.063***
2007	0.236***	0.235***	0.025***	0.070***
2008	0.183***	0.226***	0.021***	0.067***
2009	0.174***	0.214***	0.033***	0.068***
2010	0.183***	0.261***	0.049***	0.080**
2011	0.180***	0.232***	0.050***	0.071***
2012	0.246***	0.241***	0.080***	0.072***
2013	0.156***	0.265***	0.045***	0.073***
2014	0.240***	0.353***	0.058***	0.095***
2015	0.250***	0.355***	0.067***	0.096***
2016	0.271***	0.355***	0.078***	0.098***
2017	0.307***	0.365***	0.106***	0.098***
2018	0.286***	0.376***	0.102***	0.103***
2019	0.331***	0.378***	0.133***	0.099***
2020	0.263***	0.259***	0.106***	0.078***

Note: ***, **, and * respectively indicate that they pass the significance test at the level of 1%, 5%, and 10%, the same as below.

#### 5.1.2 Test of spatial econometrical model

Since the traditional econometric model cannot consider spatial autocorrelation and spatial spillover effect, we use the spatial econometric model to test the effect of urban innovation on manufacturing productivity. In order to avoid estimation bias caused by the spatial error model (SEM) or spatial lag model (SLM) in the case of missing variables, this paper uses the spatial Durbin model (SDM) for empirical study.

According to Table 4, under the two spatial weight matrices, LM-Lag and LM-Error result both significantly reject the null hypothesis, indicating that the spatial Dubin model should be used. The results of the Hausman test are all significant at the 1% level, indicating that a fixed effect should be selected. The LR statistic is significant, and the spatiotemporal double fixed effect should be selected. In order to determine whether the SDM model will degenerate into SEM and SAR models, the LR test and Wald test are carried out, the test results are shown in Table 5.

**Table 4:** LM test, LR test, and Hausman test.

Test statistic	Rook matrix	Inverse distance matrix
LM-Lag	345.564***	935.963***
R-LM-Lag	284.554***	490.806***
LM-Error	134.123***	601.653***
R-LM-Error	73.114***	156.496***
Hausman test	84.69***	59.31***
ind nested in both	85.36***	24.58***
time nested in both	1459.03***	1437.07***

**Table 5:** LR test and Wald test of spatial Dubin model.

Test statistic	rook matrix	Inverse distance matrix
LR_Lag	54.06***	68.87***
LR_Errow	65.50***	46.50***
WALD_Lag	7.62	7.89
WALD_Errow	8.47	7.97
Log-likelihood_sdm	5993.86	5991.18
Log-likelihood_sdr	5985.51	5981.83
Log-likelihood_sem	5983.97	5981.47

For the two matrices, the LR test is significant, while the Wald test is insignificant. The log-likelihood value of the spatial Dubin model under the two weight matrices is greater than those of the spatial error model and the spatial lag model, so we use the spatial Durbin model (SDM) with spatiotemporal dual fixed effects for estimation.

#### 5.1.3 Regression results

In this section, the spatial Durbin model (SDM) is further used to analyze spillover effects, and the fixed effects model (Fe) is used as a reference for testing. The regression results are shown in Table 6. (1)~(4) are estimation results of the spatial Dubin model, and (5) and (6) are estimation results of the fixed effect model.

**Table 6:** Spatial regression results of effects of urban innovation on manufacturing TFP.

variable	Adjacency matrix		Inverse distance matrix		Fe	
	(1)Lntfp	(2)Lntfp	(3)Lntfp	(4)Lntfp	(5)Lntfp	(6)Lntfp
Lninnov	0.0031***	0.0056***	0.0037***	0.0065***	0.0054***	0.0077***
	(4.18)	(6.82)	(4.84)	(7.92)	(7.40)	(9.32)
WLninnov	0.0072***	0.0066***	0.0234***	0.0207***		
	(6.15)	(4.76)	(5.06)	(2.92)		
rho	0.1232***	0.0959***	0.2154*	0.0415		
	(3.89)	(3.00)	(1.85)	(0.31)		
sigma2_e	0.0001***	0.0001***	0.0001***	0.0001***		
	(30.24)	(30.26)	(30.28)	(30.30)		
Control variable	NO	YES	NO	YES	NO	YES
Regional effect	YES	YES	YES	YES	YES	YES
Time effect	YES	YES	YES	YES	YES	YES
*N*	1836	1836	1836	1836	1836	1836
*R* ^2^	0.2455	0.08	0.4941	0.0009	0.8052	0.8165

Without spatial factors taken into consideration, urban innovation has a significant positive relationship with the TFP of the manufacturing industry, which verifies H1. Every 1% increase in urban innovation will increase the total factor productivity of the manufacturing industry by 0.008%. Under the adjacent-weight weight matrix, the ρ of the spatial autocorrelation coefficient of manufacturing total factor productivity is significantly positive regardless of whether control variables are added. under the inverse distance weight matrix, the ρ of the spatial autocorrelation coefficient without control variables is significantly positive at the significance level of 10%, while the ρ of the spatial autocorrelation coefficient with control variables is positive but not significant. The above results can further confirm Moran’s I test result that the TFP of the manufacturing industry has a positive spatial correlation, that is, the TFP of the manufacturing industry in local cities will be affected by the spillover effect of surrounding cities, which verifies H2. The estimated coefficients of urban innovation in models (1)~(4) are significantly positive, and the influence degree is similar to that of the fixed effect model, which proves that the growth of urban manufacturing TFP will be positively affected by its innovation level. The spatial lag variable (Wlninnov) of urban innovation is also significantly positive, that is, the development of the innovation level of the city itself will promote the growth of the TFP of the surrounding city. The possible reason may be that the inter-regional movements of talents, technology, and cross-regional cooperation of research and development will make the innovation of cities benefit the productivity of surrounding cities.

#### 5.1.4 Decomposition effects of spatial Dubin model

In the SDM model, the influence of independent variables on the dependent variables cannot be simply expressed by the regression coefficient. LeSage and Pace (2008) divide the influence of independent variables on dependent variables into direct effect, indirect effect, and total effect according to the different scopes and objects of spatial effect [32]. The indirect effect is an important research topic in spatial econometric models, also known as a spatial spillover effect. Table 7 reports the decomposition effects of the spatial Dubin model under two weight matrices. From the perspective of direct effect, urban innovation has a significant positive impact on the total factor productivity of the manufacturing industry, that is, every 1% increase in the level of urban innovation will increase the total factor productivity of the manufacturing industry by about 0.006%-0.007%, and the degree of impact is close to the estimated coefficient obtained by using the fixed effect model in Table 6. From the perspective of indirect effect, the spatial spillover effect of urban innovation on the total factor productivity of the manufacturing industry is significantly positive, indicating that there is a positive spatial correlation between the total factor productivity of the urban manufacturing industry in the Yangtze River Economic Belt, that is, the growth of urban total factor productivity not only depends on the improvement of regional urban innovation level. The urban innovation level of neighboring cities and other cities will also have a positive spillover effect on the total factor productivity of the local manufacturing industry through spatial linkage. It can be seen from the ratio of indirect effect and total effect that the spatial spillover effect of urban innovation accounts for more than 50% of the total growth effect, indicating that the spatial spillover effect is greater than the direct effect. This indicates that in the Yangtze River Economic Belt, with the improvement of regional integration and inter-city cooperation, urban development is increasingly influenced by other cities under the role of space. The movements of innovation factors and the positive interaction of technology research and development are important basis to promote the improvement of urban total factor productivity.

**Table 7:** Decomposition effect of spatial Dubin model.

variable	Adjacency matrix			Inverse distance matrix		
	(1) Direct effect	(2) Indirect effect	(3) Total effect	(4) direct effect	(5) Indirect effect	(6) Total effect
Lninnov	0.0058***	0.0078***	0.0136***	0.0066***	0.0224***	0.0289***
	(6.98)	(5.21)	(8.72)	(7.80)	(2.68)	(3.51)
Lnptech	-0.0032***	-0.0005	-0.0038***	-0.0033***	-0.0007	-0.0039
	(-5.56)	(-0.49)	(-3.21)	(-5.61)	(-0.10)	(-0.60)
Lnpopu	-0.0021	0.0094**	0.0074*	-0.0009	0.0414	0.0405
	(-0.97)	(2.27)	(1.71)	(-0.43)	(1.49)	(1.45)
Lnedu	0.0030***	0.0015	0.0044**	0.0029***	-0.004	-0.0011
	(3.45)	(0.98)	(2.42)	(3.33)	(-0.40)	(-0.11)
Lnroad	-0.0011**	-0.0017*	-0.0028***	-0.0012**	-0.0058	-0.007
	(-2.02)	(-1.65)	(-2.62)	(-2.16)	(-1.27)	(-1.54)
Lnopen	-0.0001	-0.0012**	-0.0013*	0	-0.0101***	-0.0101***
	(-0.28)	(-1.98)	(-1.87)	(-0.02)	(-2.61)	(-2.58)
Lnpgdp	-0.0773***	0.009	-0.0683***	-0.0862***	0.0504	-0.0358
	(-6.19)	(0.35)	(-2.66)	(-6.92)	(0.41)	(-0.29)
Lnind	-0.0026	0.0177*	0.0151	-0.0041	0.0043	0.0002
	(-0.55)	(1.94)	(1.55)	(-0.86)	(0.10)	(0.00)
Lngov	-0.0101	0.0259	0.0158	-0.0076	0.066	0.0584
	(-0.93)	(1.13)	(0.64)	(-0.70)	(0.48)	(0.43)

### 5.2 Robustness and endogeneity

Replacing the spatial weight matrix, changing the measurement method of the core explanatory variable, and changing the estimation method are used respectively to test the robustness of the benchmark regression results.

Since the above studies are all based on spatial geographical distance factors to construct the spatial weight matrix, without considering the spatial correlation of economic activities, here we take the actual per capita GDP of each city as the representative object to construct the spatial weight matrix of urban economic distance in the Yangtze River Economic Belt. The calculation formula is shown in Eq 7, where *M*_*ij*_ is the economic distance matrix. *Pgdp*_*i*_ and *Pgdp*_*j*_ are the real per capita GDP of the city *i* and city *j*. The regression results are shown in S2 Table of the table. Whether control variables are added or not, the coefficient of urban innovation is significantly positive, and the coefficient size is not much different from the basic regression results above.


$$M_{\mathrm{ij}}=1/|\left({\mathrm{Pgdp}}_{i}{‐\mathrm{Pgdp}}_{j})/({\mathrm{Pgdp}}_{i}{+\mathrm{Pgdp}}_{j}\right)|$$ (7)

The number of patent applications per ten thousand people in cities is used to replace the number of patent grants per ten thousand people. The decomposition results of the Dubin model in S3 Table shows that the regression results under the two weight matrices are consistent with the benchmark regression results.

Regions with high manufacturing total factor productivity may have more advanced technology levels and a better innovation environment. Considering the endogeneity problem caused by bidirectional causality between an explanatory variable and explained variable, we introduce the spatial lag term of manufacturing total factor productivity into the original model [33, 35]. The regression results in S4 Table show that the positive relationship between urban innovation still exists significantly, and the spillover effect is obvious. All these substitution analyses indicate that the results of this paper are robust.

### 5.3 Heterogeneity

#### 5.3.1 Spatial decomposition effect in different periods

Considering the large research time from 2004 to 2020, it can be seen from the temporal and spatial trends that the urban innovation level and the total factor productivity of the manufacturing industry in the Yangtze River Economic Belt have undergone significant changes. Meanwhile, we pay more attention to the impact of urban innovation on the total factor productivity of the manufacturing industry in recent years. Therefore, the period from 2004 to 2020 is divided into two periods: 2004–2012 and 2013–2020. The reason for this division is mainly based on the following considerations: First, from the perspective of the history of China’s economic development, due to the continuous improvement and reform of the economic system, China’s economy has shown different development characteristics in different periods, so it is worth paying attention to study the impact of urban innovation on the total factor productivity and its spatial effect in different economic stages. Second, the 18th CPC National Congress at the end of 2012 proposed to "adhere to the path of independent innovation with Chinese characteristics and implement the innovation-driven development strategy". Therefore, 2013, the first year when the innovation-driven strategy began to be implemented, was taken as the beginning of high-quality development in the new era. The effects change of in urban innovation on the total factor productivity of the manufacturing industry also should reserve studied. Thirdly, from the perspective of sample size, with the year 2012 as a breakpoint, the sample sizes of the preceding and following periods are roughly equivalent, which will reduce the resulting bias and other problems caused by the huge gap in sample sizes.

SUR test (Suest) based on the seemingly uncorrelated model is conducted on the fixed panel data to verify whether there are significant differences in coefficients between groups. The results show that the Suest test value is 26.21 and significant at the level of 1%, so we reject the null hypothesis that there is no significant difference between the two groups, that is, it is reasonable to divide the period into two groups from 2004 to 2012 and from 2013 to 2020. Table 8 shows the decomposition effects of the spatial Durbin model in two periods, among which (1)~(4) are direct and indirect effects of spatial Durbin model decomposition from 2004 to 2012, and (5)~(8) are decomposition effects of spatial Durbin model from 2013 to 2020. It can be seen from the first four columns that, under the two weight matrices, the impact of urban innovation on the total factor productivity of the manufacturing industry is significantly positive. From the coefficient size, every 1% increase in urban innovation level will increase the total factor productivity of the manufacturing industry by 0.006%-0.007%. The indirect effect indicates that the improvement of urban total factor productivity is also influenced by the innovation level of surrounding cities, and the spillover effect is generally greater than the direct effect, which is consistent with the above results, indicating that from 2004 to 2012, the total factor productivity of urban manufacturing industry depends more on the spatial spillover effect of surrounding cities and neighboring cities. This may have something to do with the city’s lack of competitiveness and development pattern [23], urban development should be affected by the surrounding urban talent, resources, technology and other factors. The situation from 2013 to 2020 is different. From the last four columns, the direct effect of urban innovation on the total factor productivity of the manufacturing industry is negative and significant at a 10% level, while the indirect effect is positive and insignificant. The possible reason is that since 2012, all cities have been affected by talent competition, the transfer of foreign capital, and the technological restrictions of the trade disputes between China and the United States. As a result, the marginal cost of innovation factors keeps rising, and the ability to applicating innovation to production decreases, thus hindering the improvement of the overall productivity level of the manufacturing industry. The insignificant indirect effect may be due to the large development gap between big and small cities. The innovation capabilities improvement of big cities does not fully benefit small and medium-sized cities, and the spillover effect of innovation factors is also not significant.

**Table 8:** Decomposition effect of spatial Durbin model by period.

variable	Adjacency matrix		Inverse distance matrix		Adjacency matrix		Inverse distance matrix	
	(1) Direct effect	(2) Indirect effect	(3) Direct effect	(4) Indirect effect	(5) Direct effect	(6) Indirect effect	(7) Direct effect	(8) Indirect effect
Lninnov	0.0058***	0.0097***	0.0066***	0.0310**	-0.0012*	0.0012	-0.0013*	0.0051
	(3.81)	(3.53)	(4.27)	(2.38)	(-1.80)	(1.10)	(-1.95)	(1.32)
Lnptech	-0.0028***	-0.0008	-0.0026***	0.0005	-0.0006	0.0005	-0.0007	0.0057*
	(-2.80)	(-0.37)	(-2.59)	(0.05)	(-1.37)	(0.58)	(-1.50)	(1.91)
Lnpopu	-0.0341***	-0.0015	-0.0307***	0.1383	-0.0009	-0.0016	-0.001	-0.0086
	(-3.44)	(-0.06)	(-3.04)	(1.20)	(-0.80)	(-0.84)	(-0.95)	(-1.08)
Lnedu	0.0045***	0.0014	0.0046***	0.0015	0.0006	-0.0041***	0.0005	-0.0228***
	(3.02)	(0.58)	(3.02)	(0.10)	(0.92)	(-3.35)	(0.69)	(-4.61)
Lnroad	-0.002	-0.0022	-0.0025	-0.0374*	-0.0003	-0.0005	-0.0003	0.0019
	(-1.16)	(-0.56)	(-1.40)	(-1.67)	(-0.96)	(-1.02)	(-1.03)	(1.52)
Lnopen	-0.0002	-0.0011	-0.0002	0.0016	-0.0001	0	0	-0.0037**
	(-0.29)	(-0.93)	(-0.34)	(0.22)	(-0.46)	(-0.08)	(-0.16)	(-2.20)
Lnpgdp	0.0046	0.0322	0.0096	0.2016	-0.0034	0.0004	-0.0074	0.1211**
	(0.17)	(0.60)	(0.35)	(0.91)	(-0.29)	(0.02)	(-0.66)	(2.17)
Lnind	0.0185**	0.0022	0.0228***	-0.0335	-0.007	0.0125	-0.0086*	0.0196
	(2.15)	(0.13)	(2.63)	(-0.41)	(-1.41)	(1.38)	(-1.76)	(0.64)
Lngov	-0.0009	-0.066	0.0032	-0.458	-0.0154**	-0.0011	-0.0161***	-0.0085
	(-0.04)	(-1.31)	(0.13)	(-1.47)	(-2.44)	(-0.09)	(-2.66)	(-0.19)
*N*	972	972	972	972	864	864	864	864
*R* ^2^	0.0341	0.0341	0.2548	0.2548	0.054	0.054	0.0017	0.0017

#### 5.3.2 Spatial decomposition effect of different regions

The 108 cities along the Yangtze River Economic Belt span the east and central parts of China, covering more than 1/3 of prefecture-level cities. The differences between cities are mainly reflected in geographical location and economic level. Therefore, it is also necessary to investigate urban innovation and its spillover effect in different regions. Table 9 shows the decomposition results of the spatial Durbin model in the middle, upper and lower reaches of the Yangtze River. Since the importance of the divided samples is less than that of the whole samples due to the influence of distance, only the results based on the adjacency matrix are displayed. The direct effect of urban innovation in the middle and upper reaches is significantly positive, while the indirect and indirect effect is significantly negative, indicating that the improvement of the innovation level of the middle and upper reaches cities will significantly improve the total factor productivity of the manufacturing industry in the local region while having a negative spillover effect on the total factor productivity of the manufacturing industry in neighboring cities because the development level of the middle and upper reaches cities is quite different. The development of most small and medium-sized cities is more influenced by surrounding big cities such as Wuhan, Changsha, Chengdu and Chongqing. In the stage of low development level, big cities in the region enjoy more advantages than small and medium-sized cities in terms of talent policy, technical level and capital conditions [34], so these resources are concentrated in big cities. And this spillover effect is mostly a negative siphon effect for most small cities. The direct and indirect effects of urban innovation in the downstream area are positive, but not significant. The possible reason is that most of the downstream cities have strong strength, and after years of development of innovation level, their marginal contribution to the total factor productivity is gradually declining, and more direct input factors, such as high-end talent, digital technology, intelligent equipment and so on, that affect the growth of manufacturing total factor productivity are increasing. The effect of innovation on the total factor productivity of the manufacturing industry is not obvious [35].

**Table 9:** Decomposition of spatial Dubin model by region.

variable	upstream		midstream		downstream	
	(1) Direct effect	(2) Indirect effect	(3) Direct effect	(4) Indirect effect	(5) Direct effect	(6) Indirect effect
Lninnov	0.0050***	-0.0100***	0.0060***	-0.0044*	0.0018	0.005
	(3.00)	(-4.20)	(3.96)	(-1.70)	(1.10)	(1.53)
Lnptech	-0.0023**	-0.0014	-0.0028***	-0.0036**	-0.0060***	0.0029
	(-2.14)	(-0.78)	(-3.15)	(-2.29)	(-4.82)	(1.10)
Lnpopu	-0.0064	0.0418***	0.0216**	0.0088	-0.0061*	0.0072
	(-0.94)	(3.49)	(2.36)	(0.39)	(-1.77)	(1.17)
Lnedu	0.0005	0.0023	0.0005	-0.0098***	0.0177***	0.0044
	(0.52)	(1.42)	(0.34)	(-3.56)	(6.54)	(0.78)
Lnroad	-0.0002	0.0008	0.0019	0.0055*	-0.0032	0.0038
	(-0.23)	(0.76)	(1.21)	(1.85)	(-1.37)	(0.60)
Lnopen	-0.0002	0.0014**	0.0018**	0.0046***	-0.0023**	0.0002
	(-0.42)	(2.57)	(2.20)	(3.59)	(-2.40)	(0.10)
Lnpgdp	0.0475	-0.0131	-0.0012	0.0058	-0.1441***	-0.0314**
	(1.50)	(-0.32)	(-0.04)	(0.11)	(-6.51)	(-2.07)
Lnind	0.0029	0.0286**	-0.0166	0.018	-0.0005	-0.0153
	(0.36)	(2.36)	(-1.57)	(1.14)	(-0.05)	(-1.07)
Lngov	-0.0761***	-0.0112	0.0622***	0.0793**	-0.0169	-0.0254
	(-5.08)	(-0.50)	(2.93)	(2.50)	(-0.76)	(-0.60)
*N*	527	527	612	612	697	697
*R* ^2^	0.0058	0.0058	0.0005	0.0005	0.1398	0.1398

### 5.4 Adjustment effect

To verify the moderating effect of talent agglomeration, the interaction term between urban innovation and talent agglomeration is introduced into the model. The results are shown in Table 10. The coefficient of urban innovation in the first row is significantly positive, which is consistent with the basic conclusion of this paper. After adding the moderating term of talent agglomeration, the interaction term between urban innovation and talent agglomeration in the fixed effect model in column (5) is significantly positive, indicating that talent agglomeration significantly promotes the positive effect of urban innovation on the regional manufacturing total factor productivity when there is no spatial effect. Taking spatial factors into consideration, the adjustment effect of talent agglomeration is different: In columns (1) and (3), under the two weight matrices, talent agglomeration significantly moderates the positive impact of urban innovation on the total factor productivity of the manufacturing industry in this region, and the coefficients are not different, which verifies hypothesis H_3_, while the indirect effect in column (2) is positive and insignificant, and the indirect effect in column (4) is negative and insignificant. It shows that the improvement of talent concentration mainly affects the inner city of the local region, and it is difficult to have an impact on the surrounding area through the spillover effect at the current stage, and the direction of the effect is uncertain. The reason may be restricted by the movements of talents and the household registration system. Although traffic and information convenience enhance the spatial interaction between cities, it is still difficult to replace face-to-face communication and learning under some conditions. Therefore, it is difficult to have a significant effect on the surrounding cities through the spillover effect. Due to the differences in the degree of talent agglomeration, urban development, talent policies, and other factors, the spillover effect may be the siphoning effect or diffusion effect, so the direction of the spillover effect of talent agglomeration is not clear at the present stage.

**Table 10:** Moderating effect of talent agglomeration.

variable	Adjacency matrix		Inverse distance matrix		Fe
	(1) Direct effect	(2) Indirect effect	(3) Direct effect	(4) Indirect effect	
Lninnov	0.0035***	0.0082***	0.0044***	0.0273***	0.0029***
	(3.46)	(4.90)	(4.31)	(2.95)	(7.91)
Lninnov×Lntalent	0.0032***	0.0005	0.0031***	-0.002	0.0012***
	(4.13)	(0.28)	(3.88)	(-0.20)	(3.62)
Lnptech	-0.0031***	-0.001	-0.0031***	-0.0043	-0.0007***
	(-5.49)	(-0.94)	(-5.43)	(-0.64)	(-3.33)
Lnpopu	-0.0034	0.0104**	-0.002	0.0455	0.0007
	(-1.54)	(2.54)	(-0.94)	(1.57)	(0.82)
Lnedu	0.0029***	0.001	0.0028***	-0.0068	-0.0001
	(3.41)	(0.63)	(3.27)	(-0.70)	(-0.17)
Lnroad	-0.0010*	-0.0017*	-0.0011*	-0.006	-0.0002
	(-1.72)	(-1.65)	(-1.91)	(-1.23)	(-0.70)
Lnopen	-0.0003	-0.001	-0.0002	-0.0085**	-0.0002
	(-0.78)	(-1.64)	(-0.47)	(-2.07)	(-1.20)
Lnpgdp	-0.0730***	0.0056	-0.0827***	0.0399	-0.0444***
	(-6.26)	(0.22)	(-7.13)	(0.31)	(-50.31)
Lnind	0.0002	0.0190**	-0.0017	0.0126	-0.0187***
	(0.04)	(2.10)	(-0.37)	(0.27)	(-15.18)
Lngov	-0.0128	0.0193	-0.0106	0.0292	-0.0213***
	(-1.15)	(0.80)	(-0.96)	(0.19)	(-4.85)

## 6. Conclusions

Taking 108 cities in the Yangtze River Economic Belt as samples, this paper studies the impacts of urban innovation on manufacturing total factor productivity and its spatial spillover effect during 2004–2020. The main conclusions are as follows: (1) There is an obvious spatial correlation and heterogeneity between urban innovation and manufacturing total factor productivity among cities in the Yangtze River Economic Belt. (2) Urban innovation not only has a significant positive impact on the local manufacturing total factor productivity but also has an impact on neighboring cities through spatial spillover. By replacing the spatial weight matrix, changing the measurement index of the core explanatory variable and changing the estimation method, the conclusions remain robust. (3) In terms of structure stability, we find in the period from 2004 to 2012, urban innovation has a significant positive impact on manufacturing total factor productivity, and the spillover effect is greater than the direct effect, and from 2013 to 2020, things are different. The direct effect of urban innovation on the total factor productivity of the manufacturing industry is negative, while the indirect effect is positive and insignificant. By region, the direct effect of urban innovation in the middle and upper reaches on the total factor productivity of the manufacturing industry is significantly positive, while the spillover effect is significantly negative. The direct and spillover effects of downstream urban innovation on manufacturing total factor productivity are positive but not significant. (4) By constructing the interaction term between urban innovation and talent agglomeration, we find talent agglomeration significantly promotes the positive impact of urban innovation on the regional manufacturing total factor productivity, but the spillover effect is not significant.

The conclusions have specific implications. First, the widespread spillover effect between cities makes it necessary to establish an open system of innovation cooperation between cities and remove regional barriers that hinder the free movement of urban innovation factors. and second, differentiated urban innovation strategies should be adopted. Middle and upstream cities should endeavor to attract more innovation factors and enhance innovation cooperation, to make innovation collaboration more feasible. for downstream cities, the innovation factors should also be spatially optimally distributed for higher practical value. In the end, as an important intermediary innovation factor, different cities are expected to strengthen the share of talent, to exert the value of intelligence resources to a greater extent.

## Supporting information

S1 Table Correlation description of variables. (DOCX)

S2 Table Decomposition of spatial Dubin model under the weight matrix of economic distance space. (DOCX)

S3 Table Decomposition of spatial Dubin model with replacement of core explanatory variables. (DOCX)

S4 Table Decomposition of spatial Dubin model with explained variables lagging one stage. (DOCX)

S1 Data (DOCX)

## References

[1] Anselin L. Spatial Econometrics: Methods and Models: Springer Dordrecht; 1988. Springer Dordrecht p.

[2] Yonghong X, Liang S, Chuanwang S. Re-estimating the Total Factor Productivity in China—Improvement and Examples of Elasticity Estimation in ACF Model. Statistical Research. 2020;37(01):33–46. doi: 10.19343/j.cnki.11-1302/c.2020.01.003

[3] Duguet E. Innovation height, spillovers and tfp growth at the firm level: Evidence from French manufacturing. Economics of Innovation and New Technology. 2006;15(4–5):415–42. doi: 10.1080/10438590500512968

[4] Crepon B, Duguet E, Mairessec J. Research, Innovation And Productivi[Ty: An Econometric Analysis At The Firm Level. Economics of Innovation and New Technology. 1998;7(2):115–58. doi: 10.1080/10438599800000031

[5] Bernstein JI, Nadiri MI. Product Demand, Cost Of Production, Spillovers And The Social Rate Or Return To R&D. Working Papers. 1990.

[6] Henderson R, Jaffe AB, Trajtenberg M. Universities as a Source of Commercial Technology: A Detailed Analysis of University Patenting, 1965–1988. The Review of Economics and Statistics. 1998;80(1):119–27. doi: 10.1162/003465398557221

[7] Griffith R, Redding S, Reenen JV. Mapping the Two Faces of R& D: Productivity Growth in a Panel of OECD Industries. The Review of Economics and Statistics. 2004;86(4):883–95. doi: 10.1162/0034653043125194

[8] Acemoglu D, Antràs P, Helpman E. Contracts and Technology Adoption. American Economic Review. 2007;97(3):916–43. doi: 10.1257/aer.97.3.916

[9] Borensztein E, De Gregorio J, Lee JW. How does foreign direct investment affect economic growth? Journal of International Economics. 1998;45(1):115–35. 10.1016/S0022-1996(97)00033-0.

[10] Glaeser ELHD. Growth in Cities Journal of Political Economy. 1992;100(6):1126–52.

[11] Kijek T, Matras-Bolibok A. The relationship between TFP and innovation performance: evidence from EU regions. Equilibrium. 2019;14(4):695–709. doi: 10.24136/eq.2019.032

[12] Keller W. Geographic Localization of International Technology Diffusion. The American Economic Review. 2002;92(1):120–42.

[13] Anselin L, Varga A, Acs Z. Local Geographic Spillovers between University Research and High Technology Innovations. Journal of Urban Economics. 1997;3(42):422–48.

[14] Yi Ming PJ. Research on the Evolution Law and Spatial Difference of Patent Innovation Efficiency in Chinese High-Tech Industy——Estimation and Decomposition based on Total Factor Productivity Science & Technology Progress and Policy. 2018;35(05):68–73.

[15] Zhang Yan SQ, Chen Ting. The Analysis of International R&D Spillover and Productivity Growth Path of Manufacturing Based on Panel Data. Science & Technology Progress and Policy. 2010;27(03):40–4.

[16] Yufen JCC. R&D resource allocation, spatial correlation and regional TFP growth. Studies in Science of Science. 2018;36(01):81–92. doi: 10.16192/j.cnki.1003-2053.2018.01.010

[17] Nanyun G, dian H. Corporate Innovation Behavior, Institutional Enviroment and Industrial Total Factor Productivity Improvement Journal of Capital University of Economics and Business. 2021;23(06):43–58. doi: 10.13504/j.cnki.issn1008-2700.2021.06.004

[18] Lun X. Spatial Effect of Industrial Integration on Total Factor Productivity: From the Perspective of the Integration of Advanced Manufacturing Industry and Modern Service Industry. Journal of Beijing University of Aeronautics and Astronautics(Social Sciences Edition). 2022:1–12. doi: 10.13766/j.bhsk.1008-2204.2021.0692

[19] Junqing L, Yu G, Xiang L. Environmental egulation and Dynamic Productivity Changes in China: A Study from the Perspective of Heterogeneous Firms. The Journal of World Economy. 2022;45(01):82–109. doi: 10.19985/j.cnki.cassjwe.2022.01.009

[20] Cabrer-Borrás B, Serrano-Domingo G. Innovation and R&D spillover effects in Spanish regions: A spatial approach. Research Policy. 2007;36(9):1357–71. doi: 10.1016/j.respol.2007.04.012

[21] Yongze Y. Spatial and Temporal Characteristics of the Propulsion Transform of China’s Economic Growth since the Reform and Opening up. Journal of Quantitative & Technological Economics. 2015;32(02):19–34. doi: 10.13653/j.cnki.jqte.2015.02.002

[22] Wenjun Z, Jinping Y. Marketization and Economic Growth Pattern in China: Empirical Analysis Based on Provincial Panel Data. Nankai Economic Studies. 2014; No.177(03):3–22. doi: 10.14116/j.nkes.2014.03.005

[23] Simon CJ, Nardinelli C. Human capital and the rise of American cities, 1900–1990. Regional science and urban economics. 2002;32(1):59–96.

[24] Ye L, Jingyuan Z, Ruoyu W, Peiyu Z, Zhuolin P. The Relationship between Geographical Concentration of Researchers and Regional Innovation in China. Economic Geography. 2019;39(07):139–47. doi: 10.15957/j.cnki.jjdl.2019.07.016

[25] Jun Z, Guiying W, Jipeng Z. The Estimation of China’ s provincial capital stock:1952–2000. Economic Research Journal. 2004;(10):35–44.

[26] Crescenzi R, Rodríguez-Pose A, Storper M. The territorial dynamics of innovation in China and India. Journal of Economic Geography. 2012;12:1055–85. doi: 10.1093/jeg/lbs020

[27] Acs Z, Anselin L, Varga A. Patents and Innovation Counts as Measures of Regional Production of New Knowledge. Research Policy. 2002;31:1069–85. doi: 10.1016/S0048-7333(01)00184-6

[28] Lina Z, Zhiliang N. Scientific and Technological Talent Agglomeration and Region Innovation Efficiency——Empirical Test Based on Spatial Spillover and Threshold Effect. Soft Science. 2022;36(09):45–50. doi: 10.13956/j.ss.1001-8409.2022.09.07

[29] Jianguo L, Guoping L, Juntao Z, Tieshan S. Spatial Distribution and Its Affecting Factors of Economy Efficiency and Total Factor Productivity in China: 1990–2009. Acta Geographica Sinica. 2012;67(08):1069–84.

[30] Ping L, Xueyi Z, Hongwei w, Shilin Z. Productivity Change and the Source of Economic Growth in China. Journal of Quantitative & Technological Economics. 2013;30(01):3–21. doi: 10.13653/j.cnki.jqte.2013.01.011

[31] Yuanheng L, Lingyan S, Yu L. Measurement and Comparison of Total Factor Productivity Growth in China’s Manufacturing Industry. On Economic Problems. 2020; No.487(03):83–91. doi: 10.16011/j.cnki.jjwt.2020.03.011

[32] LeSage JP, Pace RK. Spatial Econometric Modeling of Origin-Destination Flows*. Journal of Regional Science. 2008;48(5):941–67. doi: 10.1111/j.1467-9787.2008.00573.x

[33] Jing M, Hongbing D, Aixin C. Analysis on City Innovation Output of Space-Time Distribution Pattern an Influential Factors in China: Empirical Analysis of 285 Cities in China. Science of Science and Management of S& T. 2017;38(10):12–25.

[34] Mengyu S, Kunrong S. Research on Economic Growth and Spatial Spillover Effect of Talent Introduction Policy Tools———A Case Study of Yangtze River Delta. Inquiry into Economic Issues. 2022; No.474(01):32–49.

[35] Maomao P, Yulin Z. Internet convergence, labor structure,and total factor productivity in manufacturing. Studies in Science of Science. 2020;38(12):2171–82+219. doi: 10.16192/j.cnki.1003-2053.2020.12.007

