# Supplementary material for: Spatial spillover effects of urban innovation on productivity growth: A case study of 108 cities in the Yangtze River Economic Belt
Source: PLoS One. 2023 Dec 21;18(12):e0294997. doi: 10.1371/journal.pone.0294997 (PMC10734961; doi:10.1371/journal.pone.0294997)
Supplement: S1 Table — (DOCX) [file pone.0294997.s001.docx]

**Supporting information-S1**

**S1 Table. Correlation description of variables**

|  | Lntfp | Lninnov | Lnptech | Lnpopu | Lnedu | Lnroad | Lnopen | Lnpgdp | Lnind | Lngov |
| --- | --- | --- | --- | --- | --- | --- | --- | --- | --- | --- |
| Lntfp | 1 | -0.407*** | -0.497*** | -0.043* | -0.098*** | -0.309*** | -0.137*** | -0.323*** | 0.030 | -0.397*** |
| Lninnov | -0.356*** | 1 | 0.900*** | 0.071*** | 0.620*** | 0.549*** | 0.656*** | 0.912*** | 0.346*** | 0.599*** |
| Lnptech | -0.559*** | 0.847*** | 1 | 0.009 | 0.509*** | 0.540*** | 0.582*** | 0.844*** | 0.327*** | 0.698*** |
| Lnpopu | -0.043* | 0.087*** | -0.003 | 1 | 0.466*** | -0.019 | 0.362*** | 0.009 | -0.180*** | -0.015 |
| Lnedu | -0.081*** | 0.576*** | 0.497*** | 0.459*** | 1 | 0.166*** | 0.643*** | 0.619*** | 0.171*** | 0.309*** |
| Lnroad | -0.270*** | 0.388*** | 0.397*** | -0.050** | 0.124*** | 1 | 0.422*** | 0.464*** | 0.255*** | 0.350*** |
| Lnopen | -0.147*** | 0.626*** | 0.549*** | 0.358*** | 0.601*** | 0.309*** | 1 | 0.632*** | 0.219*** | 0.447*** |
| Lnpgdp | -0.326*** | 0.892*** | 0.833*** | 0.029 | 0.623*** | 0.321*** | 0.634*** | 1 | 0.461*** | 0.494*** |
| Lnind | 0.016 | 0.322*** | 0.297*** | -0.206*** | 0.098*** | 0.167*** | 0.162*** | 0.445*** | 1 | 0.154*** |
| Lngov | -0.382*** | 0.514*** | 0.650*** | -0.036 | 0.337*** | 0.209*** | 0.380*** | 0.448*** | 0.122*** | 1 |
