# Supplementary material for: Spatial spillover effects of urban innovation on productivity growth: A case study of 108 cities in the Yangtze River Economic Belt
Source: PLoS One. 2023 Dec 21;18(12):e0294997. doi: 10.1371/journal.pone.0294997 (PMC10734961; doi:10.1371/journal.pone.0294997)
Supplement: S2 Table — (DOCX) [file pone.0294997.s002.docx]

**Supporting information-S2**

**S2 Table. Decomposition of spatial Dubin model under the weight matrix of economic distance space**

| **variable** | **(1) Direct effect** | **(2) Indirect effect** | **(3)Total effect** | **(4) Direct effect** | **(5) Indirect effect** | **(6)Total effect** |
| --- | --- | --- | --- | --- | --- | --- |
| Lninnov | 0.0042^***^ | 0.0015^***^ | 0.0057^***^ | 0.0058^***^ | 0.0026^***^ | 0.0085^***^ |
|  | (5.34) | (3.47) | (6.82) | (6.80) | (3.69) | (8.29) |
| Lnptech |  |  |  | -0.0030^***^ | -0.0003 | -0.0033^***^ |
|  |  |  |  | (-5.25) | (-0.62) | (-4.42) |
| Lnpopu |  |  |  | -0.0004 | -0.0005 | -0.001 |
|  |  |  |  | (-0.19) | (-0.27) | (-0.36) |
| Lnedu |  |  |  | 0.0021^**^ | 0.0001 | 0.0023^*^ |
|  |  |  |  | (2.57) | (0.12) | (1.65) |
| Lnroad |  |  |  | -0.0007 | -0.0008 | -0.0016^**^ |
|  |  |  |  | (-1.32) | (-1.31) | (-2.10) |
| Lnopen |  |  |  | -0.0004 | -0.0003 | -0.0006 |
|  |  |  |  | (-1.06) | (-0.80) | (-1.16) |
| Lnpgdp |  |  |  | -0.0659^***^ | -0.0161^***^ | -0.0820^***^ |
|  |  |  |  | (-5.47) | (-5.24) | (-5.74) |
| Lnind |  |  |  | 0.0068 | -0.0013 | 0.0055 |
|  |  |  |  | (1.50) | (-0.41) | (0.95) |
| Lngov |  |  |  | -0.0207^*^ | 0.0465^***^ | 0.0259^*^ |
|  |  |  |  | (-1.89) | (4.69) | (1.74) |
