# Supplementary material for: Spatial spillover effects of urban innovation on productivity growth: A case study of 108 cities in the Yangtze River Economic Belt
Source: PLoS One. 2023 Dec 21;18(12):e0294997. doi: 10.1371/journal.pone.0294997 (PMC10734961; doi:10.1371/journal.pone.0294997)
Supplement: S3 Table — (DOCX) [file pone.0294997.s003.docx]

**Supporting information-S3**

**S3 Table. Decomposition of spatial Dubin model with replacement of core explanatory variables**

| **variable** | **Adjacency matrix** | | | **Inverse distance matrix** | | |
| --- | --- | --- | --- | --- | --- | --- |
|  | **(1) Direct effect** | **(2) Indirect effect** | **(3)Total effect** | **(4) Direct effect** | **(5) Indirect effect** | **(6)Total effect** |
| LnInnova | 0.0051^***^ | 0.0093^***^ | 0.0144^***^ | 0.0057^***^ | 0.0274^***^ | 0.0331^***^ |
|  | (5.47) | (5.39) | (7.87) | (6.10) | (2.87) | (3.50) |
| Lnptech | -0.0032^***^ | -0.0012 | -0.0044^***^ | -0.0034^***^ | -0.0038 | -0.0072 |
|  | (-5.37) | (-1.03) | (-3.56) | (-5.52) | (-0.53) | (-1.01) |
| Lnpopu | -0.0018 | 0.0121^***^ | 0.0103^**^ | -0.0008 | 0.0299 | 0.0291 |
|  | (-0.84) | (2.87) | (2.34) | (-0.36) | (1.08) | (1.05) |
| Lnedu | 0.0028^***^ | 0.0014 | 0.0042^**^ | 0.0027^***^ | -0.0058 | -0.0031 |
|  | (3.24) | (0.90) | (2.24) | (3.08) | (-0.57) | (-0.30) |
| Lnroad | -0.0011^*^ | -0.0017^*^ | -0.0028^***^ | -0.0010^*^ | -0.0062 | -0.0073 |
|  | (-1.92) | (-1.69) | (-2.59) | (-1.89) | (-1.32) | (-1.55) |
| Lnopen | -0.0003 | -0.0015^**^ | -0.0018^***^ | -0.0001 | -0.0104^***^ | -0.0105^***^ |
|  | (-0.80) | (-2.51) | (-2.60) | (-0.38) | (-2.63) | (-2.64) |
| Lnpgdp | -0.0738^***^ | 0.0062 | -0.0675^***^ | -0.0828^***^ | 0.0673 | -0.0155 |
|  | (-5.86) | (0.24) | (-2.58) | (-6.59) | (0.54) | (-0.13) |
| Lnind | -0.0009 | 0.0202^**^ | 0.0193^**^ | -0.0026 | 0.0256 | 0.0231 |
|  | (-0.19) | (2.19) | (1.96) | (-0.54) | (0.58) | (0.52) |
| Lngov | -0.0146 | 0.016 | 0.0014 | -0.0121 | 0.0607 | 0.0486 |
|  | (-1.34) | (0.68) | (0.06) | (-1.11) | (0.44) | (0.35) |
