# Supplementary material for: Spatial spillover effects of urban innovation on productivity growth: A case study of 108 cities in the Yangtze River Economic Belt
Source: PLoS One. 2023 Dec 21;18(12):e0294997. doi: 10.1371/journal.pone.0294997 (PMC10734961; doi:10.1371/journal.pone.0294997)
Supplement: S4 Table — (DOCX) [file pone.0294997.s004.docx]

**Supporting information-S4**

**S4 Table. Decomposition of spatial Dubin model with explained variables lagging one stage**

| **variable** | **Adjacency matrix** | | | **Inverse distance matrix** | | |
| --- | --- | --- | --- | --- | --- | --- |
|  | **(1) Direct effect** | **(2) Indirect effect** | **(3)Total effect** | **(4) Direct effect** | **(5) Indirect effect** | **(6)Total effect** |
| LnInnova | 0.0043^***^ | 0.0080^***^ | 0.0123^***^ | 0.0048^***^ | 0.0290^**^ | 0.0338^***^ |
|  | (4.35) | (4.13) | (5.96) | (4.77) | (2.37) | (2.78) |
| Lnptech | -0.0030^***^ | -0.0012 | -0.0042^***^ | -0.0029^***^ | -0.0099 | -0.0128 |
|  | (-4.55) | (-0.94) | (-2.99) | (-4.48) | (-1.12) | (-1.45) |
| Lnpopu | -0.0031 | 0.0153^***^ | 0.0122^***^ | 0 | 0.05 | 0.05 |
|  | (-1.39) | (3.43) | (2.61) | (-0.71) | (1.58) | (1.53) |
| Lnedu | 0.0025^***^ | 0.001 | 0.0035^*^ | 0.0025^***^ | -0.0012 | 0.0013 |
|  | (2.70) | (0.61) | (1.75) | (2.59) | (-0.09) | (0.10) |
| Lnroad | -0.0012^**^ | -0.0017 | -0.0029^**^ | -0.0013^**^ | -0.01 | -0.01 |
|  | (-2.07) | (-1.54) | (-2.48) | (-2.19) | (-1.24) | (-1.48) |
| Lnopen | -0.0007^*^ | -0.0014^**^ | -0.0022^***^ | -0.0005 | -0.0092^*^ | -0.0097^*^ |
|  | (-1.90) | (-2.21) | (-2.88) | (-1.33) | (-1.84) | (-1.92) |
| Lnpgdp | -0.0884^***^ | -0.0066 | -0.0950^***^ | -0.0993^***^ | -0.0069 | -0.1062 |
|  | (-6.64) | (-0.23) | (-3.33) | (-7.47) | (-0.05) | (-0.70) |
| Lnind | -0.0051 | 0.0293^***^ | 0.0242^**^ | -0.008 | 0.1166^**^ | 0.1086^*^ |
|  | (-0.99) | (2.80) | (2.14) | (-1.52) | (2.05) | (1.89) |
| Lngov | -0.0078 | 0.0213 | 0.0135 | -0.0061 | 0.0408 | 0.0347 |
|  | (-0.68) | (0.84) | (0.49) | (-0.53) | (0.25) | (0.21) |
