# Supplementary material for: Spatial spillover effects of urban innovation on productivity growth: A case study of 108 cities in the Yangtze River Economic Belt
Source: PLoS One. 2023 Dec 21;18(12):e0294997. doi: 10.1371/journal.pone.0294997 (PMC10734961; doi:10.1371/journal.pone.0294997)
Supplement: S1 Data — (DOCX) [file pone.0294997.s005.docx]

| Year | City Code | Lntfp | Lninnov | Lntalent | Lnptech | Lnpopu | Lnedu | Lnroad | Lnopen | Lnpgdp | Lnind | Lngov |
| --- | --- | --- | --- | --- | --- | --- | --- | --- | --- | --- | --- | --- |
| 2004 | 310000 | 0.796 | 2.09 | 0.782 | 13.81 | 7.21 | 12.94 | 2.732 | 15.5 | 10.87 | 1.108 | 0.147 |
| 2004 | 320100 | 0.788 | 1.352 | 1.221 | 12.32 | 6.369 | 13.11 | 2.566 | 14.57 | 10.34 | 1.223 | 0.09 |
| 2004 | 320200 | 0.768 | 1.352 | 0.492 | 10.95 | 6.103 | 11.02 | 3.02 | 14.37 | 10.79 | 1.555 | 0.06 |
| 2004 | 320300 | 0.797 | 0.524 | 0.407 | 9.828 | 6.821 | 11.37 | 2.435 | 12.52 | 9.326 | 1.441 | 0.04 |
| 2004 | 320400 | 0.779 | 1.474 | 0.631 | 10.85 | 5.855 | 11.21 | 2.855 | 13.08 | 10.29 | 1.679 | 0.063 |
| 2004 | 320500 | 0.769 | 1.69 | 0.613 | 11.17 | 6.395 | 11.46 | 2.727 | 15.24 | 10.89 | 2.003 | 0.066 |
| 2004 | 320600 | 0.778 | 0.583 | 0.348 | 10.33 | 6.651 | 10.85 | 2.303 | 13.73 | 9.588 | 1.599 | 0.045 |
| 2004 | 320700 | 0.787 | 0.343 | 0.27 | 10.18 | 6.15 | 10.13 | 2.575 | 12.23 | 9.044 | 1.367 | 0.046 |
| 2004 | 320800 | 0.798 | 0.332 | 0.336 | 9.157 | 6.262 | 10.54 | 2.318 | 11.51 | 9.118 | 1.656 | 0.045 |
| 2004 | 320900 | 0.783 | 0.355 | 0.215 | 9.366 | 6.682 | 10.23 | 2.137 | 11.75 | 9.258 | 1.493 | 0.035 |
| 2004 | 321000 | 0.806 | 0.907 | 0.529 | 10.17 | 6.119 | 10.88 | 2.499 | 13.42 | 9.684 | 1.472 | 0.05 |
| 2004 | 321100 | 0.776 | 1.087 | 0.675 | 11.06 | 5.588 | 10.84 | 2.436 | 13.13 | 10.2 | 1.632 | 0.048 |
| 2004 | 321200 | 0.788 | 0.623 | 0.202 | 10.41 | 6.22 | 9.523 | 2.868 | 12.75 | 9.468 | 1.563 | 0.05 |
| 2004 | 321300 | 0.803 | 0.111 | 0.067 | 8.124 | 6.257 | 9.151 | 3.114 | 9.48 | 8.715 | 1.526 | 0.034 |
| 2004 | 330100 | 0.779 | 1.595 | 1.074 | 11.74 | 6.48 | 12.59 | 2.471 | 13.97 | 10.52 | 1.317 | 0.08 |
| 2004 | 330200 | 0.75 | 1.869 | 0.639 | 11.65 | 6.315 | 11.48 | 2.563 | 14.37 | 10.51 | 1.587 | 0.072 |
| 2004 | 330300 | 0.69 | 1.546 | 0.331 | 11.19 | 6.615 | 10.72 | 2.202 | 12.06 | 9.832 | 1.601 | 0.06 |
| 2004 | 330400 | 0.646 | 0.869 | 0.243 | 11.91 | 5.811 | 9.779 | 3.063 | 13.65 | 10.27 | 1.931 | 0.048 |
| 2004 | 330500 | 0.691 | 0.872 | 0.288 | 10.67 | 5.55 | 9.566 | 3.053 | 13.13 | 9.986 | 1.551 | 0.052 |
| 2004 | 330600 | 0.71 | 1.105 | 0.315 | 11.75 | 6.075 | 10.13 | 2.547 | 13.43 | 10.26 | 1.837 | 0.041 |
| 2004 | 330700 | 0.732 | 1.589 | 0.451 | 11.37 | 6.113 | 10.63 | 2.946 | 12.78 | 9.917 | 1.547 | 0.055 |
| 2004 | 330800 | 0.737 | 0.294 | 0.214 | 10.63 | 5.504 | 9.01 | 2.894 | 9.754 | 9.294 | 1.366 | 0.063 |
| 2004 | 330900 | 0.75 | 0.402 | 0.543 | 11.85 | 4.574 | 9.235 | 2.652 | 9.833 | 9.909 | 1.077 | 0.067 |
| 2004 | 331000 | 0.75 | 1.331 | 0.201 | 11.63 | 6.321 | 9.64 | 2.523 | 12.43 | 9.917 | 1.904 | 0.049 |
| 2004 | 331100 | 0.772 | 0.397 | 0.282 | 11.58 | 5.524 | 9.656 | 2.465 | 9.74 | 9.203 | 1.239 | 0.063 |
| 2004 | 340100 | 0.788 | 0.798 | 1.265 | 10.27 | 6.097 | 12.23 | 2.76 | 12.47 | 9.408 | 1.246 | 0.079 |
| 2004 | 340200 | 0.847 | 0.493 | 0.995 | 9.26 | 5.414 | 11.32 | 2.209 | 12.1 | 9.6 | 1.698 | 0.063 |
| 2004 | 340300 | 0.773 | 0.288 | 0.546 | 9.47 | 5.849 | 10.49 | 2.139 | 11.33 | 8.887 | 1.206 | 0.048 |
| 2004 | 340400 | 0.755 | 0.14 | 0.747 | 8.401 | 5.454 | 10.61 | 1.996 | 9.652 | 9.141 | 1.353 | 0.059 |
| 2004 | 340500 | 0.816 | 0.549 | 0.694 | 10.22 | 4.823 | 9.999 | 2.624 | 10.69 | 9.778 | 2.495 | 0.081 |
| 2004 | 340600 | 0.771 | 0.155 | 0.479 | 9.415 | 5.344 | 9.879 | 2.17 | 10.28 | 8.914 | 1.612 | 0.058 |
| 2004 | 340700 | 0.767 | 0.479 | 0.784 | 11.11 | 4.272 | 9.342 | 2.198 | 10.68 | 9.709 | 1.689 | 0.061 |
| 2004 | 340800 | 0.773 | 0.111 | 0.173 | 9.346 | 6.404 | 9.41 | 2.442 | 9.516 | 8.704 | 1.46 | 0.049 |
| 2004 | 341000 | 0.789 | 0.072 | 0.227 | 10.11 | 4.986 | 8.965 | 1.73 | 10.46 | 9.027 | 0.769 | 0.054 |
| 2004 | 341100 | 0.768 | 0.146 | 0.277 | 9.869 | 6.072 | 9.955 | 2.537 | 10.56 | 8.992 | 1.252 | 0.031 |
| 2004 | 341200 | 0.771 | 0.088 | 0.096 | 9.118 | 6.823 | 9.793 | 2.362 | 10.04 | 7.988 | 0.825 | 0.048 |
| 2004 | 341300 | 0.775 | 0.075 | 0.114 | 8.847 | 6.396 | 9.618 | 2.44 | 8.9 | 8.351 | 0.691 | 0.032 |
| 2004 | 341500 | 0.769 | 0.065 | 0.153 | 9.452 | 6.515 | 9.842 | 2.578 | 9.599 | 8.176 | 1.03 | 0.046 |
| 2004 | 341600 | 0.772 | 0.114 | 0.067 | 8.338 | 6.302 | 8.137 | 2.765 | 8.817 | 8.3 | 0.76 | 0.032 |
| 2004 | 341700 | 0.783 | 0.092 | 0.392 | 8.534 | 5.046 | 8.937 | 2.212 | 10.13 | 8.62 | 1.12 | 0.054 |
| 2004 | 341800 | 0.776 | 0.187 | 0.041 | 9.397 | 5.61 | 7.164 | 2.412 | 10.31 | 9.058 | 1.112 | 0.039 |
| 2004 | 360100 | 0.759 | 0.682 | 1.3 | 10.46 | 6.133 | 12.58 | 2.01 | 13.31 | 9.694 | 1.327 | 0.057 |
| 2004 | 360200 | 0.741 | 0.171 | 0.454 | 10.25 | 5.014 | 9.578 | 2.088 | 9.37 | 9.267 | 1.565 | 0.047 |
| 2004 | 360300 | 0.748 | 0.241 | 0.139 | 10.45 | 5.194 | 8.416 | 2.426 | 10.66 | 9.127 | 2.111 | 0.057 |
| 2004 | 360400 | 0.777 | 0.121 | 0.518 | 10.03 | 6.142 | 10.94 | 1.908 | 12.45 | 8.929 | 1.477 | 0.053 |
| 2004 | 360500 | 0.768 | 0.289 | 0.287 | 10.49 | 4.704 | 8.708 | 2.524 | 10.66 | 9.323 | 1.668 | 0.059 |
| 2004 | 360600 | 0.763 | 0.364 | 0.164 | 9.721 | 4.715 | 7.899 | 2.166 | 10.3 | 9.05 | 1.385 | 0.057 |
| 2004 | 360700 | 0.783 | 0.111 | 0.293 | 9.832 | 6.74 | 10.66 | 2.46 | 13.01 | 8.493 | 0.919 | 0.057 |
| 2004 | 360800 | 0.77 | 0.084 | 0.148 | 9.138 | 6.146 | 9.403 | 1.535 | 11.74 | 8.528 | 1.301 | 0.061 |
| 2004 | 360900 | 0.764 | 0.115 | 0.224 | 9.733 | 6.264 | 9.609 | 2.36 | 11.58 | 8.587 | 1.272 | 0.053 |
| 2004 | 361000 | 0.764 | 0.122 | 0.208 | 9.708 | 5.937 | 9.624 | 2.163 | 10.49 | 8.531 | 1.551 | 0.045 |
| 2004 | 361100 | 0.765 | 0.078 | 0.132 | 9.174 | 6.506 | 9.306 | 2.531 | 11.6 | 8.426 | 1.03 | 0.058 |
| 2004 | 420100 | 0.769 | 1.211 | 1.272 | 11.33 | 6.667 | 13.31 | 2.208 | 14.05 | 10.1 | 0.965 | 0.055 |
| 2004 | 420200 | 0.769 | 0.359 | 0.411 | 9.908 | 5.541 | 10.11 | 2.419 | 12.02 | 9.428 | 1.461 | 0.032 |
| 2004 | 420300 | 0.767 | 0.38 | 0.362 | 9.175 | 5.834 | 10.48 | 2.422 | 10.34 | 8.989 | 1.562 | 0.037 |
| 2004 | 420500 | 0.773 | 0.526 | 0.443 | 9.866 | 5.988 | 10.21 | 2.602 | 12.54 | 9.512 | 1.837 | 0.035 |
| 2004 | 420600 | 0.772 | 0.06 | 0.2 | 9.778 | 6.361 | 9.995 | 2.418 | 11.1 | 9.2 | 1.55 | 0.032 |
| 2004 | 420700 | 0.774 | 0.251 | 0.306 | 10.25 | 4.657 | 8.855 | 2.62 | 10.8 | 9.527 | 1.596 | 0.028 |
| 2004 | 420800 | 0.772 | 0.256 | 0.308 | 9.021 | 5.698 | 9.882 | 2.558 | 10.94 | 9.425 | 1.411 | 0.025 |
| 2004 | 420900 | 0.775 | 0.101 | 0.253 | 9.628 | 6.229 | 9.945 | 2.884 | 11.02 | 8.955 | 1.335 | 0.026 |
| 2004 | 421000 | 0.777 | 0.159 | 0.521 | 9.371 | 6.462 | 11.14 | 1.895 | 10.82 | 8.85 | 1.408 | 0.031 |
| 2004 | 421100 | 0.772 | 0.076 | 0.197 | 9.491 | 6.588 | 10.03 | 2.821 | 10.76 | 8.712 | 1.61 | 0.03 |
| 2004 | 421200 | 0.773 | 0.106 | 0.493 | 9.313 | 5.624 | 9.917 | 2.542 | 10.66 | 8.868 | 1.573 | 0.033 |
| 2004 | 421300 | 0.769 | 0.12 | 0.051 | 8.541 | 5.552 | 7.831 | 2.395 | 9.427 | 8.874 | 1.713 | 0.025 |
| 2004 | 430100 | 0.745 | 1.133 | 1.211 | 10.81 | 6.414 | 12.71 | 2.497 | 12.94 | 9.735 | 0.913 | 0.075 |
| 2004 | 430200 | 0.766 | 0.735 | 0.679 | 9.965 | 5.916 | 10.89 | 1.095 | 11.57 | 9.411 | 1.359 | 0.047 |
| 2004 | 430300 | 0.758 | 0.522 | 0.838 | 9.804 | 5.645 | 11.28 | 2.432 | 11.67 | 9.348 | 0.934 | 0.04 |
| 2004 | 430400 | 0.776 | 0.174 | 0.482 | 9.293 | 6.578 | 10.55 | 1.913 | 11.75 | 8.874 | 0.901 | 0.034 |
| 2004 | 430500 | 0.759 | 0.14 | 0.153 | 8.892 | 6.606 | 9.628 | 1.858 | 10.54 | 8.401 | 0.746 | 0.04 |
| 2004 | 430600 | 0.777 | 0.274 | 0.334 | 9.536 | 6.272 | 10.33 | 2.322 | 11.36 | 9.28 | 1.237 | 0.04 |
| 2004 | 430700 | 0.788 | 0.183 | 0.218 | 9.289 | 6.399 | 9.915 | 2.276 | 11.59 | 9.134 | 1.196 | 0.039 |
| 2004 | 430800 | 0.776 | 0.444 | 0.267 | 9.564 | 5.058 | 9.12 | 2.358 | 9.609 | 8.691 | 0.47 | 0.045 |
| 2004 | 430900 | 0.772 | 0.136 | 0.152 | 9.423 | 6.128 | 9.447 | 2.293 | 11.01 | 8.7 | 0.656 | 0.031 |
| 2004 | 431000 | 0.765 | 0.154 | 0.157 | 9.784 | 6.126 | 9.507 | 2.036 | 12.01 | 9.006 | 0.983 | 0.062 |
| 2004 | 431100 | 0.768 | 0.131 | 0.177 | 9.535 | 6.349 | 9.899 | 1.853 | 11.61 | 8.634 | 0.671 | 0.035 |
| 2004 | 431200 | 0.766 | 0.136 | 0.183 | 8.764 | 6.205 | 9.886 | 1.892 | 9.75 | 8.665 | 0.61 | 0.034 |
| 2004 | 431300 | 0.772 | 0.315 | 0.174 | 9.724 | 6.004 | 9.567 | 2.51 | 10.96 | 8.696 | 1.126 | 0.044 |
| 2004 | 500000 | 0.771 | 0.701 | 0.478 | 10.38 | 8.053 | 12.62 | 1.825 | 13.24 | 9.129 | 1.094 | 0.113 |
| 2004 | 510100 | 0.779 | 1.223 | 0.907 | 10.73 | 6.966 | 12.77 | 2.542 | 12.72 | 9.913 | 1.045 | 0.05 |
| 2004 | 510300 | 0.774 | 0.182 | 0.379 | 10.22 | 5.755 | 10.35 | 2.089 | 9.712 | 8.875 | 1.174 | 0.033 |
| 2004 | 510400 | 0.775 | 0.503 | 0.699 | 11.74 | 4.673 | 9.38 | 2.029 | 9.712 | 9.765 | 3.14 | 0.065 |
| 2004 | 510500 | 0.777 | 0.209 | 0.357 | 9.61 | 6.161 | 10.15 | 2.26 | 8.392 | 8.554 | 1.144 | 0.039 |
| 2004 | 510600 | 0.772 | 0.42 | 0.277 | 9.858 | 5.943 | 9.928 | 2.274 | 8.819 | 9.255 | 1.452 | 0.036 |
| 2004 | 510700 | 0.771 | 0.315 | 0.427 | 10.13 | 6.271 | 10.62 | 2.149 | 10.53 | 9.043 | 1.05 | 0.036 |
| 2004 | 510800 | 0.776 | 0.07 | 0.048 | 9.708 | 5.717 | 5.485 | 2.222 | 7.312 | 8.251 | 0.829 | 0.031 |
| 2004 | 510900 | 0.774 | 0.131 | 0.088 | 8.771 | 5.94 | 7.261 | 2.466 | 7.312 | 8.46 | 0.982 | 0.024 |
| 2004 | 511000 | 0.774 | 0.137 | 0.21 | 9.46 | 6.042 | 9.381 | 1.964 | 9.759 | 8.6 | 1.385 | 0.03 |
| 2004 | 511100 | 0.773 | 0.251 | 0.185 | 10.11 | 5.851 | 9.267 | 2.425 | 8.543 | 8.865 | 1.63 | 0.044 |
| 2004 | 511300 | 0.776 | 0.106 | 0.426 | 9.596 | 6.586 | 10.84 | 2.618 | 11.57 | 8.262 | 0.812 | 0.029 |
| 2004 | 511400 | 0.778 | 0.197 | 0.056 | 8.864 | 5.831 | 7.867 | 2.38 | 8.059 | 8.652 | 1.441 | 0.033 |
| 2004 | 511500 | 0.77 | 0.328 | 0.171 | 9.746 | 6.25 | 9.77 | 1.782 | 10.49 | 8.754 | 1.701 | 0.038 |
| 2004 | 511600 | 0.784 | 0.052 | 0.072 | 8.74 | 6.116 | 8.049 | 2.39 | 8.669 | 8.394 | 1.134 | 0.035 |
| 2004 | 511700 | 0.757 | 0.046 | 0.079 | 9.624 | 6.457 | 8.552 | 1.567 | 7.96 | 8.418 | 1.134 | 0.025 |
| 2004 | 511800 | 0.767 | 0.179 | 1.012 | 10.37 | 5.033 | 9.595 | 2.28 | 5.249 | 8.911 | 1.844 | 0.033 |
| 2004 | 511900 | 0.76 | 0.12 | 0 | 9.071 | 5.889 | 5.442 | 2.297 | 9.593 | 8.136 | 0.605 | 0.022 |
| 2004 | 512000 | 0.763 | 0.105 | 0 | 8.517 | 6.19 | 6.866 | 1.942 | 7.041 | 8.412 | 1.131 | 0.026 |
| 2004 | 520100 | 0.769 | 0.833 | 1.082 | 10.98 | 5.852 | 12.04 | 1.633 | 5.332 | 9.424 | 1.262 | 0.092 |
| 2004 | 520200 | 0.749 | 0.04 | 0.155 | 9.408 | 5.689 | 8.623 | 1.459 | 11.08 | 8.404 | 2.085 | 0.072 |
| 2004 | 520300 | 0.767 | 0.127 | 0.116 | 9.436 | 6.583 | 9.522 | 1.831 | 5.249 | 8.466 | 1.265 | 0.052 |
| 2004 | 520400 | 0.773 | 0.125 | 0.18 | 10 | 5.546 | 8.931 | 1.567 | 9.057 | 8.142 | 1.379 | 0.065 |
| 2004 | 530100 | 0.771 | 0.963 | 0.834 | 11.1 | 6.22 | 11.86 | 1.613 | 6.613 | 9.801 | 1.054 | 0.078 |
| 2004 | 530300 | 0.755 | 0.046 | 0.059 | 9.283 | 6.356 | 8.743 | 2.279 | 6.876 | 8.627 | 1.891 | 0.073 |
| 2004 | 530400 | 0.776 | 0.342 | 0.215 | 10.63 | 5.34 | 9.195 | 1.954 | 9.15 | 9.641 | 2.944 | 0.089 |
| 2004 | 530500 | 0.766 | 0.064 | 0.076 | 9.803 | 5.485 | 8.019 | 1.768 | 8.848 | 8.307 | 0.5 | 0.052 |
| 2004 | 530600 | 0.745 | 0.017 | 0.078 | 9.752 | 6.262 | 8.201 | 1.261 | 6.876 | 7.872 | 0.761 | 0.051 |
| 2004 | 530700 | 0.787 | 0.069 | 0.259 | 10.8 | 4.725 | 8.747 | 3.001 | 7.977 | 8.321 | 0.713 | 0.072 |
| 2004 | 530800 | 0.769 | 0.042 | 0.054 | 10.39 | 5.541 | 8.018 | 1.386 | 8.798 | 8.127 | 0.815 | 0.058 |
| 2004 | 530900 | 0.766 | 0.049 | 0.064 | 10.2 | 5.386 | 7.506 | 1.95 | 7.977 | 8.172 | 1.085 | 0.051 |
| 2005 | 310000 | 0.747 | 2.228 | 0.83 | 13.97 | 7.215 | 13 | 2.466 | 15.54 | 10.97 | 1.16 | 0.165 |
| 2005 | 320100 | 0.757 | 1.466 | 1.333 | 12.54 | 6.39 | 13.24 | 2.672 | 13.97 | 10.44 | 1.281 | 0.098 |
| 2005 | 320200 | 0.751 | 1.655 | 0.573 | 11.07 | 6.116 | 11.28 | 3.027 | 14.31 | 10.91 | 1.658 | 0.071 |
| 2005 | 320300 | 0.778 | 0.537 | 0.417 | 9.969 | 6.83 | 11.44 | 2.39 | 12.27 | 9.444 | 1.536 | 0.046 |
| 2005 | 320400 | 0.794 | 1.51 | 0.734 | 10.83 | 5.863 | 11.47 | 2.885 | 13.3 | 10.41 | 1.79 | 0.078 |
| 2005 | 320500 | 0.754 | 1.792 | 0.711 | 11.3 | 6.409 | 11.6 | 2.945 | 15.25 | 11.01 | 2.136 | 0.083 |
| 2005 | 320600 | 0.76 | 0.707 | 0.372 | 10.4 | 6.648 | 10.95 | 2.514 | 14.04 | 9.69 | 1.674 | 0.053 |
| 2005 | 320700 | 0.765 | 0.388 | 0.276 | 10.46 | 6.157 | 10.22 | 2.615 | 12.32 | 9.162 | 1.457 | 0.053 |
| 2005 | 320800 | 0.759 | 0.398 | 0.378 | 9.383 | 6.269 | 10.75 | 2.412 | 10.92 | 9.235 | 1.765 | 0.048 |
| 2005 | 320900 | 0.699 | 0.353 | 0.239 | 9.536 | 6.683 | 10.39 | 2.34 | 11.79 | 9.376 | 1.592 | 0.038 |
| 2005 | 321000 | 0.776 | 0.985 | 0.564 | 10.09 | 6.123 | 11.03 | 2.759 | 12.97 | 9.802 | 1.569 | 0.057 |
| 2005 | 321100 | 0.721 | 1.181 | 0.755 | 10.96 | 5.59 | 11.07 | 2.726 | 13.1 | 10.32 | 1.74 | 0.055 |
| 2005 | 321200 | 0.735 | 0.766 | 0.217 | 9.943 | 6.219 | 9.905 | 2.899 | 12.83 | 9.586 | 1.666 | 0.06 |
| 2005 | 321300 | 0.659 | 0.098 | 0.065 | 8.054 | 6.263 | 7.726 | 3.095 | 10.22 | 8.817 | 1.598 | 0.037 |
| 2005 | 330100 | 0.779 | 1.88 | 1.084 | 11.88 | 6.493 | 12.7 | 2.788 | 14.15 | 10.63 | 1.404 | 0.089 |
| 2005 | 330200 | 0.762 | 1.98 | 0.65 | 12.06 | 6.322 | 11.61 | 2.566 | 14.45 | 10.63 | 1.691 | 0.089 |
| 2005 | 330300 | 0.766 | 1.54 | 0.343 | 11.38 | 6.62 | 10.82 | 2.573 | 12.59 | 9.949 | 1.707 | 0.068 |
| 2005 | 330400 | 0.761 | 1.496 | 0.26 | 12.06 | 5.812 | 9.966 | 3.134 | 13.76 | 10.39 | 2.059 | 0.059 |
| 2005 | 330500 | 0.766 | 1.013 | 0.364 | 11.55 | 5.551 | 9.705 | 2.994 | 13.19 | 10.1 | 1.654 | 0.061 |
| 2005 | 330600 | 0.743 | 1.086 | 0.367 | 11.94 | 6.076 | 10.33 | 2.552 | 13.51 | 10.38 | 1.959 | 0.053 |
| 2005 | 330700 | 0.727 | 1.758 | 0.462 | 11.6 | 6.118 | 10.76 | 3.294 | 12.83 | 10.04 | 1.589 | 0.064 |
| 2005 | 330800 | 0.769 | 0.367 | 0.221 | 10.78 | 5.504 | 9.173 | 3.063 | 10.12 | 9.416 | 1.403 | 0.065 |
| 2005 | 330900 | 0.747 | 0.514 | 0.579 | 12.07 | 4.572 | 9.509 | 2.697 | 10.15 | 10.03 | 1.106 | 0.08 |
| 2005 | 331000 | 0.773 | 1.45 | 0.208 | 11.53 | 6.328 | 9.802 | 2.595 | 12.53 | 10.04 | 2.029 | 0.055 |
| 2005 | 331100 | 0.773 | 0.624 | 0.377 | 11.76 | 5.527 | 9.857 | 2.61 | 9.675 | 9.321 | 1.321 | 0.069 |
| 2005 | 340100 | 0.764 | 0.851 | 1.279 | 10.6 | 6.122 | 12.4 | 2.769 | 12.72 | 9.53 | 1.28 | 0.091 |
| 2005 | 340200 | 0.779 | 0.564 | 1.087 | 11.2 | 5.424 | 11.41 | 2.596 | 12.26 | 9.722 | 1.744 | 0.075 |
| 2005 | 340300 | 0.773 | 0.306 | 0.538 | 9.385 | 5.856 | 10.52 | 2.262 | 11.46 | 9.01 | 1.239 | 0.048 |
| 2005 | 340400 | 0.772 | 0.227 | 0.803 | 8.906 | 5.463 | 10.71 | 2.027 | 11.69 | 9.264 | 1.39 | 0.07 |
| 2005 | 340500 | 0.785 | 0.675 | 0.701 | 10.21 | 4.833 | 9.977 | 2.647 | 10.58 | 9.9 | 2.562 | 0.103 |
| 2005 | 340600 | 0.774 | 0.174 | 0.361 | 9.857 | 5.35 | 10.05 | 2.185 | 8.408 | 9.036 | 1.656 | 0.066 |
| 2005 | 340700 | 0.75 | 0.493 | 0.78 | 11.42 | 4.28 | 9.646 | 2.233 | 10.8 | 9.831 | 1.734 | 0.074 |
| 2005 | 340800 | 0.765 | 0.13 | 0.172 | 9.325 | 6.406 | 9.663 | 2.531 | 10.51 | 8.826 | 1.499 | 0.048 |
| 2005 | 341000 | 0.784 | 0.186 | 0.302 | 10.48 | 4.99 | 9.253 | 2.078 | 10.67 | 9.129 | 0.827 | 0.061 |
| 2005 | 341100 | 0.761 | 0.172 | 0.307 | 9.888 | 6.077 | 10.27 | 2.574 | 9.9 | 9.114 | 1.286 | 0.032 |
| 2005 | 341200 | 0.765 | 0.082 | 0.127 | 9.325 | 6.838 | 9.765 | 2.325 | 10.49 | 8.11 | 0.847 | 0.041 |
| 2005 | 341300 | 0.769 | 0.108 | 0.104 | 9.008 | 6.404 | 9.697 | 2.554 | 10.01 | 8.473 | 0.71 | 0.025 |
| 2005 | 341500 | 0.761 | 0.112 | 0.144 | 9.436 | 6.524 | 9.777 | 2.682 | 9.414 | 8.298 | 1.058 | 0.043 |
| 2005 | 341600 | 0.763 | 0.099 | 0.071 | 8.303 | 6.31 | 8.294 | 2.752 | 8.518 | 8.422 | 0.78 | 0.025 |
| 2005 | 341700 | 0.771 | 0.12 | 0.403 | 8.502 | 5.052 | 8.92 | 2.279 | 10.33 | 8.743 | 1.151 | 0.072 |
| 2005 | 341800 | 0.751 | 0.29 | 0.048 | 9.615 | 5.612 | 7.445 | 2.488 | 10.83 | 9.18 | 1.142 | 0.043 |
| 2005 | 360100 | 0.749 | 0.783 | 1.517 | 10.56 | 6.164 | 12.89 | 2.052 | 13.52 | 9.796 | 1.427 | 0.069 |
| 2005 | 360200 | 0.768 | 0.421 | 0.646 | 10.11 | 5.016 | 9.856 | 2.072 | 10.64 | 9.384 | 1.645 | 0.046 |
| 2005 | 360300 | 0.736 | 0.321 | 0.159 | 10.66 | 5.191 | 8.717 | 2.441 | 10.82 | 9.244 | 2.218 | 0.063 |
| 2005 | 360400 | 0.762 | 0.194 | 0.644 | 10.17 | 6.145 | 11.27 | 1.987 | 12.49 | 9.031 | 1.589 | 0.056 |
| 2005 | 360500 | 0.756 | 0.373 | 0.292 | 10.84 | 4.707 | 8.973 | 2.533 | 10.98 | 9.425 | 1.795 | 0.073 |
| 2005 | 360600 | 0.765 | 0.39 | 0.18 | 10.04 | 4.711 | 8.016 | 2.151 | 10.9 | 9.168 | 1.455 | 0.064 |
| 2005 | 360700 | 0.761 | 0.106 | 0.33 | 9.972 | 6.74 | 10.93 | 2.416 | 13.13 | 8.611 | 0.966 | 0.057 |
| 2005 | 360800 | 0.762 | 0.133 | 0.168 | 9.521 | 6.142 | 9.635 | 1.82 | 11.94 | 8.629 | 1.4 | 0.065 |
| 2005 | 360900 | 0.766 | 0.148 | 0.326 | 9.749 | 6.269 | 9.815 | 2.376 | 12.07 | 8.689 | 1.369 | 0.055 |
| 2005 | 361000 | 0.766 | 0.135 | 0.243 | 9.775 | 5.944 | 9.649 | 2.255 | 11.34 | 8.633 | 1.669 | 0.046 |
| 2005 | 361100 | 0.78 | 0.097 | 0.143 | 9.423 | 6.516 | 9.527 | 2.589 | 11.78 | 8.528 | 1.108 | 0.065 |
| 2005 | 420100 | 0.769 | 1.29 | 1.278 | 11.56 | 6.686 | 13.45 | 2.22 | 14.17 | 10.22 | 1.014 | 0.055 |
| 2005 | 420200 | 0.762 | 0.395 | 0.516 | 10.06 | 5.53 | 10.27 | 2.442 | 12.12 | 9.546 | 1.539 | 0.035 |
| 2005 | 420300 | 0.768 | 0.331 | 0.378 | 9.478 | 5.84 | 10.49 | 2.452 | 9.631 | 9.107 | 1.641 | 0.037 |
| 2005 | 420500 | 0.769 | 0.539 | 0.477 | 9.97 | 5.988 | 10.5 | 2.662 | 12.01 | 9.63 | 1.931 | 0.036 |
| 2005 | 420600 | 0.722 | 0.102 | 0.249 | 10.18 | 6.358 | 10.05 | 2.445 | 10.3 | 9.318 | 1.632 | 0.029 |
| 2005 | 420700 | 0.77 | 0.268 | 0.337 | 10.2 | 4.647 | 9.003 | 2.648 | 10.06 | 9.644 | 1.681 | 0.031 |
| 2005 | 420800 | 0.764 | 0.285 | 0.271 | 9.09 | 5.674 | 9.874 | 2.589 | 11.14 | 9.542 | 1.483 | 0.023 |
| 2005 | 420900 | 0.764 | 0.121 | 0.32 | 9.7 | 6.227 | 10.04 | 2.918 | 11.15 | 9.073 | 1.403 | 0.025 |
| 2005 | 421000 | 0.811 | 0.197 | 0.559 | 9.484 | 6.463 | 11.49 | 1.895 | 10.94 | 8.968 | 1.48 | 0.025 |
| 2005 | 421100 | 0.759 | 0.07 | 0.202 | 9.634 | 6.588 | 10.43 | 2.822 | 10.96 | 8.83 | 1.695 | 0.027 |
| 2005 | 421200 | 0.738 | 0.113 | 0.497 | 9.465 | 5.623 | 10.16 | 2.544 | 9.102 | 8.986 | 1.653 | 0.034 |
| 2005 | 421300 | 0.765 | 0.137 | 0.084 | 8.866 | 5.532 | 8.392 | 2.403 | 9.597 | 8.991 | 1.804 | 0.022 |
| 2005 | 430100 | 0.763 | 1.224 | 1.351 | 10.94 | 6.431 | 12.89 | 2.595 | 13.51 | 9.841 | 0.958 | 0.089 |
| 2005 | 430200 | 0.784 | 0.843 | 0.812 | 10.05 | 5.92 | 11.08 | 1.297 | 11.79 | 9.528 | 1.431 | 0.05 |
| 2005 | 430300 | 0.77 | 0.644 | 0.934 | 10.25 | 5.649 | 11.39 | 2.555 | 11.76 | 9.454 | 0.98 | 0.044 |
| 2005 | 430400 | 0.748 | 0.23 | 0.477 | 9.466 | 6.583 | 10.55 | 1.981 | 11.86 | 8.98 | 0.945 | 0.036 |
| 2005 | 430500 | 0.764 | 0.16 | 0.171 | 9.275 | 6.612 | 9.736 | 1.921 | 10.71 | 8.508 | 0.783 | 0.039 |
| 2005 | 430600 | 0.765 | 0.27 | 0.396 | 9.619 | 6.276 | 10.36 | 2.447 | 11.16 | 9.398 | 1.303 | 0.039 |
| 2005 | 430700 | 0.769 | 0.173 | 0.228 | 9.486 | 6.401 | 9.859 | 2.466 | 11.51 | 9.252 | 1.259 | 0.04 |
| 2005 | 430800 | 0.757 | 0.223 | 0.228 | 10.01 | 5.076 | 9.174 | 2.395 | 9.136 | 8.809 | 0.495 | 0.046 |
| 2005 | 430900 | 0.728 | 0.205 | 0.194 | 9.743 | 6.133 | 9.687 | 2.29 | 10.25 | 8.806 | 0.689 | 0.03 |
| 2005 | 431000 | 0.788 | 0.203 | 0.196 | 9.936 | 6.133 | 9.794 | 2.043 | 12.21 | 9.112 | 1.031 | 0.065 |
| 2005 | 431100 | 0.774 | 0.148 | 0.213 | 9.736 | 6.354 | 9.902 | 2.138 | 11.74 | 8.74 | 0.704 | 0.036 |
| 2005 | 431200 | 0.758 | 0.119 | 0.197 | 9.039 | 6.211 | 10.11 | 1.905 | 9.65 | 8.783 | 0.643 | 0.035 |
| 2005 | 431300 | 0.794 | 0.281 | 0.111 | 9.82 | 6.01 | 9.024 | 2.538 | 10.93 | 8.814 | 1.186 | 0.046 |
| 2005 | 500000 | 0.75 | 0.679 | 0.515 | 10.44 | 8.061 | 12.72 | 1.893 | 13.27 | 9.235 | 1.148 | 0.131 |
| 2005 | 510100 | 0.757 | 1.245 | 1.082 | 10.83 | 6.987 | 12.97 | 2.547 | 12.96 | 10.05 | 1.061 | 0.058 |
| 2005 | 510300 | 0.758 | 0.138 | 0.403 | 10.26 | 5.76 | 10.29 | 2.369 | 8.167 | 9.013 | 1.192 | 0.032 |
| 2005 | 510400 | 0.707 | 0.544 | 0.729 | 11.96 | 4.682 | 9.539 | 2.046 | 8.167 | 9.903 | 3.189 | 0.086 |
| 2005 | 510500 | 0.765 | 0.114 | 0.362 | 9.392 | 6.173 | 10.11 | 2.301 | 8.273 | 8.692 | 1.162 | 0.04 |
| 2005 | 510600 | 0.759 | 0.446 | 0.363 | 9.96 | 5.946 | 10.42 | 2.321 | 8.734 | 9.393 | 1.475 | 0.041 |
| 2005 | 510700 | 0.736 | 0.285 | 0.499 | 9.979 | 6.274 | 10.95 | 2.178 | 10.64 | 9.181 | 1.066 | 0.037 |
| 2005 | 510800 | 0.759 | 0.112 | 0.122 | 9.921 | 5.718 | 8.628 | 2.213 | 7.233 | 8.389 | 0.842 | 0.029 |
| 2005 | 510900 | 0.765 | 0.137 | 0.098 | 9.288 | 5.926 | 8.78 | 2.462 | 7.233 | 8.598 | 0.997 | 0.024 |
| 2005 | 511000 | 0.751 | 0.16 | 0.222 | 9.429 | 6.041 | 9.529 | 2.048 | 9.988 | 8.706 | 1.453 | 0.03 |
| 2005 | 511100 | 0.773 | 0.273 | 0.193 | 10.39 | 5.852 | 9.322 | 2.485 | 8.341 | 8.972 | 1.71 | 0.048 |
| 2005 | 511300 | 0.765 | 0.097 | 0.484 | 9.671 | 6.592 | 10.87 | 2.609 | 9.985 | 8.368 | 0.852 | 0.028 |
| 2005 | 511400 | 0.765 | 0.17 | 0.058 | 9.24 | 5.831 | 8.011 | 2.412 | 7.947 | 8.79 | 1.464 | 0.032 |
| 2005 | 511500 | 0.77 | 0.319 | 0.191 | 9.806 | 6.252 | 9.863 | 1.77 | 8.726 | 8.861 | 1.785 | 0.04 |
| 2005 | 511600 | 0.745 | 0.054 | 0.072 | 8.92 | 6.119 | 8.132 | 2.43 | 9.037 | 8.532 | 1.152 | 0.035 |
| 2005 | 511700 | 0.779 | 0.044 | 0.116 | 9.687 | 6.462 | 8.726 | 1.548 | 8.122 | 8.556 | 1.152 | 0.027 |
| 2005 | 511800 | 0.784 | 0.099 | 1.004 | 10.61 | 5.039 | 9.67 | 2.373 | 5.239 | 9.049 | 1.873 | 0.035 |
| 2005 | 511900 | 0.761 | 0.046 | 0 | 9.373 | 5.894 | 5.442 | 2.293 | 9.62 | 8.242 | 0.635 | 0.019 |
| 2005 | 512000 | 0.744 | 0.105 | 0 | 8.811 | 6.192 | 6.866 | 2.026 | 7.203 | 8.55 | 1.149 | 0.026 |
| 2005 | 520100 | 0.768 | 1.012 | 1.036 | 11.08 | 5.86 | 12.24 | 1.852 | 6.728 | 9.538 | 1.334 | 0.1 |
| 2005 | 520200 | 0.761 | 0.102 | 0.173 | 9.497 | 5.694 | 8.353 | 1.459 | 11.12 | 8.519 | 2.204 | 0.087 |
| 2005 | 520300 | 0.767 | 0.111 | 0.147 | 9.745 | 6.587 | 9.703 | 1.81 | 9.638 | 8.58 | 1.337 | 0.056 |
| 2005 | 520400 | 0.793 | 0.086 | 0.184 | 10.06 | 5.549 | 8.935 | 1.468 | 7.705 | 8.256 | 1.458 | 0.073 |
| 2005 | 530100 | 0.742 | 1.004 | 1.028 | 11.45 | 6.231 | 12.14 | 1.629 | 6.603 | 9.916 | 1.114 | 0.089 |
| 2005 | 530300 | 0.77 | 0.061 | 0.094 | 9.43 | 6.376 | 9.291 | 2.409 | 6.971 | 8.741 | 1.998 | 0.083 |
| 2005 | 530400 | 0.74 | 0.285 | 0.218 | 10.74 | 5.343 | 9.076 | 1.987 | 9.144 | 9.755 | 3.112 | 0.087 |
| 2005 | 530500 | 0.769 | 0.083 | 0.086 | 9.912 | 5.491 | 8.284 | 1.825 | 6.947 | 8.422 | 0.529 | 0.06 |
| 2005 | 530600 | 0.764 | 0.041 | 0.074 | 9.878 | 6.27 | 8.191 | 1.356 | 6.971 | 7.987 | 0.804 | 0.057 |
| 2005 | 530700 | 0.737 | 0.22 | 0.264 | 11.25 | 4.734 | 9.014 | 2.954 | 9.282 | 8.435 | 0.754 | 0.078 |
| 2005 | 530800 | 0.776 | 0.057 | 0.1 | 10.42 | 5.547 | 8.369 | 1.406 | 9.324 | 8.242 | 0.844 | 0.065 |
| 2005 | 530900 | 0.747 | 0.023 | 0.075 | 10.37 | 5.392 | 7.877 | 2.043 | 9.282 | 8.286 | 1.147 | 0.051 |
| 2006 | 310000 | 0.78 | 2.447 | 0.863 | 14.21 | 7.221 | 13.05 | 2.471 | 15.55 | 11.12 | 1.212 | 0.163 |
| 2006 | 320100 | 0.767 | 1.654 | 1.437 | 12.93 | 6.409 | 13.34 | 2.841 | 14.01 | 10.55 | 1.349 | 0.099 |
| 2006 | 320200 | 0.756 | 1.876 | 0.632 | 11.64 | 6.126 | 11.42 | 3.05 | 14.6 | 11.02 | 1.746 | 0.075 |
| 2006 | 320300 | 0.777 | 0.778 | 0.463 | 10.27 | 6.84 | 11.55 | 2.232 | 12.18 | 9.56 | 1.618 | 0.051 |
| 2006 | 320400 | 0.766 | 1.682 | 0.77 | 10.97 | 5.871 | 11.59 | 2.633 | 13.81 | 10.52 | 1.885 | 0.084 |
| 2006 | 320500 | 0.745 | 2.035 | 0.786 | 11.35 | 6.423 | 11.76 | 2.716 | 15.4 | 11.12 | 2.249 | 0.091 |
| 2006 | 320600 | 0.762 | 1.169 | 0.419 | 10.39 | 6.646 | 11.09 | 2.473 | 14.53 | 9.806 | 1.763 | 0.059 |
| 2006 | 320700 | 0.764 | 0.476 | 0.291 | 10.28 | 6.173 | 10.24 | 2.911 | 12.53 | 9.286 | 1.535 | 0.064 |
| 2006 | 320800 | 0.784 | 0.476 | 0.438 | 9.697 | 6.278 | 10.91 | 2.547 | 11.43 | 9.351 | 1.859 | 0.057 |
| 2006 | 320900 | 0.77 | 0.441 | 0.273 | 9.578 | 6.691 | 10.55 | 2.424 | 12.47 | 9.492 | 1.677 | 0.043 |
| 2006 | 321000 | 0.778 | 1.201 | 0.608 | 10.45 | 6.128 | 11.15 | 2.673 | 13.32 | 9.918 | 1.652 | 0.063 |
| 2006 | 321100 | 0.762 | 1.462 | 0.81 | 10.91 | 5.594 | 11.2 | 2.658 | 13.27 | 10.44 | 1.832 | 0.061 |
| 2006 | 321200 | 0.771 | 0.795 | 0.272 | 10.17 | 6.222 | 10.15 | 2.84 | 13.17 | 9.702 | 1.755 | 0.068 |
| 2006 | 321300 | 0.767 | 0.157 | 0.075 | 8.098 | 6.272 | 7.591 | 3.175 | 10.56 | 8.933 | 1.682 | 0.047 |
| 2006 | 330100 | 0.717 | 2.162 | 1.107 | 12.1 | 6.502 | 12.77 | 2.741 | 14.4 | 10.76 | 1.478 | 0.094 |
| 2006 | 330200 | 0.727 | 2.292 | 0.68 | 12.25 | 6.329 | 11.71 | 1.985 | 14.48 | 10.75 | 1.781 | 0.094 |
| 2006 | 330300 | 0.729 | 1.653 | 0.392 | 11.63 | 6.629 | 10.95 | 2.316 | 12.82 | 10.07 | 1.797 | 0.07 |
| 2006 | 330400 | 0.68 | 1.969 | 0.292 | 12.47 | 5.816 | 10.15 | 2.715 | 13.79 | 10.52 | 2.168 | 0.063 |
| 2006 | 330500 | 0.709 | 1.699 | 0.351 | 11.75 | 5.553 | 9.841 | 2.622 | 13.31 | 10.23 | 1.742 | 0.067 |
| 2006 | 330600 | 0.719 | 1.771 | 0.389 | 12.15 | 6.076 | 10.55 | 2.769 | 13.56 | 10.5 | 2.063 | 0.058 |
| 2006 | 330700 | 0.714 | 2.252 | 0.491 | 11.72 | 6.124 | 10.86 | 3.055 | 12.92 | 10.14 | 1.661 | 0.067 |
| 2006 | 330800 | 0.76 | 0.541 | 0.221 | 11.09 | 5.508 | 9.125 | 2.958 | 10.37 | 9.464 | 1.557 | 0.067 |
| 2006 | 330900 | 0.764 | 0.821 | 0.56 | 12.24 | 4.57 | 9.759 | 2.11 | 10.59 | 10.15 | 1.165 | 0.092 |
| 2006 | 331000 | 0.748 | 1.808 | 0.303 | 11.64 | 6.336 | 9.956 | 2.476 | 12.71 | 10.16 | 2.137 | 0.058 |
| 2006 | 331100 | 0.765 | 0.985 | 0.305 | 12.01 | 5.532 | 10 | 2.21 | 9.639 | 9.445 | 1.391 | 0.075 |
| 2006 | 340100 | 0.745 | 0.918 | 1.347 | 10.82 | 6.152 | 12.52 | 2.843 | 13.26 | 9.635 | 1.338 | 0.11 |
| 2006 | 340200 | 0.788 | 0.644 | 1.194 | 11.44 | 5.434 | 11.61 | 2.535 | 12.52 | 9.827 | 1.823 | 0.082 |
| 2006 | 340300 | 0.76 | 0.334 | 0.579 | 9.741 | 5.865 | 10.65 | 2.423 | 11.64 | 9.114 | 1.295 | 0.056 |
| 2006 | 340400 | 0.72 | 0.251 | 0.772 | 8.862 | 5.472 | 10.8 | 2.063 | 11.87 | 9.368 | 1.453 | 0.079 |
| 2006 | 340500 | 0.783 | 0.814 | 0.787 | 10.79 | 4.841 | 10.16 | 2.773 | 11.14 | 10 | 2.679 | 0.109 |
| 2006 | 340600 | 0.75 | 0.234 | 0.493 | 9.655 | 5.358 | 10.06 | 2.235 | 10.2 | 9.141 | 1.731 | 0.069 |
| 2006 | 340700 | 0.752 | 0.48 | 0.836 | 11.8 | 4.292 | 9.908 | 2.556 | 11.29 | 9.935 | 1.813 | 0.092 |
| 2006 | 340800 | 0.754 | 0.134 | 0.196 | 9.785 | 6.41 | 9.778 | 2.341 | 10.74 | 8.93 | 1.568 | 0.053 |
| 2006 | 341000 | 0.783 | 0.224 | 0.322 | 10.67 | 4.994 | 9.373 | 2.64 | 10.87 | 9.235 | 0.864 | 0.066 |
| 2006 | 341100 | 0.747 | 0.184 | 0.327 | 10.22 | 6.087 | 10.39 | 2.64 | 11.55 | 9.219 | 1.345 | 0.037 |
| 2006 | 341200 | 0.759 | 0.105 | 0.131 | 9.271 | 6.861 | 9.786 | 2.404 | 10.72 | 8.215 | 0.886 | 0.045 |
| 2006 | 341300 | 0.757 | 0.086 | 0.119 | 9.565 | 6.413 | 9.868 | 1.573 | 9.882 | 8.578 | 0.742 | 0.029 |
| 2006 | 341500 | 0.742 | 0.114 | 0.173 | 9.576 | 6.532 | 9.979 | 2.453 | 10.23 | 8.403 | 1.106 | 0.05 |
| 2006 | 341600 | 0.754 | 0.127 | 0.074 | 8.59 | 6.331 | 8.429 | 3.134 | 9.207 | 8.526 | 0.816 | 0.03 |
| 2006 | 341700 | 0.776 | 0.142 | 0.406 | 8.89 | 5.057 | 8.976 | 2.53 | 10.69 | 8.847 | 1.203 | 0.083 |
| 2006 | 341800 | 0.777 | 0.192 | 0.047 | 9.565 | 5.614 | 7.811 | 2.352 | 11.12 | 9.285 | 1.194 | 0.054 |
| 2006 | 360100 | 0.733 | 0.835 | 1.575 | 10.78 | 6.182 | 13.13 | 2.049 | 13.64 | 9.902 | 1.49 | 0.071 |
| 2006 | 360200 | 0.745 | 0.306 | 0.688 | 10.24 | 5.034 | 10.08 | 2.055 | 10.77 | 9.491 | 1.717 | 0.047 |
| 2006 | 360300 | 0.733 | 0.313 | 0.262 | 10.87 | 5.204 | 8.882 | 2.464 | 10.84 | 9.351 | 2.315 | 0.066 |
| 2006 | 360400 | 0.757 | 0.158 | 0.641 | 10.33 | 6.158 | 11.34 | 2.053 | 12.51 | 9.137 | 1.659 | 0.057 |
| 2006 | 360500 | 0.752 | 0.356 | 0.311 | 11.07 | 4.722 | 9.299 | 2.667 | 11.5 | 9.531 | 1.873 | 0.085 |
| 2006 | 360600 | 0.742 | 0.449 | 0.184 | 9.895 | 4.727 | 7.916 | 2.383 | 11.07 | 9.274 | 1.519 | 0.072 |
| 2006 | 360700 | 0.77 | 0.105 | 0.365 | 10.11 | 6.758 | 11.06 | 2.369 | 13.2 | 8.717 | 1.008 | 0.06 |
| 2006 | 360800 | 0.753 | 0.135 | 0.19 | 9.944 | 6.155 | 9.719 | 2.189 | 12.06 | 8.736 | 1.461 | 0.067 |
| 2006 | 360900 | 0.742 | 0.268 | 0.288 | 10.03 | 6.282 | 10.09 | 2.411 | 12.14 | 8.795 | 1.428 | 0.059 |
| 2006 | 361000 | 0.742 | 0.148 | 0.363 | 10.3 | 5.95 | 10.37 | 2.281 | 11.45 | 8.739 | 1.742 | 0.056 |
| 2006 | 361100 | 0.735 | 0.089 | 0.14 | 9.659 | 6.537 | 9.694 | 2.708 | 11.91 | 8.634 | 1.156 | 0.068 |
| 2006 | 420100 | 0.751 | 1.478 | 1.364 | 11.79 | 6.708 | 13.52 | 2.172 | 14.28 | 10.36 | 1.061 | 0.074 |
| 2006 | 420200 | 0.753 | 0.461 | 0.533 | 10.29 | 5.539 | 10.29 | 2.45 | 12.16 | 9.682 | 1.609 | 0.039 |
| 2006 | 420300 | 0.75 | 0.452 | 0.348 | 9.637 | 5.848 | 10.5 | 2.278 | 10.22 | 9.213 | 1.713 | 0.038 |
| 2006 | 420500 | 0.76 | 0.514 | 0.531 | 10.23 | 5.992 | 10.58 | 2.603 | 11.97 | 9.766 | 2.019 | 0.039 |
| 2006 | 420600 | 0.759 | 0.149 | 0.232 | 10.24 | 6.361 | 10.03 | 2.584 | 11.21 | 9.454 | 1.706 | 0.029 |
| 2006 | 420700 | 0.756 | 0.391 | 0.356 | 11.01 | 4.668 | 9.09 | 2.673 | 10.88 | 9.781 | 1.757 | 0.036 |
| 2006 | 420800 | 0.758 | 0.282 | 0.282 | 9.057 | 5.687 | 9.867 | 2.776 | 11.17 | 9.679 | 1.551 | 0.022 |
| 2006 | 420900 | 0.763 | 0.155 | 0.3 | 9.939 | 6.243 | 10.09 | 2.903 | 11.24 | 9.209 | 1.467 | 0.026 |
| 2006 | 421000 | 0.766 | 0.3 | 0.614 | 9.682 | 6.473 | 11.67 | 2.426 | 11.15 | 9.104 | 1.547 | 0.023 |
| 2006 | 421100 | 0.757 | 0.084 | 0.208 | 9.976 | 6.592 | 10.36 | 3.106 | 11.23 | 8.966 | 1.772 | 0.028 |
| 2006 | 421200 | 0.762 | 0.151 | 0.512 | 9.755 | 5.641 | 10.29 | 2.509 | 10.8 | 9.107 | 1.76 | 0.035 |
| 2006 | 421300 | 0.754 | 0.141 | 0.103 | 9.366 | 5.528 | 8.766 | 2.377 | 9.706 | 9.128 | 1.886 | 0.021 |
| 2006 | 430100 | 0.743 | 1.688 | 1.381 | 11.04 | 6.447 | 12.94 | 2.612 | 13.77 | 9.949 | 0.978 | 0.097 |
| 2006 | 430200 | 0.754 | 0.891 | 0.797 | 9.983 | 5.932 | 11.12 | 2.275 | 11.95 | 9.636 | 1.461 | 0.054 |
| 2006 | 430300 | 0.74 | 0.732 | 1.051 | 10.19 | 5.676 | 11.51 | 2.55 | 11.91 | 9.562 | 1 | 0.049 |
| 2006 | 430400 | 0.765 | 0.245 | 0.472 | 9.812 | 6.588 | 10.55 | 2.073 | 12 | 9.088 | 0.965 | 0.039 |
| 2006 | 430500 | 0.737 | 0.173 | 0.19 | 9.307 | 6.615 | 9.882 | 1.975 | 10.86 | 8.616 | 0.8 | 0.04 |
| 2006 | 430600 | 0.762 | 0.316 | 0.395 | 10.05 | 6.289 | 10.42 | 2.341 | 10.96 | 9.534 | 1.362 | 0.04 |
| 2006 | 430700 | 0.766 | 0.238 | 0.223 | 9.698 | 6.41 | 9.859 | 2.587 | 11.7 | 9.36 | 1.286 | 0.043 |
| 2006 | 430800 | 0.77 | 0.329 | 0.281 | 9.955 | 5.084 | 9.586 | 2.4 | 9.193 | 8.917 | 0.505 | 0.051 |
| 2006 | 430900 | 0.752 | 0.203 | 0.234 | 9.952 | 6.138 | 9.823 | 1.589 | 10.4 | 8.914 | 0.703 | 0.034 |
| 2006 | 431000 | 0.777 | 0.234 | 0.201 | 10.28 | 6.138 | 9.892 | 2.153 | 12.27 | 9.22 | 1.053 | 0.064 |
| 2006 | 431100 | 0.75 | 0.167 | 0.209 | 9.922 | 6.359 | 9.942 | 2.332 | 12.1 | 8.848 | 0.719 | 0.037 |
| 2006 | 431200 | 0.747 | 0.119 | 0.213 | 9.242 | 6.215 | 10.25 | 1.939 | 9.633 | 8.891 | 0.656 | 0.038 |
| 2006 | 431300 | 0.762 | 0.319 | 0.122 | 9.991 | 6.021 | 9.15 | 2.538 | 11.18 | 8.95 | 1.24 | 0.05 |
| 2006 | 500000 | 0.765 | 0.817 | 0.571 | 10.65 | 8.071 | 12.84 | 2.097 | 13.46 | 9.383 | 1.208 | 0.154 |
| 2006 | 510100 | 0.759 | 1.609 | 1.252 | 11.07 | 7.006 | 13.15 | 2.505 | 13.23 | 10.2 | 1.116 | 0.067 |
| 2006 | 510300 | 0.763 | 0.277 | 0.44 | 10.47 | 5.768 | 10.33 | 1.996 | 8.582 | 9.161 | 1.254 | 0.034 |
| 2006 | 510400 | 0.762 | 0.524 | 0.593 | 12.13 | 4.691 | 9.657 | 2.071 | 8.582 | 10.05 | 3.355 | 0.091 |
| 2006 | 510500 | 0.767 | 0.2 | 0.376 | 9.678 | 6.182 | 10.2 | 2.246 | 8.727 | 8.84 | 1.223 | 0.044 |
| 2006 | 510600 | 0.764 | 0.468 | 0.366 | 10.27 | 5.95 | 10.84 | 2.316 | 9.665 | 9.541 | 1.552 | 0.042 |
| 2006 | 510700 | 0.759 | 0.352 | 0.554 | 9.989 | 6.279 | 11.17 | 2.466 | 10.79 | 9.329 | 1.122 | 0.041 |
| 2006 | 510800 | 0.755 | 0.099 | 0.119 | 9.971 | 5.724 | 8.855 | 2.316 | 6.821 | 8.538 | 0.886 | 0.034 |
| 2006 | 510900 | 0.759 | 0.134 | 0.108 | 9.763 | 5.933 | 8.989 | 2.195 | 6.821 | 8.725 | 1.031 | 0.027 |
| 2006 | 511000 | 0.762 | 0.12 | 0.245 | 9.862 | 6.045 | 9.61 | 1.637 | 10.2 | 8.855 | 1.529 | 0.031 |
| 2006 | 511100 | 0.757 | 0.29 | 0.202 | 10.41 | 5.858 | 9.334 | 2.513 | 8.435 | 9.12 | 1.8 | 0.054 |
| 2006 | 511300 | 0.764 | 0.142 | 0.491 | 9.841 | 6.598 | 10.71 | 2.208 | 10.43 | 8.517 | 0.896 | 0.031 |
| 2006 | 511400 | 0.778 | 0.224 | 0.1 | 9.709 | 5.835 | 8.525 | 2.5 | 7.925 | 8.938 | 1.54 | 0.034 |
| 2006 | 511500 | 0.755 | 0.37 | 0.219 | 10.07 | 6.26 | 9.728 | 1.797 | 10.09 | 9.009 | 1.878 | 0.044 |
| 2006 | 511600 | 0.775 | 0.059 | 0.093 | 9.098 | 6.126 | 8.267 | 1.981 | 9.15 | 8.68 | 1.212 | 0.037 |
| 2006 | 511700 | 0.739 | 0.075 | 0.136 | 9.728 | 6.471 | 9.751 | 1.423 | 7.399 | 8.682 | 1.191 | 0.031 |
| 2006 | 511800 | 0.77 | 0.129 | 1.017 | 11.03 | 5.028 | 9.778 | 2.076 | 5.926 | 9.175 | 1.936 | 0.038 |
| 2006 | 511900 | 0.744 | 0.093 | 0 | 9.8 | 5.915 | 5.442 | 2.107 | 9.595 | 8.39 | 0.668 | 0.019 |
| 2006 | 512000 | 0.746 | 0.171 | 0 | 9.031 | 6.193 | 6.866 | 2.108 | 7.812 | 8.698 | 1.209 | 0.027 |
| 2006 | 520100 | 0.757 | 1.209 | 1.059 | 11.19 | 5.871 | 12.28 | 1.488 | 6.928 | 9.664 | 1.379 | 0.109 |
| 2006 | 520200 | 0.728 | 0.071 | 0.184 | 9.645 | 5.705 | 8.589 | 1.701 | 10.93 | 8.645 | 2.278 | 0.098 |
| 2006 | 520300 | 0.756 | 0.181 | 0.113 | 9.809 | 6.596 | 9.735 | 1.737 | 7.597 | 8.707 | 1.382 | 0.06 |
| 2006 | 520400 | 0.753 | 0.143 | 0.176 | 10.1 | 5.564 | 8.974 | 1.416 | 7.145 | 8.382 | 1.507 | 0.084 |
| 2006 | 530100 | 0.759 | 1.113 | 1.019 | 11.35 | 6.243 | 12.24 | 1.772 | 6.576 | 10.04 | 1.151 | 0.091 |
| 2006 | 530300 | 0.739 | 0.082 | 0.093 | 9.486 | 6.388 | 9.338 | 2.198 | 7.119 | 8.878 | 2.08 | 0.092 |
| 2006 | 530400 | 0.761 | 0.228 | 0.225 | 11.01 | 5.35 | 9.151 | 2.768 | 9.686 | 9.892 | 3.239 | 0.082 |
| 2006 | 530500 | 0.734 | 0.116 | 0.125 | 10.29 | 5.498 | 8.442 | 2.481 | 8.05 | 8.548 | 0.546 | 0.069 |
| 2006 | 530600 | 0.715 | 0.046 | 0.074 | 8.745 | 6.285 | 8.194 | 1.206 | 7.119 | 8.113 | 0.831 | 0.061 |
| 2006 | 530700 | 0.77 | 0.174 | 0.273 | 11.16 | 4.749 | 9.259 | 2.905 | 9.56 | 8.562 | 0.779 | 0.087 |
| 2006 | 530800 | 0.752 | 0.154 | 0.104 | 10.57 | 5.484 | 8.268 | 1.426 | 9.68 | 8.379 | 0.879 | 0.073 |
| 2006 | 530900 | 0.759 | 0.049 | 0.093 | 10.64 | 5.396 | 8.147 | 2.127 | 9.56 | 8.413 | 1.186 | 0.055 |
| 2007 | 310000 | 0.767 | 2.81 | 0.88 | 15.85 | 7.229 | 13.09 | 1.504 | 15.61 | 11.26 | 1.262 | 0.184 |
| 2007 | 320100 | 0.765 | 1.902 | 1.563 | 13.88 | 6.425 | 13.43 | 2.84 | 14.22 | 10.66 | 1.424 | 0.115 |
| 2007 | 320200 | 0.751 | 2.289 | 0.655 | 14.12 | 6.135 | 11.54 | 3.071 | 14.56 | 11.13 | 1.842 | 0.088 |
| 2007 | 320300 | 0.772 | 0.831 | 0.492 | 12.22 | 6.847 | 11.64 | 2.557 | 12.73 | 9.668 | 1.707 | 0.062 |
| 2007 | 320400 | 0.761 | 1.969 | 0.789 | 14.05 | 5.879 | 11.69 | 2.91 | 14.15 | 10.63 | 1.99 | 0.097 |
| 2007 | 320500 | 0.742 | 2.583 | 0.828 | 14.66 | 6.437 | 11.93 | 3.311 | 15.51 | 11.23 | 2.373 | 0.106 |
| 2007 | 320600 | 0.756 | 1.775 | 0.44 | 13.23 | 6.641 | 11.2 | 2.741 | 14.68 | 9.914 | 1.86 | 0.071 |
| 2007 | 320700 | 0.758 | 0.485 | 0.316 | 12.47 | 6.178 | 10.33 | 3.006 | 13.24 | 9.394 | 1.619 | 0.079 |
| 2007 | 320800 | 0.77 | 0.568 | 0.475 | 12.37 | 6.28 | 11.07 | 2.631 | 12.29 | 9.459 | 1.961 | 0.068 |
| 2007 | 320900 | 0.764 | 0.712 | 0.286 | 12.53 | 6.697 | 10.74 | 2.862 | 13.33 | 9.6 | 1.769 | 0.05 |
| 2007 | 321000 | 0.769 | 1.413 | 0.625 | 13.14 | 6.13 | 11.24 | 2.821 | 13.41 | 10.03 | 1.744 | 0.074 |
| 2007 | 321100 | 0.756 | 2.007 | 0.847 | 13.39 | 5.594 | 11.28 | 2.807 | 13.6 | 10.55 | 1.934 | 0.07 |
| 2007 | 321200 | 0.763 | 1.253 | 0.327 | 12.9 | 6.216 | 10.45 | 2.971 | 13.25 | 9.81 | 1.852 | 0.081 |
| 2007 | 321300 | 0.757 | 0.177 | 0.082 | 11.97 | 6.276 | 9.641 | 3.245 | 10.93 | 9.041 | 1.776 | 0.066 |
| 2007 | 330100 | 0.713 | 2.41 | 1.122 | 14.41 | 6.511 | 12.81 | 2.682 | 14.57 | 10.89 | 1.569 | 0.106 |
| 2007 | 330200 | 0.728 | 2.676 | 0.684 | 14.81 | 6.336 | 11.74 | 2.463 | 14.46 | 10.88 | 1.89 | 0.105 |
| 2007 | 330300 | 0.733 | 1.983 | 0.407 | 13.21 | 6.639 | 11.07 | 2.474 | 13.06 | 10.21 | 1.907 | 0.074 |
| 2007 | 330400 | 0.697 | 1.999 | 0.325 | 14.02 | 5.82 | 10.31 | 2.799 | 14.05 | 10.62 | 2.288 | 0.071 |
| 2007 | 330500 | 0.715 | 1.91 | 0.353 | 13.71 | 5.552 | 9.957 | 3.033 | 13.37 | 10.36 | 1.848 | 0.073 |
| 2007 | 330600 | 0.72 | 2.378 | 0.4 | 13.85 | 6.078 | 10.69 | 2.805 | 13.64 | 10.63 | 2.189 | 0.065 |
| 2007 | 330700 | 0.741 | 2.391 | 0.532 | 13.76 | 6.129 | 11.02 | 3.154 | 12.86 | 10.28 | 1.763 | 0.074 |
| 2007 | 330800 | 0.756 | 0.871 | 0.242 | 13.21 | 5.512 | 9.172 | 3.086 | 10.34 | 9.596 | 1.653 | 0.073 |
| 2007 | 330900 | 0.754 | 0.992 | 0.595 | 14.24 | 4.572 | 9.943 | 2.213 | 10.95 | 10.29 | 1.236 | 0.115 |
| 2007 | 331000 | 0.738 | 2.081 | 0.232 | 13.25 | 6.345 | 10.1 | 2.931 | 12.38 | 10.27 | 2.255 | 0.064 |
| 2007 | 331100 | 0.762 | 1.167 | 0.377 | 13.38 | 5.537 | 10.41 | 2.355 | 9.739 | 9.553 | 1.468 | 0.085 |
| 2007 | 340100 | 0.739 | 1.169 | 1.391 | 13.07 | 6.171 | 12.6 | 2.874 | 13.55 | 9.767 | 1.42 | 0.123 |
| 2007 | 340200 | 0.772 | 1.277 | 1.066 | 13.61 | 5.44 | 11.63 | 2.801 | 12.77 | 9.969 | 1.929 | 0.092 |
| 2007 | 340300 | 0.758 | 0.434 | 0.585 | 11.5 | 5.873 | 10.68 | 2.442 | 11.94 | 9.256 | 1.37 | 0.066 |
| 2007 | 340400 | 0.71 | 0.369 | 0.779 | 12.06 | 5.478 | 10.86 | 2.229 | 11.75 | 9.5 | 1.542 | 0.082 |
| 2007 | 340500 | 0.773 | 0.878 | 0.829 | 13.61 | 4.847 | 10.3 | 2.709 | 12.44 | 10.15 | 2.835 | 0.126 |
| 2007 | 340600 | 0.741 | 0.244 | 0.562 | 11.59 | 5.364 | 10.21 | 2.241 | 10.78 | 9.273 | 1.837 | 0.071 |
| 2007 | 340700 | 0.748 | 0.668 | 0.904 | 13.35 | 4.299 | 10.05 | 2.405 | 11.38 | 10.08 | 1.919 | 0.103 |
| 2007 | 340800 | 0.748 | 0.208 | 0.218 | 10.93 | 6.415 | 10.05 | 2.429 | 11.59 | 9.062 | 1.663 | 0.061 |
| 2007 | 341000 | 0.771 | 0.336 | 0.343 | 12.58 | 4.997 | 9.494 | 2.809 | 11.27 | 9.377 | 0.914 | 0.073 |
| 2007 | 341100 | 0.734 | 0.232 | 0.367 | 11.53 | 6.096 | 10.34 | 2.729 | 11.9 | 9.351 | 1.427 | 0.045 |
| 2007 | 341200 | 0.753 | 0.142 | 0.144 | 10.33 | 6.882 | 9.825 | 2.497 | 10.81 | 8.357 | 0.937 | 0.048 |
| 2007 | 341300 | 0.751 | 0.149 | 0.146 | 10.71 | 6.425 | 9.912 | 2.717 | 10.64 | 8.71 | 0.787 | 0.033 |
| 2007 | 341500 | 0.735 | 0.159 | 0.182 | 10.61 | 6.545 | 10.12 | 2.01 | 10.69 | 8.535 | 1.174 | 0.059 |
| 2007 | 341600 | 0.745 | 0.116 | 0.092 | 10.08 | 6.357 | 8.711 | 3.075 | 10.75 | 8.658 | 0.866 | 0.033 |
| 2007 | 341700 | 0.765 | 0.152 | 0.407 | 11.38 | 5.063 | 8.952 | 2.892 | 11.03 | 8.979 | 1.277 | 0.1 |
| 2007 | 341800 | 0.773 | 0.277 | 0.092 | 11.93 | 5.617 | 8.012 | 2.39 | 11.31 | 9.417 | 1.267 | 0.065 |
| 2007 | 360100 | 0.73 | 0.918 | 1.609 | 12.93 | 6.197 | 13.08 | 2.079 | 13.75 | 10.04 | 1.577 | 0.08 |
| 2007 | 360200 | 0.745 | 0.491 | 0.573 | 11.98 | 5.051 | 10.56 | 2.083 | 10.99 | 9.615 | 1.775 | 0.051 |
| 2007 | 360300 | 0.734 | 0.484 | 0.192 | 11.72 | 5.218 | 9.049 | 2.306 | 11.2 | 9.475 | 2.394 | 0.075 |
| 2007 | 360400 | 0.751 | 0.251 | 0.693 | 11.4 | 6.173 | 11.32 | 2.555 | 12.66 | 9.279 | 1.755 | 0.062 |
| 2007 | 360500 | 0.741 | 0.44 | 0.268 | 12.41 | 4.735 | 9.338 | 2.778 | 12.33 | 9.656 | 1.936 | 0.103 |
| 2007 | 360600 | 0.743 | 0.672 | 0.182 | 12.27 | 4.744 | 8.38 | 2.376 | 11.02 | 9.399 | 1.57 | 0.092 |
| 2007 | 360700 | 0.765 | 0.147 | 0.428 | 11.03 | 6.777 | 11.22 | 2.466 | 13.25 | 8.842 | 1.042 | 0.067 |
| 2007 | 360800 | 0.745 | 0.14 | 0.209 | 11.21 | 6.173 | 9.846 | 2.23 | 12.17 | 8.878 | 1.546 | 0.073 |
| 2007 | 360900 | 0.735 | 0.287 | 0.299 | 10.96 | 6.29 | 10.1 | 2.524 | 12.22 | 8.937 | 1.512 | 0.068 |
| 2007 | 361000 | 0.731 | 0.251 | 0.358 | 11.53 | 5.954 | 10.38 | 2.614 | 11.62 | 8.864 | 1.801 | 0.071 |
| 2007 | 361100 | 0.729 | 0.157 | 0.135 | 11.22 | 6.557 | 9.785 | 2.756 | 12.19 | 8.777 | 1.224 | 0.075 |
| 2007 | 420100 | 0.746 | 1.725 | 1.365 | 13.68 | 6.719 | 13.56 | 2.227 | 14.35 | 10.48 | 1.096 | 0.079 |
| 2007 | 420200 | 0.745 | 0.524 | 0.477 | 10.46 | 5.543 | 10.33 | 2.527 | 12.21 | 9.809 | 1.629 | 0.044 |
| 2007 | 420300 | 0.745 | 0.477 | 0.342 | 11.84 | 5.855 | 10.22 | 2.66 | 10.35 | 9.338 | 1.771 | 0.039 |
| 2007 | 420500 | 0.783 | 0.702 | 0.576 | 12.98 | 5.995 | 10.73 | 2.775 | 11.58 | 9.891 | 2.087 | 0.044 |
| 2007 | 420600 | 0.75 | 0.177 | 0.24 | 12.01 | 6.366 | 10.06 | 2.589 | 11.31 | 9.581 | 1.727 | 0.029 |
| 2007 | 420700 | 0.745 | 0.498 | 0.369 | 12.2 | 4.673 | 9.141 | 2.723 | 11.03 | 9.907 | 1.779 | 0.041 |
| 2007 | 420800 | 0.751 | 0.463 | 0.359 | 11.86 | 5.699 | 9.931 | 2.8 | 11.27 | 9.805 | 1.57 | 0.023 |
| 2007 | 420900 | 0.755 | 0.22 | 0.305 | 11.67 | 6.248 | 10.25 | 2.924 | 11.36 | 9.334 | 1.517 | 0.03 |
| 2007 | 421000 | 0.757 | 0.302 | 0.662 | 9.898 | 6.48 | 11.63 | 2.395 | 11.24 | 9.229 | 1.599 | 0.052 |
| 2007 | 421100 | 0.749 | 0.166 | 0.235 | 11.15 | 6.594 | 10.26 | 3.212 | 11.4 | 9.093 | 1.794 | 0.029 |
| 2007 | 421200 | 0.754 | 0.219 | 0.445 | 11.96 | 5.656 | 10.25 | 2.589 | 10.75 | 9.232 | 1.819 | 0.039 |
| 2007 | 421300 | 0.744 | 0.291 | 0.156 | 11.67 | 5.538 | 8.995 | 2.461 | 10.1 | 9.254 | 1.909 | 0.021 |
| 2007 | 430100 | 0.741 | 1.533 | 1.413 | 13.98 | 6.457 | 13.03 | 2.635 | 13.95 | 10.07 | 1 | 0.11 |
| 2007 | 430200 | 0.745 | 0.955 | 0.777 | 12.6 | 5.941 | 10.92 | 2.487 | 12.11 | 9.763 | 1.479 | 0.059 |
| 2007 | 430300 | 0.734 | 0.913 | 1.077 | 12.94 | 5.679 | 11.66 | 2.575 | 12.06 | 9.687 | 1.022 | 0.054 |
| 2007 | 430400 | 0.76 | 0.293 | 0.467 | 11.2 | 6.592 | 10.55 | 2.419 | 12.17 | 9.213 | 0.986 | 0.042 |
| 2007 | 430500 | 0.727 | 0.299 | 0.198 | 10.79 | 6.62 | 9.944 | 2.218 | 10.91 | 8.74 | 0.817 | 0.041 |
| 2007 | 430600 | 0.75 | 0.406 | 0.475 | 12.23 | 6.297 | 10.64 | 2.377 | 11.25 | 9.661 | 1.379 | 0.043 |
| 2007 | 430700 | 0.76 | 0.345 | 0.314 | 11.23 | 6.417 | 10.16 | 2.635 | 11.88 | 9.486 | 1.301 | 0.047 |
| 2007 | 430800 | 0.767 | 0.195 | 0.347 | 11.27 | 5.09 | 9.624 | 2.427 | 9.582 | 9.043 | 0.511 | 0.058 |
| 2007 | 430900 | 0.741 | 0.251 | 0.282 | 11.84 | 6.143 | 10.04 | 2.335 | 10.73 | 9.039 | 0.718 | 0.039 |
| 2007 | 431000 | 0.776 | 0.328 | 0.226 | 12.4 | 6.145 | 9.936 | 2.25 | 12.44 | 9.345 | 1.076 | 0.063 |
| 2007 | 431100 | 0.741 | 0.223 | 0.252 | 11.27 | 6.363 | 9.985 | 2.46 | 12.25 | 8.975 | 0.727 | 0.039 |
| 2007 | 431200 | 0.739 | 0.187 | 0.233 | 11.07 | 6.219 | 10.22 | 2.121 | 9.912 | 9.017 | 0.664 | 0.046 |
| 2007 | 431300 | 0.761 | 0.481 | 0.357 | 10.3 | 6.031 | 10.16 | 2.425 | 10.79 | 9.077 | 1.255 | 0.049 |
| 2007 | 500000 | 0.759 | 0.886 | 0.607 | 12.74 | 8.082 | 12.93 | 2.215 | 13.74 | 9.508 | 1.234 | 0.113 |
| 2007 | 510100 | 0.753 | 1.891 | 1.197 | 13.23 | 7.014 | 13.2 | 2.522 | 13.62 | 10.34 | 1.114 | 0.089 |
| 2007 | 510300 | 0.754 | 0.28 | 0.471 | 11.83 | 5.776 | 10.34 | 2.082 | 9.869 | 9.298 | 1.251 | 0.04 |
| 2007 | 510400 | 0.755 | 0.693 | 0.596 | 13.13 | 4.701 | 9.722 | 2.152 | 9.869 | 10.19 | 3.347 | 0.092 |
| 2007 | 510500 | 0.76 | 0.162 | 0.401 | 10.7 | 6.193 | 10.41 | 2.301 | 10.11 | 8.977 | 1.22 | 0.053 |
| 2007 | 510600 | 0.758 | 0.571 | 0.531 | 12.23 | 5.954 | 10.57 | 2.321 | 9.104 | 9.679 | 1.548 | 0.049 |
| 2007 | 510700 | 0.752 | 0.595 | 0.627 | 12.73 | 6.288 | 11.22 | 2.465 | 11.32 | 9.467 | 1.119 | 0.042 |
| 2007 | 510800 | 0.746 | 0.186 | 0.119 | 11.33 | 5.728 | 8.854 | 2.312 | 7.104 | 8.675 | 0.884 | 0.039 |
| 2007 | 510900 | 0.747 | 0.17 | 0.126 | 11.15 | 5.949 | 9.068 | 2.476 | 9.406 | 8.862 | 1.028 | 0.031 |
| 2007 | 511000 | 0.757 | 0.185 | 0.246 | 10.71 | 6.049 | 9.705 | 1.785 | 10.56 | 8.98 | 1.563 | 0.034 |
| 2007 | 511100 | 0.75 | 0.382 | 0.207 | 11.86 | 5.865 | 9.369 | 2.56 | 8.626 | 9.245 | 1.839 | 0.064 |
| 2007 | 511300 | 0.758 | 0.184 | 0.489 | 10.94 | 6.609 | 11.07 | 2.509 | 10.67 | 8.642 | 0.916 | 0.036 |
| 2007 | 511400 | 0.768 | 0.276 | 0.213 | 11.01 | 5.854 | 9.075 | 2.564 | 7.462 | 9.076 | 1.536 | 0.041 |
| 2007 | 511500 | 0.75 | 0.388 | 0.231 | 12.02 | 6.267 | 9.818 | 1.969 | 10.04 | 9.134 | 1.919 | 0.051 |
| 2007 | 511600 | 0.769 | 0.099 | 0.1 | 10.19 | 6.137 | 8.311 | 2.032 | 9.247 | 8.817 | 1.209 | 0.039 |
| 2007 | 511700 | 0.73 | 0.126 | 0.136 | 11.15 | 6.477 | 9.955 | 1.562 | 6.97 | 8.819 | 1.188 | 0.037 |
| 2007 | 511800 | 0.764 | 0.21 | 1.099 | 12.19 | 5.035 | 10.69 | 2.104 | 6.233 | 9.282 | 2.02 | 0.045 |
| 2007 | 511900 | 0.736 | 0.106 | 0 | 10.79 | 5.925 | 5.442 | 2.167 | 11.3 | 8.515 | 0.683 | 0.02 |
| 2007 | 512000 | 0.738 | 0.339 | 0 | 11.31 | 6.201 | 6.866 | 2.132 | 9.199 | 8.835 | 1.206 | 0.044 |
| 2007 | 520100 | 0.751 | 1.404 | 1.093 | 13.33 | 5.886 | 12.25 | 1.658 | 9.901 | 9.771 | 1.438 | 0.117 |
| 2007 | 520200 | 0.724 | 0.113 | 0.169 | 11.53 | 5.733 | 8.75 | 2.185 | 11.03 | 8.752 | 2.376 | 0.108 |
| 2007 | 520300 | 0.754 | 0.272 | 0.226 | 11.27 | 6.606 | 10.41 | 1.705 | 8.976 | 8.82 | 1.446 | 0.067 |
| 2007 | 520400 | 0.734 | 0.218 | 0.199 | 11.54 | 5.589 | 8.959 | 1.649 | 7.493 | 8.49 | 1.572 | 0.091 |
| 2007 | 530100 | 0.751 | 1.261 | 1.117 | 13.03 | 6.249 | 12.31 | 1.934 | 6.528 | 10.16 | 1.205 | 0.103 |
| 2007 | 530300 | 0.726 | 0.119 | 0.127 | 11.38 | 6.402 | 9.455 | 2.298 | 7.342 | 8.992 | 2.178 | 0.104 |
| 2007 | 530400 | 0.746 | 0.457 | 0.297 | 12.35 | 5.358 | 9.192 | 2.837 | 9.64 | 10.01 | 3.391 | 0.086 |
| 2007 | 530500 | 0.724 | 0.086 | 0.16 | 11.39 | 5.507 | 8.582 | 2.514 | 8.501 | 8.662 | 0.572 | 0.077 |
| 2007 | 530600 | 0.707 | 0.031 | 0.06 | 10.83 | 6.303 | 8.325 | 1.535 | 7.327 | 8.226 | 0.87 | 0.068 |
| 2007 | 530700 | 0.741 | 0.22 | 0.295 | 12.35 | 4.771 | 9.388 | 2.874 | 9.563 | 8.675 | 0.816 | 0.11 |
| 2007 | 530800 | 0.744 | 0.144 | 0.125 | 11.58 | 5.5 | 8.546 | 1.463 | 9.727 | 8.492 | 0.92 | 0.091 |
| 2007 | 530900 | 0.755 | 0.065 | 0.088 | 11.5 | 5.406 | 8.496 | 2.205 | 9.563 | 8.526 | 1.241 | 0.061 |
| 2008 | 310000 | 0.764 | 2.88 | 0.883 | 15.97 | 7.238 | 13.13 | 1.533 | 15.76 | 11.37 | 1.321 | 0.19 |
| 2008 | 320100 | 0.759 | 2.112 | 1.645 | 14.23 | 6.437 | 13.49 | 2.913 | 14.31 | 10.78 | 1.49 | 0.119 |
| 2008 | 320200 | 0.748 | 2.399 | 0.668 | 14.37 | 6.14 | 11.6 | 3.09 | 14.6 | 11.24 | 1.929 | 0.094 |
| 2008 | 320300 | 0.767 | 1.023 | 0.508 | 12.55 | 6.853 | 11.7 | 2.631 | 12.91 | 9.782 | 1.787 | 0.069 |
| 2008 | 320400 | 0.76 | 2.039 | 0.831 | 14.24 | 5.883 | 11.72 | 3.081 | 14.16 | 10.74 | 2.083 | 0.1 |
| 2008 | 320500 | 0.748 | 3.309 | 0.79 | 15.1 | 6.445 | 12.02 | 3.33 | 15.55 | 11.36 | 2.398 | 0.116 |
| 2008 | 320600 | 0.754 | 1.852 | 0.451 | 13.41 | 6.638 | 11.33 | 2.835 | 14.53 | 10.03 | 1.948 | 0.078 |
| 2008 | 320700 | 0.753 | 0.539 | 0.303 | 13.03 | 6.191 | 10.23 | 3.073 | 13.38 | 9.518 | 1.636 | 0.094 |
| 2008 | 320800 | 0.759 | 0.621 | 0.457 | 12.96 | 6.286 | 11.15 | 2.686 | 12.66 | 9.583 | 1.982 | 0.084 |
| 2008 | 320900 | 0.76 | 0.916 | 0.353 | 12.58 | 6.699 | 10.93 | 2.927 | 13.39 | 9.724 | 1.788 | 0.061 |
| 2008 | 321000 | 0.763 | 1.644 | 0.631 | 13.41 | 6.131 | 11.27 | 2.91 | 13.99 | 10.14 | 1.826 | 0.08 |
| 2008 | 321100 | 0.752 | 2.31 | 0.896 | 13.73 | 5.594 | 11.33 | 2.919 | 13.63 | 10.68 | 1.954 | 0.067 |
| 2008 | 321200 | 0.758 | 1.357 | 0.368 | 13.2 | 6.216 | 10.59 | 3.038 | 13.5 | 9.923 | 1.939 | 0.086 |
| 2008 | 321300 | 0.752 | 0.323 | 0.102 | 12.46 | 6.281 | 9.665 | 3.328 | 11.09 | 9.154 | 1.859 | 0.083 |
| 2008 | 330100 | 0.71 | 2.692 | 1.121 | 14.82 | 6.519 | 12.85 | 2.676 | 14.65 | 11.01 | 1.585 | 0.112 |
| 2008 | 330200 | 0.729 | 2.875 | 0.701 | 14.82 | 6.342 | 11.78 | 2.516 | 14.38 | 11.01 | 1.91 | 0.112 |
| 2008 | 330300 | 0.739 | 2.011 | 0.438 | 13.32 | 6.649 | 11.17 | 2.556 | 12.11 | 10.33 | 1.927 | 0.077 |
| 2008 | 330400 | 0.707 | 2.291 | 0.351 | 14.25 | 5.823 | 10.51 | 2.889 | 13.76 | 10.75 | 2.312 | 0.077 |
| 2008 | 330500 | 0.718 | 2.26 | 0.364 | 13.9 | 5.555 | 10.06 | 3.052 | 13.23 | 10.48 | 1.867 | 0.077 |
| 2008 | 330600 | 0.716 | 3.111 | 0.419 | 14.16 | 6.08 | 10.82 | 2.806 | 13.28 | 10.76 | 2.211 | 0.069 |
| 2008 | 330700 | 0.746 | 2.44 | 0.559 | 13.98 | 6.134 | 11.15 | 3.169 | 12.78 | 10.41 | 1.77 | 0.078 |
| 2008 | 330800 | 0.751 | 1.161 | 0.22 | 13.45 | 5.517 | 9.231 | 3.123 | 10.61 | 9.726 | 1.66 | 0.077 |
| 2008 | 330900 | 0.744 | 1.205 | 0.601 | 14.42 | 4.572 | 10.03 | 2.328 | 11.61 | 10.42 | 1.242 | 0.128 |
| 2008 | 331000 | 0.733 | 2.182 | 0.236 | 13.47 | 6.353 | 10.21 | 2.959 | 12.02 | 10.39 | 2.279 | 0.067 |
| 2008 | 331100 | 0.758 | 1.269 | 0.381 | 13.54 | 5.543 | 10.48 | 2.413 | 10.94 | 9.677 | 1.483 | 0.086 |
| 2008 | 340100 | 0.733 | 1.31 | 1.403 | 13.8 | 6.188 | 12.69 | 2.797 | 13.63 | 9.897 | 1.426 | 0.169 |
| 2008 | 340200 | 0.765 | 1.636 | 1.274 | 14.17 | 5.442 | 11.69 | 2.801 | 12.89 | 10.1 | 1.919 | 0.101 |
| 2008 | 340300 | 0.756 | 0.442 | 0.631 | 13.23 | 5.881 | 10.69 | 2.526 | 11.99 | 9.387 | 1.363 | 0.071 |
| 2008 | 340400 | 0.711 | 0.584 | 0.789 | 12.52 | 5.484 | 10.94 | 2.293 | 11.19 | 9.63 | 1.549 | 0.093 |
| 2008 | 340500 | 0.767 | 1.061 | 0.924 | 13.72 | 4.853 | 10.64 | 2.728 | 12.58 | 10.28 | 2.82 | 0.118 |
| 2008 | 340600 | 0.732 | 0.365 | 0.575 | 11.91 | 5.374 | 10.28 | 2.316 | 11.04 | 9.403 | 1.845 | 0.075 |
| 2008 | 340700 | 0.748 | 0.867 | 0.892 | 13.59 | 4.303 | 10.1 | 2.433 | 11.85 | 10.21 | 1.909 | 0.12 |
| 2008 | 340800 | 0.748 | 0.176 | 0.239 | 11.99 | 6.42 | 10.22 | 2.523 | 11.71 | 9.192 | 1.671 | 0.064 |
| 2008 | 341000 | 0.763 | 0.47 | 0.359 | 13.13 | 5 | 9.514 | 2.809 | 11.41 | 9.507 | 0.909 | 0.082 |
| 2008 | 341100 | 0.739 | 0.284 | 0.373 | 11.93 | 6.103 | 10.44 | 2.819 | 11.28 | 9.481 | 1.433 | 0.051 |
| 2008 | 341200 | 0.749 | 0.173 | 0.172 | 10.51 | 6.895 | 9.992 | 2.622 | 10.97 | 8.487 | 0.932 | 0.056 |
| 2008 | 341300 | 0.74 | 0.144 | 0.142 | 11.19 | 6.439 | 10.12 | 2.761 | 10.82 | 8.84 | 0.791 | 0.038 |
| 2008 | 341500 | 0.729 | 0.142 | 0.217 | 10.94 | 6.553 | 10.21 | 2.104 | 11.24 | 8.665 | 1.179 | 0.067 |
| 2008 | 341600 | 0.741 | 0.222 | 0.102 | 9.972 | 6.378 | 8.883 | 3.435 | 10.99 | 8.788 | 0.869 | 0.038 |
| 2008 | 341700 | 0.758 | 0.141 | 0.456 | 12.02 | 5.068 | 9.438 | 3.143 | 11.21 | 9.109 | 1.282 | 0.123 |
| 2008 | 341800 | 0.765 | 0.426 | 0.143 | 12.6 | 5.623 | 8.161 | 2.512 | 11.4 | 9.547 | 1.273 | 0.073 |
| 2008 | 360100 | 0.733 | 0.98 | 1.582 | 13.03 | 6.204 | 13.01 | 2.078 | 13.79 | 10.17 | 1.568 | 0.083 |
| 2008 | 360200 | 0.735 | 0.64 | 0.564 | 12.23 | 5.064 | 10.15 | 2.695 | 11.02 | 9.708 | 1.899 | 0.069 |
| 2008 | 360300 | 0.733 | 0.431 | 0.228 | 12.09 | 5.225 | 9.129 | 2.33 | 11.41 | 9.568 | 2.562 | 0.085 |
| 2008 | 360400 | 0.756 | 0.384 | 0.698 | 11.68 | 6.183 | 11.26 | 2.644 | 12.62 | 9.409 | 1.746 | 0.066 |
| 2008 | 360500 | 0.718 | 0.434 | 0.841 | 12.61 | 4.814 | 9.991 | 2.878 | 12.65 | 9.786 | 1.926 | 0.122 |
| 2008 | 360600 | 0.741 | 1.227 | 0.225 | 12.53 | 4.755 | 8.379 | 2.412 | 11.17 | 9.491 | 1.68 | 0.106 |
| 2008 | 360700 | 0.758 | 0.169 | 0.447 | 11.33 | 6.79 | 11.21 | 2.228 | 13.26 | 8.935 | 1.115 | 0.084 |
| 2008 | 360800 | 0.745 | 0.201 | 0.221 | 11.69 | 6.185 | 9.863 | 2.378 | 12.25 | 9.008 | 1.538 | 0.082 |
| 2008 | 360900 | 0.729 | 0.254 | 0.3 | 11.78 | 6.299 | 10.19 | 2.527 | 12.23 | 9.068 | 1.504 | 0.076 |
| 2008 | 361000 | 0.729 | 0.172 | 0.363 | 11.96 | 5.96 | 10.45 | 2.516 | 11.56 | 8.994 | 1.792 | 0.081 |
| 2008 | 361100 | 0.731 | 0.131 | 0.164 | 11.66 | 6.574 | 9.871 | 2.786 | 12.25 | 8.907 | 1.217 | 0.076 |
| 2008 | 420100 | 0.742 | 1.989 | 1.432 | 13.85 | 6.725 | 13.6 | 2.277 | 14.4 | 10.57 | 1.173 | 0.087 |
| 2008 | 420200 | 0.738 | 0.533 | 0.503 | 10.64 | 5.55 | 10.22 | 2.574 | 12.27 | 9.901 | 1.743 | 0.047 |
| 2008 | 420300 | 0.74 | 0.5 | 0.361 | 11.84 | 5.861 | 10.3 | 2.606 | 10.46 | 9.431 | 1.895 | 0.044 |
| 2008 | 420500 | 0.777 | 0.771 | 0.604 | 13.16 | 5.994 | 10.74 | 2.785 | 11.62 | 9.983 | 2.234 | 0.048 |
| 2008 | 420600 | 0.741 | 0.149 | 0.241 | 12.25 | 6.371 | 10.12 | 2.604 | 11.67 | 9.674 | 1.848 | 0.032 |
| 2008 | 420700 | 0.735 | 0.51 | 0.362 | 12.35 | 4.671 | 9.384 | 2.807 | 11.08 | 10 | 1.904 | 0.045 |
| 2008 | 420800 | 0.745 | 0.554 | 0.364 | 12.05 | 5.704 | 9.882 | 2.774 | 11.32 | 9.898 | 1.68 | 0.024 |
| 2008 | 420900 | 0.747 | 0.289 | 0.245 | 11.71 | 6.264 | 10.3 | 2.932 | 11.48 | 9.426 | 1.624 | 0.033 |
| 2008 | 421000 | 0.748 | 0.377 | 0.674 | 11.16 | 6.49 | 11.71 | 2.394 | 11.3 | 9.322 | 1.711 | 0.026 |
| 2008 | 421100 | 0.74 | 0.22 | 0.258 | 11.72 | 6.6 | 10.44 | 3.21 | 11.44 | 9.185 | 1.92 | 0.036 |
| 2008 | 421200 | 0.745 | 0.348 | 0.429 | 12.07 | 5.664 | 10.33 | 2.573 | 11.11 | 9.325 | 1.947 | 0.044 |
| 2008 | 421300 | 0.739 | 0.298 | 0.181 | 11.92 | 5.546 | 8.81 | 2.47 | 10.21 | 9.347 | 2.043 | 0.023 |
| 2008 | 430100 | 0.738 | 1.659 | 1.381 | 14.21 | 6.469 | 13.09 | 2.597 | 14.04 | 10.2 | 1.034 | 0.113 |
| 2008 | 430200 | 0.737 | 1.049 | 0.704 | 13.05 | 5.948 | 11.02 | 2.489 | 12.25 | 9.885 | 1.531 | 0.064 |
| 2008 | 430300 | 0.731 | 0.962 | 1.147 | 13.1 | 5.684 | 11.8 | 2.606 | 12.2 | 9.809 | 1.058 | 0.061 |
| 2008 | 430400 | 0.754 | 0.318 | 0.475 | 11.43 | 6.595 | 10.58 | 2.514 | 12.38 | 9.335 | 1.02 | 0.045 |
| 2008 | 430500 | 0.718 | 0.289 | 0.207 | 10.98 | 6.626 | 9.996 | 2.506 | 10.86 | 8.863 | 0.846 | 0.041 |
| 2008 | 430600 | 0.744 | 0.346 | 0.498 | 12.36 | 6.313 | 10.79 | 2.463 | 11.43 | 9.754 | 1.476 | 0.046 |
| 2008 | 430700 | 0.756 | 0.381 | 0.332 | 11.46 | 6.42 | 10.28 | 2.608 | 11.9 | 9.609 | 1.347 | 0.048 |
| 2008 | 430800 | 0.766 | 0.213 | 0.478 | 11.88 | 5.103 | 9.605 | 2.516 | 9.682 | 9.165 | 0.529 | 0.061 |
| 2008 | 430900 | 0.732 | 0.353 | 0.287 | 12.17 | 6.148 | 10.07 | 2.536 | 10.93 | 9.161 | 0.743 | 0.041 |
| 2008 | 431000 | 0.764 | 0.385 | 0.301 | 12.63 | 6.155 | 10.16 | 2.249 | 12.51 | 9.467 | 1.113 | 0.062 |
| 2008 | 431100 | 0.732 | 0.223 | 0.267 | 11.5 | 6.363 | 10.05 | 2.471 | 12.26 | 9.097 | 0.753 | 0.042 |
| 2008 | 431200 | 0.732 | 0.173 | 0.245 | 11.15 | 6.232 | 10.26 | 2.167 | 10.25 | 9.14 | 0.687 | 0.047 |
| 2008 | 431300 | 0.762 | 0.443 | 0.355 | 10.28 | 6.049 | 10.17 | 2.591 | 10.69 | 9.17 | 1.343 | 0.056 |
| 2008 | 500000 | 0.754 | 0.891 | 0.635 | 13.05 | 8.089 | 13.09 | 2.25 | 14.5 | 9.63 | 1.277 | 0.128 |
| 2008 | 510100 | 0.748 | 2.285 | 1.23 | 13.54 | 7.026 | 13.25 | 2.608 | 14.45 | 10.42 | 1.153 | 0.099 |
| 2008 | 510300 | 0.744 | 0.418 | 0.478 | 12.13 | 5.786 | 10.42 | 2.086 | 9.259 | 9.381 | 1.296 | 0.044 |
| 2008 | 510400 | 0.755 | 0.952 | 0.603 | 13.15 | 4.711 | 9.83 | 2.129 | 9.259 | 10.27 | 3.467 | 0.101 |
| 2008 | 510500 | 0.753 | 0.191 | 0.421 | 11.11 | 6.201 | 10.44 | 2.351 | 11.65 | 9.06 | 1.263 | 0.061 |
| 2008 | 510600 | 0.752 | 0.563 | 0.593 | 12.79 | 5.959 | 10.88 | 2.402 | 9.289 | 9.761 | 1.603 | 0.038 |
| 2008 | 510700 | 0.743 | 0.68 | 0.676 | 12.67 | 6.293 | 11.32 | 2.471 | 11.48 | 9.549 | 1.159 | 0.035 |
| 2008 | 510800 | 0.738 | 0.245 | 0.136 | 11.68 | 5.738 | 8.168 | 2.336 | 7.776 | 8.757 | 0.916 | 0.032 |
| 2008 | 510900 | 0.736 | 0.197 | 0.162 | 11.18 | 5.953 | 9.158 | 2.484 | 7.776 | 8.974 | 1.048 | 0.034 |
| 2008 | 511000 | 0.749 | 0.276 | 0.232 | 11.09 | 6.052 | 9.877 | 1.792 | 9.21 | 9.102 | 1.617 | 0.037 |
| 2008 | 511100 | 0.745 | 0.448 | 0.203 | 12.02 | 5.868 | 9.386 | 2.548 | 10.1 | 9.367 | 1.903 | 0.071 |
| 2008 | 511300 | 0.753 | 0.271 | 0.379 | 11.27 | 6.619 | 11.08 | 2.511 | 10.85 | 8.764 | 0.948 | 0.04 |
| 2008 | 511400 | 0.771 | 0.345 | 0.21 | 11.54 | 5.848 | 9.077 | 2.559 | 8.641 | 9.158 | 1.591 | 0.045 |
| 2008 | 511500 | 0.745 | 0.551 | 0.233 | 11.95 | 6.274 | 9.923 | 2.144 | 9.998 | 9.256 | 1.986 | 0.062 |
| 2008 | 511600 | 0.766 | 0.092 | 0.09 | 10.6 | 6.145 | 8.249 | 2.077 | 9.416 | 8.9 | 1.252 | 0.041 |
| 2008 | 511700 | 0.734 | 0.137 | 0.187 | 11.32 | 6.486 | 9.822 | 1.589 | 6.592 | 8.932 | 1.211 | 0.042 |
| 2008 | 511800 | 0.76 | 0.225 | 1.098 | 12.6 | 5.04 | 10.63 | 2.099 | 5.87 | 9.395 | 2.059 | 0.044 |
| 2008 | 511900 | 0.729 | 0.073 | 0 | 11.19 | 5.988 | 5.442 | 2.178 | 11.59 | 8.598 | 0.707 | 0.022 |
| 2008 | 512000 | 0.729 | 0.247 | 0 | 11.61 | 6.209 | 6.866 | 2.241 | 9.284 | 8.918 | 1.249 | 0.045 |
| 2008 | 520100 | 0.747 | 1.344 | 1.054 | 13.52 | 5.897 | 12.3 | 1.658 | 9.988 | 9.884 | 1.466 | 0.123 |
| 2008 | 520200 | 0.723 | 0.089 | 0.169 | 11.83 | 5.746 | 9.05 | 2.358 | 11.08 | 8.865 | 2.422 | 0.132 |
| 2008 | 520300 | 0.76 | 0.356 | 0.237 | 11.7 | 6.612 | 10.53 | 1.683 | 10.55 | 8.932 | 1.474 | 0.069 |
| 2008 | 520400 | 0.731 | 0.276 | 0.245 | 11.72 | 5.6 | 9.052 | 1.52 | 10.42 | 8.602 | 1.602 | 0.108 |
| 2008 | 530100 | 0.748 | 1.276 | 0.155 | 13.38 | 6.27 | 9.238 | 2.485 | 6.438 | 10.27 | 1.229 | 0.122 |
| 2008 | 530300 | 0.729 | 0.139 | 0.126 | 11.6 | 6.41 | 9.668 | 2.347 | 7.463 | 9.104 | 2.22 | 0.113 |
| 2008 | 530400 | 0.744 | 0.516 | 0.226 | 12.26 | 5.361 | 9.543 | 2.837 | 10.15 | 10.13 | 3.516 | 0.1 |
| 2008 | 530500 | 0.718 | 0.118 | 0.173 | 11.38 | 5.514 | 8.706 | 2.514 | 8.779 | 8.774 | 0.583 | 0.082 |
| 2008 | 530600 | 0.703 | 0.011 | 0.061 | 11.01 | 6.309 | 8.507 | 2.008 | 7.455 | 8.339 | 0.887 | 0.081 |
| 2008 | 530700 | 0.741 | 0.176 | 0.393 | 12.47 | 4.783 | 9.575 | 2.874 | 8.779 | 8.788 | 0.831 | 0.129 |
| 2008 | 530800 | 0.738 | 0.096 | 0.153 | 11.71 | 5.553 | 8.805 | 1.587 | 9.376 | 8.618 | 0.954 | 0.101 |
| 2008 | 530900 | 0.751 | 0.048 | 0.105 | 11.24 | 5.415 | 9.231 | 2.133 | 8.335 | 8.639 | 1.265 | 0.067 |
| 2009 | 310000 | 0.762 | 3.185 | 0.911 | 16.55 | 7.245 | 13.15 | 1.5 | 15.79 | 11.5 | 1.37 | 0.187 |
| 2009 | 320100 | 0.751 | 2.391 | 1.68 | 14.6 | 6.445 | 13.56 | 2.94 | 14.31 | 10.9 | 1.545 | 0.119 |
| 2009 | 320200 | 0.748 | 2.963 | 0.664 | 14.96 | 6.143 | 11.63 | 3.118 | 14.6 | 11.37 | 2 | 0.095 |
| 2009 | 320300 | 0.762 | 1.378 | 0.501 | 12.87 | 6.864 | 11.74 | 2.638 | 13.07 | 9.907 | 1.853 | 0.08 |
| 2009 | 320400 | 0.761 | 2.619 | 0.78 | 14.29 | 5.886 | 11.74 | 3.089 | 14.38 | 10.87 | 2.16 | 0.104 |
| 2009 | 320500 | 0.75 | 4.097 | 0.853 | 15.33 | 6.451 | 12.14 | 3.317 | 15.54 | 11.46 | 2.482 | 0.115 |
| 2009 | 320600 | 0.75 | 2.712 | 0.492 | 13.75 | 6.637 | 11.4 | 2.812 | 14.13 | 10.15 | 2.019 | 0.087 |
| 2009 | 320700 | 0.749 | 1.029 | 0.307 | 13.18 | 6.196 | 10.28 | 3.083 | 13.47 | 9.628 | 1.694 | 0.113 |
| 2009 | 320800 | 0.755 | 1.021 | 0.49 | 13.2 | 6.281 | 11.28 | 2.7 | 12.95 | 9.709 | 2.055 | 0.1 |
| 2009 | 320900 | 0.754 | 1.192 | 0.334 | 13.22 | 6.7 | 10.93 | 2.918 | 13.48 | 9.849 | 1.853 | 0.075 |
| 2009 | 321000 | 0.757 | 1.849 | 0.65 | 13.7 | 6.129 | 11.24 | 2.943 | 14.25 | 10.27 | 1.893 | 0.087 |
| 2009 | 321100 | 0.749 | 2.781 | 0.924 | 14.13 | 5.598 | 11.37 | 2.982 | 13.8 | 10.79 | 2.022 | 0.07 |
| 2009 | 321200 | 0.752 | 1.851 | 0.389 | 13.51 | 6.223 | 10.69 | 3.055 | 13.49 | 10.05 | 2.01 | 0.104 |
| 2009 | 321300 | 0.746 | 0.375 | 0.115 | 13 | 6.293 | 9.911 | 3.319 | 11.24 | 9.28 | 1.927 | 0.099 |
| 2009 | 330100 | 0.712 | 3.12 | 1.105 | 14.99 | 6.527 | 12.88 | 2.698 | 14.82 | 11.12 | 1.64 | 0.117 |
| 2009 | 330200 | 0.725 | 3.296 | 0.695 | 14.9 | 6.347 | 11.81 | 2.641 | 14.23 | 11.12 | 1.976 | 0.114 |
| 2009 | 330300 | 0.74 | 2.263 | 0.441 | 13.5 | 6.658 | 11.22 | 2.632 | 11.98 | 10.44 | 1.98 | 0.077 |
| 2009 | 330400 | 0.714 | 2.748 | 0.368 | 14.37 | 5.828 | 10.57 | 2.754 | 13.72 | 10.86 | 2.393 | 0.079 |
| 2009 | 330500 | 0.722 | 2.901 | 0.373 | 14.04 | 5.557 | 10.1 | 3.139 | 13.22 | 10.59 | 1.919 | 0.078 |
| 2009 | 330600 | 0.717 | 3.508 | 0.422 | 14.39 | 6.082 | 10.88 | 2.773 | 13.23 | 10.87 | 2.272 | 0.071 |
| 2009 | 330700 | 0.74 | 2.731 | 0.543 | 14.11 | 6.139 | 11.23 | 3.163 | 12.52 | 10.51 | 1.819 | 0.078 |
| 2009 | 330800 | 0.749 | 1.634 | 0.229 | 13.68 | 5.521 | 9.229 | 3.224 | 10.68 | 9.834 | 1.705 | 0.078 |
| 2009 | 330900 | 0.734 | 1.256 | 0.603 | 14.59 | 4.572 | 10.02 | 2.44 | 11.19 | 10.52 | 1.276 | 0.132 |
| 2009 | 331000 | 0.731 | 2.686 | 0.246 | 13.58 | 6.36 | 10.28 | 3.115 | 11.76 | 10.5 | 2.358 | 0.066 |
| 2009 | 331100 | 0.757 | 1.444 | 0.369 | 13.62 | 5.551 | 10.45 | 2.543 | 9.898 | 9.787 | 1.535 | 0.082 |
| 2009 | 340100 | 0.718 | 1.743 | 1.319 | 14.4 | 6.197 | 12.77 | 2.838 | 13.28 | 10.01 | 1.491 | 0.168 |
| 2009 | 340200 | 0.756 | 2.429 | 1.229 | 14.96 | 5.439 | 11.73 | 2.924 | 13.02 | 10.2 | 2.021 | 0.113 |
| 2009 | 340300 | 0.756 | 0.729 | 0.653 | 13.4 | 5.888 | 10.86 | 2.552 | 12.01 | 9.489 | 1.436 | 0.072 |
| 2009 | 340400 | 0.71 | 0.82 | 0.766 | 12.87 | 5.491 | 11 | 2.303 | 11.32 | 9.739 | 1.619 | 0.102 |
| 2009 | 340500 | 0.76 | 1.316 | 0.979 | 14.18 | 4.857 | 10.59 | 2.741 | 12.88 | 10.38 | 2.97 | 0.15 |
| 2009 | 340600 | 0.722 | 0.462 | 0.591 | 12.64 | 5.383 | 10.3 | 2.316 | 11.43 | 9.512 | 1.929 | 0.079 |
| 2009 | 340700 | 0.744 | 1.298 | 0.909 | 13.96 | 4.304 | 10.11 | 2.425 | 12.09 | 10.31 | 2.01 | 0.146 |
| 2009 | 340800 | 0.745 | 0.304 | 0.243 | 12.52 | 6.423 | 10.25 | 2.693 | 11.63 | 9.301 | 1.746 | 0.062 |
| 2009 | 341000 | 0.755 | 0.646 | 0.373 | 13.39 | 5.001 | 9.556 | 2.851 | 11.5 | 9.61 | 0.957 | 0.09 |
| 2009 | 341100 | 0.736 | 0.632 | 0.378 | 12.02 | 6.11 | 10.53 | 3.012 | 11.06 | 9.59 | 1.498 | 0.059 |
| 2009 | 341200 | 0.742 | 0.171 | 0.173 | 10.6 | 6.908 | 10.29 | 2.678 | 10.41 | 8.59 | 0.982 | 0.065 |
| 2009 | 341300 | 0.728 | 0.154 | 0.136 | 11.28 | 6.454 | 9.903 | 2.845 | 11.23 | 8.949 | 0.826 | 0.04 |
| 2009 | 341500 | 0.726 | 0.175 | 0.255 | 12.34 | 6.559 | 10.33 | 1.927 | 11.27 | 8.773 | 1.212 | 0.075 |
| 2009 | 341600 | 0.738 | 0.256 | 0.102 | 10.32 | 6.392 | 9.084 | 3.461 | 11.28 | 8.896 | 0.893 | 0.045 |
| 2009 | 341700 | 0.752 | 0.238 | 0.52 | 12.77 | 5.074 | 9.861 | 3.174 | 11.31 | 9.218 | 1.34 | 0.145 |
| 2009 | 341800 | 0.752 | 0.751 | 0.089 | 12.91 | 5.627 | 8.317 | 2.97 | 11.52 | 9.656 | 1.331 | 0.082 |
| 2009 | 360100 | 0.73 | 1.173 | 1.741 | 13.4 | 6.209 | 13.09 | 2.129 | 13.89 | 10.28 | 1.652 | 0.083 |
| 2009 | 360200 | 0.729 | 0.699 | 0.613 | 12.5 | 5.077 | 10.19 | 2.693 | 11.14 | 9.82 | 1.947 | 0.08 |
| 2009 | 360300 | 0.73 | 0.499 | 0.23 | 12.11 | 5.231 | 9.148 | 2.327 | 11.46 | 9.68 | 2.625 | 0.088 |
| 2009 | 360400 | 0.751 | 0.394 | 0.669 | 11.72 | 6.197 | 11.31 | 2.732 | 12.78 | 9.512 | 1.839 | 0.076 |
| 2009 | 360500 | 0.748 | 0.67 | 0.353 | 12.84 | 4.825 | 9.385 | 3.009 | 12.59 | 9.898 | 1.974 | 0.136 |
| 2009 | 360600 | 0.737 | 0.873 | 0.21 | 12.69 | 4.786 | 8.365 | 2.425 | 11.23 | 9.604 | 1.722 | 0.103 |
| 2009 | 360700 | 0.749 | 0.219 | 0.465 | 11.75 | 6.799 | 11.25 | 2.266 | 13.31 | 9.047 | 1.143 | 0.09 |
| 2009 | 360800 | 0.74 | 0.198 | 0.2 | 11.82 | 6.193 | 9.833 | 2.472 | 12.37 | 9.111 | 1.62 | 0.088 |
| 2009 | 360900 | 0.728 | 0.416 | 0.304 | 11.93 | 6.31 | 10.33 | 2.609 | 12.31 | 9.17 | 1.584 | 0.088 |
| 2009 | 361000 | 0.727 | 0.28 | 0.401 | 12.1 | 5.989 | 10.59 | 2.914 | 11.44 | 9.107 | 1.837 | 0.097 |
| 2009 | 361100 | 0.742 | 0.16 | 0.209 | 11.68 | 6.591 | 9.912 | 2.788 | 12.38 | 9.009 | 1.282 | 0.087 |
| 2009 | 420100 | 0.741 | 2.172 | 1.418 | 14.01 | 6.728 | 13.65 | 2.402 | 14.51 | 10.69 | 1.203 | 0.088 |
| 2009 | 420200 | 0.728 | 0.96 | 0.507 | 10.79 | 5.555 | 10.38 | 2.768 | 12.37 | 10.01 | 1.782 | 0.045 |
| 2009 | 420300 | 0.729 | 0.78 | 0.388 | 12.18 | 5.867 | 10.39 | 2.64 | 10.58 | 9.543 | 1.943 | 0.05 |
| 2009 | 420500 | 0.748 | 0.936 | 0.602 | 13.25 | 5.995 | 10.8 | 2.85 | 11.73 | 10.1 | 2.289 | 0.052 |
| 2009 | 420600 | 0.728 | 0.263 | 0.299 | 12.4 | 6.378 | 10.51 | 2.661 | 12.05 | 9.782 | 1.89 | 0.035 |
| 2009 | 420700 | 0.722 | 0.807 | 0.56 | 12.44 | 4.675 | 9.141 | 2.868 | 11.18 | 10.11 | 1.946 | 0.049 |
| 2009 | 420800 | 0.734 | 0.499 | 0.259 | 12.29 | 5.707 | 9.819 | 2.793 | 11.46 | 10.01 | 1.718 | 0.026 |
| 2009 | 420900 | 0.736 | 0.4 | 0.257 | 12.08 | 6.27 | 10.36 | 2.944 | 11.58 | 9.539 | 1.664 | 0.037 |
| 2009 | 421000 | 0.736 | 0.5 | 0.691 | 11.23 | 6.495 | 11.67 | 2.376 | 11.4 | 9.434 | 1.754 | 0.027 |
| 2009 | 421100 | 0.728 | 0.338 | 0.284 | 12.21 | 6.606 | 10.55 | 3.188 | 11.56 | 9.293 | 1.963 | 0.039 |
| 2009 | 421200 | 0.736 | 0.562 | 0.393 | 12.56 | 5.672 | 10.14 | 2.625 | 11.25 | 9.437 | 1.996 | 0.049 |
| 2009 | 421300 | 0.736 | 0.366 | 0.205 | 12.08 | 5.552 | 8.801 | 2.45 | 10.31 | 9.455 | 2.088 | 0.023 |
| 2009 | 430100 | 0.729 | 1.873 | 1.369 | 14.32 | 6.479 | 13.13 | 2.648 | 14.14 | 10.31 | 1.031 | 0.119 |
| 2009 | 430200 | 0.729 | 1.36 | 0.719 | 13.47 | 5.948 | 11.21 | 2.643 | 12.42 | 10 | 1.525 | 0.079 |
| 2009 | 430300 | 0.733 | 1.244 | 1.135 | 13.35 | 5.688 | 11.6 | 2.712 | 12.39 | 9.925 | 1.054 | 0.066 |
| 2009 | 430400 | 0.743 | 0.322 | 0.461 | 11.59 | 6.606 | 11.4 | 2.657 | 12.42 | 9.451 | 1.017 | 0.049 |
| 2009 | 430500 | 0.709 | 0.435 | 0.212 | 11.35 | 6.639 | 10.09 | 2.532 | 10.78 | 8.979 | 0.843 | 0.042 |
| 2009 | 430600 | 0.734 | 0.51 | 0.528 | 12.55 | 6.326 | 10.49 | 2.474 | 11.74 | 9.862 | 1.509 | 0.041 |
| 2009 | 430700 | 0.746 | 0.416 | 0.335 | 11.85 | 6.424 | 10.45 | 2.576 | 11.99 | 9.717 | 1.377 | 0.049 |
| 2009 | 430800 | 0.761 | 0.336 | 0.361 | 11.65 | 5.108 | 9.464 | 2.622 | 9.894 | 9.273 | 0.541 | 0.068 |
| 2009 | 430900 | 0.722 | 0.395 | 0.372 | 11.5 | 6.154 | 10.22 | 2.442 | 11.05 | 9.277 | 0.741 | 0.04 |
| 2009 | 431000 | 0.758 | 0.494 | 0.217 | 12.91 | 6.161 | 9.91 | 2.287 | 12.67 | 9.583 | 1.11 | 0.069 |
| 2009 | 431100 | 0.722 | 0.247 | 0.26 | 11.68 | 6.374 | 10.1 | 2.601 | 12.34 | 9.213 | 0.75 | 0.044 |
| 2009 | 431200 | 0.722 | 0.191 | 0.258 | 11.24 | 6.232 | 10.3 | 2.164 | 10.45 | 9.248 | 0.702 | 0.052 |
| 2009 | 431300 | 0.748 | 0.499 | 0.313 | 11.72 | 6.042 | 10.1 | 2.528 | 11.03 | 9.278 | 1.373 | 0.056 |
| 2009 | 500000 | 0.75 | 1.143 | 0.653 | 13.07 | 8.094 | 13.17 | 2.28 | 14.87 | 9.746 | 1.273 | 0.131 |
| 2009 | 510100 | 0.743 | 2.647 | 1.242 | 13.61 | 7.038 | 13.29 | 2.711 | 14.82 | 10.54 | 1.145 | 0.095 |
| 2009 | 510300 | 0.73 | 0.462 | 0.5 | 12.13 | 5.795 | 10.43 | 2.096 | 7.406 | 9.483 | 1.353 | 0.04 |
| 2009 | 510400 | 0.752 | 1.071 | 0.588 | 13.25 | 4.715 | 9.913 | 2.226 | 7.406 | 10.39 | 3.443 | 0.097 |
| 2009 | 510500 | 0.741 | 0.248 | 0.412 | 11.49 | 6.209 | 10.61 | 2.322 | 11.71 | 9.179 | 1.255 | 0.069 |
| 2009 | 510600 | 0.73 | 0.847 | 0.616 | 12.54 | 5.962 | 10.86 | 2.471 | 9.498 | 9.881 | 1.592 | 0.042 |
| 2009 | 510700 | 0.723 | 0.795 | 0.747 | 12.45 | 6.3 | 11.55 | 2.5 | 11.33 | 9.669 | 1.151 | 0.04 |
| 2009 | 510800 | 0.718 | 0.191 | 0.058 | 12.24 | 5.745 | 8.275 | 2.386 | 8.013 | 8.877 | 0.909 | 0.046 |
| 2009 | 510900 | 0.725 | 0.32 | 0.167 | 11.57 | 5.959 | 9.173 | 2.559 | 8.903 | 9.077 | 1.094 | 0.039 |
| 2009 | 511000 | 0.736 | 0.29 | 0.293 | 11.11 | 6.054 | 9.916 | 1.797 | 8.844 | 9.218 | 1.612 | 0.038 |
| 2009 | 511100 | 0.734 | 0.534 | 0.253 | 12.4 | 5.867 | 9.508 | 2.553 | 8.473 | 9.483 | 1.897 | 0.074 |
| 2009 | 511300 | 0.745 | 0.232 | 0.339 | 11.44 | 6.625 | 11.17 | 2.497 | 10.95 | 8.883 | 0.942 | 0.044 |
| 2009 | 511400 | 0.758 | 0.415 | 0.265 | 11.5 | 5.852 | 9.55 | 2.53 | 8.839 | 9.278 | 1.58 | 0.05 |
| 2009 | 511500 | 0.736 | 0.519 | 0.232 | 12.36 | 6.282 | 9.966 | 2.129 | 8.845 | 9.376 | 1.972 | 0.066 |
| 2009 | 511600 | 0.76 | 0.099 | 0.082 | 10.73 | 6.153 | 8.335 | 2.307 | 9.515 | 9.02 | 1.243 | 0.043 |
| 2009 | 511700 | 0.734 | 0.175 | 0.207 | 11.48 | 6.489 | 10.05 | 1.763 | 7.648 | 9.034 | 1.264 | 0.043 |
| 2009 | 511800 | 0.753 | 0.229 | 1.226 | 12.33 | 5.044 | 10.81 | 2.089 | 6.08 | 9.497 | 2.149 | 0.051 |
| 2009 | 511900 | 0.715 | 0.128 | 0 | 11.18 | 5.994 | 5.442 | 2.261 | 11.24 | 8.717 | 0.702 | 0.024 |
| 2009 | 512000 | 0.718 | 0.295 | 0 | 11.8 | 6.217 | 6.866 | 2.234 | 9.406 | 9.021 | 1.304 | 0.044 |
| 2009 | 520100 | 0.741 | 1.515 | 1.1 | 13.57 | 5.906 | 12.41 | 1.677 | 9.831 | 9.986 | 1.53 | 0.13 |
| 2009 | 520200 | 0.721 | 0.081 | 0.185 | 12.02 | 5.766 | 8.905 | 2.341 | 11.25 | 8.967 | 2.528 | 0.147 |
| 2009 | 520300 | 0.756 | 0.363 | 0.265 | 11.97 | 6.621 | 10.46 | 1.677 | 8.477 | 9.035 | 1.539 | 0.073 |
| 2009 | 520400 | 0.729 | 0.361 | 0.233 | 11.88 | 5.611 | 9.252 | 1.595 | 9.925 | 8.705 | 1.672 | 0.098 |
| 2009 | 530100 | 0.744 | 1.481 | 1.149 | 13.59 | 6.28 | 12.51 | 2.046 | 6.421 | 10.37 | 1.282 | 0.125 |
| 2009 | 530300 | 0.727 | 0.131 | 0.137 | 11.85 | 6.424 | 9.842 | 2.405 | 7.625 | 9.21 | 2.266 | 0.113 |
| 2009 | 530400 | 0.74 | 0.729 | 0.228 | 12.95 | 5.368 | 9.321 | 2.825 | 10.14 | 10.24 | 3.589 | 0.096 |
| 2009 | 530500 | 0.71 | 0.148 | 0.171 | 11.81 | 5.522 | 9.24 | 2.536 | 9.445 | 8.877 | 0.609 | 0.093 |
| 2009 | 530600 | 0.697 | 0.05 | 0.065 | 11.37 | 6.33 | 8.622 | 2.078 | 7.625 | 8.441 | 0.9 | 0.088 |
| 2009 | 530700 | 0.735 | 0.581 | 0.428 | 12.55 | 4.792 | 9.675 | 3.046 | 5.14 | 8.89 | 0.868 | 0.14 |
| 2009 | 530800 | 0.729 | 0.117 | 0.154 | 12.02 | 5.52 | 8.94 | 1.633 | 7.668 | 8.724 | 0.974 | 0.109 |
| 2009 | 530900 | 0.745 | 0.053 | 0.131 | 11.21 | 5.479 | 8.584 | 2.246 | 5.14 | 8.741 | 1.321 | 0.071 |
| 2010 | 310000 | 0.759 | 3.554 | 0.941 | 16.48 | 7.253 | 13.15 | 1.396 | 15.83 | 11.6 | 1.398 | 0.191 |
| 2010 | 320100 | 0.747 | 2.686 | 1.708 | 14.78 | 6.45 | 13.58 | 2.963 | 14.46 | 11.01 | 1.577 | 0.125 |
| 2010 | 320200 | 0.738 | 3.953 | 0.657 | 15.22 | 6.145 | 11.6 | 3.158 | 14.62 | 11.48 | 2.041 | 0.104 |
| 2010 | 320300 | 0.756 | 1.75 | 0.5 | 13.05 | 6.88 | 11.7 | 2.789 | 13.44 | 10.01 | 1.891 | 0.095 |
| 2010 | 320400 | 0.759 | 3.266 | 0.752 | 14.7 | 5.888 | 11.72 | 3.13 | 14.41 | 10.98 | 2.204 | 0.121 |
| 2010 | 320500 | 0.745 | 4.149 | 0.843 | 15.54 | 6.458 | 12.09 | 3.319 | 15.57 | 11.57 | 2.533 | 0.123 |
| 2010 | 320600 | 0.747 | 3.424 | 0.495 | 14.09 | 6.637 | 11.32 | 2.94 | 14.15 | 10.26 | 2.061 | 0.111 |
| 2010 | 320700 | 0.744 | 1.254 | 0.307 | 13.66 | 6.21 | 10.45 | 3.11 | 13.52 | 9.734 | 1.728 | 0.154 |
| 2010 | 320800 | 0.748 | 1.171 | 0.473 | 13.63 | 6.289 | 11.14 | 2.84 | 13.48 | 9.815 | 2.097 | 0.128 |
| 2010 | 320900 | 0.749 | 1.37 | 0.346 | 13.51 | 6.705 | 10.95 | 2.955 | 13.69 | 9.956 | 1.892 | 0.099 |
| 2010 | 321000 | 0.751 | 2.148 | 0.641 | 14.21 | 6.129 | 11.26 | 3.035 | 14.37 | 10.37 | 1.932 | 0.1 |
| 2010 | 321100 | 0.745 | 3.121 | 0.932 | 14.48 | 5.601 | 11.37 | 3.035 | 13.9 | 10.89 | 2.064 | 0.084 |
| 2010 | 321200 | 0.746 | 2.212 | 0.415 | 13.67 | 6.224 | 10.76 | 3.101 | 13.73 | 10.16 | 2.051 | 0.113 |
| 2010 | 321300 | 0.741 | 0.593 | 0.118 | 13.28 | 6.303 | 9.679 | 3.288 | 11.71 | 9.386 | 1.967 | 0.123 |
| 2010 | 330100 | 0.71 | 3.624 | 1.033 | 15.25 | 6.535 | 12.98 | 2.662 | 14.9 | 11.23 | 1.646 | 0.134 |
| 2010 | 330200 | 0.726 | 3.792 | 0.695 | 15.17 | 6.353 | 11.86 | 2.671 | 14.27 | 11.23 | 1.983 | 0.125 |
| 2010 | 330300 | 0.74 | 2.634 | 0.589 | 13.65 | 6.668 | 11.22 | 2.772 | 11.69 | 10.55 | 1.987 | 0.08 |
| 2010 | 330400 | 0.715 | 3.251 | 0.443 | 14.61 | 5.834 | 10.87 | 2.862 | 13.9 | 10.97 | 2.401 | 0.088 |
| 2010 | 330500 | 0.72 | 3.3 | 0.374 | 14.16 | 5.561 | 10.12 | 3.114 | 13.34 | 10.7 | 1.926 | 0.085 |
| 2010 | 330600 | 0.716 | 3.338 | 0.431 | 14.59 | 6.084 | 10.89 | 2.84 | 13.38 | 10.97 | 2.281 | 0.076 |
| 2010 | 330700 | 0.738 | 3.088 | 0.555 | 14.3 | 6.146 | 11.25 | 3.173 | 12.38 | 10.61 | 1.887 | 0.083 |
| 2010 | 330800 | 0.745 | 1.866 | 0.217 | 13.9 | 5.526 | 9.231 | 3.242 | 10.65 | 9.942 | 1.712 | 0.086 |
| 2010 | 330900 | 0.732 | 1.539 | 0.574 | 14.74 | 4.572 | 10.01 | 2.453 | 10.73 | 10.63 | 1.28 | 0.147 |
| 2010 | 331000 | 0.73 | 2.897 | 0.245 | 13.8 | 6.368 | 10.3 | 3.155 | 11.4 | 10.59 | 2.371 | 0.071 |
| 2010 | 331100 | 0.754 | 1.858 | 0.366 | 13.76 | 5.559 | 10.48 | 2.538 | 10.14 | 9.893 | 1.567 | 0.087 |
| 2010 | 340100 | 0.71 | 2.08 | 1.325 | 15.1 | 6.204 | 12.83 | 2.86 | 13.78 | 10.11 | 1.547 | 0.206 |
| 2010 | 340200 | 0.744 | 2.817 | 1.245 | 15.29 | 5.436 | 11.81 | 3.082 | 13.13 | 10.3 | 2.097 | 0.133 |
| 2010 | 340300 | 0.75 | 1.236 | 0.652 | 13.77 | 5.892 | 10.89 | 2.614 | 12.13 | 9.602 | 1.452 | 0.085 |
| 2010 | 340400 | 0.704 | 1.279 | 0.824 | 13.16 | 5.497 | 11.05 | 2.309 | 11.11 | 9.839 | 1.68 | 0.122 |
| 2010 | 340500 | 0.755 | 1.893 | 1.036 | 14.45 | 4.861 | 10.66 | 2.837 | 13.08 | 10.5 | 2.883 | 0.145 |
| 2010 | 340600 | 0.713 | 0.683 | 0.617 | 12.84 | 5.392 | 10.32 | 2.328 | 11.78 | 9.612 | 2.001 | 0.091 |
| 2010 | 340700 | 0.74 | 2.208 | 0.952 | 14.3 | 4.304 | 10.16 | 2.439 | 12.28 | 10.43 | 1.952 | 0.135 |
| 2010 | 340800 | 0.74 | 0.456 | 0.259 | 12.91 | 6.423 | 10.5 | 2.756 | 11.98 | 9.401 | 1.812 | 0.065 |
| 2010 | 341000 | 0.749 | 1.043 | 0.373 | 13.76 | 4.998 | 9.623 | 2.903 | 11.68 | 9.732 | 0.929 | 0.115 |
| 2010 | 341100 | 0.732 | 1.136 | 0.424 | 12.59 | 6.111 | 10.59 | 3.191 | 11.28 | 9.689 | 1.554 | 0.07 |
| 2010 | 341200 | 0.733 | 0.341 | 0.178 | 10.97 | 6.92 | 10.2 | 2.743 | 11 | 8.712 | 0.953 | 0.078 |
| 2010 | 341300 | 0.717 | 0.271 | 0.171 | 11.54 | 6.465 | 9.996 | 2.894 | 11.42 | 9.049 | 0.857 | 0.05 |
| 2010 | 341500 | 0.723 | 0.447 | 0.292 | 11.7 | 6.558 | 10.44 | 2.02 | 11.44 | 8.873 | 1.257 | 0.084 |
| 2010 | 341600 | 0.734 | 0.394 | 0.13 | 10.99 | 6.398 | 9.182 | 3.621 | 11.63 | 8.996 | 0.927 | 0.053 |
| 2010 | 341700 | 0.745 | 0.637 | 0.621 | 13.36 | 5.078 | 10.11 | 3.167 | 11.6 | 9.318 | 1.39 | 0.163 |
| 2010 | 341800 | 0.75 | 1.396 | 0.097 | 13.63 | 5.629 | 8.516 | 2.982 | 11.87 | 9.755 | 1.38 | 0.1 |
| 2010 | 360100 | 0.728 | 1.366 | 1.65 | 13.47 | 6.219 | 13.1 | 2.14 | 14.13 | 10.37 | 1.7 | 0.092 |
| 2010 | 360200 | 0.726 | 0.969 | 0.636 | 12.8 | 5.095 | 10.21 | 2.776 | 11.32 | 9.972 | 1.818 | 0.11 |
| 2010 | 360300 | 0.724 | 0.574 | 0.23 | 13.02 | 5.237 | 9.102 | 2.773 | 11.55 | 9.792 | 2.621 | 0.114 |
| 2010 | 360400 | 0.745 | 0.468 | 0.678 | 11.96 | 6.21 | 11.31 | 3.049 | 13.02 | 9.603 | 1.892 | 0.093 |
| 2010 | 360500 | 0.741 | 0.813 | 0.449 | 13.72 | 4.771 | 9.474 | 3.062 | 12.79 | 10.05 | 1.844 | 0.176 |
| 2010 | 360600 | 0.725 | 0.93 | 0.198 | 12.46 | 4.803 | 8.351 | 2.933 | 11.3 | 9.715 | 1.719 | 0.145 |
| 2010 | 360700 | 0.739 | 0.336 | 0.454 | 11.61 | 6.81 | 11.27 | 2.317 | 13.25 | 9.159 | 1.141 | 0.092 |
| 2010 | 360800 | 0.734 | 0.418 | 0.204 | 11.96 | 6.205 | 9.807 | 2.776 | 12.6 | 9.201 | 1.667 | 0.11 |
| 2010 | 360900 | 0.726 | 0.552 | 0.303 | 12.21 | 6.324 | 10.43 | 2.624 | 12.41 | 9.322 | 1.479 | 0.106 |
| 2010 | 361000 | 0.725 | 0.382 | 0.372 | 12.26 | 6.001 | 10.73 | 2.895 | 11.54 | 9.259 | 1.715 | 0.13 |
| 2010 | 361100 | 0.736 | 0.326 | 0.152 | 11.96 | 6.607 | 9.922 | 3.024 | 12.74 | 9.1 | 1.319 | 0.114 |
| 2010 | 420100 | 0.738 | 2.499 | 1.427 | 14.15 | 6.73 | 13.69 | 2.431 | 14.62 | 10.8 | 1.201 | 0.094 |
| 2010 | 420200 | 0.717 | 1.177 | 0.531 | 12.91 | 5.561 | 10.62 | 2.829 | 12.32 | 10.12 | 1.78 | 0.051 |
| 2010 | 420300 | 0.718 | 1.055 | 0.502 | 12.54 | 5.867 | 10.7 | 2.641 | 10.66 | 9.655 | 1.939 | 0.073 |
| 2010 | 420500 | 0.742 | 1.288 | 0.624 | 13.49 | 5.988 | 10.83 | 2.851 | 11.85 | 10.21 | 2.285 | 0.058 |
| 2010 | 420600 | 0.714 | 0.33 | 0.305 | 12.63 | 6.382 | 10.53 | 2.734 | 12.3 | 9.893 | 1.886 | 0.042 |
| 2010 | 420700 | 0.71 | 0.939 | 0.392 | 12.62 | 4.686 | 9.218 | 2.964 | 11.34 | 10.22 | 1.942 | 0.051 |
| 2010 | 420800 | 0.723 | 0.664 | 0.259 | 12.39 | 5.705 | 9.859 | 2.624 | 11.61 | 10.12 | 1.715 | 0.029 |
| 2010 | 420900 | 0.724 | 0.611 | 0.252 | 12.2 | 6.275 | 10.2 | 2.969 | 11.67 | 9.65 | 1.661 | 0.041 |
| 2010 | 421000 | 0.724 | 0.625 | 0.69 | 11.37 | 6.489 | 11.68 | 2.362 | 10.54 | 9.546 | 1.751 | 0.029 |
| 2010 | 421100 | 0.718 | 0.517 | 0.301 | 12.55 | 6.61 | 10.74 | 3.223 | 11.43 | 9.403 | 1.961 | 0.041 |
| 2010 | 421200 | 0.728 | 0.557 | 0.605 | 12.71 | 5.673 | 10.34 | 2.532 | 11.56 | 9.549 | 1.992 | 0.055 |
| 2010 | 421300 | 0.732 | 0.511 | 0.215 | 12.33 | 5.553 | 9.036 | 2.458 | 10.39 | 9.566 | 2.085 | 0.025 |
| 2010 | 430100 | 0.724 | 2.289 | 1.341 | 14.53 | 6.481 | 13.14 | 2.662 | 14.23 | 10.42 | 1.03 | 0.131 |
| 2010 | 430200 | 0.722 | 1.624 | 0.694 | 13.19 | 5.967 | 11.14 | 2.818 | 12.51 | 10.11 | 1.524 | 0.081 |
| 2010 | 430300 | 0.726 | 1.447 | 1.148 | 13.54 | 5.666 | 11.62 | 2.733 | 12.52 | 10.03 | 1.053 | 0.071 |
| 2010 | 430400 | 0.732 | 0.579 | 0.547 | 11.96 | 6.674 | 11.46 | 2.725 | 12.52 | 9.56 | 1.016 | 0.068 |
| 2010 | 430500 | 0.702 | 0.402 | 0.215 | 11.28 | 6.677 | 10.16 | 2.56 | 10.88 | 9.088 | 0.842 | 0.045 |
| 2010 | 430600 | 0.724 | 0.674 | 0.317 | 12.67 | 6.338 | 10.51 | 2.514 | 11.75 | 9.971 | 1.507 | 0.043 |
| 2010 | 430700 | 0.736 | 0.758 | 0.333 | 12.02 | 6.435 | 10.5 | 2.727 | 12.04 | 9.826 | 1.375 | 0.058 |
| 2010 | 430800 | 0.756 | 0.388 | 0.371 | 11.79 | 5.104 | 9.439 | 2.752 | 10.11 | 9.383 | 0.541 | 0.07 |
| 2010 | 430900 | 0.715 | 0.642 | 0.372 | 11.91 | 6.166 | 10.26 | 2.324 | 11.13 | 9.387 | 0.74 | 0.042 |
| 2010 | 431000 | 0.751 | 0.621 | 0.214 | 13.05 | 6.219 | 9.924 | 1.807 | 12.79 | 9.692 | 1.109 | 0.077 |
| 2010 | 431100 | 0.719 | 0.38 | 0.256 | 11.84 | 6.415 | 10.13 | 2.624 | 12.49 | 9.323 | 0.75 | 0.048 |
| 2010 | 431200 | 0.712 | 0.669 | 0.257 | 11.72 | 6.234 | 10.38 | 2.227 | 10.66 | 9.357 | 0.702 | 0.058 |
| 2010 | 431300 | 0.74 | 0.683 | 0.326 | 12.23 | 6.071 | 10.15 | 2.553 | 11.31 | 9.387 | 1.372 | 0.057 |
| 2010 | 500000 | 0.746 | 1.475 | 0.672 | 13.2 | 8.103 | 13.25 | 2.238 | 15.28 | 9.856 | 1.272 | 0.098 |
| 2010 | 510100 | 0.739 | 3.148 | 1.239 | 13.74 | 7.047 | 13.33 | 2.701 | 15.27 | 10.64 | 1.135 | 0.111 |
| 2010 | 510300 | 0.718 | 0.61 | 0.474 | 12.27 | 5.787 | 10.34 | 2.183 | 9.228 | 9.575 | 1.378 | 0.044 |
| 2010 | 510400 | 0.749 | 1.666 | 0.644 | 13.43 | 4.713 | 9.974 | 2.176 | 9.228 | 10.49 | 3.412 | 0.094 |
| 2010 | 510500 | 0.73 | 0.453 | 0.425 | 11.78 | 6.219 | 10.48 | 2.235 | 11.86 | 9.28 | 1.244 | 0.088 |
| 2010 | 510600 | 0.727 | 1.015 | 0.589 | 12.39 | 5.964 | 10.75 | 2.519 | 9.936 | 9.981 | 1.578 | 0.052 |
| 2010 | 510700 | 0.72 | 1.081 | 0.729 | 12.77 | 6.295 | 11.34 | 2.833 | 11.54 | 9.769 | 1.141 | 0.046 |
| 2010 | 510800 | 0.721 | 0.376 | 0.077 | 12.01 | 5.739 | 8.213 | 2.449 | 9.414 | 8.978 | 0.901 | 0.065 |
| 2010 | 510900 | 0.73 | 0.359 | 0.177 | 11.63 | 5.944 | 9.21 | 2.442 | 9.414 | 9.169 | 1.114 | 0.046 |
| 2010 | 511000 | 0.725 | 0.395 | 0.317 | 11.19 | 6.053 | 9.952 | 1.785 | 9.648 | 9.319 | 1.597 | 0.042 |
| 2010 | 511100 | 0.724 | 0.631 | 0.541 | 12.62 | 5.867 | 10.51 | 2.549 | 10.35 | 9.584 | 1.88 | 0.085 |
| 2010 | 511300 | 0.742 | 0.38 | 0.437 | 11.59 | 6.622 | 10.91 | 2.496 | 11.04 | 8.984 | 0.933 | 0.052 |
| 2010 | 511400 | 0.747 | 0.545 | 0.344 | 11.52 | 5.855 | 9.67 | 2.684 | 9.149 | 9.378 | 1.567 | 0.058 |
| 2010 | 511500 | 0.727 | 0.893 | 0.247 | 12.57 | 6.29 | 9.999 | 2.109 | 9.431 | 9.476 | 1.955 | 0.077 |
| 2010 | 511600 | 0.754 | 0.187 | 0.087 | 11 | 6.145 | 8.598 | 2.226 | 10.23 | 9.12 | 1.232 | 0.049 |
| 2010 | 511700 | 0.733 | 0.164 | 0.189 | 11.57 | 6.53 | 9.833 | 1.541 | 9.709 | 9.126 | 1.288 | 0.049 |
| 2010 | 511800 | 0.746 | 0.391 | 0.793 | 12.53 | 5.043 | 10.55 | 2.161 | 6.537 | 9.589 | 2.189 | 0.063 |
| 2010 | 511900 | 0.703 | 0.215 | 0 | 11.31 | 5.961 | 5.442 | 0.438 | 11.43 | 8.818 | 0.696 | 0.03 |
| 2010 | 512000 | 0.711 | 0.516 | 0 | 11.98 | 6.217 | 6.866 | 2.431 | 10.44 | 9.112 | 1.328 | 0.051 |
| 2010 | 520100 | 0.735 | 1.92 | 1.151 | 13.88 | 5.821 | 12.46 | 1.825 | 10.08 | 10.08 | 1.559 | 0.147 |
| 2010 | 520200 | 0.717 | 0.135 | 0.218 | 12.17 | 5.766 | 9.221 | 2.317 | 11.42 | 9.059 | 2.576 | 0.171 |
| 2010 | 520300 | 0.752 | 0.436 | 0.273 | 12.09 | 6.665 | 10.82 | 1.664 | 9.43 | 9.127 | 1.568 | 0.08 |
| 2010 | 520400 | 0.723 | 0.374 | 0.323 | 12.15 | 5.634 | 9.355 | 1.617 | 9.91 | 8.796 | 1.704 | 0.106 |
| 2010 | 530100 | 0.738 | 1.62 | 1.103 | 13.54 | 6.37 | 12.51 | 2.131 | 6.412 | 10.46 | 1.307 | 0.139 |
| 2010 | 530300 | 0.724 | 0.127 | 0.145 | 12.35 | 6.44 | 9.974 | 2.396 | 7.616 | 9.323 | 2.248 | 0.116 |
| 2010 | 530400 | 0.733 | 0.854 | 0.215 | 13.22 | 5.441 | 9.395 | 2.785 | 9.594 | 10.35 | 3.56 | 0.102 |
| 2010 | 530500 | 0.704 | 0.2 | 0.185 | 11.8 | 5.532 | 9.08 | 2.515 | 10.03 | 8.968 | 0.62 | 0.111 |
| 2010 | 530600 | 0.689 | 0.056 | 0.071 | 11.93 | 6.353 | 8.69 | 2.56 | 7.616 | 8.547 | 0.899 | 0.096 |
| 2010 | 530700 | 0.73 | 0.202 | 0.413 | 12.84 | 4.791 | 9.674 | 2.923 | 7.554 | 8.982 | 0.884 | 0.174 |
| 2010 | 530800 | 0.72 | 0.159 | 0.165 | 12.71 | 5.54 | 9.019 | 1.775 | 9.93 | 8.836 | 0.966 | 0.175 |
| 2010 | 530900 | 0.733 | 0.048 | 0.122 | 11.79 | 5.494 | 8.677 | 2.375 | 7.554 | 8.833 | 1.346 | 0.09 |
| 2011 | 310000 | 0.749 | 3.536 | 0.912 | 16.47 | 7.258 | 13.14 | 1.396 | 15.91 | 11.71 | 1.387 | 0.209 |
| 2011 | 320100 | 0.741 | 2.959 | 1.696 | 15.15 | 6.456 | 13.6 | 2.977 | 14.65 | 11.12 | 1.565 | 0.137 |
| 2011 | 320200 | 0.737 | 4.082 | 0.656 | 15.46 | 6.148 | 11.59 | 3.19 | 14.63 | 11.59 | 2.024 | 0.112 |
| 2011 | 320300 | 0.751 | 2.038 | 0.529 | 13.87 | 6.884 | 11.79 | 2.858 | 13.76 | 10.13 | 1.876 | 0.121 |
| 2011 | 320400 | 0.741 | 3.478 | 0.735 | 15.05 | 5.894 | 11.73 | 3.158 | 14.49 | 11.09 | 2.187 | 0.133 |
| 2011 | 320500 | 0.744 | 4.707 | 0.832 | 15.94 | 6.465 | 12.09 | 3.3 | 15.58 | 11.63 | 2.54 | 0.134 |
| 2011 | 320600 | 0.743 | 3.737 | 0.444 | 14.35 | 6.64 | 11.22 | 3.239 | 14.15 | 10.37 | 2.044 | 0.127 |
| 2011 | 320700 | 0.741 | 1.44 | 0.307 | 13.97 | 6.225 | 10.43 | 3.052 | 12.88 | 9.793 | 1.733 | 0.175 |
| 2011 | 320800 | 0.746 | 1.476 | 0.465 | 13.96 | 6.297 | 11.12 | 2.918 | 13.86 | 9.928 | 2.081 | 0.164 |
| 2011 | 320900 | 0.746 | 1.576 | 0.338 | 14.19 | 6.71 | 10.92 | 3.043 | 13.9 | 10.01 | 1.897 | 0.124 |
| 2011 | 321000 | 0.746 | 2.49 | 0.653 | 14.56 | 6.131 | 11.26 | 2.971 | 14.12 | 10.48 | 1.916 | 0.116 |
| 2011 | 321100 | 0.741 | 3.311 | 0.94 | 14.76 | 5.605 | 11.39 | 3.081 | 13.97 | 10.95 | 2.069 | 0.099 |
| 2011 | 321200 | 0.742 | 2.42 | 0.421 | 13.86 | 6.229 | 10.77 | 3.214 | 13.73 | 10.27 | 2.035 | 0.129 |
| 2011 | 321300 | 0.738 | 0.865 | 0.15 | 13.63 | 6.319 | 9.774 | 3.221 | 11.72 | 9.499 | 1.951 | 0.148 |
| 2011 | 330100 | 0.711 | 3.719 | 1.014 | 15.43 | 6.545 | 13.01 | 2.665 | 14.93 | 11.29 | 1.651 | 0.143 |
| 2011 | 330200 | 0.72 | 4.169 | 0.793 | 15.4 | 6.357 | 11.88 | 2.717 | 14.41 | 11.28 | 1.989 | 0.14 |
| 2011 | 330300 | 0.733 | 2.683 | 0.421 | 13.73 | 6.683 | 11.23 | 2.73 | 11.1 | 10.6 | 1.992 | 0.087 |
| 2011 | 330400 | 0.719 | 3.366 | 0.462 | 14.83 | 5.838 | 10.95 | 2.86 | 13.92 | 11.03 | 2.407 | 0.103 |
| 2011 | 330500 | 0.719 | 3.319 | 0.469 | 14.38 | 5.565 | 10.14 | 3.132 | 13.32 | 10.76 | 1.931 | 0.097 |
| 2011 | 330600 | 0.715 | 3.047 | 0.429 | 14.85 | 6.087 | 10.94 | 2.883 | 13.16 | 11.06 | 2.322 | 0.086 |
| 2011 | 330700 | 0.735 | 3.225 | 0.529 | 14.39 | 6.151 | 11.26 | 3.18 | 11.92 | 10.73 | 1.869 | 0.091 |
| 2011 | 330800 | 0.741 | 1.883 | 0.2 | 14.06 | 5.532 | 9.298 | 3.235 | 10.29 | 10.06 | 1.695 | 0.096 |
| 2011 | 330900 | 0.732 | 1.966 | 0.583 | 14.86 | 4.575 | 10.04 | 2.611 | 11.15 | 10.75 | 1.268 | 0.167 |
| 2011 | 331000 | 0.725 | 2.891 | 0.248 | 13.78 | 6.375 | 10.34 | 3.232 | 11.43 | 10.65 | 2.377 | 0.079 |
| 2011 | 331100 | 0.749 | 2.108 | 0.349 | 13.99 | 5.527 | 10.51 | 2.551 | 10.26 | 9.952 | 1.571 | 0.101 |
| 2011 | 340100 | 0.7 | 2.816 | 1.217 | 14.81 | 6.56 | 12.92 | 2.935 | 13.97 | 10.27 | 1.473 | 0.234 |
| 2011 | 340200 | 0.733 | 2.975 | 0.969 | 15.15 | 5.954 | 11.85 | 3.207 | 13.42 | 10.4 | 2.117 | 0.17 |
| 2011 | 340300 | 0.739 | 1.851 | 0.599 | 14.03 | 5.901 | 10.85 | 2.688 | 12.7 | 9.695 | 1.467 | 0.106 |
| 2011 | 340400 | 0.7 | 1.361 | 0.727 | 13.61 | 5.504 | 11.08 | 2.311 | 11.56 | 9.932 | 1.696 | 0.147 |
| 2011 | 340500 | 0.74 | 1.862 | 0.815 | 14.24 | 5.432 | 10.74 | 2.82 | 13.37 | 10.6 | 2.716 | 0.165 |
| 2011 | 340600 | 0.707 | 0.991 | 0.61 | 13.17 | 5.402 | 10.36 | 2.478 | 12.18 | 9.705 | 2.021 | 0.103 |
| 2011 | 340700 | 0.735 | 2.5 | 0.903 | 14.7 | 4.307 | 10.25 | 2.441 | 11.94 | 10.53 | 1.971 | 0.154 |
| 2011 | 340800 | 0.734 | 0.596 | 0.289 | 13.24 | 6.428 | 10.54 | 2.713 | 12.08 | 9.566 | 1.726 | 0.082 |
| 2011 | 341000 | 0.747 | 1.351 | 0.411 | 14.17 | 4.998 | 9.702 | 2.976 | 11.81 | 9.829 | 0.938 | 0.147 |
| 2011 | 341100 | 0.728 | 1.695 | 0.437 | 12.86 | 6.116 | 10.64 | 3.397 | 12.36 | 9.783 | 1.57 | 0.089 |
| 2011 | 341200 | 0.727 | 0.649 | 0.24 | 11.76 | 6.933 | 10.27 | 2.843 | 10.64 | 8.798 | 0.937 | 0.093 |
| 2011 | 341300 | 0.71 | 0.373 | 0.205 | 11.97 | 6.476 | 10.39 | 3.074 | 12.11 | 9.142 | 0.866 | 0.065 |
| 2011 | 341500 | 0.726 | 0.85 | 0.318 | 12.2 | 6.565 | 10.6 | 2.802 | 11.88 | 9.037 | 1.197 | 0.098 |
| 2011 | 341600 | 0.732 | 0.481 | 0.134 | 11.87 | 6.404 | 9.285 | 3.562 | 12.06 | 9.117 | 0.918 | 0.068 |
| 2011 | 341700 | 0.738 | 1.466 | 0.635 | 13.35 | 5.084 | 10.21 | 3.205 | 11.67 | 9.411 | 1.404 | 0.187 |
| 2011 | 341800 | 0.703 | 2.088 | 0.131 | 14.24 | 5.633 | 8.706 | 3.123 | 12.21 | 9.92 | 1.315 | 0.12 |
| 2011 | 360100 | 0.715 | 1.601 | 1.632 | 13.51 | 6.225 | 13.1 | 2.186 | 14.21 | 10.46 | 1.715 | 0.104 |
| 2011 | 360200 | 0.722 | 1.072 | 0.661 | 13.06 | 5.104 | 10.13 | 2.801 | 10.77 | 10.07 | 1.835 | 0.128 |
| 2011 | 360300 | 0.723 | 0.756 | 0.272 | 13.36 | 5.249 | 8.919 | 2.823 | 11.66 | 9.889 | 2.645 | 0.143 |
| 2011 | 360400 | 0.736 | 0.639 | 0.827 | 12.37 | 6.219 | 11.35 | 3.068 | 13.12 | 9.7 | 1.91 | 0.116 |
| 2011 | 360500 | 0.741 | 1.009 | 1.055 | 13.92 | 4.782 | 10.24 | 3.119 | 12.86 | 10.15 | 1.861 | 0.214 |
| 2011 | 360600 | 0.724 | 1.115 | 0.226 | 12.76 | 4.804 | 8.475 | 2.885 | 11.45 | 9.812 | 1.735 | 0.168 |
| 2011 | 360700 | 0.733 | 0.498 | 0.44 | 11.82 | 6.823 | 11.33 | 2.26 | 13.3 | 9.256 | 1.151 | 0.113 |
| 2011 | 360800 | 0.729 | 0.49 | 0.217 | 12.2 | 6.218 | 9.819 | 2.994 | 12.69 | 9.299 | 1.682 | 0.13 |
| 2011 | 360900 | 0.718 | 0.598 | 0.303 | 12.48 | 6.34 | 10.46 | 2.577 | 12.54 | 9.419 | 1.492 | 0.13 |
| 2011 | 361000 | 0.72 | 0.368 | 0.377 | 12.49 | 6.021 | 10.77 | 2.933 | 11.64 | 9.356 | 1.731 | 0.156 |
| 2011 | 361100 | 0.732 | 0.252 | 0.156 | 12.14 | 6.62 | 9.936 | 3.025 | 12.86 | 9.197 | 1.331 | 0.132 |
| 2011 | 420100 | 0.734 | 2.716 | 1.484 | 14.43 | 6.718 | 13.73 | 2.507 | 14.7 | 10.88 | 1.222 | 0.139 |
| 2011 | 420200 | 0.71 | 1.393 | 0.568 | 13.25 | 5.561 | 10.44 | 2.85 | 12.26 | 10.2 | 1.812 | 0.07 |
| 2011 | 420300 | 0.71 | 0.879 | 0.529 | 13.29 | 5.853 | 11 | 2.667 | 11.15 | 9.752 | 1.957 | 0.096 |
| 2011 | 420500 | 0.734 | 1.574 | 0.663 | 13.73 | 5.988 | 10.88 | 2.934 | 11.69 | 10.3 | 2.306 | 0.082 |
| 2011 | 420600 | 0.705 | 0.297 | 0.342 | 13.06 | 6.386 | 10.94 | 2.762 | 12.22 | 9.975 | 1.921 | 0.069 |
| 2011 | 420700 | 0.705 | 0.875 | 0.396 | 13.28 | 4.695 | 9.347 | 3.107 | 11.4 | 10.3 | 1.978 | 0.074 |
| 2011 | 420800 | 0.714 | 0.764 | 0.286 | 12.68 | 5.71 | 9.9 | 2.671 | 11.7 | 10.2 | 1.746 | 0.043 |
| 2011 | 420900 | 0.715 | 0.692 | 0.257 | 12.5 | 6.271 | 10.22 | 2.82 | 11.77 | 9.747 | 1.676 | 0.054 |
| 2011 | 421000 | 0.716 | 0.616 | 0.68 | 12.5 | 6.496 | 11.62 | 2.346 | 10.78 | 9.627 | 1.783 | 0.041 |
| 2011 | 421100 | 0.715 | 0.523 | 0.289 | 12.88 | 6.615 | 10.65 | 3.259 | 9.61 | 9.484 | 1.997 | 0.048 |
| 2011 | 421200 | 0.722 | 0.711 | 0.506 | 13.03 | 5.688 | 10.6 | 2.656 | 11.61 | 9.646 | 2.01 | 0.073 |
| 2011 | 421300 | 0.725 | 0.618 | 0.206 | 12.67 | 5.558 | 8.96 | 2.581 | 10.6 | 9.648 | 2.122 | 0.039 |
| 2011 | 430100 | 0.72 | 2.389 | 1.34 | 14.67 | 6.487 | 13.16 | 2.581 | 14.33 | 10.51 | 1.051 | 0.156 |
| 2011 | 430200 | 0.716 | 1.697 | 0.741 | 13.14 | 5.97 | 11.17 | 2.852 | 12.67 | 10.2 | 1.555 | 0.1 |
| 2011 | 430300 | 0.724 | 1.788 | 1.138 | 13.55 | 5.671 | 11.9 | 2.741 | 12.65 | 10.12 | 1.074 | 0.085 |
| 2011 | 430400 | 0.724 | 0.737 | 0.684 | 11.96 | 6.68 | 11.68 | 2.863 | 12.68 | 9.645 | 1.036 | 0.082 |
| 2011 | 430500 | 0.7 | 0.51 | 0.22 | 11.31 | 6.681 | 10.3 | 2.615 | 10.98 | 9.173 | 0.859 | 0.052 |
| 2011 | 430600 | 0.718 | 0.897 | 0.333 | 12.97 | 6.344 | 10.54 | 2.546 | 11.7 | 10.05 | 1.535 | 0.053 |
| 2011 | 430700 | 0.729 | 0.857 | 0.318 | 12.31 | 6.439 | 10.54 | 2.811 | 12.2 | 9.908 | 1.4 | 0.069 |
| 2011 | 430800 | 0.748 | 0.432 | 0.467 | 12.08 | 5.126 | 9.921 | 2.856 | 10.22 | 9.465 | 0.55 | 0.079 |
| 2011 | 430900 | 0.71 | 0.793 | 0.348 | 12.21 | 6.171 | 10.28 | 2.52 | 11.28 | 9.471 | 0.755 | 0.051 |
| 2011 | 431000 | 0.742 | 0.658 | 0.307 | 13.28 | 6.228 | 9.933 | 2.63 | 12.96 | 9.777 | 1.131 | 0.102 |
| 2011 | 431100 | 0.719 | 0.547 | 0.253 | 12.04 | 6.448 | 10.13 | 2.68 | 12.6 | 9.407 | 0.765 | 0.058 |
| 2011 | 431200 | 0.708 | 0.445 | 0.237 | 11.83 | 6.241 | 10.47 | 2.014 | 10.73 | 9.439 | 0.715 | 0.068 |
| 2011 | 431300 | 0.731 | 0.771 | 0.355 | 12.2 | 6.077 | 10.17 | 2.527 | 11.59 | 9.469 | 1.397 | 0.067 |
| 2011 | 500000 | 0.737 | 1.709 | 0.693 | 13.53 | 8.111 | 13.33 | 2.345 | 15.74 | 9.935 | 1.319 | 0.202 |
| 2011 | 510100 | 0.731 | 2.913 | 1.172 | 14.05 | 7.059 | 13.38 | 2.707 | 15.73 | 10.73 | 1.124 | 0.124 |
| 2011 | 510300 | 0.712 | 0.687 | 0.477 | 12.44 | 5.79 | 10.3 | 2.25 | 9.497 | 9.664 | 1.382 | 0.051 |
| 2011 | 510400 | 0.74 | 2.109 | 0.64 | 13.79 | 4.716 | 9.958 | 2.203 | 9.497 | 10.58 | 3.379 | 0.104 |
| 2011 | 510500 | 0.723 | 0.556 | 0.432 | 11.85 | 6.221 | 10.53 | 2.293 | 11.49 | 9.37 | 1.232 | 0.104 |
| 2011 | 510600 | 0.725 | 1.174 | 0.607 | 12.92 | 5.967 | 10.78 | 2.612 | 10.12 | 10.03 | 1.575 | 0.066 |
| 2011 | 510700 | 0.718 | 1.245 | 0.806 | 13.11 | 6.298 | 11.42 | 2.823 | 11.73 | 9.861 | 1.144 | 0.058 |
| 2011 | 510800 | 0.719 | 0.415 | 0.08 | 12.36 | 5.741 | 8.3 | 2.479 | 9.567 | 9.057 | 0.935 | 0.076 |
| 2011 | 510900 | 0.727 | 0.434 | 0.175 | 11.78 | 5.947 | 9.269 | 2.434 | 9.611 | 9.267 | 1.111 | 0.053 |
| 2011 | 511000 | 0.718 | 0.385 | 0.309 | 11.51 | 6.055 | 9.957 | 1.823 | 10 | 9.397 | 1.657 | 0.045 |
| 2011 | 511100 | 0.721 | 0.734 | 0.545 | 12.75 | 5.87 | 10.58 | 2.614 | 11.29 | 9.663 | 1.95 | 0.096 |
| 2011 | 511300 | 0.739 | 0.313 | 0.45 | 11.82 | 6.628 | 10.96 | 2.482 | 11.27 | 9.063 | 0.968 | 0.061 |
| 2011 | 511400 | 0.743 | 0.698 | 0.345 | 11.82 | 5.86 | 9.816 | 2.73 | 10.12 | 9.467 | 1.571 | 0.069 |
| 2011 | 511500 | 0.721 | 0.704 | 0.248 | 12.84 | 6.297 | 10.01 | 2.164 | 10.9 | 9.555 | 2.028 | 0.08 |
| 2011 | 511600 | 0.749 | 0.26 | 0.092 | 11.33 | 6.15 | 8.631 | 2.243 | 10.38 | 9.199 | 1.278 | 0.056 |
| 2011 | 511700 | 0.73 | 0.246 | 0.191 | 11.84 | 6.538 | 9.844 | 1.504 | 10 | 9.224 | 1.284 | 0.057 |
| 2011 | 511800 | 0.743 | 0.691 | 0.871 | 13.58 | 5.049 | 10.58 | 2.306 | 8.588 | 9.687 | 2.184 | 0.075 |
| 2011 | 511900 | 0.695 | 0.311 | 0 | 11.57 | 5.965 | 5.442 | 0.88 | 11.58 | 8.897 | 0.722 | 0.042 |
| 2011 | 512000 | 0.709 | 0.553 | 0 | 12.38 | 6.222 | 6.866 | 2.588 | 10.4 | 9.201 | 1.332 | 0.059 |
| 2011 | 520100 | 0.725 | 1.874 | 1.176 | 14.11 | 5.93 | 12.47 | 1.681 | 10.08 | 10.18 | 1.555 | 0.173 |
| 2011 | 520200 | 0.713 | 0.171 | 0.2 | 12.41 | 5.773 | 8.912 | 2.299 | 12.1 | 9.157 | 2.569 | 0.208 |
| 2011 | 520300 | 0.745 | 0.456 | 0.289 | 12.67 | 6.649 | 10.72 | 1.654 | 9.866 | 9.225 | 1.564 | 0.101 |
| 2011 | 520400 | 0.71 | 0.471 | 0.244 | 12.46 | 5.643 | 9.446 | 1.737 | 9.976 | 8.894 | 1.699 | 0.122 |
| 2011 | 530100 | 0.73 | 1.759 | 1.219 | 14.21 | 6.299 | 12.74 | 2.428 | 6.365 | 10.55 | 1.319 | 0.152 |
| 2011 | 530300 | 0.722 | 0.244 | 0.162 | 12.38 | 6.449 | 10.12 | 2.414 | 7.857 | 9.407 | 2.246 | 0.124 |
| 2011 | 530400 | 0.727 | 1.205 | 0.228 | 13.21 | 5.446 | 9.709 | 2.146 | 9.803 | 10.44 | 3.557 | 0.107 |
| 2011 | 530500 | 0.698 | 0.218 | 0.24 | 12.53 | 5.538 | 9.723 | 2.64 | 8.923 | 9.06 | 0.626 | 0.127 |
| 2011 | 530600 | 0.681 | 0.057 | 0.064 | 12.13 | 6.362 | 8.686 | 2.612 | 7.857 | 8.641 | 0.897 | 0.107 |
| 2011 | 530700 | 0.726 | 0.385 | 0.418 | 13.24 | 4.797 | 9.852 | 2.839 | 8.637 | 9.073 | 0.892 | 0.236 |
| 2011 | 530800 | 0.711 | 0.185 | 0.176 | 13.03 | 5.546 | 9.078 | 1.914 | 9.544 | 8.921 | 0.965 | 0.194 |
| 2011 | 530900 | 0.719 | 0.108 | 0.135 | 12.27 | 5.463 | 8.702 | 2.309 | 8.637 | 8.931 | 1.342 | 0.116 |
| 2012 | 310000 | 0.746 | 3.45 | 0.914 | 16.66 | 7.263 | 13.14 | 1.406 | 16.08 | 11.8 | 1.386 | 0.212 |
| 2012 | 320100 | 0.737 | 3.238 | 1.565 | 15.52 | 6.459 | 13.61 | 3.003 | 14.77 | 11.21 | 1.583 | 0.144 |
| 2012 | 320200 | 0.737 | 4.664 | 0.671 | 15.65 | 6.153 | 11.6 | 3.195 | 14.74 | 11.69 | 2.096 | 0.109 |
| 2012 | 320300 | 0.745 | 2.181 | 0.51 | 14.06 | 6.898 | 11.66 | 3.066 | 13.89 | 10.22 | 1.943 | 0.126 |
| 2012 | 320400 | 0.743 | 3.765 | 0.705 | 15.46 | 5.899 | 11.73 | 3.199 | 14.57 | 11.18 | 2.264 | 0.131 |
| 2012 | 320500 | 0.742 | 4.884 | 0.871 | 16.15 | 6.474 | 12.17 | 3.32 | 15.57 | 11.73 | 2.63 | 0.133 |
| 2012 | 320600 | 0.739 | 3.879 | 0.434 | 14.45 | 6.64 | 11.23 | 3.12 | 14.15 | 10.47 | 2.117 | 0.13 |
| 2012 | 320700 | 0.738 | 2.058 | 0.306 | 14.2 | 6.236 | 10.43 | 3.035 | 13.05 | 9.889 | 1.795 | 0.183 |
| 2012 | 320800 | 0.741 | 1.646 | 0.445 | 14.12 | 6.304 | 11.11 | 2.955 | 14.11 | 10.02 | 2.154 | 0.169 |
| 2012 | 320900 | 0.741 | 1.758 | 0.342 | 14.49 | 6.712 | 10.91 | 3.061 | 14.1 | 10.11 | 1.964 | 0.131 |
| 2012 | 321000 | 0.741 | 2.762 | 0.629 | 14.58 | 6.128 | 11.34 | 3.029 | 14.12 | 10.58 | 1.984 | 0.109 |
| 2012 | 321100 | 0.736 | 3.425 | 0.906 | 15.14 | 5.604 | 11.32 | 3.113 | 14.15 | 11.03 | 2.09 | 0.106 |
| 2012 | 321200 | 0.738 | 2.607 | 0.441 | 13.95 | 6.227 | 10.79 | 3.26 | 13.73 | 10.36 | 2.107 | 0.12 |
| 2012 | 321300 | 0.734 | 1.415 | 0.145 | 14.06 | 6.328 | 9.748 | 3.31 | 12.56 | 9.595 | 2.02 | 0.173 |
| 2012 | 330100 | 0.71 | 3.863 | 1.002 | 15.56 | 6.552 | 13.04 | 2.673 | 14.96 | 11.37 | 1.667 | 0.144 |
| 2012 | 330200 | 0.72 | 4.466 | 0.647 | 15.54 | 6.359 | 11.89 | 2.764 | 14.4 | 11.36 | 2.009 | 0.143 |
| 2012 | 330300 | 0.728 | 2.878 | 0.44 | 13.88 | 6.685 | 11.25 | 2.766 | 12.44 | 10.68 | 2.012 | 0.086 |
| 2012 | 330400 | 0.72 | 3.361 | 0.5 | 14.93 | 5.842 | 11.01 | 2.876 | 13.93 | 11.1 | 2.432 | 0.108 |
| 2012 | 330500 | 0.716 | 3.513 | 0.498 | 14.5 | 5.566 | 10.17 | 3.143 | 13.38 | 10.84 | 1.95 | 0.102 |
| 2012 | 330600 | 0.715 | 3.149 | 0.43 | 14.91 | 6.089 | 11 | 2.818 | 13.31 | 11.15 | 2.349 | 0.088 |
| 2012 | 330700 | 0.724 | 3.452 | 0.54 | 14.61 | 6.154 | 11.28 | 3.231 | 12.09 | 10.82 | 1.891 | 0.097 |
| 2012 | 330800 | 0.737 | 2.431 | 0.203 | 14.19 | 5.533 | 9.366 | 3.248 | 10.37 | 10.15 | 1.715 | 0.098 |
| 2012 | 330900 | 0.728 | 2.297 | 0.548 | 15.09 | 4.577 | 10.04 | 2.752 | 11.66 | 10.84 | 1.283 | 0.173 |
| 2012 | 331000 | 0.722 | 2.895 | 0.242 | 14.1 | 6.382 | 10.35 | 3.245 | 12.61 | 10.73 | 2.401 | 0.081 |
| 2012 | 331100 | 0.745 | 2.357 | 0.338 | 14.11 | 5.571 | 10.55 | 2.538 | 11.09 | 10.03 | 1.587 | 0.105 |
| 2012 | 340100 | 0.696 | 2.494 | 1.214 | 14.98 | 6.566 | 12.94 | 2.76 | 13.86 | 10.35 | 1.491 | 0.241 |
| 2012 | 340200 | 0.725 | 3.071 | 0.945 | 15.49 | 5.949 | 11.9 | 3.252 | 13.65 | 10.49 | 2.121 | 0.193 |
| 2012 | 340300 | 0.729 | 2.154 | 0.611 | 14.25 | 5.908 | 10.98 | 2.78 | 13.14 | 9.789 | 1.469 | 0.238 |
| 2012 | 340400 | 0.697 | 2.026 | 0.765 | 14 | 5.496 | 11.11 | 2.492 | 11.83 | 10.03 | 1.7 | 0.286 |
| 2012 | 340500 | 0.739 | 2.101 | 0.832 | 14.66 | 5.431 | 10.82 | 2.88 | 13.65 | 10.69 | 2.721 | 0.316 |
| 2012 | 340600 | 0.704 | 1.829 | 0.645 | 13.42 | 5.386 | 10.38 | 2.485 | 12.38 | 9.799 | 2.024 | 0.125 |
| 2012 | 340700 | 0.729 | 2.966 | 0.888 | 15.59 | 4.307 | 10.32 | 2.443 | 12.26 | 10.62 | 1.975 | 0.19 |
| 2012 | 340800 | 0.729 | 0.764 | 0.319 | 13.5 | 6.43 | 10.61 | 2.738 | 12.24 | 9.65 | 1.746 | 0.166 |
| 2012 | 341000 | 0.743 | 1.52 | 0.484 | 14.49 | 4.992 | 9.758 | 2.988 | 11.84 | 9.923 | 0.94 | 0.163 |
| 2012 | 341100 | 0.723 | 1.792 | 0.434 | 13.46 | 6.114 | 10.76 | 3.596 | 12.73 | 9.876 | 1.573 | 0.104 |
| 2012 | 341200 | 0.72 | 0.683 | 0.237 | 12.11 | 6.947 | 10.5 | 2.907 | 11.1 | 8.891 | 0.939 | 0.103 |
| 2012 | 341300 | 0.705 | 0.527 | 0.18 | 12.09 | 6.48 | 10.42 | 3.147 | 12.36 | 9.236 | 0.867 | 0.079 |
| 2012 | 341500 | 0.719 | 1.064 | 0.315 | 12.75 | 6.566 | 10.58 | 2.972 | 12.15 | 9.122 | 1.211 | 0.107 |
| 2012 | 341600 | 0.728 | 0.774 | 0.132 | 11.94 | 6.418 | 9.307 | 3.657 | 12.34 | 9.202 | 0.929 | 0.147 |
| 2012 | 341700 | 0.736 | 1.601 | 0.654 | 13.69 | 5.087 | 10.3 | 3.19 | 11.8 | 9.505 | 1.407 | 0.212 |
| 2012 | 341800 | 0.701 | 1.973 | 0.149 | 14.48 | 5.633 | 9.046 | 3.237 | 12.53 | 10.01 | 1.317 | 0.136 |
| 2012 | 360100 | 0.713 | 1.746 | 1.825 | 13.69 | 6.23 | 13.14 | 2.359 | 14.33 | 10.55 | 1.701 | 0.119 |
| 2012 | 360200 | 0.721 | 1.036 | 0.643 | 13.26 | 5.116 | 10.08 | 2.779 | 11.22 | 10.16 | 1.82 | 0.148 |
| 2012 | 360300 | 0.721 | 0.777 | 0.455 | 13.61 | 5.26 | 9.328 | 2.789 | 11.86 | 9.979 | 2.623 | 0.161 |
| 2012 | 360400 | 0.73 | 0.758 | 0.867 | 12.7 | 6.232 | 11.27 | 3.093 | 13.34 | 9.79 | 1.894 | 0.145 |
| 2012 | 360500 | 0.735 | 1.15 | 0.938 | 13.99 | 4.792 | 10.3 | 3.163 | 12.75 | 10.24 | 1.846 | 0.229 |
| 2012 | 360600 | 0.721 | 0.997 | 0.223 | 13.06 | 4.82 | 8.546 | 2.622 | 11.57 | 9.902 | 1.721 | 0.222 |
| 2012 | 360700 | 0.727 | 0.614 | 0.444 | 11.99 | 6.832 | 11.36 | 2.314 | 13.38 | 9.345 | 1.142 | 0.129 |
| 2012 | 360800 | 0.723 | 0.545 | 0.183 | 12.35 | 6.226 | 9.776 | 2.828 | 12.78 | 9.388 | 1.668 | 0.156 |
| 2012 | 360900 | 0.715 | 0.675 | 0.306 | 12.74 | 6.351 | 10.48 | 2.612 | 12.61 | 9.509 | 1.48 | 0.165 |
| 2012 | 361000 | 0.713 | 0.567 | 0.311 | 12.98 | 6.035 | 10.11 | 2.928 | 11.73 | 9.445 | 1.717 | 0.161 |
| 2012 | 361100 | 0.721 | 0.346 | 0.177 | 12.18 | 6.634 | 9.983 | 3.051 | 12.96 | 9.272 | 1.303 | 0.165 |
| 2012 | 420100 | 0.73 | 2.698 | 1.503 | 14.74 | 6.711 | 13.76 | 2.667 | 14.85 | 10.96 | 1.208 | 0.153 |
| 2012 | 420200 | 0.704 | 1.499 | 0.646 | 13.39 | 5.566 | 10.69 | 2.938 | 12.49 | 10.29 | 1.804 | 0.076 |
| 2012 | 420300 | 0.703 | 1.179 | 0.527 | 13.51 | 5.846 | 10.75 | 2.711 | 11.33 | 9.842 | 1.941 | 0.1 |
| 2012 | 420500 | 0.727 | 1.793 | 0.548 | 14 | 5.989 | 10.94 | 3.011 | 11.88 | 10.39 | 2.279 | 0.098 |
| 2012 | 420600 | 0.695 | 0.7 | 0.329 | 13.55 | 6.387 | 11.03 | 2.784 | 12.53 | 10.06 | 1.912 | 0.088 |
| 2012 | 420700 | 0.699 | 0.84 | 0.353 | 13.48 | 4.695 | 9.441 | 3.192 | 11.55 | 10.39 | 1.969 | 0.082 |
| 2012 | 420800 | 0.705 | 0.787 | 0.316 | 13.1 | 5.711 | 9.962 | 2.745 | 11.83 | 10.29 | 1.738 | 0.05 |
| 2012 | 420900 | 0.706 | 0.829 | 0.258 | 13 | 6.267 | 10.22 | 2.902 | 11.92 | 9.832 | 1.657 | 0.065 |
| 2012 | 421000 | 0.71 | 0.706 | 0.687 | 12.74 | 6.497 | 11.64 | 2.425 | 10.95 | 9.712 | 1.762 | 0.047 |
| 2012 | 421100 | 0.713 | 0.538 | 0.292 | 13.11 | 6.618 | 10.77 | 3.25 | 10.04 | 9.571 | 1.988 | 0.052 |
| 2012 | 421200 | 0.716 | 0.559 | 0.484 | 13.31 | 5.697 | 10.59 | 2.65 | 11.8 | 9.736 | 1.994 | 0.084 |
| 2012 | 421300 | 0.717 | 0.795 | 0.19 | 12.95 | 5.549 | 9.053 | 2.388 | 10.73 | 9.734 | 2.113 | 0.047 |
| 2012 | 430100 | 0.717 | 2.621 | 1.359 | 14.75 | 6.493 | 13.17 | 2.533 | 14.45 | 10.59 | 1.061 | 0.161 |
| 2012 | 430200 | 0.711 | 1.822 | 0.75 | 13.56 | 5.981 | 11.2 | 2.814 | 12.81 | 10.28 | 1.548 | 0.11 |
| 2012 | 430300 | 0.719 | 1.917 | 1.179 | 13.52 | 5.676 | 11.68 | 2.742 | 12.82 | 10.21 | 1.069 | 0.099 |
| 2012 | 430400 | 0.717 | 0.692 | 0.554 | 12.14 | 6.685 | 11.74 | 2.871 | 12.85 | 9.731 | 1.032 | 0.096 |
| 2012 | 430500 | 0.698 | 0.535 | 0.226 | 11.3 | 6.686 | 10.23 | 2.501 | 11.56 | 9.253 | 0.868 | 0.06 |
| 2012 | 430600 | 0.714 | 0.98 | 0.438 | 13.15 | 6.351 | 10.58 | 2.578 | 11.87 | 10.14 | 1.528 | 0.059 |
| 2012 | 430700 | 0.722 | 0.924 | 0.342 | 12.36 | 6.444 | 10.57 | 2.862 | 12.46 | 9.994 | 1.394 | 0.07 |
| 2012 | 430800 | 0.745 | 0.516 | 0.354 | 12.59 | 5.136 | 9.404 | 2.843 | 10.42 | 9.551 | 0.548 | 0.087 |
| 2012 | 430900 | 0.706 | 0.912 | 0.381 | 12.5 | 6.178 | 10.26 | 2.546 | 11.41 | 9.557 | 0.752 | 0.059 |
| 2012 | 431000 | 0.735 | 0.731 | 0.235 | 13.44 | 6.234 | 9.95 | 2.686 | 13.07 | 9.857 | 1.142 | 0.115 |
| 2012 | 431100 | 0.719 | 0.594 | 0.255 | 12.08 | 6.43 | 10.12 | 2.712 | 12.74 | 9.493 | 0.761 | 0.068 |
| 2012 | 431200 | 0.701 | 0.433 | 0.238 | 12.18 | 6.247 | 10.57 | 2.096 | 10.85 | 9.525 | 0.711 | 0.087 |
| 2012 | 431300 | 0.722 | 0.966 | 0.334 | 12.15 | 6.082 | 10.1 | 2.208 | 11.74 | 9.555 | 1.39 | 0.078 |
| 2012 | 500000 | 0.718 | 1.807 | 0.672 | 13.83 | 8.115 | 13.42 | 2.367 | 15.71 | 10.02 | 1.332 | 0.203 |
| 2012 | 510100 | 0.727 | 3.161 | 1.14 | 14.3 | 7.068 | 13.44 | 2.787 | 15.71 | 10.82 | 1.11 | 0.127 |
| 2012 | 510300 | 0.709 | 0.813 | 0.529 | 12.9 | 5.795 | 10.33 | 2.248 | 8.877 | 9.758 | 1.365 | 0.051 |
| 2012 | 510400 | 0.737 | 2.081 | 0.64 | 13.89 | 4.718 | 9.983 | 2.383 | 9.476 | 10.68 | 3.337 | 0.107 |
| 2012 | 510500 | 0.716 | 0.608 | 0.44 | 12.13 | 6.225 | 10.63 | 2.433 | 11.09 | 9.464 | 1.216 | 0.117 |
| 2012 | 510600 | 0.724 | 1.142 | 0.638 | 13.27 | 5.97 | 10.91 | 2.546 | 10.39 | 10.13 | 1.556 | 0.067 |
| 2012 | 510700 | 0.714 | 1.265 | 0.767 | 13.54 | 6.302 | 11.54 | 2.727 | 11.71 | 9.955 | 1.13 | 0.064 |
| 2012 | 510800 | 0.717 | 0.484 | 0.127 | 12.5 | 5.742 | 8.684 | 2.604 | 9.303 | 9.137 | 0.944 | 0.08 |
| 2012 | 510900 | 0.721 | 0.665 | 0.2 | 12.17 | 5.93 | 9.427 | 2.563 | 10.06 | 9.361 | 1.097 | 0.056 |
| 2012 | 511000 | 0.713 | 0.48 | 0.31 | 11.95 | 6.056 | 10.01 | 1.936 | 10.37 | 9.478 | 1.674 | 0.049 |
| 2012 | 511100 | 0.718 | 0.809 | 0.595 | 12.91 | 5.872 | 10.66 | 2.663 | 11.45 | 9.743 | 1.97 | 0.101 |
| 2012 | 511300 | 0.73 | 0.4 | 0.409 | 11.95 | 6.633 | 11.04 | 2.558 | 11.3 | 9.144 | 0.978 | 0.066 |
| 2012 | 511400 | 0.738 | 0.754 | 0.376 | 12.01 | 5.859 | 9.933 | 2.766 | 10.5 | 9.562 | 1.551 | 0.087 |
| 2012 | 511500 | 0.716 | 0.774 | 0.244 | 13.06 | 6.304 | 10.06 | 2.039 | 10.46 | 9.636 | 2.048 | 0.089 |
| 2012 | 511600 | 0.742 | 0.316 | 0.095 | 11.73 | 6.15 | 8.813 | 2.319 | 10.26 | 9.28 | 1.291 | 0.059 |
| 2012 | 511700 | 0.726 | 0.284 | 0.197 | 12.1 | 6.545 | 9.893 | 1.366 | 10.13 | 9.319 | 1.268 | 0.064 |
| 2012 | 511800 | 0.739 | 0.792 | 0.866 | 14.31 | 5.053 | 10.63 | 2.406 | 8.846 | 9.781 | 2.156 | 0.093 |
| 2012 | 511900 | 0.686 | 0.18 | 0 | 11.8 | 5.966 | 5.442 | 0.875 | 11.75 | 8.977 | 0.729 | 0.058 |
| 2012 | 512000 | 0.704 | 0.523 | 0 | 12.74 | 6.226 | 6.866 | 2.649 | 10.42 | 9.296 | 1.315 | 0.066 |
| 2012 | 520100 | 0.717 | 2.012 | 1.245 | 14.38 | 5.926 | 12.88 | 1.668 | 10.16 | 10.25 | 1.556 | 0.195 |
| 2012 | 520200 | 0.703 | 0.465 | 0.196 | 12.63 | 5.776 | 9.087 | 2.377 | 12.61 | 9.251 | 2.537 | 0.263 |
| 2012 | 520300 | 0.733 | 0.765 | 0.322 | 12.87 | 6.648 | 10.88 | 1.645 | 10.95 | 9.304 | 1.565 | 0.118 |
| 2012 | 520400 | 0.695 | 0.673 | 0.331 | 12.64 | 5.65 | 9.482 | 1.837 | 9.938 | 8.989 | 1.678 | 0.15 |
| 2012 | 530100 | 0.726 | 1.867 | 1.17 | 14.4 | 6.298 | 12.8 | 2.262 | 9.944 | 10.63 | 1.283 | 0.161 |
| 2012 | 530300 | 0.713 | 0.215 | 0.158 | 12.93 | 6.457 | 10.17 | 2.416 | 8.645 | 9.482 | 2.186 | 0.129 |
| 2012 | 530400 | 0.722 | 1.235 | 0.314 | 13.56 | 5.366 | 9.509 | 3.076 | 10.13 | 10.51 | 3.462 | 0.111 |
| 2012 | 530500 | 0.696 | 0.254 | 0.258 | 12.68 | 5.544 | 9.218 | 2.523 | 10.36 | 9.135 | 0.609 | 0.142 |
| 2012 | 530600 | 0.681 | 0.09 | 0.062 | 12.24 | 6.369 | 8.695 | 2.638 | 8.645 | 8.716 | 0.873 | 0.115 |
| 2012 | 530700 | 0.721 | 0.452 | 0.38 | 13.26 | 4.779 | 9.412 | 2.885 | 9.805 | 9.152 | 0.893 | 0.295 |
| 2012 | 530800 | 0.707 | 0.312 | 0.177 | 12.76 | 5.528 | 9.053 | 2.154 | 10.36 | 8.996 | 0.939 | 0.209 |
| 2012 | 530900 | 0.712 | 0.142 | 0.141 | 12 | 5.466 | 8.651 | 2.476 | 9.805 | 9.01 | 1.343 | 0.144 |
| 2013 | 310000 | 0.741 | 3.594 | 0.885 | 16.71 | 7.267 | 13.13 | 1.413 | 16.16 | 11.87 | 1.349 | 0.215 |
| 2013 | 320100 | 0.725 | 3.462 | 1.732 | 15.65 | 6.466 | 13.6 | 3.058 | 14.73 | 11.29 | 1.541 | 0.148 |
| 2013 | 320200 | 0.722 | 4.482 | 0.653 | 15.82 | 6.157 | 11.62 | 3.208 | 14.54 | 11.78 | 2.104 | 0.107 |
| 2013 | 320300 | 0.727 | 2.401 | 0.564 | 14.18 | 6.915 | 11.82 | 3.113 | 13.74 | 10.31 | 1.95 | 0.132 |
| 2013 | 320400 | 0.723 | 3.927 | 0.716 | 15.61 | 5.902 | 11.75 | 3.238 | 14.6 | 11.26 | 2.204 | 0.129 |
| 2013 | 320500 | 0.718 | 4.943 | 0.776 | 16.27 | 6.483 | 12.22 | 3.329 | 15.5 | 11.82 | 2.639 | 0.134 |
| 2013 | 320600 | 0.709 | 3.395 | 0.483 | 14.73 | 6.642 | 11.24 | 3.272 | 14.16 | 10.54 | 2.06 | 0.136 |
| 2013 | 320700 | 0.728 | 2.212 | 0.332 | 14.35 | 6.254 | 10.53 | 3.049 | 13.2 | 9.981 | 1.801 | 0.186 |
| 2013 | 320800 | 0.729 | 2.294 | 0.443 | 14.29 | 6.315 | 11.11 | 3.013 | 13.6 | 10.12 | 2.162 | 0.178 |
| 2013 | 320900 | 0.726 | 1.97 | 0.352 | 14.69 | 6.714 | 10.91 | 3.064 | 13.77 | 10.2 | 1.971 | 0.139 |
| 2013 | 321000 | 0.715 | 3.262 | 0.644 | 14.64 | 6.131 | 11.31 | 3.057 | 14.01 | 10.67 | 1.992 | 0.114 |
| 2013 | 321100 | 0.729 | 3.575 | 0.894 | 15.19 | 5.605 | 11.33 | 3.173 | 14.47 | 11.12 | 2.098 | 0.114 |
| 2013 | 321200 | 0.713 | 2.957 | 0.444 | 14.23 | 6.23 | 10.8 | 3.197 | 13.62 | 10.46 | 2.115 | 0.123 |
| 2013 | 321300 | 0.71 | 2.164 | 0.158 | 14.29 | 6.349 | 9.772 | 3.321 | 12.66 | 9.67 | 1.967 | 0.183 |
| 2013 | 330100 | 0.711 | 4.146 | 0.993 | 15.69 | 6.56 | 13.06 | 2.647 | 15 | 11.42 | 1.663 | 0.146 |
| 2013 | 330200 | 0.72 | 4.621 | 0.631 | 15.68 | 6.363 | 11.91 | 2.756 | 14.52 | 11.44 | 1.996 | 0.144 |
| 2013 | 330300 | 0.727 | 3.419 | 0.432 | 14.03 | 6.694 | 11.28 | 2.623 | 12.65 | 10.74 | 2.007 | 0.088 |
| 2013 | 330400 | 0.719 | 3.789 | 0.453 | 15.06 | 5.846 | 11.06 | 2.876 | 14.13 | 11.19 | 2.394 | 0.109 |
| 2013 | 330500 | 0.716 | 3.693 | 0.494 | 14.64 | 5.57 | 10.19 | 3.153 | 13.39 | 10.89 | 1.945 | 0.105 |
| 2013 | 330600 | 0.714 | 3.576 | 0.549 | 15.09 | 6.091 | 11.26 | 2.88 | 13.12 | 11.2 | 2.343 | 0.09 |
| 2013 | 330700 | 0.723 | 3.563 | 0.557 | 14.76 | 6.16 | 11.31 | 3.247 | 11.95 | 10.88 | 1.886 | 0.101 |
| 2013 | 330800 | 0.736 | 2.675 | 0.225 | 14.48 | 5.538 | 9.468 | 3.326 | 10.62 | 10.2 | 1.71 | 0.103 |
| 2013 | 330900 | 0.725 | 2.846 | 0.563 | 15.23 | 4.578 | 10.06 | 2.911 | 11.77 | 10.89 | 1.279 | 0.172 |
| 2013 | 331000 | 0.72 | 3.112 | 0.237 | 14.21 | 6.387 | 10.37 | 3.289 | 12.42 | 10.82 | 2.41 | 0.084 |
| 2013 | 331100 | 0.743 | 2.846 | 0.338 | 14.19 | 5.576 | 10.59 | 2.519 | 11.28 | 10.12 | 1.593 | 0.111 |
| 2013 | 340100 | 0.69 | 2.848 | 1.254 | 15.11 | 6.567 | 13 | 2.806 | 13.99 | 10.41 | 1.487 | 0.246 |
| 2013 | 340200 | 0.718 | 3.326 | 0.956 | 15.74 | 5.952 | 11.95 | 3.313 | 13.83 | 10.57 | 2.143 | 0.208 |
| 2013 | 340300 | 0.722 | 2.388 | 0.663 | 14.5 | 5.904 | 11.05 | 2.861 | 13.35 | 9.866 | 1.484 | 0.129 |
| 2013 | 340400 | 0.694 | 2.15 | 0.756 | 14.12 | 5.494 | 11.3 | 2.63 | 11.94 | 10.1 | 1.717 | 0.182 |
| 2013 | 340500 | 0.728 | 2.89 | 0.863 | 14.8 | 5.431 | 10.84 | 2.891 | 13.89 | 10.77 | 2.728 | 0.21 |
| 2013 | 340600 | 0.696 | 2.215 | 0.637 | 13.35 | 5.368 | 10.41 | 2.556 | 12.56 | 9.876 | 2.045 | 0.111 |
| 2013 | 340700 | 0.719 | 3.322 | 0.904 | 15.69 | 4.307 | 10.39 | 2.441 | 12.43 | 10.69 | 1.98 | 0.176 |
| 2013 | 340800 | 0.72 | 1.129 | 0.32 | 13.71 | 6.432 | 10.62 | 2.812 | 12.54 | 9.706 | 1.741 | 0.09 |
| 2013 | 341000 | 0.736 | 1.993 | 0.49 | 14.58 | 4.993 | 9.848 | 2.999 | 11.96 | 9.996 | 0.942 | 0.156 |
| 2013 | 341100 | 0.715 | 2.081 | 0.464 | 13.56 | 6.108 | 10.69 | 3.672 | 13.02 | 9.953 | 1.589 | 0.111 |
| 2013 | 341200 | 0.718 | 1.029 | 0.243 | 12.1 | 6.96 | 10.47 | 3.023 | 11.33 | 8.965 | 0.942 | 0.115 |
| 2013 | 341300 | 0.697 | 0.813 | 0.152 | 12.27 | 6.464 | 9.926 | 3.185 | 12.62 | 9.312 | 0.876 | 0.089 |
| 2013 | 341500 | 0.716 | 1.557 | 0.325 | 12.82 | 6.575 | 10.6 | 3.021 | 12.15 | 9.178 | 1.208 | 0.114 |
| 2013 | 341600 | 0.718 | 0.973 | 0.142 | 12.38 | 6.45 | 9.378 | 3.643 | 12.6 | 9.258 | 0.926 | 0.103 |
| 2013 | 341700 | 0.723 | 2.028 | 0.685 | 13.92 | 5.087 | 9.969 | 3.196 | 12 | 9.582 | 1.421 | 0.236 |
| 2013 | 341800 | 0.698 | 2.293 | 0.194 | 14.7 | 5.636 | 9.329 | 3.282 | 12.78 | 10.09 | 1.331 | 0.151 |
| 2013 | 360100 | 0.706 | 2.033 | 1.911 | 13.88 | 6.235 | 13.16 | 2.339 | 14.42 | 10.63 | 1.66 | 0.131 |
| 2013 | 360200 | 0.717 | 1.246 | 0.636 | 13.44 | 5.114 | 10.05 | 2.793 | 11.37 | 10.24 | 1.776 | 0.149 |
| 2013 | 360300 | 0.709 | 1.061 | 0.397 | 14.04 | 5.266 | 9.246 | 2.735 | 11.97 | 10.05 | 2.514 | 0.168 |
| 2013 | 360400 | 0.726 | 0.978 | 0.762 | 13.53 | 6.231 | 11.28 | 3.132 | 13.54 | 9.867 | 1.848 | 0.162 |
| 2013 | 360500 | 0.723 | 1.457 | 0.939 | 14.35 | 4.8 | 10.28 | 3.16 | 12.18 | 10.31 | 1.801 | 0.213 |
| 2013 | 360600 | 0.711 | 1.057 | 0.23 | 14.17 | 4.826 | 8.532 | 2.625 | 11.69 | 9.975 | 1.649 | 0.227 |
| 2013 | 360700 | 0.72 | 1.047 | 0.483 | 13.1 | 6.834 | 11.35 | 2.395 | 13.44 | 9.418 | 1.094 | 0.152 |
| 2013 | 360800 | 0.71 | 0.759 | 0.225 | 13.9 | 6.233 | 9.782 | 2.877 | 12.96 | 9.465 | 1.628 | 0.166 |
| 2013 | 360900 | 0.708 | 0.893 | 0.326 | 13.36 | 6.36 | 10.69 | 2.638 | 12.71 | 9.586 | 1.444 | 0.179 |
| 2013 | 361000 | 0.706 | 0.81 | 0.273 | 13.26 | 6.039 | 10.12 | 2.99 | 11.83 | 9.522 | 1.675 | 0.168 |
| 2013 | 361100 | 0.719 | 0.549 | 0.17 | 12.81 | 6.633 | 10.03 | 3.081 | 13.06 | 9.349 | 1.271 | 0.182 |
| 2013 | 420100 | 0.726 | 3.029 | 1.455 | 15.16 | 6.712 | 13.78 | 2.591 | 14.99 | 11.04 | 1.158 | 0.163 |
| 2013 | 420200 | 0.701 | 1.659 | 0.602 | 13.39 | 5.569 | 10.51 | 2.963 | 12.62 | 10.37 | 1.778 | 0.082 |
| 2013 | 420300 | 0.695 | 1.45 | 0.554 | 13.72 | 5.848 | 10.8 | 2.761 | 11.48 | 9.914 | 1.86 | 0.087 |
| 2013 | 420500 | 0.718 | 2.124 | 0.627 | 14.27 | 5.992 | 11 | 3.058 | 12.03 | 10.46 | 2.184 | 0.119 |
| 2013 | 420600 | 0.688 | 0.365 | 0.335 | 13.57 | 6.389 | 11.08 | 2.917 | 12.72 | 10.15 | 1.884 | 0.108 |
| 2013 | 420700 | 0.697 | 1.121 | 0.422 | 13.75 | 4.699 | 9.482 | 3.252 | 11.52 | 10.47 | 1.94 | 0.088 |
| 2013 | 420800 | 0.702 | 1.343 | 0.33 | 13.55 | 5.706 | 9.963 | 2.791 | 12 | 10.37 | 1.713 | 0.053 |
| 2013 | 420900 | 0.698 | 1.024 | 0.244 | 13.12 | 6.268 | 10.54 | 2.919 | 12.25 | 9.904 | 1.588 | 0.075 |
| 2013 | 421000 | 0.704 | 0.946 | 0.653 | 13.04 | 6.494 | 11.64 | 2.505 | 11.11 | 9.797 | 1.737 | 0.054 |
| 2013 | 421100 | 0.705 | 0.728 | 0.443 | 13.4 | 6.62 | 10.78 | 3.273 | 10.64 | 9.656 | 1.959 | 0.06 |
| 2013 | 421200 | 0.71 | 0.789 | 0.485 | 13.45 | 5.705 | 10.57 | 2.572 | 11.94 | 9.808 | 1.911 | 0.097 |
| 2013 | 421300 | 0.71 | 1.052 | 0.189 | 13.23 | 5.551 | 8.825 | 2.252 | 10.9 | 9.819 | 2.082 | 0.055 |
| 2013 | 430100 | 0.714 | 2.834 | 1.379 | 14.93 | 6.496 | 13.26 | 2.546 | 14.56 | 10.66 | 1.046 | 0.16 |
| 2013 | 430200 | 0.707 | 2.118 | 0.707 | 13.78 | 5.99 | 11.21 | 2.858 | 12.97 | 10.35 | 1.549 | 0.11 |
| 2013 | 430300 | 0.713 | 2.104 | 1.189 | 13.89 | 5.67 | 11.71 | 2.851 | 12.98 | 10.28 | 1.07 | 0.104 |
| 2013 | 430400 | 0.709 | 1.145 | 0.591 | 12.41 | 6.667 | 11.53 | 2.872 | 13.06 | 9.802 | 1.033 | 0.101 |
| 2013 | 430500 | 0.696 | 0.662 | 0.231 | 11.72 | 6.695 | 10.21 | 2.56 | 11.35 | 9.325 | 0.868 | 0.069 |
| 2013 | 430600 | 0.71 | 1.137 | 0.45 | 13.27 | 6.328 | 10.6 | 2.59 | 12.05 | 10.22 | 1.506 | 0.062 |
| 2013 | 430700 | 0.716 | 1.092 | 0.373 | 12.72 | 6.409 | 10.58 | 2.794 | 12.66 | 10.08 | 1.374 | 0.073 |
| 2013 | 430800 | 0.741 | 0.602 | 0.372 | 12.33 | 5.141 | 9.387 | 2.82 | 10.63 | 9.636 | 0.54 | 0.089 |
| 2013 | 430900 | 0.703 | 1.123 | 0.395 | 12.84 | 6.174 | 10.29 | 2.537 | 11.56 | 9.629 | 0.752 | 0.063 |
| 2013 | 431000 | 0.728 | 1.108 | 0.226 | 13.56 | 6.239 | 9.988 | 2.72 | 13.35 | 9.929 | 1.143 | 0.127 |
| 2013 | 431100 | 0.717 | 0.844 | 0.251 | 12.42 | 6.434 | 10.12 | 2.779 | 12.87 | 9.564 | 0.762 | 0.072 |
| 2013 | 431200 | 0.699 | 0.633 | 0.255 | 12.38 | 6.244 | 10.55 | 2.158 | 10.97 | 9.596 | 0.712 | 0.088 |
| 2013 | 431300 | 0.718 | 1.009 | 0.355 | 12.69 | 6.084 | 10.25 | 2.231 | 12.07 | 9.64 | 1.37 | 0.085 |
| 2013 | 500000 | 0.711 | 2.135 | 0.667 | 13.96 | 8.119 | 13.47 | 2.419 | 15.7 | 10.09 | 1.313 | 0.182 |
| 2013 | 510100 | 0.707 | 3.423 | 1.163 | 14.37 | 7.08 | 13.46 | 2.763 | 15.7 | 10.9 | 1.094 | 0.133 |
| 2013 | 510300 | 0.701 | 1.135 | 0.563 | 13.38 | 5.798 | 10.36 | 2.338 | 9.416 | 9.84 | 1.347 | 0.054 |
| 2013 | 510400 | 0.726 | 2.284 | 0.632 | 14.12 | 4.718 | 9.992 | 2.442 | 9.555 | 10.75 | 3.288 | 0.1 |
| 2013 | 510500 | 0.707 | 0.732 | 0.401 | 13.07 | 6.231 | 10.67 | 2.449 | 11.14 | 9.537 | 1.198 | 0.139 |
| 2013 | 510600 | 0.713 | 1.401 | 0.68 | 13.38 | 5.971 | 10.93 | 2.565 | 10.4 | 10.2 | 1.533 | 0.065 |
| 2013 | 510700 | 0.708 | 1.835 | 0.779 | 13.83 | 6.305 | 11.62 | 2.56 | 11.74 | 10.04 | 1.115 | 0.065 |
| 2013 | 510800 | 0.712 | 0.81 | 0.134 | 12.83 | 5.737 | 9.043 | 2.618 | 9.302 | 9.209 | 0.931 | 0.083 |
| 2013 | 510900 | 0.714 | 0.942 | 0.208 | 12.36 | 5.939 | 9.591 | 3.22 | 10.27 | 9.443 | 1.083 | 0.06 |
| 2013 | 511000 | 0.708 | 0.683 | 0.368 | 11.94 | 6.056 | 10.06 | 1.98 | 10.44 | 9.55 | 1.649 | 0.054 |
| 2013 | 511100 | 0.714 | 0.927 | 0.591 | 13.01 | 5.875 | 10.69 | 2.684 | 11.03 | 9.816 | 1.941 | 0.098 |
| 2013 | 511300 | 0.717 | 0.619 | 0.45 | 12.1 | 6.632 | 11.11 | 2.588 | 11.09 | 9.216 | 0.964 | 0.074 |
| 2013 | 511400 | 0.728 | 0.896 | 0.375 | 12.26 | 5.864 | 10.01 | 2.787 | 10.36 | 9.643 | 1.531 | 0.102 |
| 2013 | 511500 | 0.714 | 1.043 | 0.241 | 13.22 | 6.311 | 10.09 | 2.093 | 10.51 | 9.708 | 2.018 | 0.098 |
| 2013 | 511600 | 0.736 | 0.537 | 0.116 | 11.94 | 6.154 | 8.98 | 2.539 | 10.25 | 9.352 | 1.272 | 0.063 |
| 2013 | 511700 | 0.717 | 0.455 | 0.209 | 12.21 | 6.533 | 9.969 | 1.218 | 10.14 | 9.4 | 1.252 | 0.067 |
| 2013 | 511800 | 0.736 | 1.329 | 0.889 | 13.6 | 5.056 | 10.66 | 2.439 | 9.194 | 9.863 | 2.129 | 0.065 |
| 2013 | 511900 | 0.674 | 0.323 | 0.01 | 11.99 | 5.967 | 5.442 | 1.051 | 11.74 | 9.05 | 0.719 | 0.072 |
| 2013 | 512000 | 0.69 | 0.78 | 0.036 | 12.85 | 6.229 | 6.866 | 2.664 | 10.3 | 9.377 | 1.298 | 0.071 |
| 2013 | 520100 | 0.709 | 2.37 | 1.287 | 14.72 | 5.938 | 12.69 | 2.276 | 8.864 | 10.34 | 1.537 | 0.199 |
| 2013 | 520200 | 0.698 | 0.817 | 0.175 | 12.85 | 5.785 | 9.169 | 2.52 | 12.87 | 9.333 | 2.505 | 0.277 |
| 2013 | 520300 | 0.725 | 1.038 | 0.312 | 12.94 | 6.657 | 10.98 | 1.623 | 12.06 | 9.385 | 1.545 | 0.126 |
| 2013 | 520400 | 0.69 | 1.074 | 0.297 | 12.81 | 5.655 | 9.48 | 2.086 | 10.83 | 9.07 | 1.657 | 0.166 |
| 2013 | 530100 | 0.72 | 2.209 | 1.192 | 14.62 | 6.304 | 12.86 | 2.224 | 10.16 | 10.71 | 1.267 | 0.17 |
| 2013 | 530300 | 0.71 | 0.441 | 0.172 | 12.9 | 6.464 | 10.08 | 2.611 | 9.125 | 9.56 | 2.146 | 0.135 |
| 2013 | 530400 | 0.718 | 1.437 | 0.254 | 13.79 | 5.369 | 9.753 | 3.091 | 10.48 | 10.59 | 3.399 | 0.116 |
| 2013 | 530500 | 0.693 | 0.326 | 0.232 | 12.73 | 5.549 | 9.314 | 2.46 | 10.13 | 9.217 | 0.601 | 0.152 |
| 2013 | 530600 | 0.682 | 0.116 | 0.059 | 12.25 | 6.374 | 8.798 | 2.638 | 7.527 | 8.798 | 0.862 | 0.123 |
| 2013 | 530700 | 0.717 | 0.624 | 0.453 | 13.52 | 4.788 | 10.06 | 2.573 | 10.12 | 9.234 | 0.882 | 0.314 |
| 2013 | 530800 | 0.705 | 0.379 | 0.18 | 12.98 | 5.555 | 9.156 | 1.946 | 10.8 | 9.074 | 0.922 | 0.209 |
| 2013 | 530900 | 0.706 | 0.142 | 0.125 | 12.44 | 5.466 | 8.635 | 2.424 | 10.12 | 9.091 | 1.326 | 0.155 |
| 2014 | 310000 | 0.739 | 3.555 | 0.794 | 16.72 | 7.271 | 13.14 | 1.413 | 16.23 | 11.95 | 1.324 | 0.236 |
| 2014 | 320100 | 0.722 | 3.556 | 1.476 | 15.75 | 6.475 | 13.6 | 3.099 | 14.52 | 11.36 | 1.513 | 0.148 |
| 2014 | 320200 | 0.723 | 4.071 | 0.661 | 15.82 | 6.168 | 11.65 | 3.228 | 14.39 | 11.85 | 2.066 | 0.107 |
| 2014 | 320300 | 0.725 | 2.281 | 0.567 | 14.35 | 6.931 | 11.83 | 3.231 | 13.83 | 10.39 | 1.914 | 0.136 |
| 2014 | 320400 | 0.724 | 3.917 | 0.707 | 15.59 | 5.91 | 11.75 | 3.239 | 14.21 | 11.34 | 2.164 | 0.126 |
| 2014 | 320500 | 0.716 | 4.378 | 0.797 | 16.25 | 6.494 | 12.25 | 3.337 | 15.42 | 11.88 | 2.603 | 0.134 |
| 2014 | 320600 | 0.705 | 2.842 | 0.507 | 14.86 | 6.643 | 11.3 | 3.376 | 14.16 | 10.62 | 2.023 | 0.142 |
| 2014 | 320700 | 0.725 | 2.534 | 0.333 | 14.46 | 6.266 | 10.55 | 3.063 | 13.28 | 10.04 | 1.776 | 0.192 |
| 2014 | 320800 | 0.723 | 2.545 | 0.433 | 14.45 | 6.328 | 11.12 | 3.04 | 13.51 | 10.18 | 2.133 | 0.186 |
| 2014 | 320900 | 0.724 | 1.847 | 0.356 | 14.86 | 6.72 | 10.93 | 3 | 13.37 | 10.26 | 1.944 | 0.146 |
| 2014 | 321000 | 0.711 | 3.206 | 0.61 | 14.71 | 6.134 | 11.3 | 3.077 | 13.66 | 10.75 | 1.956 | 0.12 |
| 2014 | 321100 | 0.725 | 3.814 | 0.897 | 15.29 | 5.606 | 11.34 | 3.186 | 13.59 | 11.18 | 2.069 | 0.114 |
| 2014 | 321200 | 0.71 | 2.913 | 0.453 | 14.22 | 6.231 | 10.81 | 3.19 | 13.27 | 10.53 | 2.077 | 0.125 |
| 2014 | 321300 | 0.711 | 2.006 | 0.154 | 14.34 | 6.364 | 9.777 | 3.321 | 12.92 | 9.748 | 1.931 | 0.191 |
| 2014 | 330100 | 0.71 | 3.844 | 0.988 | 15.81 | 6.573 | 13.07 | 2.693 | 15.17 | 11.48 | 1.64 | 0.148 |
| 2014 | 330200 | 0.719 | 4.299 | 0.646 | 15.81 | 6.37 | 11.92 | 2.763 | 14.72 | 11.5 | 1.969 | 0.145 |
| 2014 | 330300 | 0.724 | 3.405 | 0.431 | 14.14 | 6.702 | 11.31 | 2.719 | 12.7 | 10.8 | 1.979 | 0.089 |
| 2014 | 330400 | 0.718 | 3.911 | 0.453 | 15.21 | 5.852 | 11.09 | 2.84 | 14.24 | 11.25 | 2.362 | 0.11 |
| 2014 | 330500 | 0.715 | 3.866 | 0.387 | 14.79 | 5.575 | 10.2 | 3.218 | 13.31 | 10.97 | 1.894 | 0.105 |
| 2014 | 330600 | 0.713 | 3.642 | 0.538 | 15.24 | 6.094 | 11.29 | 2.889 | 12.93 | 11.28 | 2.282 | 0.09 |
| 2014 | 330700 | 0.72 | 3.476 | 0.604 | 14.85 | 6.164 | 11.37 | 3.273 | 12.05 | 10.95 | 1.837 | 0.103 |
| 2014 | 330800 | 0.734 | 2.468 | 0.227 | 14.64 | 5.544 | 9.523 | 3.436 | 10.67 | 10.28 | 1.666 | 0.106 |
| 2014 | 330900 | 0.697 | 3.05 | 0.557 | 15.34 | 4.58 | 10.03 | 2.903 | 11.72 | 10.97 | 1.246 | 0.174 |
| 2014 | 331000 | 0.718 | 3.287 | 0.242 | 14.31 | 6.392 | 10.39 | 3.324 | 12.04 | 10.88 | 2.377 | 0.083 |
| 2014 | 331100 | 0.741 | 2.691 | 0.355 | 14.37 | 5.582 | 10.59 | 2.533 | 11.6 | 10.18 | 1.571 | 0.113 |
| 2014 | 340100 | 0.691 | 2.899 | 1.245 | 15.22 | 6.569 | 13.12 | 2.797 | 14.11 | 10.49 | 1.448 | 0.256 |
| 2014 | 340200 | 0.717 | 3.147 | 0.931 | 16 | 5.952 | 11.75 | 3.256 | 14.02 | 10.64 | 2.087 | 0.208 |
| 2014 | 340300 | 0.718 | 2.251 | 0.633 | 14.54 | 5.916 | 11.02 | 2.957 | 13.55 | 9.939 | 1.476 | 0.133 |
| 2014 | 340400 | 0.698 | 2.184 | 0.779 | 13.77 | 5.495 | 11.28 | 2.633 | 11.72 | 10.18 | 1.672 | 0.118 |
| 2014 | 340500 | 0.729 | 2.655 | 0.875 | 14.74 | 5.426 | 10.9 | 2.872 | 13.89 | 10.84 | 2.713 | 0.163 |
| 2014 | 340600 | 0.697 | 2.01 | 0.633 | 13.56 | 5.372 | 10.46 | 2.589 | 12.72 | 9.954 | 1.992 | 0.107 |
| 2014 | 340700 | 0.717 | 3.004 | 0.993 | 16.12 | 4.301 | 10.43 | 2.446 | 11.7 | 10.77 | 1.969 | 0.167 |
| 2014 | 340800 | 0.717 | 1.559 | 0.328 | 13.81 | 6.431 | 10.64 | 2.836 | 12.01 | 9.785 | 1.696 | 0.089 |
| 2014 | 341000 | 0.737 | 1.835 | 0.526 | 14.7 | 4.995 | 9.95 | 3.047 | 12.05 | 10.07 | 0.937 | 0.163 |
| 2014 | 341100 | 0.715 | 2.02 | 0.462 | 13.85 | 6.108 | 10.75 | 3.796 | 13.25 | 10.03 | 1.547 | 0.11 |
| 2014 | 341200 | 0.714 | 0.768 | 0.237 | 11.99 | 6.958 | 10.46 | 3.167 | 11.52 | 9.038 | 0.936 | 0.126 |
| 2014 | 341300 | 0.697 | 0.913 | 0.198 | 12.76 | 6.465 | 9.898 | 3.298 | 12.8 | 9.391 | 0.853 | 0.094 |
| 2014 | 341500 | 0.714 | 1.366 | 0.321 | 12.99 | 6.58 | 10.63 | 3.052 | 12.28 | 9.257 | 1.177 | 0.12 |
| 2014 | 341600 | 0.715 | 0.871 | 0.143 | 12.33 | 6.453 | 9.387 | 3.684 | 12.81 | 9.337 | 0.902 | 0.106 |
| 2014 | 341700 | 0.722 | 2.102 | 0.684 | 13.88 | 5.079 | 9.953 | 3.222 | 12.13 | 9.661 | 1.384 | 0.228 |
| 2014 | 341800 | 0.738 | 2.303 | 0.121 | 14.83 | 5.634 | 8.653 | 3.309 | 12.96 | 10.17 | 1.296 | 0.154 |
| 2014 | 360100 | 0.705 | 2.22 | 1.69 | 14.24 | 6.249 | 13.23 | 2.406 | 14.5 | 10.71 | 1.651 | 0.139 |
| 2014 | 360200 | 0.716 | 1.36 | 0.681 | 13.43 | 5.123 | 10.17 | 2.786 | 11.46 | 10.31 | 1.766 | 0.151 |
| 2014 | 360300 | 0.709 | 1.005 | 0.363 | 14.23 | 5.289 | 9.328 | 2.763 | 12.06 | 10.12 | 2.5 | 0.169 |
| 2014 | 360400 | 0.721 | 1.094 | 0.722 | 13.74 | 6.24 | 11.33 | 3.145 | 13.7 | 9.94 | 1.838 | 0.178 |
| 2014 | 360500 | 0.725 | 1.88 | 0.936 | 14.25 | 4.806 | 10.39 | 3.188 | 12.27 | 10.39 | 1.791 | 0.207 |
| 2014 | 360600 | 0.709 | 1.649 | 0.265 | 14.41 | 4.843 | 8.706 | 2.577 | 11.79 | 10.05 | 1.64 | 0.229 |
| 2014 | 360700 | 0.718 | 1.12 | 0.469 | 13.37 | 6.861 | 11.38 | 2.454 | 13.53 | 9.491 | 1.088 | 0.168 |
| 2014 | 360800 | 0.708 | 1.071 | 0.214 | 14.04 | 6.267 | 9.854 | 2.93 | 13.09 | 9.539 | 1.619 | 0.176 |
| 2014 | 360900 | 0.707 | 1.059 | 0.341 | 13.83 | 6.39 | 10.71 | 2.656 | 12.81 | 9.659 | 1.436 | 0.193 |
| 2014 | 361000 | 0.705 | 1.09 | 0.273 | 13.5 | 6.058 | 10.27 | 3.02 | 11.95 | 9.596 | 1.666 | 0.177 |
| 2014 | 361100 | 0.717 | 0.63 | 0.205 | 13.21 | 6.65 | 10.1 | 3.054 | 13.15 | 9.423 | 1.264 | 0.194 |
| 2014 | 420100 | 0.723 | 2.996 | 1.467 | 15.74 | 6.718 | 13.78 | 2.638 | 15.15 | 11.12 | 1.144 | 0.166 |
| 2014 | 420200 | 0.701 | 1.364 | 0.6 | 13.53 | 5.58 | 10.52 | 2.898 | 12.73 | 10.45 | 1.757 | 0.086 |
| 2014 | 420300 | 0.689 | 1.399 | 0.564 | 13.85 | 5.849 | 10.81 | 2.767 | 11.62 | 10.03 | 1.735 | 0.092 |
| 2014 | 420500 | 0.715 | 1.881 | 0.638 | 14.33 | 5.992 | 10.99 | 3.061 | 12.16 | 10.58 | 2.037 | 0.141 |
| 2014 | 420600 | 0.682 | 0.323 | 0.311 | 14.96 | 6.389 | 10.87 | 2.866 | 12.77 | 10.23 | 1.862 | 0.127 |
| 2014 | 420700 | 0.696 | 1.082 | 0.454 | 13.18 | 4.702 | 9.602 | 3.316 | 11.77 | 10.55 | 1.917 | 0.089 |
| 2014 | 420800 | 0.701 | 1.269 | 0.28 | 13.75 | 5.705 | 9.924 | 2.855 | 12.11 | 10.45 | 1.692 | 0.057 |
| 2014 | 420900 | 0.692 | 1.152 | 0.295 | 13.4 | 6.265 | 10.58 | 3.121 | 12.16 | 10.02 | 1.481 | 0.082 |
| 2014 | 421000 | 0.702 | 0.918 | 0.63 | 13.29 | 6.49 | 11.62 | 2.682 | 11.22 | 9.879 | 1.716 | 0.06 |
| 2014 | 421100 | 0.693 | 0.782 | 0.424 | 13.52 | 6.609 | 10.74 | 3.439 | 10.89 | 9.738 | 1.935 | 0.066 |
| 2014 | 421200 | 0.708 | 0.954 | 0.402 | 13.63 | 5.692 | 10.6 | 2.562 | 10.49 | 9.928 | 1.782 | 0.106 |
| 2014 | 421300 | 0.709 | 0.914 | 0.211 | 13.3 | 5.549 | 8.724 | 2.286 | 11.02 | 9.902 | 2.057 | 0.062 |
| 2014 | 430100 | 0.713 | 2.866 | 1.371 | 15.02 | 6.509 | 13.21 | 2.537 | 14.71 | 10.74 | 1.018 | 0.171 |
| 2014 | 430200 | 0.706 | 2.188 | 0.68 | 13.92 | 5.982 | 11.4 | 2.935 | 13.13 | 10.44 | 1.507 | 0.113 |
| 2014 | 430300 | 0.702 | 1.974 | 1.232 | 13.69 | 5.675 | 11.72 | 2.895 | 13.14 | 10.36 | 1.042 | 0.151 |
| 2014 | 430400 | 0.707 | 1.048 | 0.595 | 12.42 | 6.674 | 11.56 | 2.549 | 13.22 | 9.888 | 1.005 | 0.101 |
| 2014 | 430500 | 0.695 | 0.76 | 0.225 | 11.91 | 6.708 | 10.22 | 2.669 | 11.64 | 9.41 | 0.845 | 0.074 |
| 2014 | 430600 | 0.71 | 1.167 | 0.43 | 13.35 | 6.334 | 10.79 | 2.583 | 12.22 | 10.31 | 1.488 | 0.065 |
| 2014 | 430700 | 0.714 | 0.991 | 0.368 | 12.84 | 6.411 | 10.64 | 2.964 | 12.83 | 10.16 | 1.358 | 0.073 |
| 2014 | 430800 | 0.737 | 0.633 | 0.26 | 12.28 | 5.148 | 9.386 | 2.848 | 10.81 | 9.719 | 0.534 | 0.129 |
| 2014 | 430900 | 0.703 | 1.102 | 0.378 | 12.97 | 6.18 | 10.36 | 2.6 | 11.76 | 9.714 | 0.732 | 0.066 |
| 2014 | 431000 | 0.724 | 1.014 | 0.27 | 13.66 | 6.252 | 10.08 | 2.738 | 13.49 | 10.01 | 1.112 | 0.185 |
| 2014 | 431100 | 0.714 | 0.917 | 0.252 | 12.52 | 6.447 | 10.12 | 2.787 | 13.04 | 9.65 | 0.741 | 0.105 |
| 2014 | 431200 | 0.699 | 0.583 | 0.26 | 12.37 | 6.264 | 10.51 | 2.076 | 11.11 | 9.679 | 0.704 | 0.104 |
| 2014 | 431300 | 0.716 | 1.09 | 0.513 | 12.26 | 6.098 | 10.25 | 2.227 | 12.1 | 9.723 | 1.354 | 0.074 |
| 2014 | 500000 | 0.707 | 2.076 | 0.668 | 13.94 | 8.124 | 13.52 | 2.458 | 15.69 | 10.17 | 1.278 | 0.186 |
| 2014 | 510100 | 0.717 | 3.289 | 1.174 | 14.55 | 7.099 | 13.5 | 2.693 | 15.69 | 10.98 | 1.067 | 0.139 |
| 2014 | 510300 | 0.7 | 1.341 | 0.587 | 13.79 | 5.799 | 10.69 | 2.574 | 9.52 | 9.921 | 1.318 | 0.055 |
| 2014 | 510400 | 0.724 | 2.298 | 0.634 | 14.42 | 4.718 | 10.28 | 2.512 | 9.52 | 10.83 | 3.208 | 0.099 |
| 2014 | 510500 | 0.705 | 0.914 | 0.421 | 13.12 | 6.232 | 10.71 | 2.463 | 11.13 | 9.618 | 1.169 | 0.136 |
| 2014 | 510600 | 0.713 | 1.663 | 0.669 | 13.73 | 5.973 | 11.11 | 2.539 | 10.56 | 10.28 | 1.496 | 0.062 |
| 2014 | 510700 | 0.707 | 1.847 | 0.765 | 14.15 | 6.308 | 11.69 | 2.563 | 11.75 | 10.12 | 1.088 | 0.067 |
| 2014 | 510800 | 0.712 | 0.687 | 0.155 | 12.9 | 5.737 | 9.593 | 2.628 | 9.719 | 9.291 | 0.908 | 0.087 |
| 2014 | 510900 | 0.711 | 1.256 | 0.195 | 12.23 | 5.941 | 9.548 | 3.267 | 10.45 | 9.524 | 1.06 | 0.066 |
| 2014 | 511000 | 0.702 | 0.776 | 0.377 | 12.25 | 6.054 | 10.19 | 1.992 | 10.52 | 9.636 | 1.605 | 0.059 |
| 2014 | 511100 | 0.717 | 1.063 | 0.614 | 13.15 | 5.874 | 10.79 | 2.646 | 10.69 | 9.901 | 1.889 | 0.095 |
| 2014 | 511300 | 0.715 | 0.67 | 0.455 | 12 | 6.632 | 11.16 | 2.631 | 11.09 | 9.298 | 0.94 | 0.08 |
| 2014 | 511400 | 0.724 | 0.941 | 0.295 | 12.28 | 5.866 | 10.05 | 2.655 | 10.52 | 9.725 | 1.494 | 0.111 |
| 2014 | 511500 | 0.713 | 1.027 | 0.236 | 13.22 | 6.318 | 10.11 | 1.787 | 10.81 | 9.79 | 1.969 | 0.094 |
| 2014 | 511600 | 0.732 | 0.544 | 0.134 | 12.01 | 6.156 | 9.058 | 3.112 | 10.34 | 9.434 | 1.241 | 0.068 |
| 2014 | 511700 | 0.716 | 0.577 | 0.217 | 12.26 | 6.534 | 10.01 | 1.144 | 10.16 | 9.482 | 1.224 | 0.074 |
| 2014 | 511800 | 0.733 | 1.412 | 0.911 | 14.58 | 5.058 | 10.7 | 2.561 | 9.453 | 9.945 | 2.082 | 0.072 |
| 2014 | 511900 | 0.67 | 0.459 | 0.02 | 12.02 | 5.948 | 6.698 | 1.022 | 11.74 | 9.131 | 0.701 | 0.08 |
| 2014 | 512000 | 0.692 | 0.79 | 0.111 | 12.77 | 6.229 | 7.742 | 2.711 | 10.51 | 9.459 | 1.27 | 0.075 |
| 2014 | 520100 | 0.707 | 2.355 | 1.262 | 14.99 | 5.948 | 12.79 | 2.286 | 9.449 | 10.42 | 1.503 | 0.213 |
| 2014 | 520200 | 0.696 | 0.545 | 0.187 | 12.93 | 5.794 | 9.232 | 2.513 | 13.06 | 9.414 | 2.45 | 0.263 |
| 2014 | 520300 | 0.718 | 1.248 | 0.312 | 13.02 | 6.668 | 11.11 | 1.953 | 12.39 | 9.467 | 1.512 | 0.133 |
| 2014 | 520400 | 0.685 | 1.335 | 0.299 | 12.95 | 5.67 | 9.483 | 2.177 | 11.26 | 9.152 | 1.621 | 0.185 |
| 2014 | 530100 | 0.722 | 2.297 | 1.201 | 14.64 | 6.311 | 12.92 | 2.973 | 10.59 | 10.79 | 1.239 | 0.167 |
| 2014 | 530300 | 0.709 | 0.481 | 0.181 | 12.65 | 6.472 | 10.1 | 2.844 | 8.702 | 9.642 | 2.1 | 0.119 |
| 2014 | 530400 | 0.716 | 1.401 | 0.263 | 13.97 | 5.375 | 9.822 | 3.261 | 10.52 | 10.67 | 3.325 | 0.115 |
| 2014 | 530500 | 0.692 | 0.362 | 0.201 | 12.6 | 5.556 | 9.365 | 2.551 | 8.877 | 9.298 | 0.588 | 0.154 |
| 2014 | 530600 | 0.686 | 0.209 | 0.061 | 11.98 | 6.388 | 8.933 | 2.576 | 7.807 | 8.879 | 0.843 | 0.122 |
| 2014 | 530700 | 0.717 | 0.831 | 0.439 | 13.4 | 4.797 | 10.07 | 2.514 | 8.478 | 9.315 | 0.862 | 0.295 |
| 2014 | 530800 | 0.705 | 0.403 | 0.185 | 13.08 | 5.537 | 9.185 | 2.46 | 10.65 | 9.149 | 0.914 | 0.166 |
| 2014 | 530900 | 0.704 | 0.258 | 0.136 | 12.48 | 5.471 | 8.575 | 2.385 | 8.478 | 9.173 | 1.297 | 0.146 |
| 2015 | 310000 | 0.736 | 3.724 | 0.82 | 16.75 | 7.274 | 13.15 | 1.452 | 16.26 | 12.03 | 1.312 | 0.248 |
| 2015 | 320100 | 0.723 | 3.77 | 1.48 | 15.89 | 6.482 | 13.61 | 3.138 | 14.55 | 11.44 | 1.499 | 0.154 |
| 2015 | 320200 | 0.724 | 4.263 | 0.688 | 15.83 | 6.176 | 11.66 | 3.286 | 14.51 | 11.93 | 2.047 | 0.106 |
| 2015 | 320300 | 0.723 | 2.236 | 0.577 | 14.44 | 6.936 | 11.83 | 3.176 | 13.7 | 10.47 | 1.897 | 0.14 |
| 2015 | 320400 | 0.726 | 4.081 | 0.702 | 15.63 | 5.916 | 11.73 | 3.209 | 13.88 | 11.41 | 2.144 | 0.125 |
| 2015 | 320500 | 0.717 | 4.545 | 0.783 | 16.4 | 6.503 | 12.27 | 3.349 | 15.13 | 11.92 | 2.701 | 0.134 |
| 2015 | 320600 | 0.703 | 3.552 | 0.506 | 14.97 | 6.642 | 11.33 | 3.403 | 14.18 | 10.7 | 2.004 | 0.148 |
| 2015 | 320700 | 0.723 | 2.44 | 0.365 | 14.55 | 6.274 | 10.56 | 3.123 | 13.12 | 10.08 | 1.843 | 0.196 |
| 2015 | 320800 | 0.722 | 2.866 | 0.483 | 14.57 | 6.336 | 11.13 | 3.01 | 13.54 | 10.25 | 2.113 | 0.194 |
| 2015 | 320900 | 0.721 | 2.311 | 0.361 | 15.07 | 6.719 | 10.96 | 3.059 | 13.11 | 10.3 | 2.017 | 0.153 |
| 2015 | 321000 | 0.71 | 3.455 | 0.548 | 14.87 | 6.134 | 11.31 | 3.103 | 13.18 | 10.82 | 1.938 | 0.125 |
| 2015 | 321100 | 0.724 | 3.968 | 0.906 | 15.32 | 5.605 | 11.35 | 3.201 | 13.61 | 11.22 | 2.147 | 0.115 |
| 2015 | 321200 | 0.708 | 3.257 | 0.483 | 14.61 | 6.23 | 10.94 | 3.259 | 13.41 | 10.61 | 2.057 | 0.131 |
| 2015 | 321300 | 0.71 | 2.25 | 0.142 | 14.2 | 6.374 | 9.811 | 3.322 | 12.13 | 9.823 | 1.913 | 0.197 |
| 2015 | 330100 | 0.709 | 4.144 | 0.989 | 16.09 | 6.584 | 13.07 | 2.525 | 15.3 | 11.52 | 1.701 | 0.163 |
| 2015 | 330200 | 0.719 | 4.374 | 0.683 | 15.9 | 6.374 | 11.96 | 2.797 | 14.79 | 11.54 | 2.042 | 0.157 |
| 2015 | 330300 | 0.721 | 3.517 | 0.435 | 14.21 | 6.699 | 11.34 | 2.746 | 12.14 | 10.84 | 2.054 | 0.094 |
| 2015 | 330400 | 0.717 | 3.966 | 0.375 | 15.36 | 5.856 | 10.87 | 2.908 | 14.33 | 11.29 | 2.45 | 0.116 |
| 2015 | 330500 | 0.714 | 4.116 | 0.394 | 14.97 | 5.575 | 10.38 | 3.259 | 13.28 | 11.01 | 1.965 | 0.111 |
| 2015 | 330600 | 0.711 | 4.263 | 0.54 | 15.37 | 6.094 | 11.34 | 2.872 | 13.28 | 11.34 | 2.251 | 0.096 |
| 2015 | 330700 | 0.718 | 3.542 | 0.282 | 15.03 | 6.17 | 11.37 | 3.273 | 12.05 | 11.01 | 1.811 | 0.11 |
| 2015 | 330800 | 0.733 | 2.715 | 0.249 | 14.79 | 5.547 | 9.524 | 3.468 | 10.53 | 10.34 | 1.643 | 0.114 |
| 2015 | 330900 | 0.695 | 3.415 | 0.615 | 15.47 | 4.578 | 10.06 | 3.027 | 10.79 | 11.03 | 1.229 | 0.18 |
| 2015 | 331000 | 0.717 | 3.507 | 0.237 | 14.84 | 6.393 | 10.42 | 3.324 | 11.19 | 10.92 | 2.466 | 0.086 |
| 2015 | 331100 | 0.738 | 2.8 | 0.299 | 14.53 | 5.585 | 10.54 | 2.548 | 11.82 | 10.22 | 1.63 | 0.121 |
| 2015 | 340100 | 0.691 | 3.168 | 1.281 | 15.46 | 6.576 | 13.18 | 2.82 | 14.26 | 10.55 | 1.428 | 0.267 |
| 2015 | 340200 | 0.717 | 3.144 | 0.94 | 16.04 | 5.953 | 11.78 | 3.214 | 14.18 | 10.72 | 2.043 | 0.215 |
| 2015 | 340300 | 0.716 | 2.226 | 0.61 | 14.91 | 5.931 | 11.03 | 2.994 | 13.67 | 10.01 | 1.445 | 0.139 |
| 2015 | 340400 | 0.697 | 2.369 | 0.608 | 13.76 | 5.506 | 11.26 | 2.708 | 11.77 | 10.25 | 1.637 | 0.111 |
| 2015 | 340500 | 0.729 | 2.969 | 0.85 | 14.88 | 5.432 | 10.94 | 2.954 | 14 | 10.91 | 2.657 | 0.249 |
| 2015 | 340600 | 0.701 | 1.804 | 0.686 | 13.34 | 5.378 | 10.56 | 2.691 | 12.83 | 10.03 | 1.95 | 0.112 |
| 2015 | 340700 | 0.712 | 2.926 | 0.658 | 16.17 | 4.301 | 10.48 | 2.412 | 11.84 | 10.84 | 1.928 | 0.156 |
| 2015 | 340800 | 0.717 | 1.831 | 0.382 | 13.96 | 6.433 | 10.63 | 2.804 | 11.64 | 9.842 | 1.673 | 0.083 |
| 2015 | 341000 | 0.737 | 2.05 | 0.552 | 14.73 | 4.995 | 10.01 | 3.045 | 11.51 | 10.14 | 0.917 | 0.158 |
| 2015 | 341100 | 0.715 | 2.072 | 0.467 | 14.14 | 6.107 | 10.8 | 3.837 | 13.4 | 10.1 | 1.515 | 0.117 |
| 2015 | 341200 | 0.711 | 1.203 | 0.259 | 12.83 | 6.95 | 10.5 | 3.167 | 11.65 | 9.11 | 0.917 | 0.134 |
| 2015 | 341300 | 0.697 | 1.186 | 0.198 | 12.75 | 6.476 | 10.03 | 3.288 | 12.95 | 9.448 | 0.842 | 0.097 |
| 2015 | 341500 | 0.716 | 1.598 | 0.233 | 13.02 | 6.576 | 10.67 | 3.154 | 12.39 | 9.313 | 1.16 | 0.121 |
| 2015 | 341600 | 0.713 | 1.065 | 0.144 | 13.02 | 6.454 | 9.397 | 3.83 | 12.92 | 9.393 | 0.89 | 0.111 |
| 2015 | 341700 | 0.723 | 2.362 | 0.684 | 13.98 | 5.085 | 10.04 | 3.233 | 12.28 | 9.717 | 1.365 | 0.285 |
| 2015 | 341800 | 0.738 | 2.242 | 0.141 | 14.83 | 5.635 | 8.803 | 3.386 | 13.11 | 10.23 | 1.278 | 0.155 |
| 2015 | 360100 | 0.704 | 2.537 | 1.713 | 14.27 | 6.255 | 13.28 | 2.546 | 14.34 | 10.78 | 1.616 | 0.145 |
| 2015 | 360200 | 0.716 | 1.751 | 0.645 | 14.26 | 5.116 | 10.29 | 2.781 | 11.61 | 10.38 | 1.761 | 0.153 |
| 2015 | 360300 | 0.709 | 1.576 | 0.371 | 14.47 | 5.29 | 9.333 | 2.781 | 12.16 | 10.2 | 2.493 | 0.174 |
| 2015 | 360400 | 0.717 | 1.62 | 0.683 | 13.82 | 6.247 | 11.39 | 3.145 | 13.83 | 10.01 | 1.799 | 0.188 |
| 2015 | 360500 | 0.725 | 2.324 | 0.998 | 14.57 | 4.816 | 10.56 | 3.184 | 12.34 | 10.46 | 1.786 | 0.209 |
| 2015 | 360600 | 0.705 | 2.323 | 0.346 | 14.64 | 4.846 | 8.997 | 2.633 | 11.92 | 10.12 | 1.636 | 0.236 |
| 2015 | 360700 | 0.715 | 1.637 | 0.453 | 13.67 | 6.868 | 11.42 | 2.588 | 13.66 | 9.563 | 1.085 | 0.167 |
| 2015 | 360800 | 0.705 | 1.424 | 0.214 | 14.16 | 6.274 | 9.839 | 2.95 | 13.22 | 9.611 | 1.614 | 0.183 |
| 2015 | 360900 | 0.704 | 1.447 | 0.322 | 14.14 | 6.392 | 10.7 | 2.676 | 12.92 | 9.731 | 1.432 | 0.199 |
| 2015 | 361000 | 0.704 | 1.63 | 0.278 | 14.03 | 5.99 | 10.31 | 3.012 | 12.4 | 9.668 | 1.661 | 0.176 |
| 2015 | 361100 | 0.714 | 1.09 | 0.149 | 13.43 | 6.652 | 10.17 | 3.014 | 13.28 | 9.495 | 1.238 | 0.203 |
| 2015 | 420100 | 0.72 | 3.267 | 1.459 | 15.92 | 6.721 | 13.77 | 2.696 | 15.34 | 11.19 | 1.141 | 0.173 |
| 2015 | 420200 | 0.701 | 1.61 | 0.646 | 13.72 | 5.591 | 10.67 | 2.909 | 11.28 | 10.49 | 1.84 | 0.088 |
| 2015 | 420300 | 0.684 | 1.581 | 0.541 | 13.83 | 5.846 | 10.85 | 2.43 | 11.91 | 10.11 | 1.73 | 0.093 |
| 2015 | 420500 | 0.712 | 2.263 | 0.591 | 15.05 | 5.987 | 10.97 | 3.076 | 12.31 | 10.65 | 2.032 | 0.16 |
| 2015 | 420600 | 0.678 | 0.452 | 0.281 | 15.04 | 6.383 | 10.84 | 2.895 | 13.02 | 10.27 | 1.95 | 0.157 |
| 2015 | 420700 | 0.695 | 1.259 | 0.477 | 13.5 | 4.703 | 9.633 | 2.475 | 11.93 | 10.59 | 2.008 | 0.09 |
| 2015 | 420800 | 0.7 | 1.39 | 0.224 | 14.29 | 5.701 | 9.552 | 2.968 | 12.26 | 10.49 | 1.773 | 0.06 |
| 2015 | 420900 | 0.687 | 1.307 | 0.316 | 14.21 | 6.266 | 10.69 | 3.14 | 12.28 | 10.1 | 1.477 | 0.086 |
| 2015 | 421000 | 0.701 | 1.182 | 0.623 | 13.62 | 6.466 | 11.49 | 2.573 | 11.32 | 9.916 | 1.798 | 0.065 |
| 2015 | 421100 | 0.693 | 0.992 | 0.308 | 13.73 | 6.613 | 10.62 | 3.439 | 11.07 | 9.775 | 2.028 | 0.071 |
| 2015 | 421200 | 0.706 | 1.374 | 0.404 | 13.82 | 5.705 | 10.6 | 2.601 | 10.6 | 10 | 1.778 | 0.11 |
| 2015 | 421300 | 0.709 | 1.054 | 0.204 | 13.66 | 5.525 | 8.704 | 2.292 | 11.17 | 9.938 | 2.155 | 0.067 |
| 2015 | 430100 | 0.711 | 3.077 | 1.468 | 15.08 | 6.523 | 13.25 | 2.514 | 14.82 | 10.82 | 1.004 | 0.178 |
| 2015 | 430200 | 0.704 | 2.395 | 0.755 | 14.46 | 5.999 | 11.32 | 2.982 | 13.28 | 10.47 | 1.579 | 0.175 |
| 2015 | 430300 | 0.704 | 2.073 | 1.222 | 13.73 | 5.667 | 11.71 | 2.91 | 13.26 | 10.44 | 1.027 | 0.152 |
| 2015 | 430400 | 0.707 | 1.246 | 0.759 | 12.48 | 6.684 | 11.6 | 2.298 | 13.38 | 9.966 | 0.991 | 0.105 |
| 2015 | 430500 | 0.695 | 0.984 | 0.248 | 12.32 | 6.711 | 10.25 | 2.849 | 11.69 | 9.488 | 0.833 | 0.11 |
| 2015 | 430600 | 0.71 | 1.3 | 0.372 | 13.38 | 6.336 | 10.69 | 2.612 | 12.33 | 10.34 | 1.558 | 0.144 |
| 2015 | 430700 | 0.713 | 1.247 | 0.4 | 12.86 | 6.412 | 10.71 | 2.855 | 13.04 | 10.2 | 1.422 | 0.074 |
| 2015 | 430800 | 0.736 | 1.061 | 0.467 | 12.1 | 5.136 | 9.442 | 2.995 | 10.96 | 9.755 | 0.559 | 0.132 |
| 2015 | 430900 | 0.704 | 1.388 | 0.396 | 12.92 | 6.175 | 10.39 | 2.595 | 11.83 | 9.792 | 0.722 | 0.07 |
| 2015 | 431000 | 0.722 | 1.19 | 0.29 | 13.67 | 6.27 | 10.1 | 2.675 | 13.65 | 10.09 | 1.097 | 0.158 |
| 2015 | 431100 | 0.712 | 0.952 | 0.273 | 12.74 | 6.454 | 10.16 | 3.015 | 13.16 | 9.728 | 0.731 | 0.08 |
| 2015 | 431200 | 0.7 | 0.852 | 0.294 | 12.41 | 6.25 | 10.53 | 2.114 | 11.17 | 9.715 | 0.737 | 0.106 |
| 2015 | 431300 | 0.715 | 1.159 | 0.375 | 12.42 | 6.104 | 10.23 | 2.317 | 12.28 | 9.759 | 1.418 | 0.069 |
| 2015 | 500000 | 0.702 | 2.498 | 0.698 | 14.12 | 8.123 | 13.55 | 2.489 | 15.72 | 10.25 | 1.26 | 0.188 |
| 2015 | 510100 | 0.707 | 3.585 | 1.126 | 14.97 | 7.113 | 13.54 | 2.682 | 15.72 | 11.06 | 1.053 | 0.145 |
| 2015 | 510300 | 0.702 | 1.507 | 0.602 | 13.87 | 5.791 | 10.52 | 2.56 | 9.541 | 10.01 | 1.281 | 0.054 |
| 2015 | 510400 | 0.718 | 2.651 | 0.595 | 14.24 | 4.706 | 10.08 | 2.444 | 9.541 | 10.91 | 3.164 | 0.079 |
| 2015 | 510500 | 0.702 | 1.024 | 0.399 | 13.45 | 6.226 | 10.7 | 2.469 | 11.04 | 9.707 | 1.136 | 0.139 |
| 2015 | 510600 | 0.714 | 1.753 | 0.763 | 13.42 | 5.966 | 11.2 | 2.556 | 10.69 | 10.36 | 1.475 | 0.061 |
| 2015 | 510700 | 0.706 | 2.175 | 0.81 | 13.66 | 6.302 | 11.73 | 2.565 | 11.75 | 10.21 | 1.057 | 0.064 |
| 2015 | 510800 | 0.711 | 1.2 | 0.207 | 12.74 | 5.721 | 9.784 | 2.618 | 9.253 | 9.369 | 0.896 | 0.094 |
| 2015 | 510900 | 0.711 | 1.279 | 0.209 | 12.31 | 5.937 | 9.559 | 3.368 | 10.07 | 9.613 | 1.03 | 0.075 |
| 2015 | 511000 | 0.7 | 0.986 | 0.388 | 12.89 | 6.041 | 10.2 | 2.096 | 10.72 | 9.713 | 1.583 | 0.061 |
| 2015 | 511100 | 0.712 | 1.173 | 0.593 | 13.17 | 5.869 | 10.8 | 2.481 | 10.7 | 9.979 | 1.863 | 0.095 |
| 2015 | 511300 | 0.712 | 0.825 | 0.471 | 12.39 | 6.61 | 11.18 | 2.645 | 11.18 | 9.375 | 0.927 | 0.082 |
| 2015 | 511400 | 0.722 | 1.125 | 0.307 | 12.28 | 5.856 | 10.06 | 2.86 | 10.47 | 9.814 | 1.452 | 0.114 |
| 2015 | 511500 | 0.711 | 1.345 | 0.238 | 13.25 | 6.314 | 10.14 | 2.089 | 10.72 | 9.868 | 1.942 | 0.095 |
| 2015 | 511600 | 0.727 | 0.696 | 0.135 | 12.25 | 6.147 | 9.058 | 3.143 | 10.44 | 9.512 | 1.224 | 0.078 |
| 2015 | 511700 | 0.713 | 0.782 | 0.196 | 12.37 | 6.526 | 10.05 | 1.267 | 10.44 | 9.571 | 1.19 | 0.075 |
| 2015 | 511800 | 0.731 | 1.58 | 0.972 | 14.27 | 5.043 | 10.75 | 2.58 | 9.644 | 10.03 | 2.024 | 0.073 |
| 2015 | 511900 | 0.666 | 0.55 | 0.027 | 12.28 | 5.939 | 7.777 | 1.03 | 11.38 | 9.209 | 0.691 | 0.087 |
| 2015 | 512000 | 0.693 | 0.892 | 0.138 | 12.66 | 6.222 | 8.422 | 2.792 | 10.82 | 9.548 | 1.234 | 0.077 |
| 2015 | 520100 | 0.706 | 3.041 | 1.248 | 15.13 | 5.971 | 12.82 | 2.297 | 9.43 | 10.51 | 1.461 | 0.217 |
| 2015 | 520200 | 0.696 | 0.839 | 0.195 | 13.81 | 5.809 | 9.313 | 2.781 | 13.27 | 9.503 | 2.381 | 0.243 |
| 2015 | 520300 | 0.717 | 1.263 | 0.317 | 13.08 | 6.676 | 10.91 | 1.947 | 11.79 | 9.556 | 1.469 | 0.133 |
| 2015 | 520400 | 0.681 | 1.33 | 0.309 | 13.3 | 5.692 | 9.537 | 2.607 | 11.48 | 9.241 | 1.575 | 0.198 |
| 2015 | 530100 | 0.718 | 2.532 | 1.183 | 14.77 | 6.32 | 12.99 | 2.558 | 10.83 | 10.86 | 1.221 | 0.162 |
| 2015 | 530300 | 0.71 | 0.763 | 0.164 | 12.82 | 6.474 | 9.943 | 2.845 | 7.997 | 9.712 | 2.069 | 0.112 |
| 2015 | 530400 | 0.713 | 1.631 | 0.266 | 14.08 | 5.375 | 9.611 | 3.105 | 10.61 | 10.74 | 3.276 | 0.117 |
| 2015 | 530500 | 0.689 | 0.796 | 0.21 | 12.81 | 5.557 | 9.422 | 2.495 | 7.347 | 9.369 | 0.58 | 0.157 |
| 2015 | 530600 | 0.687 | 0.313 | 0.069 | 11.74 | 6.396 | 9.163 | 2.609 | 7.997 | 8.95 | 0.83 | 0.122 |
| 2015 | 530700 | 0.716 | 0.983 | 0.402 | 13.52 | 4.795 | 10.09 | 2.361 | 7.625 | 9.386 | 0.85 | 0.283 |
| 2015 | 530800 | 0.705 | 0.673 | 0.202 | 13.17 | 5.521 | 9.265 | 2.252 | 10.4 | 9.219 | 0.9 | 0.161 |
| 2015 | 530900 | 0.704 | 0.443 | 0.135 | 12.54 | 5.461 | 8.575 | 2.395 | 7.625 | 9.262 | 1.261 | 0.138 |
| 2016 | 310000 | 0.734 | 3.828 | 0.911 | 16.98 | 7.279 | 13.15 | 1.475 | 16.32 | 12.1 | 1.293 | 0.255 |
| 2016 | 320100 | 0.723 | 3.804 | 1.49 | 15.9 | 6.497 | 13.63 | 3.151 | 14.65 | 11.51 | 1.477 | 0.159 |
| 2016 | 320200 | 0.725 | 4.185 | 0.674 | 15.85 | 6.186 | 11.64 | 3.281 | 14.63 | 12 | 2.017 | 0.104 |
| 2016 | 320300 | 0.721 | 2.38 | 0.597 | 14.51 | 6.948 | 11.86 | 3.195 | 13.82 | 10.54 | 1.869 | 0.127 |
| 2016 | 320400 | 0.727 | 3.88 | 0.745 | 15.67 | 5.927 | 11.73 | 3.258 | 14.32 | 11.48 | 2.112 | 0.119 |
| 2016 | 320500 | 0.718 | 4.396 | 0.81 | 16.46 | 6.519 | 12.3 | 3.533 | 15.2 | 11.96 | 2.581 | 0.137 |
| 2016 | 320600 | 0.702 | 3.488 | 0.512 | 14.91 | 6.642 | 11.46 | 3.428 | 14.28 | 10.77 | 1.975 | 0.131 |
| 2016 | 320700 | 0.72 | 2.25 | 0.356 | 14.45 | 6.28 | 10.56 | 3.171 | 12.81 | 10.15 | 1.811 | 0.136 |
| 2016 | 320800 | 0.72 | 2.71 | 0.49 | 14.31 | 6.342 | 11.15 | 3.075 | 13.56 | 10.32 | 2.082 | 0.164 |
| 2016 | 320900 | 0.719 | 2.381 | 0.406 | 15.17 | 6.723 | 11.15 | 3.135 | 13.06 | 10.37 | 1.988 | 0.125 |
| 2016 | 321000 | 0.71 | 3.385 | 0.603 | 14.79 | 6.136 | 11.3 | 3.088 | 13.59 | 10.89 | 1.909 | 0.12 |
| 2016 | 321100 | 0.724 | 3.988 | 0.943 | 15.42 | 5.606 | 11.38 | 3.257 | 13.71 | 11.29 | 2.109 | 0.103 |
| 2016 | 321200 | 0.706 | 3.292 | 0.504 | 14.65 | 6.23 | 10.99 | 3.287 | 13.7 | 10.68 | 2.027 | 0.124 |
| 2016 | 321300 | 0.709 | 2.354 | 0.148 | 14.19 | 6.384 | 9.896 | 3.307 | 12.61 | 9.894 | 1.885 | 0.185 |
| 2016 | 330100 | 0.708 | 4.059 | 0.986 | 16.14 | 6.601 | 13.08 | 2.51 | 15.38 | 11.59 | 1.672 | 0.171 |
| 2016 | 330200 | 0.719 | 4.257 | 0.654 | 16.08 | 6.382 | 11.95 | 2.632 | 14.91 | 11.61 | 2.007 | 0.161 |
| 2016 | 330300 | 0.719 | 3.623 | 0.451 | 14.31 | 6.707 | 11.37 | 2.749 | 11.99 | 10.9 | 2.018 | 0.096 |
| 2016 | 330400 | 0.716 | 4.066 | 0.376 | 15.44 | 5.864 | 11.09 | 2.834 | 14.4 | 11.36 | 2.408 | 0.119 |
| 2016 | 330500 | 0.712 | 3.999 | 0.396 | 15.07 | 5.58 | 10.19 | 3.292 | 13.41 | 11.08 | 1.931 | 0.114 |
| 2016 | 330600 | 0.711 | 4.224 | 0.55 | 15.45 | 6.098 | 11.41 | 2.885 | 13.18 | 11.41 | 2.211 | 0.096 |
| 2016 | 330700 | 0.717 | 3.588 | 0.316 | 15.15 | 6.176 | 11.35 | 3.129 | 12.34 | 11.08 | 1.78 | 0.112 |
| 2016 | 330800 | 0.732 | 2.801 | 0.265 | 14.9 | 5.549 | 9.512 | 3.517 | 10.61 | 10.41 | 1.614 | 0.116 |
| 2016 | 330900 | 0.694 | 3.022 | 0.626 | 15.62 | 4.575 | 10.11 | 2.953 | 11.85 | 11.1 | 1.208 | 0.179 |
| 2016 | 331000 | 0.717 | 3.558 | 0.242 | 14.47 | 6.397 | 10.44 | 3.337 | 12.32 | 10.99 | 2.423 | 0.092 |
| 2016 | 331100 | 0.735 | 2.968 | 0.306 | 14.68 | 5.591 | 10.51 | 2.573 | 11.9 | 10.29 | 1.601 | 0.124 |
| 2016 | 340100 | 0.69 | 3.297 | 1.266 | 16.45 | 6.593 | 13.12 | 2.836 | 14.45 | 10.61 | 1.403 | 0.264 |
| 2016 | 340200 | 0.717 | 3.304 | 0.959 | 16.41 | 5.961 | 12.01 | 3.271 | 14.33 | 10.79 | 2.012 | 0.223 |
| 2016 | 340300 | 0.713 | 2.261 | 0.618 | 15.07 | 5.94 | 11.02 | 3.002 | 13.81 | 10.08 | 1.423 | 0.143 |
| 2016 | 340400 | 0.699 | 1.892 | 0.676 | 13.53 | 5.964 | 11.29 | 2.736 | 11.91 | 10.33 | 1.612 | 0.128 |
| 2016 | 340500 | 0.731 | 3.009 | 0.849 | 15.32 | 5.434 | 10.89 | 2.952 | 14.15 | 10.98 | 2.615 | 0.16 |
| 2016 | 340600 | 0.701 | 1.683 | 0.648 | 13 | 5.38 | 10.59 | 2.828 | 12.97 | 10.1 | 1.92 | 0.101 |
| 2016 | 340700 | 0.711 | 2.125 | 0.655 | 15.4 | 5.142 | 10.5 | 2.492 | 11.99 | 10.91 | 1.898 | 0.171 |
| 2016 | 340800 | 0.715 | 2.137 | 1.745 | 14.21 | 6.271 | 10.6 | 2.928 | 11.7 | 9.91 | 1.644 | 0.091 |
| 2016 | 341000 | 0.735 | 1.971 | 0.541 | 14.8 | 4.997 | 10.02 | 3.126 | 11.58 | 10.21 | 0.903 | 0.154 |
| 2016 | 341100 | 0.714 | 2.19 | 0.509 | 14.35 | 6.118 | 10.85 | 3.782 | 13.54 | 10.18 | 1.491 | 0.125 |
| 2016 | 341200 | 0.708 | 1.297 | 0.273 | 13.06 | 6.968 | 10.52 | 3.267 | 11.81 | 9.183 | 0.903 | 0.137 |
| 2016 | 341300 | 0.696 | 1.035 | 0.199 | 13.91 | 6.483 | 10.08 | 3.329 | 13.09 | 9.52 | 0.829 | 0.099 |
| 2016 | 341500 | 0.717 | 1.913 | 0.241 | 13.56 | 6.375 | 10.62 | 3.188 | 12.44 | 9.382 | 1.14 | 0.106 |
| 2016 | 341600 | 0.711 | 1.152 | 0.143 | 12.93 | 6.472 | 9.325 | 3.818 | 13.08 | 9.462 | 0.874 | 0.107 |
| 2016 | 341700 | 0.723 | 2.236 | 0.67 | 13.91 | 5.088 | 10.09 | 3.245 | 12.39 | 9.789 | 1.344 | 0.204 |
| 2016 | 341800 | 0.737 | 2.222 | 0.147 | 14.99 | 5.635 | 8.825 | 3.431 | 13.26 | 10.29 | 1.256 | 0.152 |
| 2016 | 360100 | 0.702 | 2.845 | 1.945 | 14.48 | 6.26 | 13.32 | 2.491 | 14.53 | 10.85 | 1.591 | 0.137 |
| 2016 | 360200 | 0.714 | 1.818 | 0.681 | 13.92 | 5.13 | 10.38 | 3.339 | 11.73 | 10.45 | 1.726 | 0.138 |
| 2016 | 360300 | 0.708 | 2.013 | 0.391 | 14.62 | 5.298 | 9.362 | 2.761 | 12.32 | 10.27 | 2.443 | 0.16 |
| 2016 | 360400 | 0.716 | 1.898 | 0.728 | 13.94 | 6.254 | 11.43 | 3.121 | 14 | 10.08 | 1.771 | 0.182 |
| 2016 | 360500 | 0.725 | 2.592 | 1.02 | 14.58 | 4.82 | 10.59 | 3.176 | 12.49 | 10.53 | 1.758 | 0.187 |
| 2016 | 360600 | 0.705 | 2.804 | 0.346 | 14.62 | 4.852 | 9.06 | 2.871 | 12.08 | 10.19 | 1.603 | 0.216 |
| 2016 | 360700 | 0.712 | 1.86 | 0.451 | 14.08 | 6.878 | 11.83 | 2.699 | 13.82 | 9.636 | 1.064 | 0.153 |
| 2016 | 360800 | 0.704 | 1.994 | 0.273 | 14.14 | 6.282 | 10.46 | 2.933 | 13.38 | 9.683 | 1.589 | 0.164 |
| 2016 | 360900 | 0.702 | 1.3 | 0.325 | 14.31 | 6.4 | 10.67 | 2.747 | 13.06 | 9.804 | 1.41 | 0.188 |
| 2016 | 361000 | 0.704 | 1.721 | 0.279 | 14.14 | 5.994 | 10.28 | 2.976 | 12.55 | 9.74 | 1.635 | 0.158 |
| 2016 | 361100 | 0.713 | 1.196 | 0.18 | 13.32 | 6.662 | 10.27 | 3.041 | 13.44 | 9.567 | 1.218 | 0.192 |
| 2016 | 420100 | 0.719 | 3.357 | 1.466 | 16.15 | 6.726 | 13.76 | 2.667 | 15.55 | 11.26 | 1.118 | 0.17 |
| 2016 | 420200 | 0.7 | 1.746 | 0.798 | 14.23 | 5.598 | 10.65 | 2.995 | 11.48 | 10.56 | 1.804 | 0.086 |
| 2016 | 420300 | 0.681 | 1.691 | 0.513 | 13.81 | 5.852 | 10.87 | 2.429 | 12.12 | 10.18 | 1.696 | 0.092 |
| 2016 | 420500 | 0.71 | 2.361 | 0.573 | 15.06 | 5.976 | 10.97 | 3.104 | 12.47 | 10.73 | 1.991 | 0.133 |
| 2016 | 420600 | 0.678 | 0.441 | 0.262 | 15.06 | 6.387 | 10.82 | 2.868 | 13.21 | 10.34 | 1.911 | 0.138 |
| 2016 | 420700 | 0.694 | 1.561 | 0.481 | 14.81 | 4.71 | 9.764 | 2.481 | 12.1 | 10.66 | 1.968 | 0.093 |
| 2016 | 420800 | 0.698 | 1.627 | 0.224 | 14.48 | 5.704 | 9.447 | 3.024 | 12.43 | 10.56 | 1.737 | 0.063 |
| 2016 | 420900 | 0.684 | 1.488 | 0.309 | 14.42 | 6.26 | 10.69 | 3.144 | 12.42 | 10.17 | 1.447 | 0.084 |
| 2016 | 421000 | 0.7 | 1.412 | 0.646 | 14.12 | 6.471 | 11.43 | 2.5 | 11.47 | 9.988 | 1.762 | 0.067 |
| 2016 | 421100 | 0.691 | 1.05 | 0.296 | 13.86 | 6.616 | 10.61 | 3.434 | 11.36 | 9.847 | 1.987 | 0.069 |
| 2016 | 421200 | 0.705 | 1.532 | 0.412 | 14.02 | 5.717 | 10.62 | 2.645 | 10.75 | 10.07 | 1.742 | 0.106 |
| 2016 | 421300 | 0.707 | 1.315 | 0.2 | 13.52 | 5.529 | 8.718 | 2.322 | 11.36 | 10.01 | 2.112 | 0.065 |
| 2016 | 430100 | 0.712 | 3.119 | 1.459 | 15.08 | 6.545 | 13.29 | 2.732 | 14.98 | 10.89 | 0.985 | 0.171 |
| 2016 | 430200 | 0.703 | 2.325 | 0.882 | 14.31 | 6.001 | 11.45 | 2.972 | 13.46 | 10.55 | 1.55 | 0.175 |
| 2016 | 430300 | 0.701 | 2.235 | 1.206 | 13.95 | 5.67 | 11.77 | 2.909 | 13.46 | 10.51 | 1.008 | 0.151 |
| 2016 | 430400 | 0.705 | 1.338 | 0.768 | 12.5 | 6.683 | 11.68 | 2.552 | 13.54 | 10.04 | 0.973 | 0.102 |
| 2016 | 430500 | 0.695 | 1.042 | 0.249 | 12.47 | 6.721 | 10.28 | 2.892 | 11.92 | 9.56 | 0.818 | 0.11 |
| 2016 | 430600 | 0.709 | 1.319 | 0.382 | 13.77 | 6.347 | 10.72 | 2.624 | 12.53 | 10.42 | 1.527 | 0.145 |
| 2016 | 430700 | 0.71 | 1.162 | 0.397 | 12.85 | 6.415 | 10.76 | 2.31 | 13.29 | 10.27 | 1.396 | 0.073 |
| 2016 | 430800 | 0.733 | 0.945 | 0.675 | 12.56 | 5.142 | 9.973 | 3.045 | 11.12 | 9.827 | 0.549 | 0.132 |
| 2016 | 430900 | 0.702 | 1.422 | 0.384 | 13.03 | 6.182 | 10.45 | 2.531 | 11.98 | 9.864 | 0.709 | 0.064 |
| 2016 | 431000 | 0.719 | 1.044 | 0.297 | 13.73 | 6.282 | 10.17 | 1.829 | 13.83 | 10.16 | 1.077 | 0.157 |
| 2016 | 431100 | 0.71 | 1.006 | 0.266 | 12.82 | 6.469 | 10.23 | 2.975 | 13.36 | 9.8 | 0.718 | 0.082 |
| 2016 | 431200 | 0.699 | 0.802 | 0.297 | 12.82 | 6.26 | 10.28 | 2.082 | 10.21 | 9.788 | 0.724 | 0.109 |
| 2016 | 431300 | 0.712 | 1.205 | 0.41 | 12.16 | 6.116 | 10.27 | 2.322 | 12.49 | 9.832 | 1.39 | 0.073 |
| 2016 | 500000 | 0.711 | 2.612 | 0.699 | 14.24 | 8.129 | 13.5 | 2.504 | 15.83 | 10.32 | 1.237 | 0.176 |
| 2016 | 510100 | 0.705 | 3.441 | 1.124 | 15.01 | 7.244 | 13.58 | 2.631 | 15.83 | 11.12 | 1.05 | 0.137 |
| 2016 | 510300 | 0.702 | 1.525 | 0.619 | 13.25 | 5.79 | 10.45 | 2.66 | 8.831 | 10.08 | 1.277 | 0.055 |
| 2016 | 510400 | 0.718 | 2.713 | 0.636 | 14.06 | 4.71 | 10.13 | 2.516 | 8.831 | 10.97 | 3.155 | 0.078 |
| 2016 | 510500 | 0.7 | 1.15 | 0.405 | 13.52 | 6.23 | 10.75 | 2.421 | 10.4 | 9.774 | 1.133 | 0.14 |
| 2016 | 510600 | 0.713 | 2.049 | 0.804 | 13.19 | 5.971 | 11.39 | 2.465 | 10.91 | 10.42 | 1.471 | 0.064 |
| 2016 | 510700 | 0.705 | 2.309 | 0.85 | 13.74 | 6.301 | 11.77 | 2.614 | 11.3 | 10.27 | 1.055 | 0.062 |
| 2016 | 510800 | 0.711 | 1.232 | 0.24 | 12.83 | 5.72 | 9.926 | 2.597 | 8.878 | 9.441 | 0.879 | 0.087 |
| 2016 | 510900 | 0.701 | 1.181 | 0.191 | 12.48 | 5.935 | 9.534 | 3.404 | 9.32 | 9.68 | 1.027 | 0.077 |
| 2016 | 511000 | 0.7 | 0.945 | 0.378 | 12.51 | 6.04 | 10.28 | 2.131 | 10.52 | 9.786 | 1.554 | 0.061 |
| 2016 | 511100 | 0.713 | 1.387 | 0.609 | 12.77 | 5.872 | 10.64 | 2.414 | 10.35 | 10.05 | 1.829 | 0.096 |
| 2016 | 511300 | 0.711 | 0.84 | 0.487 | 12.25 | 6.608 | 11.2 | 2.652 | 10.41 | 9.448 | 0.91 | 0.085 |
| 2016 | 511400 | 0.718 | 1.185 | 0.342 | 12.28 | 5.858 | 10.08 | 2.851 | 10.67 | 9.881 | 1.448 | 0.114 |
| 2016 | 511500 | 0.71 | 1.242 | 0.236 | 13.13 | 6.321 | 10.15 | 2.297 | 10.45 | 9.94 | 1.907 | 0.096 |
| 2016 | 511600 | 0.727 | 0.722 | 0.142 | 12.09 | 6.146 | 9.123 | 3.096 | 10.22 | 9.578 | 1.221 | 0.082 |
| 2016 | 511700 | 0.71 | 0.775 | 0.177 | 12.37 | 6.528 | 10.07 | 1.374 | 10.41 | 9.637 | 1.187 | 0.075 |
| 2016 | 511800 | 0.729 | 1.655 | 0.944 | 13.32 | 5.043 | 10.78 | 2.602 | 9.481 | 10.1 | 2.018 | 0.072 |
| 2016 | 511900 | 0.667 | 0.698 | 0.047 | 12.35 | 5.927 | 8.199 | 1.322 | 11.2 | 9.281 | 0.679 | 0.091 |
| 2016 | 512000 | 0.7 | 1.075 | 0.103 | 12.48 | 5.872 | 8.791 | 2.958 | 10.43 | 9.614 | 1.231 | 0.055 |
| 2016 | 520100 | 0.705 | 2.561 | 1.294 | 15.28 | 5.994 | 12.91 | 2.293 | 8.191 | 10.57 | 1.457 | 0.195 |
| 2016 | 520200 | 0.697 | 1.019 | 0.201 | 14.18 | 5.829 | 9.52 | 2.461 | 13.52 | 9.57 | 2.375 | 0.227 |
| 2016 | 520300 | 0.714 | 1.182 | 0.337 | 13.54 | 6.687 | 11.37 | 2.465 | 12.24 | 9.622 | 1.465 | 0.128 |
| 2016 | 520400 | 0.68 | 1.015 | 0.3 | 13.38 | 5.704 | 9.66 | 2.937 | 12.4 | 9.307 | 1.571 | 0.182 |
| 2016 | 530100 | 0.717 | 2.66 | 1.254 | 14.81 | 6.328 | 13.05 | 2.736 | 9.356 | 10.94 | 1.202 | 0.157 |
| 2016 | 530300 | 0.709 | 0.737 | 0.187 | 12.88 | 6.482 | 10.21 | 2.949 | 6.324 | 9.787 | 2.037 | 0.111 |
| 2016 | 530400 | 0.71 | 1.654 | 0.27 | 14.17 | 5.38 | 9.661 | 2.867 | 10.76 | 10.82 | 3.225 | 0.113 |
| 2016 | 530500 | 0.687 | 0.854 | 0.219 | 12.81 | 5.565 | 9.466 | 2.488 | 7.535 | 9.444 | 0.571 | 0.159 |
| 2016 | 530600 | 0.687 | 0.256 | 0.073 | 11.8 | 6.412 | 9.331 | 2.617 | 6.324 | 9.025 | 0.818 | 0.121 |
| 2016 | 530700 | 0.716 | 1.401 | 0.396 | 13.67 | 4.804 | 10.13 | 2.389 | 6.499 | 9.461 | 0.837 | 0.266 |
| 2016 | 530800 | 0.705 | 0.701 | 0.17 | 13.23 | 5.525 | 9.274 | 2.416 | 8.897 | 9.294 | 0.886 | 0.157 |
| 2016 | 530900 | 0.7 | 0.396 | 0.131 | 12.34 | 5.468 | 8.876 | 2.629 | 6.499 | 9.337 | 1.241 | 0.128 |
| 2017 | 310000 | 0.733 | 3.928 | 0.905 | 17.1 | 7.283 | 13.15 | 1.506 | 16.26 | 12.17 | 1.273 | 0.259 |
| 2017 | 320100 | 0.722 | 3.873 | 1.513 | 16.11 | 6.524 | 13.62 | 3.168 | 14.72 | 11.58 | 1.454 | 0.165 |
| 2017 | 320200 | 0.724 | 4.085 | 0.683 | 15.96 | 6.201 | 11.63 | 3.316 | 14.72 | 12.07 | 1.998 | 0.103 |
| 2017 | 320300 | 0.719 | 2.405 | 0.616 | 14.55 | 6.946 | 11.77 | 3.132 | 13.93 | 10.6 | 1.852 | 0.116 |
| 2017 | 320400 | 0.727 | 3.792 | 0.735 | 15.71 | 5.938 | 11.72 | 3.268 | 14.22 | 11.56 | 2.079 | 0.12 |
| 2017 | 320500 | 0.718 | 4.355 | 0.791 | 16.7 | 6.538 | 12.25 | 3.491 | 14.93 | 12.03 | 2.557 | 0.141 |
| 2017 | 320600 | 0.702 | 3.256 | 0.552 | 15.09 | 6.639 | 11.41 | 3.461 | 14.31 | 10.84 | 1.944 | 0.122 |
| 2017 | 320700 | 0.719 | 2.549 | 0.37 | 14.32 | 6.279 | 10.59 | 3.199 | 13.08 | 10.22 | 1.794 | 0.13 |
| 2017 | 320800 | 0.719 | 2.641 | 0.53 | 14.37 | 6.33 | 11.16 | 3.186 | 13.59 | 10.39 | 2.063 | 0.115 |
| 2017 | 320900 | 0.717 | 2.568 | 0.413 | 14.92 | 6.717 | 11.04 | 3.173 | 13.19 | 10.44 | 1.969 | 0.102 |
| 2017 | 321000 | 0.709 | 3.46 | 0.677 | 15 | 6.131 | 11.23 | 3.072 | 13.61 | 10.96 | 1.892 | 0.104 |
| 2017 | 321100 | 0.728 | 4.015 | 0.967 | 15.36 | 5.602 | 11.29 | 3.28 | 13.72 | 11.35 | 2.09 | 0.094 |
| 2017 | 321200 | 0.704 | 3.014 | 0.52 | 14.78 | 6.225 | 11.03 | 3.346 | 13.95 | 10.75 | 2.009 | 0.121 |
| 2017 | 321300 | 0.708 | 2.126 | 0.15 | 14.05 | 6.382 | 9.952 | 3.268 | 12.41 | 9.969 | 1.856 | 0.149 |
| 2017 | 330100 | 0.708 | 4.041 | 0.98 | 16.32 | 6.625 | 13.09 | 2.566 | 15.31 | 11.66 | 1.656 | 0.177 |
| 2017 | 330200 | 0.717 | 4.139 | 0.672 | 16.1 | 6.392 | 11.96 | 2.523 | 14.82 | 11.67 | 1.988 | 0.166 |
| 2017 | 330300 | 0.715 | 3.6 | 0.432 | 14.46 | 6.715 | 11.39 | 2.752 | 12.4 | 10.97 | 1.999 | 0.094 |
| 2017 | 330400 | 0.715 | 3.951 | 0.445 | 15.55 | 5.875 | 11.14 | 2.923 | 14.52 | 11.43 | 2.385 | 0.126 |
| 2017 | 330500 | 0.711 | 3.836 | 0.417 | 15.26 | 5.583 | 10.18 | 3.33 | 13.47 | 11.14 | 1.913 | 0.118 |
| 2017 | 330600 | 0.712 | 4.068 | 0.569 | 15.58 | 6.1 | 11.49 | 2.879 | 13.67 | 11.48 | 2.191 | 0.098 |
| 2017 | 330700 | 0.719 | 3.601 | 0.52 | 15.16 | 6.186 | 11.35 | 3.234 | 12.58 | 11.15 | 1.741 | 0.11 |
| 2017 | 330800 | 0.731 | 2.98 | 0.259 | 15.07 | 5.553 | 9.682 | 3.526 | 10.82 | 10.48 | 1.579 | 0.117 |
| 2017 | 330900 | 0.713 | 3.029 | 0.652 | 15.56 | 4.575 | 10 | 3.071 | 12.52 | 11.17 | 1.197 | 0.174 |
| 2017 | 331000 | 0.715 | 3.48 | 0.249 | 14.64 | 6.404 | 10.46 | 3.259 | 12.61 | 11.05 | 2.401 | 0.095 |
| 2017 | 331100 | 0.731 | 3.092 | 0.312 | 14.81 | 5.595 | 10.53 | 2.73 | 11.89 | 10.36 | 1.587 | 0.125 |
| 2017 | 340100 | 0.69 | 3.399 | 1.307 | 16.05 | 6.611 | 13.13 | 2.889 | 14.53 | 10.69 | 1.372 | 0.26 |
| 2017 | 340200 | 0.718 | 3.168 | 0.962 | 16.51 | 5.961 | 12.02 | 3.212 | 14.41 | 10.88 | 1.92 | 0.215 |
| 2017 | 340300 | 0.713 | 1.916 | 0.628 | 15.02 | 5.943 | 11.02 | 3.03 | 13.9 | 10.17 | 1.358 | 0.139 |
| 2017 | 340400 | 0.699 | 1.928 | 0.679 | 13.72 | 5.966 | 11.05 | 2.801 | 11.99 | 10.4 | 1.576 | 0.123 |
| 2017 | 340500 | 0.731 | 2.914 | 0.844 | 15.5 | 5.434 | 10.89 | 2.992 | 14.25 | 11.07 | 2.496 | 0.147 |
| 2017 | 340600 | 0.7 | 1.696 | 0.697 | 13.5 | 5.38 | 10.59 | 2.852 | 13.04 | 10.17 | 1.878 | 0.096 |
| 2017 | 340700 | 0.712 | 1.846 | 0.683 | 15.4 | 5.142 | 10.49 | 2.645 | 12.11 | 11 | 1.812 | 0.153 |
| 2017 | 340800 | 0.713 | 1.778 | 0.383 | 14.41 | 6.275 | 10.53 | 2.791 | 11.78 | 9.982 | 1.608 | 0.08 |
| 2017 | 341000 | 0.736 | 1.889 | 0.565 | 15.11 | 4.997 | 10.04 | 3.128 | 11.67 | 10.3 | 0.862 | 0.142 |
| 2017 | 341100 | 0.716 | 2.06 | 0.554 | 14.66 | 6.118 | 10.89 | 3.719 | 13.62 | 10.27 | 1.423 | 0.125 |
| 2017 | 341200 | 0.705 | 1.163 | 0.273 | 13.11 | 6.975 | 10.53 | 3.224 | 11.91 | 9.272 | 0.862 | 0.148 |
| 2017 | 341300 | 0.695 | 0.838 | 0.195 | 13.31 | 6.486 | 10.25 | 3.45 | 13.18 | 9.591 | 0.81 | 0.095 |
| 2017 | 341500 | 0.718 | 1.712 | 0.225 | 14.28 | 6.377 | 10.57 | 3.222 | 12.6 | 9.453 | 1.115 | 0.112 |
| 2017 | 341600 | 0.709 | 1.09 | 0.15 | 13.62 | 6.479 | 9.328 | 3.548 | 13.18 | 9.533 | 0.855 | 0.107 |
| 2017 | 341700 | 0.724 | 2.361 | 0.664 | 13.58 | 5.088 | 10.15 | 3.243 | 12.48 | 9.861 | 1.314 | 0.174 |
| 2017 | 341800 | 0.734 | 2.072 | 0.159 | 15.2 | 5.635 | 8.888 | 3.482 | 13.34 | 10.37 | 1.228 | 0.144 |
| 2017 | 360100 | 0.702 | 2.811 | 1.666 | 15.24 | 6.263 | 13.32 | 2.518 | 14.77 | 10.94 | 1.519 | 0.131 |
| 2017 | 360200 | 0.712 | 2.18 | 0.81 | 14.67 | 5.13 | 10.38 | 3.191 | 11.84 | 10.53 | 1.676 | 0.125 |
| 2017 | 360300 | 0.712 | 2.327 | 0.49 | 14.81 | 5.298 | 10.22 | 2.781 | 12.42 | 10.35 | 2.372 | 0.144 |
| 2017 | 360400 | 0.713 | 1.897 | 0.664 | 14.19 | 6.254 | 11.44 | 3.091 | 14.11 | 10.17 | 1.691 | 0.17 |
| 2017 | 360500 | 0.728 | 2.156 | 1.218 | 14.56 | 4.804 | 10.54 | 3.183 | 12.59 | 10.61 | 1.707 | 0.168 |
| 2017 | 360600 | 0.702 | 1.926 | 0.332 | 15.22 | 4.852 | 9.121 | 2.809 | 12.18 | 10.27 | 1.556 | 0.185 |
| 2017 | 360700 | 0.71 | 1.943 | 0.434 | 14.45 | 6.881 | 11.46 | 2.766 | 13.93 | 9.712 | 1.033 | 0.143 |
| 2017 | 360800 | 0.702 | 1.849 | 0.268 | 14.3 | 6.284 | 10.43 | 3.028 | 13.49 | 9.773 | 1.517 | 0.151 |
| 2017 | 360900 | 0.701 | 1.697 | 0.336 | 14.82 | 6.4 | 10.73 | 2.755 | 13.16 | 9.88 | 1.369 | 0.178 |
| 2017 | 361000 | 0.703 | 2.009 | 0.287 | 14.41 | 6.066 | 10.29 | 3.05 | 12.38 | 9.816 | 1.588 | 0.143 |
| 2017 | 361100 | 0.711 | 1.412 | 0.215 | 13.54 | 6.663 | 10.36 | 3.134 | 13.55 | 9.657 | 1.163 | 0.168 |
| 2017 | 420100 | 0.717 | 3.425 | 1.471 | 16.42 | 6.75 | 13.76 | 2.478 | 15.69 | 11.34 | 1.086 | 0.167 |
| 2017 | 420200 | 0.699 | 1.808 | 0.608 | 13.96 | 5.602 | 10.68 | 2.99 | 11.58 | 10.64 | 1.756 | 0.084 |
| 2017 | 420300 | 0.679 | 1.731 | 0.455 | 14.03 | 5.846 | 10.85 | 2.514 | 12.24 | 10.26 | 1.646 | 0.092 |
| 2017 | 420500 | 0.711 | 2.468 | 0.612 | 15.11 | 5.971 | 10.95 | 3.104 | 12.06 | 10.8 | 1.933 | 0.101 |
| 2017 | 420600 | 0.678 | 0.412 | 0.276 | 15.17 | 6.384 | 10.83 | 2.876 | 13.33 | 10.41 | 1.861 | 0.127 |
| 2017 | 420700 | 0.693 | 1.567 | 0.456 | 15.18 | 4.71 | 9.687 | 2.487 | 12.19 | 10.74 | 1.916 | 0.096 |
| 2017 | 420800 | 0.697 | 1.748 | 0.229 | 14.62 | 5.684 | 9.514 | 3.05 | 12.55 | 10.63 | 1.696 | 0.065 |
| 2017 | 420900 | 0.684 | 1.638 | 0.28 | 14.53 | 6.252 | 10.44 | 3.182 | 12.27 | 10.25 | 1.405 | 0.08 |
| 2017 | 421000 | 0.699 | 1.417 | 0.621 | 14.22 | 6.465 | 11.47 | 2.506 | 9.909 | 10.06 | 1.71 | 0.066 |
| 2017 | 421100 | 0.689 | 1.091 | 0.331 | 13.94 | 6.607 | 10.68 | 3.434 | 10.23 | 9.922 | 1.935 | 0.072 |
| 2017 | 421200 | 0.706 | 1.707 | 0.421 | 14.04 | 5.717 | 10.35 | 3.42 | 9.973 | 10.15 | 1.691 | 0.104 |
| 2017 | 421300 | 0.709 | 1.178 | 0.197 | 13.31 | 5.521 | 8.718 | 2.462 | 11.46 | 10.09 | 2.056 | 0.065 |
| 2017 | 430100 | 0.711 | 3.22 | 1.443 | 15.24 | 6.564 | 13.32 | 2.21 | 15.08 | 10.98 | 0.95 | 0.171 |
| 2017 | 430200 | 0.701 | 2.323 | 0.869 | 14.82 | 5.999 | 11.4 | 3.043 | 13.54 | 10.62 | 1.509 | 0.119 |
| 2017 | 430300 | 0.704 | 2.19 | 1.223 | 14.7 | 5.663 | 11.8 | 3.343 | 13.62 | 10.59 | 0.982 | 0.097 |
| 2017 | 430400 | 0.704 | 1.448 | 0.679 | 12.91 | 6.685 | 11.64 | 2.202 | 13.67 | 10.12 | 0.938 | 0.076 |
| 2017 | 430500 | 0.694 | 1.121 | 0.295 | 12.25 | 6.717 | 10.34 | 2.986 | 12.1 | 9.641 | 0.789 | 0.071 |
| 2017 | 430600 | 0.708 | 1.397 | 0.43 | 13.78 | 6.34 | 10.79 | 2.839 | 12.69 | 10.49 | 1.487 | 0.064 |
| 2017 | 430700 | 0.708 | 1.266 | 0.416 | 13.71 | 6.407 | 10.82 | 2.533 | 13.49 | 10.35 | 1.359 | 0.07 |
| 2017 | 430800 | 0.73 | 1.12 | 0.484 | 12.84 | 5.136 | 9.534 | 3.002 | 11.29 | 9.902 | 0.534 | 0.086 |
| 2017 | 430900 | 0.701 | 1.425 | 0.44 | 13.19 | 6.172 | 10.53 | 2.609 | 12.13 | 9.945 | 0.683 | 0.061 |
| 2017 | 431000 | 0.718 | 1.134 | 0.303 | 13.82 | 6.28 | 10.24 | 1.828 | 13.96 | 10.24 | 1.038 | 0.084 |
| 2017 | 431100 | 0.712 | 1.063 | 0.265 | 13.39 | 6.465 | 10.26 | 3.034 | 13.5 | 9.875 | 0.699 | 0.081 |
| 2017 | 431200 | 0.698 | 0.982 | 0.32 | 13.64 | 6.258 | 10.32 | 2.079 | 10.44 | 9.863 | 0.705 | 0.068 |
| 2017 | 431300 | 0.711 | 1.238 | 0.42 | 12.48 | 6.118 | 10.28 | 3.148 | 12.65 | 9.907 | 1.353 | 0.07 |
| 2017 | 500000 | 0.71 | 2.414 | 0.714 | 14.37 | 8.129 | 13.52 | 2.539 | 15.74 | 10.4 | 1.192 | 0.164 |
| 2017 | 510100 | 0.703 | 3.388 | 1.071 | 15.13 | 7.269 | 13.61 | 2.643 | 15.74 | 11.2 | 1.02 | 0.138 |
| 2017 | 510300 | 0.701 | 1.503 | 0.577 | 13.29 | 5.781 | 10.5 | 2.684 | 8.124 | 10.15 | 1.241 | 0.055 |
| 2017 | 510400 | 0.716 | 2.562 | 0.594 | 14.03 | 4.691 | 10.17 | 2.487 | 8.124 | 11.05 | 3.066 | 0.077 |
| 2017 | 510500 | 0.701 | 1.327 | 0.409 | 13.33 | 6.234 | 10.76 | 2.35 | 11.01 | 9.847 | 1.101 | 0.136 |
| 2017 | 510600 | 0.712 | 2.225 | 0.844 | 13.21 | 5.961 | 11.6 | 2.695 | 11.34 | 10.5 | 1.429 | 0.063 |
| 2017 | 510700 | 0.705 | 2.285 | 0.789 | 13.95 | 6.286 | 11.81 | 2.905 | 10.93 | 10.35 | 1.025 | 0.059 |
| 2017 | 510800 | 0.71 | 1.325 | 0.214 | 12.69 | 5.714 | 9.999 | 2.591 | 9.911 | 9.522 | 0.847 | 0.088 |
| 2017 | 510900 | 0.706 | 1.297 | 0.225 | 12.46 | 5.914 | 9.524 | 2.957 | 10.08 | 9.753 | 0.998 | 0.079 |
| 2017 | 511000 | 0.701 | 1.004 | 0.367 | 12.92 | 6.028 | 10.38 | 2.209 | 10.23 | 9.866 | 1.498 | 0.059 |
| 2017 | 511100 | 0.711 | 1.548 | 0.649 | 13.23 | 5.864 | 10.71 | 2.654 | 9.821 | 10.13 | 1.763 | 0.095 |
| 2017 | 511300 | 0.709 | 0.841 | 0.446 | 12.17 | 6.597 | 11.24 | 2.682 | 9.988 | 9.528 | 0.877 | 0.086 |
| 2017 | 511400 | 0.717 | 1.11 | 0.312 | 12.72 | 5.844 | 10.1 | 2.894 | 10.69 | 9.954 | 1.407 | 0.11 |
| 2017 | 511500 | 0.708 | 1.37 | 0.246 | 13.18 | 6.319 | 10.19 | 2.339 | 10.78 | 10.02 | 1.838 | 0.098 |
| 2017 | 511600 | 0.71 | 0.818 | 0.14 | 11.87 | 6.142 | 9.224 | 3.235 | 10.18 | 9.659 | 1.177 | 0.084 |
| 2017 | 511700 | 0.705 | 0.942 | 0.201 | 12.4 | 6.51 | 10.11 | 0.811 | 10.34 | 9.711 | 1.153 | 0.074 |
| 2017 | 511800 | 0.727 | 1.548 | 1.017 | 13.85 | 5.037 | 10.81 | 2.688 | 9.154 | 10.17 | 1.961 | 0.072 |
| 2017 | 511900 | 0.667 | 0.64 | 0.069 | 12.15 | 5.93 | 8.527 | 1.418 | 11.15 | 9.362 | 0.654 | 0.087 |
| 2017 | 512000 | 0.701 | 1.07 | 0.086 | 12.74 | 5.855 | 8.348 | 2.809 | 11.07 | 9.688 | 1.196 | 0.054 |
| 2017 | 520100 | 0.703 | 2.699 | 1.324 | 15.22 | 6.011 | 12.77 | 2.333 | 8.223 | 10.64 | 1.417 | 0.183 |
| 2017 | 520200 | 0.696 | 0.921 | 0.212 | 14.4 | 5.835 | 9.741 | 2.56 | 13.72 | 9.643 | 2.308 | 0.215 |
| 2017 | 520300 | 0.711 | 1.303 | 0.352 | 13.84 | 6.691 | 11.13 | 2.402 | 12.41 | 9.692 | 1.425 | 0.133 |
| 2017 | 520400 | 0.678 | 1.08 | 0.272 | 13.68 | 5.707 | 9.751 | 3.006 | 12.48 | 9.359 | 1.529 | 0.179 |
| 2017 | 530100 | 0.715 | 2.745 | 1.255 | 14.91 | 6.333 | 13.13 | 2.088 | 11.16 | 11.01 | 1.169 | 0.153 |
| 2017 | 530300 | 0.706 | 0.962 | 0.189 | 12.95 | 6.494 | 10.28 | 2.354 | 8.001 | 9.857 | 1.981 | 0.109 |
| 2017 | 530400 | 0.708 | 1.713 | 0.274 | 14.79 | 5.389 | 9.69 | 2.888 | 9.67 | 10.89 | 3.137 | 0.108 |
| 2017 | 530500 | 0.685 | 0.62 | 0.214 | 12.67 | 5.572 | 9.519 | 2.865 | 8.631 | 9.513 | 0.555 | 0.157 |
| 2017 | 530600 | 0.686 | 0.282 | 0.083 | 11.94 | 6.428 | 9.475 | 2.671 | 8.001 | 9.094 | 0.795 | 0.125 |
| 2017 | 530700 | 0.714 | 1.123 | 0.536 | 13.62 | 4.812 | 10.02 | 2.409 | 3.989 | 9.531 | 0.814 | 0.208 |
| 2017 | 530800 | 0.703 | 0.754 | 0.148 | 13.32 | 5.533 | 9.373 | 2.638 | 8.913 | 9.364 | 0.862 | 0.153 |
| 2017 | 530900 | 0.697 | 0.635 | 0.134 | 12.46 | 5.476 | 9.069 | 2.648 | 8.006 | 9.406 | 1.208 | 0.123 |
| 2018 | 310000 | 0.731 | 4.158 | 0.899 | 17.19 | 7.288 | 13.16 | 1.521 | 16.25 | 12.24 | 1.238 | 0.26 |
| 2018 | 320100 | 0.719 | 4.159 | 1.463 | 16.26 | 6.547 | 13.65 | 3.186 | 14.75 | 11.65 | 1.414 | 0.177 |
| 2018 | 320200 | 0.724 | 4.275 | 0.67 | 16.12 | 6.209 | 11.57 | 3.318 | 14.71 | 12.13 | 1.967 | 0.105 |
| 2018 | 320300 | 0.721 | 2.454 | 0.627 | 14.71 | 6.952 | 11.78 | 3.112 | 14.04 | 10.67 | 1.823 | 0.114 |
| 2018 | 320400 | 0.727 | 4.132 | 0.727 | 15.71 | 5.945 | 11.74 | 3.038 | 14.29 | 11.62 | 2.047 | 0.122 |
| 2018 | 320500 | 0.718 | 4.689 | 0.777 | 16.89 | 6.557 | 12.29 | 3.45 | 14.91 | 12.09 | 2.517 | 0.146 |
| 2018 | 320600 | 0.702 | 3.503 | 0.533 | 15.42 | 6.637 | 11.46 | 3.436 | 14.35 | 10.91 | 1.891 | 0.118 |
| 2018 | 320700 | 0.718 | 2.469 | 0.394 | 14.43 | 6.28 | 10.64 | 3.131 | 12.9 | 10.28 | 1.766 | 0.132 |
| 2018 | 320800 | 0.723 | 2.799 | 0.64 | 14.33 | 6.33 | 11.18 | 3.152 | 13.57 | 10.45 | 2.031 | 0.115 |
| 2018 | 320900 | 0.715 | 3.002 | 0.429 | 15.05 | 6.715 | 11.07 | 3.237 | 13.31 | 10.5 | 1.938 | 0.101 |
| 2018 | 321000 | 0.708 | 3.919 | 0.808 | 15.07 | 6.129 | 11.28 | 3.132 | 13.6 | 11.03 | 1.862 | 0.104 |
| 2018 | 321100 | 0.732 | 4.052 | 1.024 | 15.62 | 5.602 | 11.34 | 3.336 | 13.26 | 11.42 | 2.057 | 0.093 |
| 2018 | 321200 | 0.703 | 3.452 | 0.525 | 14.92 | 6.221 | 11.06 | 3.364 | 13.81 | 10.81 | 1.977 | 0.12 |
| 2018 | 321300 | 0.708 | 2.727 | 0.24 | 14.5 | 6.382 | 9.945 | 3.219 | 12.43 | 10.03 | 1.815 | 0.143 |
| 2018 | 330100 | 0.709 | 4.283 | 0.98 | 16.54 | 6.652 | 13.12 | 2.609 | 15.32 | 11.73 | 1.626 | 0.191 |
| 2018 | 330200 | 0.717 | 4.32 | 0.669 | 16.38 | 6.402 | 11.92 | 2.337 | 14.87 | 11.74 | 1.952 | 0.172 |
| 2018 | 330300 | 0.715 | 3.847 | 0.447 | 14.6 | 6.72 | 11.39 | 2.732 | 12.75 | 11.04 | 1.962 | 0.103 |
| 2018 | 330400 | 0.714 | 4.234 | 0.442 | 15.71 | 5.886 | 11.17 | 3.126 | 14.55 | 11.49 | 2.348 | 0.137 |
| 2018 | 330500 | 0.711 | 4.262 | 0.417 | 15.59 | 5.587 | 10.2 | 3.341 | 13.64 | 11.21 | 1.878 | 0.133 |
| 2018 | 330600 | 0.712 | 4.426 | 0.577 | 15.77 | 6.103 | 11.51 | 3.06 | 13.7 | 11.54 | 2.151 | 0.106 |
| 2018 | 330700 | 0.716 | 3.895 | 0.536 | 15.28 | 6.192 | 11.4 | 3.249 | 12.26 | 11.22 | 1.709 | 0.112 |
| 2018 | 330800 | 0.733 | 3.2 | 0.248 | 15.27 | 5.553 | 9.576 | 3.569 | 10.8 | 10.55 | 1.55 | 0.125 |
| 2018 | 330900 | 0.714 | 3.168 | 0.646 | 15.76 | 4.575 | 10.04 | 3.088 | 12.53 | 11.23 | 1.174 | 0.187 |
| 2018 | 331000 | 0.715 | 3.78 | 0.247 | 14.85 | 6.405 | 10.47 | 3.303 | 12.16 | 11.12 | 2.363 | 0.1 |
| 2018 | 331100 | 0.729 | 3.34 | 0.313 | 15.05 | 5.598 | 10.59 | 2.864 | 11.17 | 10.42 | 1.562 | 0.134 |
| 2018 | 340100 | 0.689 | 3.653 | 1.234 | 16.31 | 6.631 | 13.12 | 2.934 | 14.58 | 10.75 | 1.347 | 0.261 |
| 2018 | 340200 | 0.716 | 3.355 | 1.019 | 16.55 | 5.964 | 11.8 | 3.169 | 14.47 | 10.95 | 1.869 | 0.205 |
| 2018 | 340300 | 0.712 | 2.264 | 0.648 | 14.83 | 5.951 | 11.03 | 3.098 | 13.74 | 10.25 | 1.322 | 0.139 |
| 2018 | 340400 | 0.7 | 1.901 | 0.68 | 13.87 | 5.966 | 11 | 2.822 | 12.15 | 10.47 | 1.535 | 0.118 |
| 2018 | 340500 | 0.723 | 3.219 | 0.881 | 15.44 | 5.434 | 10.94 | 3.018 | 14.31 | 11.15 | 2.43 | 0.148 |
| 2018 | 340600 | 0.7 | 1.926 | 0.681 | 13.1 | 5.384 | 10.56 | 2.91 | 12.04 | 10.25 | 1.828 | 0.103 |
| 2018 | 340700 | 0.711 | 2.271 | 0.703 | 15.08 | 5.142 | 10.44 | 2.609 | 12.29 | 11.08 | 1.764 | 0.136 |
| 2018 | 340800 | 0.71 | 2.019 | 0.369 | 14.51 | 6.269 | 10.53 | 3.094 | 12.04 | 10.05 | 1.578 | 0.081 |
| 2018 | 341000 | 0.732 | 2.122 | 0.581 | 15.18 | 5.004 | 10.03 | 3.239 | 11.79 | 10.38 | 0.839 | 0.136 |
| 2018 | 341100 | 0.708 | 2.42 | 0.595 | 14.69 | 6.118 | 10.89 | 3.751 | 13.73 | 10.34 | 1.386 | 0.127 |
| 2018 | 341200 | 0.7 | 1.709 | 0.265 | 13.06 | 6.976 | 10.51 | 3.184 | 12.52 | 9.348 | 0.839 | 0.162 |
| 2018 | 341300 | 0.694 | 1.177 | 0.248 | 13.46 | 6.488 | 10.13 | 3.464 | 13.32 | 9.667 | 0.789 | 0.098 |
| 2018 | 341500 | 0.713 | 2.05 | 0.216 | 14.42 | 6.378 | 10.5 | 3.276 | 12.72 | 9.521 | 1.095 | 0.113 |
| 2018 | 341600 | 0.705 | 1.36 | 0.159 | 13.58 | 6.488 | 9.475 | 3.614 | 13.3 | 9.601 | 0.839 | 0.117 |
| 2018 | 341700 | 0.721 | 2.476 | 0.62 | 14.28 | 5.088 | 10.17 | 3.241 | 12.48 | 9.936 | 1.279 | 0.16 |
| 2018 | 341800 | 0.727 | 2.526 | 0.132 | 15.42 | 5.631 | 8.879 | 3.529 | 13.52 | 10.44 | 1.196 | 0.143 |
| 2018 | 360100 | 0.701 | 3.237 | 1.58 | 15.45 | 6.277 | 13.32 | 2.575 | 14.65 | 10.97 | 1.57 | 0.134 |
| 2018 | 360200 | 0.712 | 2.494 | 0.866 | 14.27 | 5.136 | 10.39 | 3.249 | 11.9 | 10.59 | 1.627 | 0.119 |
| 2018 | 360300 | 0.717 | 2.611 | 0.414 | 15 | 5.298 | 10.25 | 3.244 | 12.49 | 10.4 | 2.303 | 0.13 |
| 2018 | 360400 | 0.715 | 2.421 | 0.673 | 14.61 | 6.26 | 11.44 | 3.08 | 14.18 | 10.23 | 1.691 | 0.16 |
| 2018 | 360500 | 0.733 | 2.259 | 0.928 | 14.49 | 4.828 | 10.48 | 3.191 | 12.66 | 10.67 | 1.657 | 0.13 |
| 2018 | 360600 | 0.701 | 3.121 | 0.254 | 15.32 | 4.86 | 9.128 | 2.904 | 12.25 | 10.33 | 1.511 | 0.183 |
| 2018 | 360700 | 0.709 | 2.31 | 0.425 | 14.64 | 6.889 | 11.49 | 2.857 | 14.01 | 9.771 | 1.002 | 0.142 |
| 2018 | 360800 | 0.701 | 2.19 | 0.266 | 14.57 | 6.29 | 10.62 | 3.086 | 13.56 | 9.832 | 1.472 | 0.15 |
| 2018 | 360900 | 0.7 | 1.988 | 0.367 | 15.09 | 6.405 | 10.83 | 3.216 | 13.23 | 9.939 | 1.329 | 0.172 |
| 2018 | 361000 | 0.703 | 2.442 | 0.354 | 14.68 | 6.068 | 10.38 | 3.121 | 12.45 | 9.876 | 1.541 | 0.137 |
| 2018 | 361100 | 0.711 | 1.836 | 0.225 | 13.98 | 6.671 | 10.39 | 3.114 | 13.63 | 9.732 | 1.132 | 0.161 |
| 2018 | 420100 | 0.716 | 3.626 | 1.575 | 16.54 | 6.784 | 13.78 | 2.577 | 15.79 | 11.39 | 1.066 | 0.169 |
| 2018 | 420200 | 0.699 | 2.381 | 0.652 | 14.36 | 5.609 | 10.73 | 3.079 | 11.7 | 10.69 | 1.724 | 0.082 |
| 2018 | 420300 | 0.68 | 1.868 | 0.446 | 14.1 | 5.849 | 10.84 | 3.147 | 12.3 | 10.31 | 1.598 | 0.09 |
| 2018 | 420500 | 0.71 | 2.901 | 0.585 | 15.1 | 5.971 | 10.96 | 3.303 | 12.12 | 10.85 | 1.897 | 0.092 |
| 2018 | 420600 | 0.678 | 1.755 | 0.322 | 15.45 | 6.384 | 10.62 | 2.861 | 13.27 | 10.47 | 1.826 | 0.111 |
| 2018 | 420700 | 0.692 | 1.817 | 0.447 | 14.81 | 4.71 | 9.615 | 2.447 | 10.15 | 10.79 | 1.881 | 0.088 |
| 2018 | 420800 | 0.696 | 2.155 | 0.238 | 14.71 | 5.68 | 9.65 | 3.045 | 12.63 | 10.69 | 1.665 | 0.063 |
| 2018 | 420900 | 0.685 | 1.952 | 0.289 | 14.53 | 6.25 | 10.48 | 3.248 | 12.35 | 10.3 | 1.364 | 0.073 |
| 2018 | 421000 | 0.698 | 1.771 | 0.627 | 14.24 | 6.463 | 11.53 | 2.517 | 9.863 | 10.12 | 1.679 | 0.067 |
| 2018 | 421100 | 0.689 | 1.344 | 0.278 | 14.05 | 6.608 | 10.64 | 3.395 | 10.48 | 9.975 | 1.899 | 0.07 |
| 2018 | 421200 | 0.71 | 1.962 | 0.428 | 14.35 | 5.72 | 10.33 | 3.419 | 10.19 | 10.21 | 1.642 | 0.1 |
| 2018 | 421300 | 0.713 | 1.405 | 0.187 | 13.31 | 5.525 | 8.824 | 2.613 | 11.52 | 10.14 | 2.018 | 0.059 |
| 2018 | 430100 | 0.711 | 3.397 | 1.455 | 15.42 | 6.592 | 13.46 | 2.578 | 15.16 | 11 | 0.933 | 0.174 |
| 2018 | 430200 | 0.701 | 2.588 | 0.903 | 15.32 | 5.999 | 11.44 | 3.051 | 13.71 | 10.64 | 1.483 | 0.095 |
| 2018 | 430300 | 0.702 | 2.423 | 1.244 | 15.21 | 5.666 | 11.82 | 3.355 | 13.71 | 10.61 | 0.965 | 0.09 |
| 2018 | 430400 | 0.704 | 1.621 | 0.763 | 13.26 | 6.686 | 11.7 | 2.492 | 13.76 | 10.14 | 0.921 | 0.07 |
| 2018 | 430500 | 0.694 | 1.437 | 0.292 | 12.92 | 6.719 | 10.42 | 2.983 | 12.21 | 9.661 | 0.775 | 0.064 |
| 2018 | 430600 | 0.707 | 1.563 | 0.451 | 14.2 | 6.344 | 10.82 | 3.104 | 12.82 | 10.54 | 1.459 | 0.057 |
| 2018 | 430700 | 0.706 | 1.53 | 0.444 | 14.13 | 6.405 | 10.88 | 2.676 | 13.63 | 10.37 | 1.335 | 0.069 |
| 2018 | 430800 | 0.728 | 1.148 | 0.566 | 13.19 | 5.136 | 9.609 | 3.029 | 11.37 | 9.922 | 0.525 | 0.082 |
| 2018 | 430900 | 0.7 | 1.696 | 0.406 | 13.6 | 6.17 | 10.6 | 2.715 | 12.24 | 9.965 | 0.671 | 0.058 |
| 2018 | 431000 | 0.719 | 1.375 | 0.289 | 14.2 | 6.282 | 10.3 | 2.539 | 14.05 | 10.26 | 1.02 | 0.079 |
| 2018 | 431100 | 0.714 | 1.245 | 0.282 | 13.6 | 6.469 | 10.31 | 3.026 | 13.6 | 9.895 | 0.687 | 0.081 |
| 2018 | 431200 | 0.697 | 1.179 | 0.325 | 13.87 | 6.261 | 10.35 | 2.556 | 10.53 | 9.882 | 0.692 | 0.07 |
| 2018 | 431300 | 0.71 | 1.541 | 0.468 | 13.24 | 6.12 | 10.43 | 3.804 | 12.78 | 9.959 | 1.328 | 0.065 |
| 2018 | 500000 | 0.709 | 2.662 | 0.726 | 14.52 | 8.133 | 13.54 | 2.604 | 15.73 | 10.46 | 1.141 | 0.156 |
| 2018 | 510100 | 0.701 | 3.687 | 1.078 | 15.42 | 7.297 | 13.64 | 2.653 | 15.73 | 11.26 | 0.999 | 0.142 |
| 2018 | 510300 | 0.7 | 1.703 | 0.57 | 13.91 | 5.778 | 10.59 | 2.697 | 8.89 | 10.21 | 1.184 | 0.058 |
| 2018 | 510400 | 0.715 | 2.591 | 0.612 | 13.96 | 4.682 | 10.19 | 2.586 | 8.89 | 11.11 | 3.004 | 0.072 |
| 2018 | 510500 | 0.697 | 1.499 | 0.448 | 13.3 | 6.234 | 10.78 | 2.353 | 11.41 | 9.907 | 1.079 | 0.13 |
| 2018 | 510600 | 0.71 | 2.433 | 0.884 | 13.25 | 5.958 | 11.64 | 2.752 | 11.57 | 10.56 | 1.4 | 0.064 |
| 2018 | 510700 | 0.705 | 2.592 | 0.84 | 15.38 | 6.284 | 11.85 | 2.899 | 10.99 | 10.4 | 0.978 | 0.061 |
| 2018 | 510800 | 0.709 | 1.407 | 0.237 | 12.59 | 5.707 | 9.976 | 2.643 | 9.633 | 9.582 | 0.83 | 0.088 |
| 2018 | 510900 | 0.706 | 1.507 | 0.211 | 12.8 | 5.9 | 9.502 | 3.292 | 9.633 | 9.81 | 0.952 | 0.077 |
| 2018 | 511000 | 0.698 | 1.427 | 0.39 | 12.92 | 6.021 | 10.44 | 2.282 | 10.19 | 9.927 | 1.467 | 0.06 |
| 2018 | 511100 | 0.71 | 1.605 | 0.633 | 12.57 | 5.861 | 10.85 | 2.612 | 10.29 | 10.19 | 1.727 | 0.097 |
| 2018 | 511300 | 0.708 | 1.078 | 0.422 | 12.5 | 6.59 | 11.24 | 2.718 | 10.55 | 9.589 | 0.859 | 0.087 |
| 2018 | 511400 | 0.718 | 1.419 | 0.508 | 12.52 | 5.844 | 10.12 | 2.875 | 10.9 | 10.01 | 1.343 | 0.112 |
| 2018 | 511500 | 0.705 | 1.409 | 0.239 | 12.75 | 6.314 | 10.25 | 2.553 | 10.94 | 10.08 | 1.8 | 0.105 |
| 2018 | 511600 | 0.708 | 1.034 | 0.127 | 11.92 | 6.136 | 9.517 | 3.236 | 10.19 | 9.719 | 1.153 | 0.087 |
| 2018 | 511700 | 0.703 | 1.103 | 0.203 | 12.35 | 6.501 | 10.17 | 2.542 | 10.37 | 9.768 | 1.1 | 0.076 |
| 2018 | 511800 | 0.725 | 2.043 | 1.026 | 13.82 | 5.03 | 10.85 | 2.747 | 8.104 | 10.23 | 1.871 | 0.077 |
| 2018 | 511900 | 0.667 | 0.95 | 0.097 | 11.81 | 5.908 | 8.604 | 1.475 | 11.12 | 9.422 | 0.641 | 0.081 |
| 2018 | 512000 | 0.701 | 1.11 | 0.088 | 13.27 | 5.846 | 8.474 | 3.031 | 9.927 | 9.745 | 1.141 | 0.053 |
| 2018 | 520100 | 0.702 | 3.141 | 1.314 | 15.6 | 6.035 | 12.85 | 2.303 | 8.825 | 10.7 | 1.352 | 0.183 |
| 2018 | 520200 | 0.697 | 1.179 | 0.202 | 14.38 | 5.855 | 9.89 | 2.913 | 13.87 | 9.701 | 2.202 | 0.203 |
| 2018 | 520300 | 0.708 | 1.606 | 0.358 | 13.92 | 6.695 | 10.81 | 2.565 | 12.06 | 9.749 | 1.36 | 0.142 |
| 2018 | 520400 | 0.679 | 1.486 | 0.251 | 13.97 | 5.717 | 9.865 | 3.006 | 12.53 | 9.416 | 1.459 | 0.168 |
| 2018 | 530100 | 0.714 | 3.118 | 1.27 | 14.96 | 6.349 | 13.21 | 2.144 | 11.41 | 11.07 | 1.148 | 0.149 |
| 2018 | 530300 | 0.705 | 1.078 | 0.213 | 12.31 | 6.498 | 10.38 | 3.002 | 7.939 | 9.918 | 1.944 | 0.105 |
| 2018 | 530400 | 0.705 | 2.038 | 0.27 | 14.41 | 5.394 | 9.729 | 3.14 | 9.172 | 10.95 | 3.079 | 0.103 |
| 2018 | 530500 | 0.683 | 0.789 | 0.226 | 12.68 | 5.576 | 9.625 | 2.929 | 9.659 | 9.574 | 0.545 | 0.154 |
| 2018 | 530600 | 0.686 | 0.406 | 0.091 | 11.96 | 6.438 | 9.668 | 2.756 | 7.939 | 9.156 | 0.78 | 0.132 |
| 2018 | 530700 | 0.713 | 1.157 | 0.552 | 13.68 | 4.812 | 9.994 | 2.398 | 10.17 | 9.592 | 0.799 | 0.204 |
| 2018 | 530800 | 0.702 | 0.881 | 0.135 | 13.29 | 5.537 | 9.402 | 2.787 | 11.04 | 9.425 | 0.846 | 0.144 |
| 2018 | 530900 | 0.695 | 0.841 | 0.143 | 12.56 | 5.485 | 9.275 | 2.617 | 8.088 | 9.467 | 1.185 | 0.124 |
| 2019 | 310000 | 0.729 | 4.241 | 0.791 | 17.09 | 7.292 | 13.2 | 1.552 | 16.39 | 12.3 | 1.215 | 0.249 |
| 2019 | 320100 | 0.719 | 4.363 | 1.35 | 16.44 | 6.565 | 13.69 | 3.191 | 14.86 | 11.72 | 1.388 | 0.18 |
| 2019 | 320200 | 0.722 | 4.347 | 0.593 | 16.21 | 6.221 | 11.7 | 3.316 | 14.73 | 12.19 | 1.95 | 0.102 |
| 2019 | 320300 | 0.725 | 2.572 | 0.592 | 14.73 | 6.949 | 11.92 | 3.137 | 14.18 | 10.72 | 1.807 | 0.097 |
| 2019 | 320400 | 0.729 | 4.183 | 0.717 | 15.75 | 5.953 | 11.78 | 3.085 | 14.41 | 11.68 | 2.009 | 0.121 |
| 2019 | 320500 | 0.718 | 4.729 | 0.642 | 17.04 | 6.583 | 12.4 | 3.39 | 14.97 | 12.15 | 2.496 | 0.145 |
| 2019 | 320600 | 0.705 | 3.3 | 0.513 | 15.28 | 6.633 | 11.7 | 3.444 | 14.42 | 10.97 | 1.856 | 0.114 |
| 2019 | 320700 | 0.721 | 2.34 | 0.36 | 14.78 | 6.28 | 10.78 | 3.181 | 12.96 | 10.33 | 1.751 | 0.129 |
| 2019 | 320800 | 0.723 | 2.688 | 0.566 | 14.3 | 6.328 | 11.24 | 3.184 | 13.49 | 10.51 | 2.013 | 0.113 |
| 2019 | 320900 | 0.723 | 3.003 | 0.393 | 14.99 | 6.711 | 11.12 | 3.225 | 13.35 | 10.56 | 1.922 | 0.096 |
| 2019 | 321000 | 0.708 | 3.738 | 0.758 | 15.15 | 6.125 | 11.48 | 3.165 | 13.77 | 11.08 | 1.846 | 0.095 |
| 2019 | 321100 | 0.732 | 3.867 | 0.881 | 15.73 | 5.598 | 11.51 | 3.38 | 13.03 | 11.47 | 2.04 | 0.09 |
| 2019 | 321200 | 0.705 | 3.426 | 0.506 | 15.08 | 6.217 | 11.09 | 3.414 | 13.83 | 10.86 | 1.96 | 0.117 |
| 2019 | 321300 | 0.709 | 2.662 | 0.216 | 14.73 | 6.384 | 10.12 | 3.189 | 12.64 | 10.1 | 1.781 | 0.14 |
| 2019 | 330100 | 0.708 | 4.362 | 0.94 | 16.74 | 6.678 | 13.16 | 2.617 | 15.26 | 11.76 | 1.654 | 0.193 |
| 2019 | 330200 | 0.716 | 4.365 | 0.571 | 16.83 | 6.41 | 11.96 | 2.554 | 14.3 | 11.8 | 1.935 | 0.171 |
| 2019 | 330300 | 0.714 | 3.817 | 0.381 | 14.86 | 6.724 | 11.74 | 2.748 | 13.17 | 11.07 | 1.997 | 0.102 |
| 2019 | 330400 | 0.713 | 4.348 | 0.511 | 16.02 | 5.897 | 11.19 | 3.181 | 14.86 | 11.55 | 2.328 | 0.14 |
| 2019 | 330500 | 0.71 | 4.131 | 0.399 | 15.76 | 5.591 | 10.27 | 3.381 | 14.09 | 11.25 | 1.91 | 0.136 |
| 2019 | 330600 | 0.713 | 4.113 | 0.608 | 16.16 | 6.105 | 11.55 | 3.12 | 13.02 | 11.58 | 2.188 | 0.104 |
| 2019 | 330700 | 0.718 | 4.032 | 0.494 | 15.43 | 6.198 | 11.46 | 3.279 | 11.91 | 11.25 | 1.739 | 0.11 |
| 2019 | 330800 | 0.729 | 2.967 | 0.235 | 15.6 | 5.553 | 9.709 | 3.616 | 10.89 | 10.58 | 1.577 | 0.125 |
| 2019 | 330900 | 0.714 | 3.086 | 0.631 | 15.86 | 4.575 | 10.14 | 3.106 | 12.75 | 11.27 | 1.195 | 0.186 |
| 2019 | 331000 | 0.715 | 3.815 | 0.22 | 15.37 | 6.409 | 10.54 | 3.298 | 12.97 | 11.17 | 2.343 | 0.095 |
| 2019 | 331100 | 0.728 | 3.349 | 0.333 | 15.25 | 5.602 | 10.52 | 2.887 | 11.33 | 10.47 | 1.549 | 0.135 |
| 2019 | 340100 | 0.69 | 3.696 | 1.178 | 16.64 | 6.646 | 13.19 | 2.97 | 14.67 | 10.79 | 1.37 | 0.256 |
| 2019 | 340200 | 0.719 | 3.28 | 1.038 | 16.39 | 5.966 | 11.86 | 3.134 | 14.52 | 11 | 1.823 | 0.194 |
| 2019 | 340300 | 0.712 | 2.337 | 0.624 | 15.28 | 5.956 | 11.09 | 3.273 | 13.78 | 10.3 | 1.289 | 0.139 |
| 2019 | 340400 | 0.7 | 1.838 | 0.654 | 14.07 | 5.969 | 11.08 | 2.857 | 12.28 | 10.52 | 1.496 | 0.115 |
| 2019 | 340500 | 0.723 | 3.16 | 0.83 | 15.37 | 5.434 | 11.03 | 3.06 | 14.42 | 11.2 | 2.37 | 0.145 |
| 2019 | 340600 | 0.702 | 2.126 | 0.705 | 12.85 | 5.389 | 10.69 | 3.008 | 12.2 | 10.29 | 1.782 | 0.103 |
| 2019 | 340700 | 0.71 | 2.514 | 0.675 | 15.53 | 5.142 | 10.44 | 2.633 | 12.53 | 11.12 | 1.72 | 0.133 |
| 2019 | 340800 | 0.709 | 1.945 | 0.356 | 14.58 | 6.271 | 10.66 | 3.128 | 12.27 | 10.08 | 1.605 | 0.078 |
| 2019 | 341000 | 0.729 | 2.193 | 0.543 | 15.22 | 5.004 | 10.03 | 3.307 | 11.9 | 10.43 | 0.818 | 0.133 |
| 2019 | 341100 | 0.707 | 2.656 | 0.564 | 14.94 | 6.12 | 10.93 | 3.694 | 13.84 | 10.39 | 1.351 | 0.127 |
| 2019 | 341200 | 0.697 | 1.764 | 0.238 | 13.42 | 6.982 | 10.66 | 3.139 | 12.65 | 9.396 | 0.818 | 0.162 |
| 2019 | 341300 | 0.693 | 1.309 | 0.262 | 13.18 | 6.489 | 10.3 | 3.466 | 13.43 | 9.701 | 0.803 | 0.106 |
| 2019 | 341500 | 0.711 | 1.885 | 0.241 | 14.48 | 6.382 | 10.75 | 3.304 | 12.84 | 9.555 | 1.114 | 0.108 |
| 2019 | 341600 | 0.704 | 1.534 | 0.158 | 13.68 | 6.497 | 9.826 | 3.659 | 13.41 | 9.635 | 0.854 | 0.123 |
| 2019 | 341700 | 0.722 | 2.516 | 0.555 | 14.23 | 5.088 | 10.31 | 3.272 | 12.53 | 9.985 | 1.248 | 0.148 |
| 2019 | 341800 | 0.727 | 2.457 | 0.108 | 15.54 | 5.631 | 8.903 | 3.553 | 13.63 | 10.48 | 1.217 | 0.143 |
| 2019 | 360100 | 0.699 | 3.233 | 1.448 | 15.66 | 6.284 | 13.35 | 2.602 | 14.77 | 11.01 | 1.532 | 0.128 |
| 2019 | 360200 | 0.716 | 2.664 | 0.685 | 14.81 | 5.142 | 10.43 | 3.402 | 12 | 10.62 | 1.587 | 0.123 |
| 2019 | 360300 | 0.717 | 2.381 | 0.534 | 15.09 | 5.298 | 10.38 | 3.207 | 12.59 | 10.44 | 2.247 | 0.127 |
| 2019 | 360400 | 0.714 | 2.539 | 0.656 | 15.02 | 6.263 | 11.5 | 3.063 | 14.3 | 10.26 | 1.65 | 0.157 |
| 2019 | 360500 | 0.729 | 2.388 | 0.96 | 14.56 | 4.828 | 10.59 | 3.213 | 12.77 | 10.7 | 1.617 | 0.124 |
| 2019 | 360600 | 0.701 | 2.785 | 0.273 | 15.89 | 4.86 | 6.933 | 2.804 | 12.37 | 10.36 | 1.474 | 0.185 |
| 2019 | 360700 | 0.707 | 2.6 | 0.414 | 14.85 | 6.891 | 11.61 | 2.964 | 14.14 | 9.806 | 0.978 | 0.139 |
| 2019 | 360800 | 0.699 | 2.273 | 0.249 | 14.69 | 6.292 | 10.65 | 3.171 | 13.68 | 9.868 | 1.437 | 0.147 |
| 2019 | 360900 | 0.699 | 2.23 | 0.361 | 15.24 | 6.404 | 10.98 | 3.189 | 13.35 | 9.974 | 1.36 | 0.167 |
| 2019 | 361000 | 0.702 | 2.616 | 0.224 | 14.92 | 6.071 | 10.48 | 3.161 | 12.56 | 9.911 | 1.504 | 0.132 |
| 2019 | 361100 | 0.71 | 2 | 0.232 | 14.37 | 6.676 | 10.56 | 3.129 | 13.74 | 9.781 | 1.104 | 0.156 |
| 2019 | 420100 | 0.715 | 3.792 | 1.432 | 16.78 | 6.809 | 13.82 | 2.582 | 15.95 | 11.43 | 1.04 | 0.162 |
| 2019 | 420200 | 0.697 | 2.472 | 0.639 | 14.39 | 5.609 | 10.77 | 3.349 | 11.92 | 10.72 | 1.73 | 0.078 |
| 2019 | 420300 | 0.68 | 2.123 | 0.426 | 14.38 | 5.846 | 11 | 3.152 | 11 | 10.35 | 1.56 | 0.088 |
| 2019 | 420500 | 0.71 | 2.952 | 0.591 | 15.26 | 5.969 | 11 | 3.388 | 12.25 | 10.89 | 1.852 | 0.088 |
| 2019 | 420600 | 0.677 | 2.078 | 0.312 | 15.34 | 6.38 | 10.84 | 2.718 | 13.4 | 10.5 | 1.83 | 0.106 |
| 2019 | 420700 | 0.691 | 2.034 | 0.475 | 14.08 | 4.718 | 9.669 | 2.947 | 10.32 | 10.83 | 1.884 | 0.085 |
| 2019 | 420800 | 0.694 | 2.298 | 0.267 | 14.89 | 5.673 | 9.836 | 3.059 | 12.74 | 10.74 | 1.592 | 0.061 |
| 2019 | 420900 | 0.685 | 1.979 | 0.282 | 14.59 | 6.244 | 10.5 | 3.204 | 12.49 | 10.34 | 1.331 | 0.071 |
| 2019 | 421000 | 0.699 | 1.912 | 0.598 | 14.22 | 6.457 | 11.68 | 2.503 | 10.44 | 10.15 | 1.638 | 0.065 |
| 2019 | 421100 | 0.689 | 1.51 | 0.318 | 14.09 | 6.604 | 10.63 | 3.242 | 10.6 | 10.01 | 1.902 | 0.066 |
| 2019 | 421200 | 0.71 | 2.052 | 0.733 | 14.59 | 5.72 | 10.39 | 3.437 | 10.15 | 10.24 | 1.602 | 0.097 |
| 2019 | 421300 | 0.712 | 1.607 | 0.181 | 13.39 | 5.517 | 8.918 | 2.656 | 11.66 | 10.18 | 2.022 | 0.057 |
| 2019 | 430100 | 0.708 | 3.45 | 1.241 | 15.71 | 6.604 | 13.41 | 2.632 | 15.3 | 11.03 | 0.937 | 0.175 |
| 2019 | 430200 | 0.7 | 2.645 | 0.725 | 15.68 | 5.996 | 11.53 | 3.078 | 13.87 | 10.68 | 1.488 | 0.093 |
| 2019 | 430300 | 0.703 | 2.393 | 1.171 | 15.32 | 5.666 | 11.85 | 3.37 | 13.88 | 10.64 | 0.968 | 0.077 |
| 2019 | 430400 | 0.701 | 1.653 | 0.645 | 13.39 | 6.681 | 11.77 | 2.55 | 13.9 | 10.17 | 0.925 | 0.069 |
| 2019 | 430500 | 0.692 | 1.727 | 0.28 | 13.33 | 6.717 | 10.51 | 2.722 | 12.37 | 9.694 | 0.778 | 0.065 |
| 2019 | 430600 | 0.702 | 1.672 | 0.333 | 14.53 | 6.346 | 10.88 | 3.102 | 13 | 10.58 | 1.465 | 0.055 |
| 2019 | 430700 | 0.703 | 1.691 | 0.418 | 14.3 | 6.404 | 10.96 | 3.036 | 13.79 | 10.4 | 1.341 | 0.068 |
| 2019 | 430800 | 0.723 | 1.288 | 0.381 | 13.62 | 5.13 | 9.693 | 2.803 | 11.53 | 9.956 | 0.527 | 0.082 |
| 2019 | 430900 | 0.698 | 1.91 | 0.429 | 13.84 | 6.159 | 10.7 | 2.946 | 12.4 | 9.998 | 0.674 | 0.057 |
| 2019 | 431000 | 0.716 | 1.563 | 0.273 | 14.35 | 6.28 | 10.33 | 2.451 | 14.19 | 10.3 | 1.024 | 0.078 |
| 2019 | 431100 | 0.714 | 1.3 | 0.276 | 14.05 | 6.468 | 10.39 | 3.046 | 13.76 | 9.928 | 0.689 | 0.08 |
| 2019 | 431200 | 0.695 | 1.452 | 0.259 | 14.31 | 6.261 | 10.41 | 2.608 | 10.71 | 9.916 | 0.695 | 0.069 |
| 2019 | 431300 | 0.707 | 1.592 | 0.414 | 13.61 | 6.12 | 10.49 | 3.149 | 12.95 | 9.993 | 1.333 | 0.066 |
| 2019 | 500000 | 0.708 | 2.628 | 0.714 | 14.66 | 8.136 | 13.72 | 2.666 | 15.78 | 10.49 | 1.145 | 0.14 |
| 2019 | 510100 | 0.699 | 3.551 | 0.965 | 15.77 | 7.313 | 13.69 | 2.764 | 16.02 | 11.29 | 0.997 | 0.138 |
| 2019 | 510300 | 0.698 | 1.927 | 0.568 | 14.41 | 5.768 | 10.72 | 2.873 | 8.853 | 10.26 | 1.119 | 0.055 |
| 2019 | 510400 | 0.713 | 2.632 | 0.516 | 14.14 | 4.682 | 10.27 | 2.693 | 10.84 | 11.14 | 2.998 | 0.069 |
| 2019 | 510500 | 0.693 | 1.569 | 0.443 | 13.83 | 6.232 | 11.17 | 2.339 | 11.61 | 9.944 | 1.077 | 0.129 |
| 2019 | 510600 | 0.709 | 2.384 | 0.874 | 13.71 | 5.951 | 11.69 | 3.223 | 11.36 | 10.59 | 1.398 | 0.064 |
| 2019 | 510700 | 0.705 | 2.566 | 0.967 | 14.81 | 6.275 | 11.96 | 2.936 | 11.73 | 10.47 | 0.959 | 0.06 |
| 2019 | 510800 | 0.706 | 1.29 | 0.211 | 12.62 | 5.7 | 10.15 | 2.682 | 9.751 | 9.618 | 0.829 | 0.083 |
| 2019 | 510900 | 0.697 | 1.62 | 0.2 | 13.26 | 5.892 | 9.571 | 3.158 | 12.19 | 9.876 | 0.885 | 0.078 |
| 2019 | 511000 | 0.697 | 1.502 | 0.4 | 12.88 | 6.011 | 10.52 | 2.707 | 10.35 | 9.96 | 1.473 | 0.058 |
| 2019 | 511100 | 0.708 | 1.787 | 0.568 | 12.57 | 5.858 | 10.78 | 2.592 | 10.79 | 10.23 | 1.734 | 0.096 |
| 2019 | 511300 | 0.706 | 1.13 | 0.436 | 12.38 | 6.585 | 11.35 | 2.759 | 10.96 | 9.622 | 0.863 | 0.088 |
| 2019 | 511400 | 0.711 | 1.624 | 0.489 | 12.21 | 5.835 | 10.17 | 2.856 | 11.04 | 10.08 | 1.297 | 0.112 |
| 2019 | 511500 | 0.703 | 1.535 | 0.243 | 13.79 | 6.314 | 10.36 | 2.574 | 10.72 | 10.12 | 1.797 | 0.107 |
| 2019 | 511600 | 0.706 | 1.131 | 0.124 | 12.22 | 6.129 | 9.619 | 3.361 | 10.22 | 9.755 | 1.151 | 0.087 |
| 2019 | 511700 | 0.701 | 1.109 | 0.215 | 12.47 | 6.491 | 10.37 | 2.621 | 10.46 | 9.782 | 1.095 | 0.076 |
| 2019 | 511800 | 0.721 | 1.811 | 0.968 | 14.42 | 5.03 | 10.88 | 3.37 | 8.847 | 10.29 | 1.738 | 0.077 |
| 2019 | 511900 | 0.667 | 0.967 | 0.06 | 12.07 | 5.903 | 8.861 | 2.891 | 11.7 | 9.459 | 0.64 | 0.079 |
| 2019 | 512000 | 0.702 | 0.976 | 0.163 | 13.46 | 5.835 | 8.975 | 3.042 | 10.74 | 9.812 | 1.054 | 0.05 |
| 2019 | 520100 | 0.699 | 3.264 | 1.267 | 15.71 | 6.059 | 13.06 | 2.327 | 14.02 | 10.7 | 1.352 | 0.172 |
| 2019 | 520200 | 0.695 | 1.53 | 0.22 | 14.26 | 5.866 | 10 | 2.911 | 14.09 | 9.701 | 2.202 | 0.145 |
| 2019 | 520300 | 0.705 | 1.807 | 0.37 | 14.05 | 6.708 | 11.52 | 2.582 | 12.61 | 9.749 | 1.36 | 0.133 |
| 2019 | 520400 | 0.676 | 1.783 | 0.222 | 14.17 | 5.727 | 9.976 | 3.191 | 11.32 | 9.416 | 1.459 | 0.132 |
| 2019 | 530100 | 0.713 | 3.248 | 1.217 | 15 | 6.36 | 13.34 | 2.286 | 13.01 | 11.07 | 1.148 | 0.146 |
| 2019 | 530300 | 0.707 | 1.176 | 0.211 | 12.45 | 6.503 | 10.49 | 3.013 | 7.887 | 9.918 | 1.944 | 0.102 |
| 2019 | 530400 | 0.708 | 2.061 | 0.24 | 14.35 | 5.398 | 9.863 | 3.11 | 8.842 | 10.95 | 3.079 | 0.09 |
| 2019 | 530500 | 0.684 | 0.602 | 0.204 | 12.5 | 5.576 | 9.819 | 2.931 | 7.92 | 9.574 | 0.545 | 0.145 |
| 2019 | 530600 | 0.684 | 0.573 | 0.092 | 11.98 | 6.444 | 9.758 | 2.79 | 6.18 | 9.156 | 0.78 | 0.128 |
| 2019 | 530700 | 0.711 | 0.424 | 0.538 | 13.62 | 4.812 | 10.02 | 2.441 | 8.922 | 9.592 | 0.799 | 0.201 |
| 2019 | 530800 | 0.7 | 0.765 | 0.121 | 13.25 | 5.537 | 9.399 | 2.973 | 8.512 | 9.425 | 0.846 | 0.123 |
| 2019 | 530900 | 0.697 | 0.666 | 0.144 | 12.86 | 5.485 | 9.34 | 2.728 | 8.166 | 9.467 | 1.185 | 0.121 |
| 2020 | 310000 | 0.726 | 4.047 | 0.835 | 16.61 | 7.819 | 13.22 | 1.56 | 16.45 | 12.3 | 1.215 | 0.241 |
| 2020 | 320100 | 0.716 | 4.669 | 1.417 | 16.45 | 6.583 | 13.73 | 3.219 | 14.95 | 11.72 | 1.388 | 0.18 |
| 2020 | 320200 | 0.72 | 4.79 | 0.57 | 16.08 | 6.232 | 11.8 | 3.301 | 14.73 | 12.19 | 1.95 | 0.102 |
| 2020 | 320300 | 0.723 | 3.309 | 0.606 | 14.54 | 6.945 | 12.01 | 3.154 | 14.23 | 10.72 | 1.807 | 0.096 |
| 2020 | 320400 | 0.726 | 4.681 | 0.753 | 15.55 | 5.957 | 11.88 | 3.248 | 14.44 | 11.68 | 2.009 | 0.122 |
| 2020 | 320500 | 0.716 | 5.234 | 0.667 | 17.18 | 6.612 | 12.48 | 3.293 | 15.16 | 12.15 | 2.496 | 0.145 |
| 2020 | 320600 | 0.703 | 3.727 | 0.532 | 15.51 | 6.628 | 11.76 | 3.347 | 14.44 | 10.97 | 1.856 | 0.114 |
| 2020 | 320700 | 0.719 | 2.777 | 0.373 | 14.26 | 6.281 | 10.92 | 3.2 | 13.05 | 10.33 | 1.751 | 0.126 |
| 2020 | 320800 | 0.721 | 3.095 | 0.586 | 14.22 | 6.324 | 11.41 | 3.152 | 13.52 | 10.51 | 2.013 | 0.112 |
| 2020 | 320900 | 0.721 | 3.312 | 0.395 | 15 | 6.703 | 11.19 | 3.204 | 13.46 | 10.56 | 1.922 | 0.097 |
| 2020 | 321000 | 0.706 | 4.153 | 0.813 | 14.82 | 6.12 | 11.5 | 3.184 | 13.83 | 11.08 | 1.846 | 0.094 |
| 2020 | 321100 | 0.729 | 4.312 | 0.925 | 15.63 | 5.596 | 11.59 | 3.391 | 13.21 | 11.47 | 2.04 | 0.088 |
| 2020 | 321200 | 0.702 | 3.732 | 0.513 | 14.66 | 6.209 | 11.14 | 3.447 | 13.87 | 10.86 | 1.96 | 0.116 |
| 2020 | 321300 | 0.707 | 3.202 | 0.246 | 14.8 | 6.384 | 10.2 | 3.208 | 12.86 | 10.1 | 1.781 | 0.14 |
| 2020 | 330100 | 0.706 | 4.741 | 0.943 | 16.69 | 6.702 | 13.22 | 2.519 | 15.42 | 11.76 | 1.654 | 0.338 |
| 2020 | 330200 | 0.713 | 4.601 | 0.563 | 16.73 | 6.419 | 12.03 | 2.922 | 14.35 | 11.8 | 1.935 | 0.299 |
| 2020 | 330300 | 0.712 | 4.153 | 0.379 | 15.02 | 6.726 | 11.7 | 2.817 | 11.43 | 11.07 | 1.997 | 0.159 |
| 2020 | 330400 | 0.712 | 4.566 | 0.52 | 16.04 | 5.906 | 11.24 | 3.21 | 14.42 | 11.55 | 2.328 | 0.228 |
| 2020 | 330500 | 0.708 | 4.206 | 0.414 | 15.8 | 5.591 | 10.43 | 3.353 | 13.64 | 11.25 | 1.91 | 0.231 |
| 2020 | 330600 | 0.711 | 4.388 | 0.678 | 15.74 | 6.104 | 11.67 | 3.12 | 12.9 | 11.58 | 2.188 | 0.158 |
| 2020 | 330700 | 0.715 | 4.465 | 0.528 | 15.24 | 6.202 | 11.63 | 3.422 | 12.32 | 11.25 | 1.739 | 0.171 |
| 2020 | 330800 | 0.727 | 3.278 | 0.224 | 15.57 | 5.549 | 9.732 | 3.589 | 12.64 | 10.58 | 1.577 | 0.194 |
| 2020 | 330900 | 0.716 | 3.406 | 0.648 | 15.83 | 4.566 | 10.13 | 3.1 | 14.46 | 11.27 | 1.195 | 0.281 |
| 2020 | 331000 | 0.712 | 4.067 | 0.216 | 14.88 | 6.408 | 10.6 | 3.199 | 14.36 | 11.17 | 2.343 | 0.14 |
| 2020 | 331100 | 0.725 | 3.781 | 0.339 | 15.26 | 5.601 | 9.995 | 2.97 | 12.56 | 10.47 | 1.549 | 0.214 |
| 2020 | 340100 | 0.689 | 5.086 | 1.283 | 17.97 | 5.543 | 13.28 | 2.932 | 14.72 | 10.79 | 1.37 | 0.252 |
| 2020 | 340200 | 0.718 | 4.625 | 1.012 | 17.54 | 4.875 | 11.91 | 3.195 | 14.56 | 11 | 1.823 | 0.192 |
| 2020 | 340300 | 0.71 | 3.709 | 0.669 | 16.56 | 4.731 | 11.14 | 3.283 | 13.63 | 10.3 | 1.289 | 0.13 |
| 2020 | 340400 | 0.699 | 3.304 | 0.761 | 14.98 | 4.823 | 11.19 | 2.866 | 12.44 | 10.52 | 1.496 | 0.106 |
| 2020 | 340500 | 0.722 | 4.663 | 0.856 | 16.6 | 4.312 | 11.1 | 3.111 | 14.48 | 11.2 | 2.37 | 0.149 |
| 2020 | 340600 | 0.701 | 3.715 | 0.705 | 15.06 | 4.291 | 10.75 | 3.054 | 12.29 | 10.29 | 1.782 | 0.105 |
| 2020 | 340700 | 0.709 | 3.807 | 0.67 | 16.76 | 3.991 | 10.54 | 3.25 | 12.61 | 11.12 | 1.72 | 0.135 |
| 2020 | 340800 | 0.709 | 3.591 | 0.399 | 15.83 | 5.046 | 10.82 | 3.025 | 12.38 | 10.08 | 1.605 | 0.078 |
| 2020 | 341000 | 0.728 | 3.448 | 0.707 | 16.37 | 3.93 | 10.17 | 3.445 | 12.03 | 10.43 | 0.818 | 0.132 |
| 2020 | 341100 | 0.706 | 4.135 | 0.553 | 16.2 | 4.947 | 11 | 3.7 | 13.91 | 10.39 | 1.351 | 0.129 |
| 2020 | 341200 | 0.695 | 3.261 | 0.266 | 14.75 | 5.654 | 10.8 | 3.139 | 12.74 | 9.396 | 0.818 | 0.155 |
| 2020 | 341300 | 0.693 | 3.083 | 0.235 | 14.93 | 5.288 | 10.45 | 3.473 | 13.51 | 9.701 | 0.803 | 0.105 |
| 2020 | 341500 | 0.71 | 3.383 | 0.434 | 15.29 | 5.246 | 10.84 | 3.317 | 12.95 | 9.555 | 1.114 | 0.11 |
| 2020 | 341600 | 0.701 | 3.249 | 0.208 | 14.94 | 5.191 | 10.02 | 3.736 | 12.61 | 9.635 | 0.854 | 0.119 |
| 2020 | 341700 | 0.721 | 3.847 | 0.609 | 15.47 | 3.947 | 10.49 | 3.275 | 12.66 | 9.985 | 1.248 | 0.15 |
| 2020 | 341800 | 0.725 | 4.003 | 0.118 | 16.5 | 4.595 | 9.376 | 3.569 | 13.7 | 10.48 | 1.217 | 0.141 |
| 2020 | 360100 | 0.697 | 3.389 | 1.495 | 15.6 | 6.439 | 13.44 | 2.428 | 14.85 | 11.01 | 1.532 | 0.126 |
| 2020 | 360200 | 0.714 | 2.892 | 0.819 | 14.97 | 5.087 | 10.51 | 3.407 | 12.06 | 10.62 | 1.587 | 0.119 |
| 2020 | 360300 | 0.715 | 2.562 | 0.586 | 15.29 | 5.196 | 10.62 | 3.136 | 12.65 | 10.44 | 2.247 | 0.124 |
| 2020 | 360400 | 0.712 | 3.027 | 0.753 | 15.02 | 6.131 | 11.72 | 3.09 | 14.37 | 10.26 | 1.65 | 0.153 |
| 2020 | 360500 | 0.726 | 3.02 | 1.069 | 14.78 | 4.79 | 10.98 | 3.22 | 12.83 | 10.7 | 1.617 | 0.123 |
| 2020 | 360600 | 0.699 | 3.037 | 0.314 | 15.67 | 4.749 | 9.4 | 3.29 | 12.44 | 10.36 | 1.474 | 0.18 |
| 2020 | 360700 | 0.766 | 3.027 | 0.455 | 15.01 | 6.799 | 11.69 | 3.036 | 14.22 | 9.806 | 0.978 | 0.137 |
| 2020 | 360800 | 0.697 | 2.723 | 0.241 | 15 | 6.102 | 10.71 | 3.136 | 13.75 | 9.868 | 1.437 | 0.142 |
| 2020 | 360900 | 0.697 | 2.738 | 0.366 | 15.44 | 6.216 | 11.04 | 3.181 | 13.42 | 9.974 | 1.36 | 0.162 |
| 2020 | 361000 | 0.699 | 5.172 | 0.31 | 15.23 | 5.89 | 10.71 | 3.307 | 12.63 | 9.911 | 1.504 | 0.129 |
| 2020 | 361100 | 0.708 | 2.41 | 0.229 | 14.63 | 6.476 | 10.67 | 3.071 | 13.82 | 9.781 | 1.104 | 0.148 |
| 2020 | 420100 | 0.712 | 4.179 | 1.439 | 16.63 | 6.82 | 13.88 | 2.749 | 15.56 | 11.43 | 1.04 | 0.136 |
| 2020 | 420200 | 0.697 | 2.756 | 0.679 | 14.59 | 5.611 | 10.9 | 3.388 | 11.83 | 10.72 | 1.73 | 0.061 |
| 2020 | 420300 | 0.68 | 2.557 | 0.411 | 14.48 | 5.833 | 10.94 | 3.121 | 10.81 | 10.35 | 1.56 | 0.07 |
| 2020 | 420500 | 0.709 | 3.098 | 0.6 | 15.17 | 5.966 | 11.02 | 3.388 | 11.49 | 10.89 | 1.852 | 0.054 |
| 2020 | 420600 | 0.677 | 2.568 | 0.335 | 15.06 | 6.378 | 11.19 | 2.723 | 13.26 | 10.5 | 1.83 | 0.061 |
| 2020 | 420700 | 0.69 | 2.526 | 0.51 | 14.66 | 4.716 | 9.718 | 2.969 | 10.4 | 10.83 | 1.884 | 0.073 |
| 2020 | 420800 | 0.693 | 2.562 | 0.271 | 15 | 5.666 | 10.03 | 3.098 | 11.37 | 10.74 | 1.592 | 0.047 |
| 2020 | 420900 | 0.685 | 2.238 | 0.283 | 14.48 | 6.232 | 10.51 | 3.206 | 12.21 | 10.34 | 1.331 | 0.056 |
| 2020 | 421000 | 0.699 | 2.248 | 0.666 | 14.16 | 6.45 | 11.5 | 2.535 | 11 | 10.15 | 1.638 | 0.052 |
| 2020 | 421100 | 0.688 | 2.009 | 0.345 | 14.18 | 6.599 | 10.72 | 3.541 | 10.63 | 10.01 | 1.902 | 0.051 |
| 2020 | 421200 | 0.709 | 2.565 | 0.441 | 14.64 | 5.721 | 10.61 | 3.443 | 10.16 | 10.24 | 1.602 | 0.077 |
| 2020 | 421300 | 0.711 | 2.049 | 0.176 | 13.68 | 5.51 | 8.964 | 2.734 | 10.4 | 10.18 | 2.022 | 0.044 |
| 2020 | 430100 | 0.703 | 3.521 | 1.401 | 15.52 | 6.914 | 13.46 | 3.072 | 15.43 | 11.03 | 0.937 | 0.193 |
| 2020 | 430200 | 0.691 | 2.992 | 0.787 | 16.08 | 5.967 | 11.61 | 3.221 | 14.03 | 10.68 | 1.488 | 0.092 |
| 2020 | 430300 | 0.699 | 2.799 | 1.197 | 15.66 | 5.608 | 11.94 | 2.854 | 14.03 | 10.64 | 0.968 | 0.075 |
| 2020 | 430400 | 0.701 | 2.124 | 0.68 | 14.4 | 6.499 | 11.84 | 2.616 | 14.04 | 10.17 | 0.925 | 0.068 |
| 2020 | 430500 | 0.689 | 2.039 | 0.291 | 13.91 | 6.486 | 10.61 | 2.795 | 12.52 | 9.694 | 0.778 | 0.064 |
| 2020 | 430600 | 0.701 | 1.807 | 0.331 | 14.83 | 6.224 | 10.97 | 3.103 | 13.15 | 10.58 | 1.465 | 0.054 |
| 2020 | 430700 | 0.703 | 2.125 | 0.421 | 14.5 | 6.268 | 11.05 | 3.096 | 13.97 | 10.4 | 1.341 | 0.067 |
| 2020 | 430800 | 0.731 | 1.752 | 0.369 | 14.06 | 5.022 | 9.822 | 2.799 | 11.7 | 9.956 | 0.527 | 0.068 |
| 2020 | 430900 | 0.695 | 2.374 | 0.425 | 14.32 | 5.953 | 10.79 | 2.995 | 12.58 | 9.998 | 0.674 | 0.057 |
| 2020 | 431000 | 0.716 | 2.009 | 0.261 | 14.79 | 6.148 | 10.4 | 2.44 | 14.33 | 10.3 | 1.024 | 0.076 |
| 2020 | 431100 | 0.714 | 1.914 | 0.323 | 14.48 | 6.272 | 10.53 | 3.046 | 13.92 | 9.928 | 0.689 | 0.079 |
| 2020 | 431200 | 0.701 | 2.25 | 0.284 | 14.85 | 6.127 | 10.58 | 2.39 | 11.2 | 9.916 | 0.695 | 0.069 |
| 2020 | 431300 | 0.703 | 2.043 | 0.412 | 14.12 | 5.947 | 10.6 | 3.158 | 13.12 | 9.993 | 1.333 | 0.066 |
| 2020 | 500000 | 0.709 | 2.906 | 0.806 | 14.77 | 8.072 | 13.73 | 2.684 | 15.75 | 10.49 | 1.145 | 0.132 |
| 2020 | 510100 | 0.696 | 3.786 | 0.944 | 15.79 | 7.326 | 13.75 | 2.929 | 15.43 | 11.29 | 0.997 | 0.006 |
| 2020 | 510300 | 0.696 | 2.085 | 0.593 | 14.07 | 5.761 | 10.81 | 2.909 | 8.868 | 10.26 | 1.119 | 0.046 |
| 2020 | 510400 | 0.71 | 2.756 | 0.546 | 14.01 | 4.682 | 10.16 | 2.853 | 11.61 | 11.14 | 2.998 | 0.198 |
| 2020 | 510500 | 0.69 | 1.803 | 0.476 | 13.67 | 6.23 | 11.05 | 2.615 | 11.78 | 9.944 | 1.077 | 0.064 |
| 2020 | 510600 | 0.707 | 2.605 | 0.905 | 13.93 | 5.946 | 11.79 | 3.301 | 12.39 | 10.59 | 1.398 | 0.083 |
| 2020 | 510700 | 0.703 | 2.738 | 1.023 | 14.24 | 6.27 | 12.01 | 2.969 | 11.79 | 10.47 | 0.959 | 0.053 |
| 2020 | 510800 | 0.703 | 1.402 | 0.25 | 12.52 | 5.694 | 10.07 | 2.794 | 9.913 | 9.618 | 0.829 | 0.218 |
| 2020 | 510900 | 0.693 | 1.944 | 0.203 | 13.17 | 5.884 | 9.572 | 3.291 | 12.22 | 9.876 | 0.885 | 0.093 |
| 2020 | 511000 | 0.695 | 1.807 | 0.455 | 12.53 | 6.005 | 10.56 | 2.736 | 10.32 | 9.96 | 1.473 | 0.104 |
| 2020 | 511100 | 0.707 | 1.92 | 0.525 | 12.21 | 5.852 | 10.82 | 2.676 | 10.88 | 10.23 | 1.734 | 0.043 |
| 2020 | 511300 | 0.703 | 1.553 | 0.516 | 12.06 | 6.578 | 11.42 | 2.777 | 11.04 | 9.622 | 0.863 | 0.092 |
| 2020 | 511400 | 0.709 | 1.921 | 0.642 | 12.44 | 5.834 | 10.35 | 2.856 | 11.98 | 10.08 | 1.297 | 0.066 |
| 2020 | 511500 | 0.7 | 2.112 | 0.282 | 14.17 | 6.312 | 10.45 | 2.647 | 10.72 | 10.12 | 1.797 | 0.029 |
| 2020 | 511600 | 0.7 | 1.481 | 0.151 | 11.94 | 6.122 | 9.726 | 3.414 | 12.37 | 9.755 | 1.151 | 0.126 |
| 2020 | 511700 | 0.698 | 0.225 | 0.225 | 12.73 | 6.481 | 10.43 | 2.625 | 10.51 | 9.782 | 1.095 | 0.047 |
| 2020 | 511800 | 0.718 | 1.981 | 0.57 | 14.48 | 5.028 | 10.88 | 3.544 | 9.219 | 10.29 | 1.738 | 0.183 |
| 2020 | 511900 | 0.666 | 1.36 | 0.061 | 11.96 | 5.897 | 9.338 | 3.074 | 11.7 | 9.459 | 0.64 | 0.225 |
| 2020 | 512000 | 0.703 | 1.216 | 0.207 | 13.5 | 5.826 | 9.458 | 3.207 | 11.41 | 9.812 | 1.054 | 0.044 |
| 2020 | 520100 | 0.695 | 3.549 | 1.249 | 15.27 | 6.395 | 13 | 10.7 | 14.15 | 10.66 | 1.301 | 0.158 |
| 2020 | 520200 | 0.693 | 1.983 | 0.227 | 14.54 | 5.715 | 10.04 | 10.94 | 14.28 | 9.664 | 2.119 | 0.142 |
| 2020 | 520300 | 0.701 | 2.331 | 0.373 | 14.31 | 6.494 | 11.6 | 10.58 | 12.72 | 9.713 | 1.309 | 0.129 |
| 2020 | 520400 | 0.673 | 2.068 | 0.255 | 14.63 | 5.51 | 10.06 | 11.19 | 11.51 | 9.38 | 1.404 | 0.131 |
| 2020 | 530100 | 0.708 | 3.123 | 1.233 | 14.51 | 6.741 | 13.46 | 2.532 | 12.01 | 11.03 | 1.105 | 0.145 |
| 2020 | 530300 | 0.705 | 1.508 | 0.221 | 12.67 | 6.357 | 10.57 | 3.072 | 8.332 | 9.881 | 1.871 | 0.102 |
| 2020 | 530400 | 0.706 | 2.318 | 0.24 | 15.28 | 5.416 | 9.968 | 3.132 | 7.541 | 10.91 | 2.964 | 0.088 |
| 2020 | 530500 | 0.682 | 1.076 | 0.212 | 12.57 | 5.493 | 9.981 | 3.176 | 9.516 | 9.538 | 0.524 | 0.137 |
| 2020 | 530600 | 0.68 | 0.805 | 0.091 | 12.1 | 6.233 | 9.684 | 2.813 | 6.623 | 9.119 | 0.751 | 0.128 |
| 2020 | 530700 | 0.709 | 1.502 | 0.54 | 13.19 | 4.832 | 10.19 | 2.363 | 5.933 | 9.555 | 0.769 | 0.185 |
| 2020 | 530800 | 0.695 | 1.071 | 0.117 | 13.09 | 5.483 | 9.292 | 3.088 | 8.483 | 9.388 | 0.814 | 0.122 |
| 2020 | 530900 | 0.694 | 0.82 | 0.133 | 13.45 | 5.42 | 9.375 | 2.801 | 8.546 | 9.431 | 1.141 | 0.12 |
